# Chiral, sequence-definable foldamer-derived macrocycles†

**DOI:** 10.1039/d1sc05021d

**Published:** 2021-11-10

**Authors:** Toyah M. C. Warnock, Sundaram Rajkumar, Matthew P. Fitzpatrick, Christopher J. Serpell, Paul Dingwall, Peter C. Knipe

**Affiliations:** School of Chemistry and Chemical Engineering, Queen's University Belfast David Keir Building Belfast BT9 5AG UK p.knipe@qub.ac.uk; Almac Group Ltd. 20 Seagoe Industrial Estate Craigavon BT63 5QD UK; School of Physical Sciences, University of Kent Ingram Building Canterbury Kent CT2 7NH UK

## Abstract

Nature's oligomeric macromolecules have been a long-standing source of inspiration for chemists producing foldamers. Natural systems are frequently conformationally stabilised by macrocyclisation, yet this approach has been rarely adopted in the field of foldamer chemistry. Here we present a new class of chiral cyclic trimers and tetramers formed by macrocyclisation of open-chain foldamer precursors. Symmetrical products are obtained *via* a [2 + 2] self-assembly approach, while full sequence control is demonstrated through linear synthesis and cyclisation of an unsymmetrical trimer. Structural characterisation is achieved through a combined X-ray and DFT approach, which indicates the tetramers adopt a near-planar conformation, while the trimers adopt a shallow bowl-like shape. Finally, a proof-of-concept experiment is conducted to demonstrate the macrocycles' capacity for cation binding.

## Abstract 2

Dipole-controlled pre-organization enables the cyclization of sequence-defined foldamers into macrocycles. The structure and properties of trimeric and tetrameric macrocycles are explored, and their ability to bind cationic guests is demonstrated.

## Graphical abstract

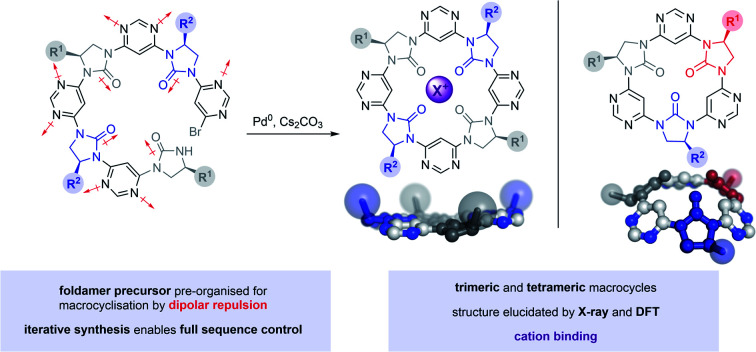

## Introduction

Nature frequently exploits macrocycles as functional molecules within living systems,^1–3^ and many of these and their derivatives have been exploited as therapeutics.^4–6^ Some of the most important drugs for human health are naturally-occurring macrocycles, including vancomycin and cyclosporin. The relative success of macrocyclic peptide drugs can be attributed to several factors including stability to degradation, cyclic constraint favouring the active conformation, and high membrane permeability. Inspired by macrocycles' use in Nature and potency in the clinic, chemists have developed numerous cyclisation strategies to stabilise the conformation of small bioactive peptides, including helix “stapling”^7^ and cross-linking to stabilise β-hairpin structures.^8^ Peptides have also been replaced entirely, with artificial folded molecules dubbed “foldamers”.^9^ Entirely abiotic macrocycles have also been used in applications outside biology, particularly in host-guest chemistry.^10^ Here, the pre-organisation of functionality within the macrocycle enables large binding constants and high guest-specificity.

**Fig. 1:**
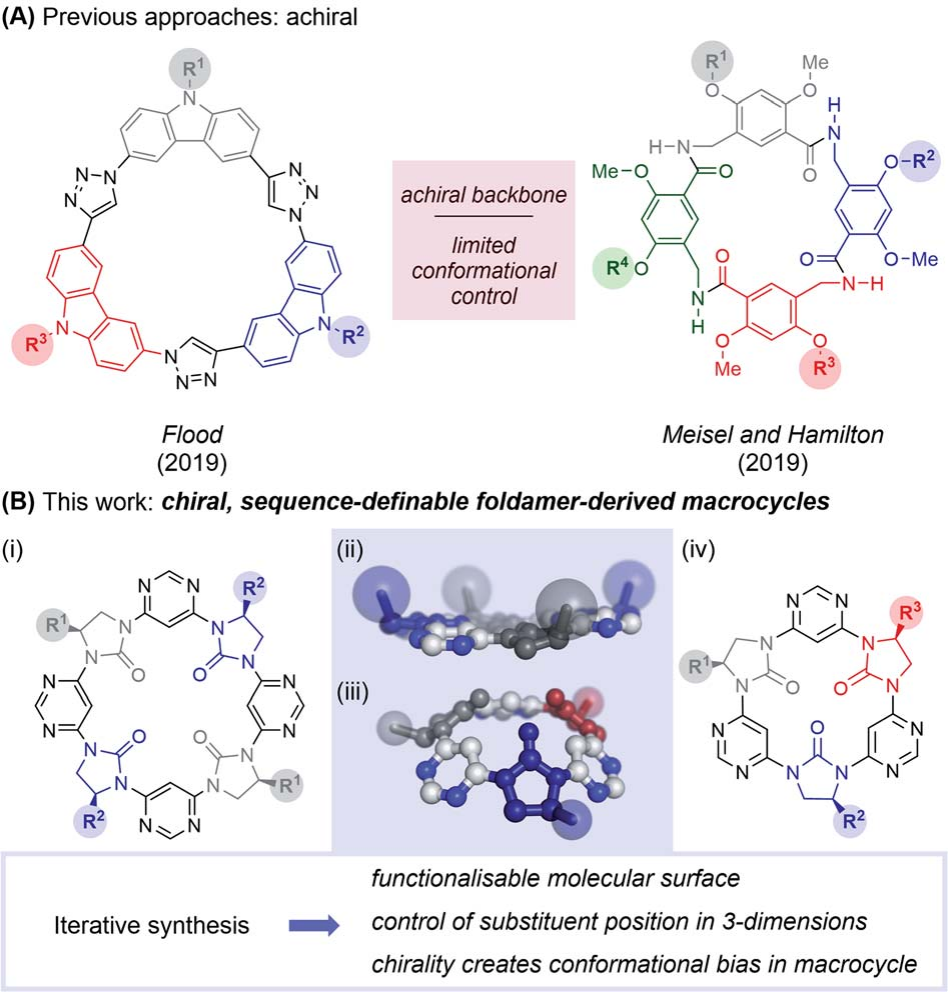
Sequence-defined chiral macrocyclic oligomers. (A) Recent examples of sequence-defined macrocyclic foldamers reported by Flood, and Hamilton and Meisel. The Flood macrocycles adopt an entirely planar conformation, forming 2-dimensional crystalline arrays, while those of Meisel and Hamilton adopt enantiomeric cavitand-like conformations under rapid exchange. (B) This study describes the synthesis, structure and cation-binding properties of a novel chiral foldamer-derived macrocycle. (i) General structure of tetrameric macrocycles; (ii) X-ray structure (top) and computed energy minimum (bottom) of tetrameric and trimeric macrocycles respectively; (iii) general structure of trimeric macrocycles. Sidechains are highlighted as coloured spheres.

Foldamers have been developed to mimic peptide secondary^11–14^ and tertiary structures,^15–17^ and have shown promise as therapeutic agents in their own right.^9^ However, the field of *foldamer-derived macrocycles* – analogous to macrocyclic peptides – remains in its infancy. Extant foldamer-derived macrocycles typically display a large degree of symmetry,^18–32^ probably due to synthetic convenience (a notable exception is in mixed peptide/foldamer^33–37^ and peptoid^38,39^ systems). However, Nature demonstrates that diversity (rather than uniformity) of structure is crucial in enabling the use of macrocycles across a range of functions. In the past year the first two examples of sequence-defined abiotic foldamer-derived macrocycles have emerged from the laboratories of Flood,^40^ who reported cyclic carbazole-triazole trimers and Meisel and Hamilton^41^ who synthesised cavitand-like molecular containers (Fig. 1). Here, we present our synthesis of semi and fully sequence-definable abiotic foldamer-derived macrocycles possessing a homochiral backbone, allowing the defined positioning of sidechains in three-dimensions.

## Results and discussion

### Synthesis of macrocycles

We have previously described a foldamer architecture where dipolar repulsion between adjacent pyrimidine and imidazolidin-2-one components leads to structures with a turn per monomer of ∼86°.^15^ The attempted synthesis of related pyridine-linked macrocycles by Meth-Cohn failed entirely, generating only oligomeric material,^42^ but we hypothesised that the dipole-mediated pre-organisation in our putative linear precursor could favour macrocyclisation over oligomerisation. Similar preorganisation towards macrocyclisation has previously been exploited by Gong in the self-assembly of oligobenzamide foldamers.^26,43^ We tested this hypothesis by first synthesising linear dimer 2a by iterative deprotection/Buchwald–Hartwig coupling steps from a chiral, amino alcohol-derived monomeric precursor (see ESI† for procedures). Removal of the *N-tert*-butyl protecting group on 1a under acidic conditions afforded dimer 2a in 81% yield. Both the *N*-terminally protected and *tert-*butyl deprotected intermediates 1a and 2a were crystalline solids, with the latter purified conveniently by trituration with hot hexanes.

**Scheme 1:**
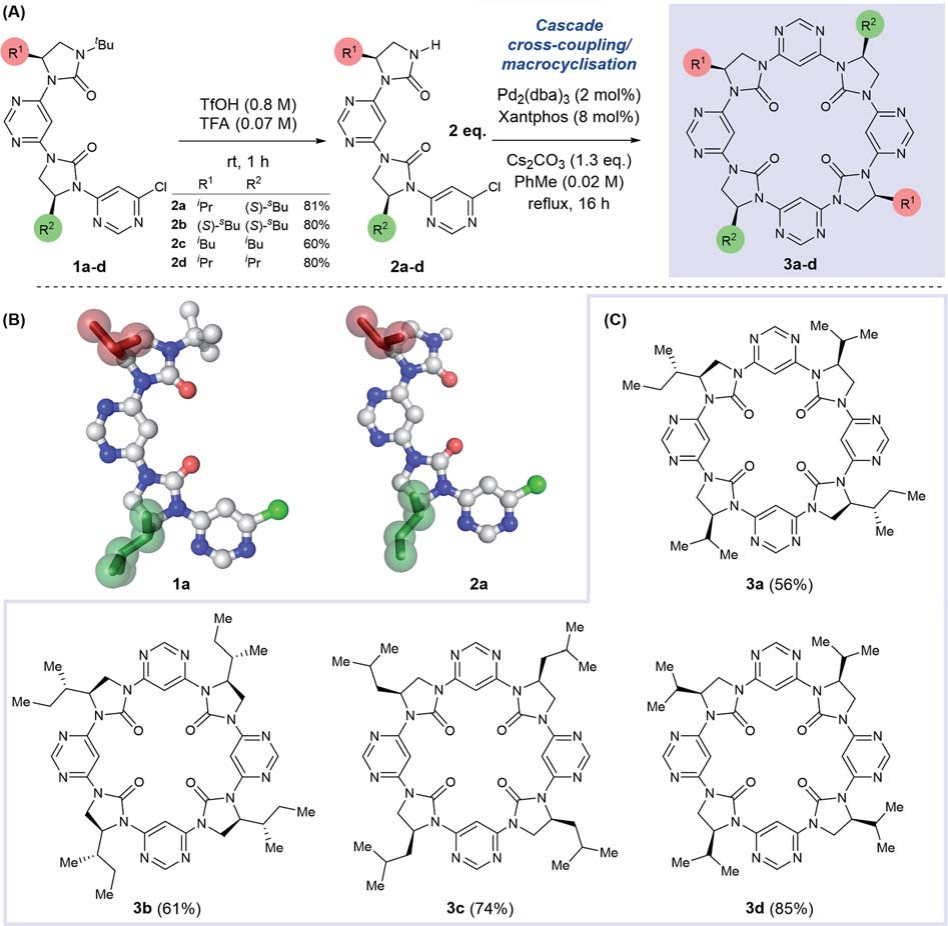
(A) Synthesis of tetrameric macrocycles *via N*-^*t*^Bu deprotection of dimer 1a–d to afford 2a–d, and cascade dimerisation-cyclisation under Buchwald–Hartwig conditions. (B) Single crystal X-ray structures of 1a and 2a, sidechains are highlighted in green and red. (C) Synthetic scope of tetrameric macrocycle formation.

Examination of their single crystal X-ray structures revealed that the expected dipole-opposed conformation was adopted, giving both molecules an overall crescent shape (Scheme 1B). We were therefore confident that treatment of dimer 2a under Buchwald–Hartwig cross-coupling conditions would lead directly to the *C*_2_-symmetrical macrocycle 3a, since the initial tetrameric product of homo-coupling would be pre-organised *via* dipolar repulsion for a second coupling reaction to generate the macrocycle. Thus, treatment of 2a with Pd_2_(dba)_3_, Xantphos and Cs_2_CO_3_ under reflux in toluene afforded macrocycle 3a in 56% isolated yield, with no evidence of the formation of larger macrocycles or polymeric material. With this synthetic strategy validated we proceeded to synthesise a further three dimeric precursor molecules 2b–d.^44^ Upon treatment under cross-coupling conditions all were converted to the corresponding *C*_4_-symmetrical macrocycles 3b–d in isolated yields of 61–85%.

**Scheme 2:**
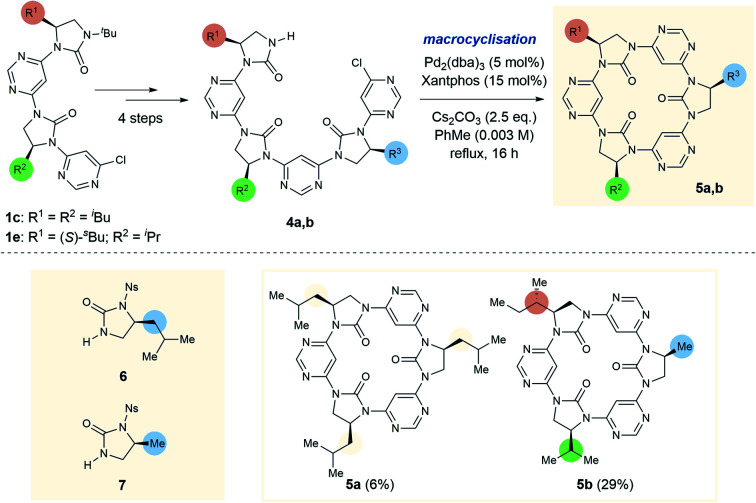
Synthesis of *C*_3_- (5a) and *C*_1_-symmetrical (5b) trimeric macrocycles. *Synthesis of4a*: (1) 6 (1.3 eq.), Pd_2_(dba)_3_ (5 mol%), Xantphos (15 mol%), K_2_CO_3_ (2.5 eq.), PhMe, reflux, 16 h, 73% brsm; (2) PhSH (1.5 eq.), K_2_CO_3_ (3 eq.), *N,N*-DMF, rt, 3 h, 90%; (3) 4,6-dichloropyrimidine (9 eq.), Pd_2_(dba)_3_ (5 mol%), Xantphos (15 mol%), K_2_CO_3_ (2.5 eq.), PhMe, reflux, 16 h, 62%; (4) TfOH:TFA 4:1 *v*:*v*, rt, 1 h, >99%. *Synthesis of4b*: (5) 7 (1.5 eq.), Pd_2_(dba)_3_ (5 mol%), Xantphos (15 mol%), K_2_CO_3_ (2.5 eq.), reflux, 16 h, 95%; (6) as step 2, 52%; (7) as step 3, 90%; (8) as step 4, >99%. 5b is an example of an entirely sequence-defined chiral foldamer-derived macrocycle.

To further test the limits of the macrocyclisation approach we synthesised linear trimers 4a and 4b (Scheme 2). While the cyclic tetramers were expected to be approximately planar and free from torsional strain, we anticipated that the trimers would be more strained, with the backbone forced out of the ideal planar conformation. To obtain the open-chain trimers it was necessary to add an additional pyrimidine and imidazolidin-2-one sub-unit to linear dimers 1c and 1e, which was achieved in four steps. In accordance with greater conformational strain, yields of the corresponding trimeric macrocycles were low relative to the tetrameric homologues, with 5a and 5b obtained in 6% and 29% yields respectively. Macrocycle 5b is especially noteworthy as it is entirely “sequence-defined”, with the cyclic ordering of the monomers predetermined by the order of monomer addition in the preparation of the linear precursor.

### Conformational studies

**Fig. 2:**
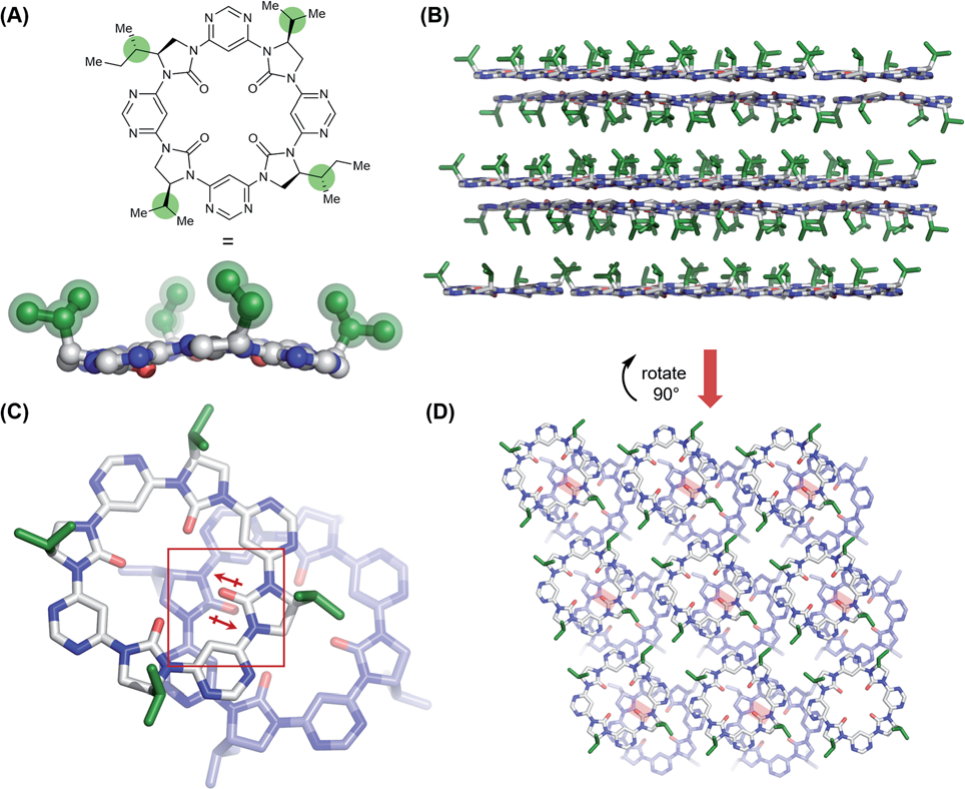
Single crystal X-ray structure of *C*_2_-symmetrical macrocycle 3a. Sidechains are highlighted in green. Sidechains cannot be identified beyond the γ-position due to disorder, so all are truncated to isopropyl. Solvent molecules and hydrogen atoms are omitted for clarity. (A) Single molecule view, showing controlled projection of side-chains from a single face; (B) crystal packing arrangement (viewed along crystalline *b*-axis) showing planes of macrocycles arranged back-to-back, with side-chains projected into the interplanar void; (C) stacking arrangement between macrocycles in adjacent planes (top plane – white; bottom plane – purple) displaying a dipole-opposed orientation (dipoles indicated in red); (D) crystal packing arrangement (viewed along the crystalline *a*-axis) showing the offset arrangement between adjacent planes of macrocycles (top plane – white; bottom plane –purple). Dipole-opposed interactions are indicated by red boxes.

**Fig. 3:**
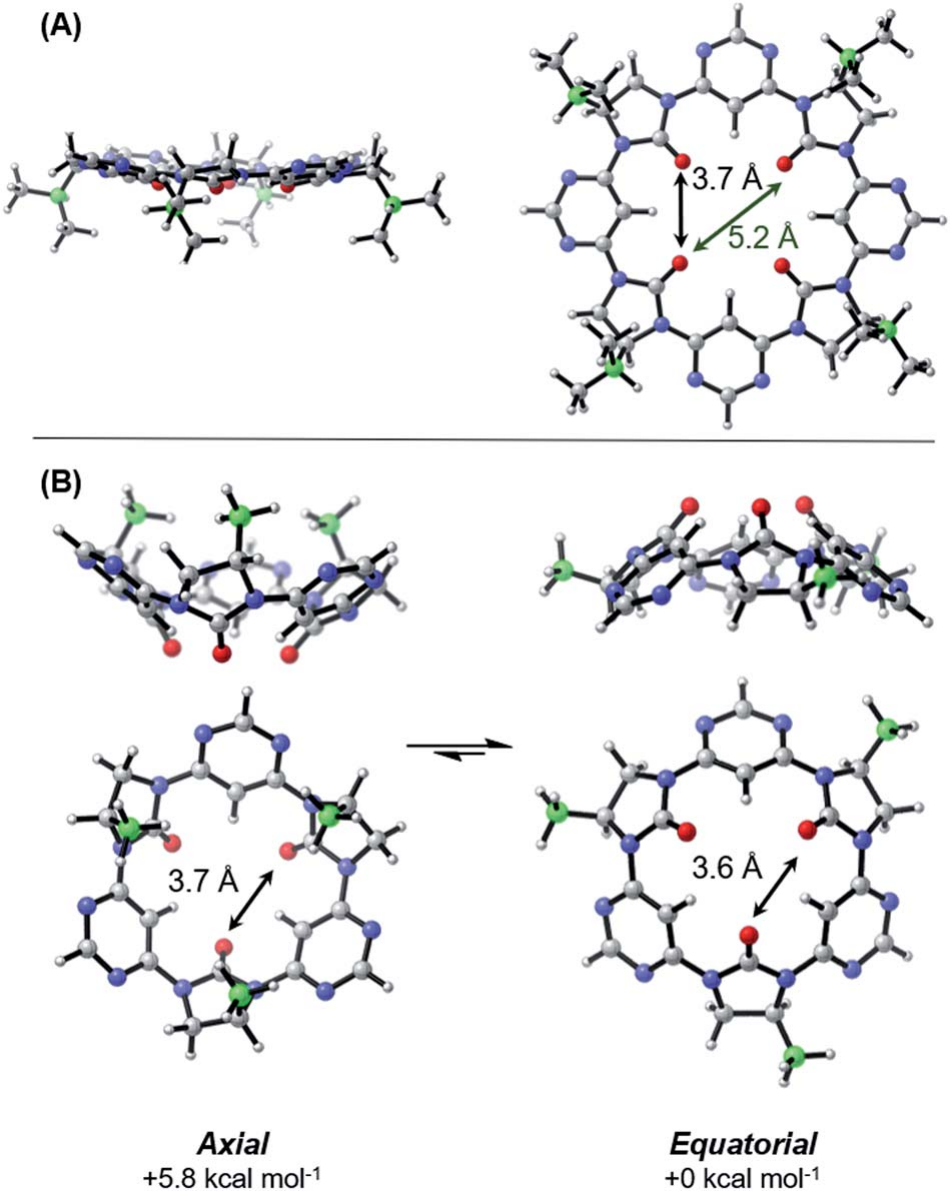
Computed lowest energy conformations of tetrameric (A) and trimeric (B) macrocycles. Averaged O–O interatomic distances are indicated. Level of theory: B3LYP/6-311+G(d,p)/GD3BJ/PCM_Toluene_//B3LYP/6-31G(d,p)/GD3BJ. Isopropyl and methyl sidechains are shown for the tetramer and trimer respectively.

In an effort to understand its conformational behavior, single crystals of macrocycle 3a were grown by vapour diffusion (Fig. 2A). Attempts to generate diffraction-quality crystals of the remaining macrocycles were unsuccessful. As expected, all four sidechains of 3a are projected from a single face of the macrocycle in a highly controlled manner. Inspection of the crystal packing reveals a layered, back-to-back stacking arrangement between planes of macrocycles (Fig. 2B–D), with the relative orientation of macrocycles between layers controlled in part by a dipole-opposed arrangement between adjacent imidazolidin-2-ones (Fig. 2C). Due to disorder and weak diffraction the X-ray data were insufficient to gain further structural insights, so we proceeded to explore the macrocycles' conformational behavior by DFT (Fig. 3).

The lowest energy conformation of the tetramer was found to be a shallow bowl, with all C=O groups puckered outwards from the same face of the macrocycle, and a transverse O–O distance of 5.2 Å. Two low energy conformers were identified for the trimer, corresponding to a macromolecular “ring-flip”, in which the sidechain substituents are placed in *pseudo*-axial or *pseudo*-equatorial positions (Fig. 3B). The equatorial conformer is lower in energy by 5.8 kcal mol^−1^ and represents the global energy minimum. No “mixed” conformers (in which one imidazolidin-2-one is puckered in an opposing direction to the others) are identified as minima. The less-planar structure of the trimer relative to the tetramer is also supported by ^1^H NMR data: the inward-pointing pyrimidine hydrogens in tetramers 3a–3d appear far downfield (∼9.9 ppm), likely due to the deshielding effect of the proximal oxygen lone-pairs, whereas the equivalent peaks in the trimers 5a and 5b appear at ∼8.7 ppm indicating that these hydrogens are subject to the deshielding effect of the lone pairs to a much lesser degree.

#### Guest binding

**Fig. 4:**
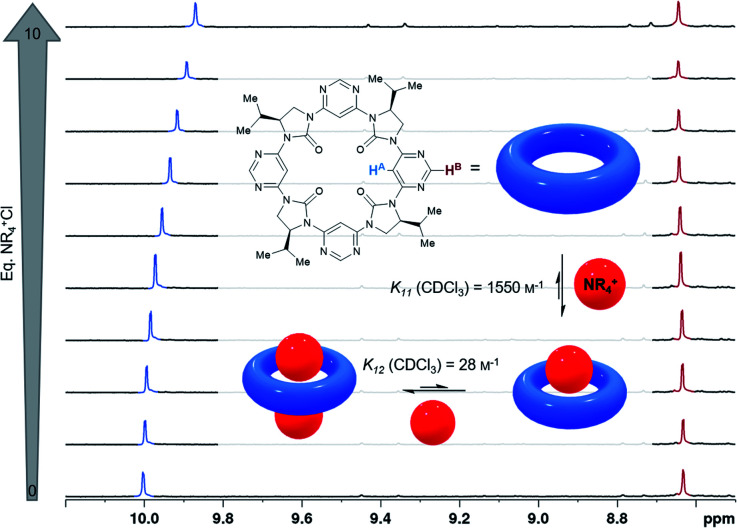
Binding of hexadecyltrimethylammonium chloride (depicted as a red sphere) by *C*_4_-symmetrical macrocycle 3d (blue torus) determined by ^1^H NMR titration (CDCl_3_, 600 MHz). Atoms H^A^ and H^B^ are highlighted and the corresponding ^1^H NMR signals are indicated in blue and red respectively. For full details refer to the ESI †.

The structural and computational data were indicative of the tetrameric macrocycles possessing a central pore with the four urea carbonyl groups directed towards its centre. We anticipated that cations would be well-stabilised within the macrocycle, as had already been observed with Cs^+^ during the isolation of 3a. We were particularly interested in the binding of ammonium cations since methylated lysines form part of the epigenetic histone code, and selective methods to probe them are therefore of importance.^45–47^ The binding of macrocycle 3d to hexadecyltrimethylammonium chloride was examined by ^1^H NMR titration in CDCl_3_ (Fig. 4).^48^ Upon treatment with the ammonium salt, H^B^ displays a small downfield shift while H^A^ displays a larger upfield shift. The greater magnitude shift of H^A^ is consistent with our assumption that binding occurs to the inner edge of the macrocycle, in closer proximity to H^A^ than H^B^, likely in the manner of “perching” complexes described by Cram, and mediated by CH⋯O hydrogen bonding between the ammonium α-C–H bonds and imidazolidin-2-one carbonyl groups.^49–51^ The data are suggestive of 1:2 host:guest complex formation, with fitted equilibrium constants of *K*_11_ = 1550 m^−1^ and *K*_12_ = 28 m^−1^. The ability to alter the size and side-chains of the macrocycle raises the prospect of tailoring the selectivity of this cation binding to the desired guest in future studies.

Lastly, an attempt was made to achieve diasteroselective recognition of a chiral guest using dibenzoyl tartaric acid, chosen for its solubility in CDCl_3_, availability in both enantiomeric forms, and ability to form hydrogen bonds to the acceptor-rich environment of the macrocycle interior. However, no difference in binding was observed between enantiomers (see ESI†). We attribute this to the ability of guests to bind the unsubstituted face of the macrocycles, where little stereo-differentiation is feasible; studies are ongoing to synthesise macrocycles incorporating substituents on both faces to address this issue, either by use of *C*_2_-symmetrical 1,2-disubstituted diamines, or through the use of alternating (*R*)- and (*S*)-configured imidazolidine-2-ones in adjacent monomers.

## Conclusions

To summarise, we have synthesised and examined the properties of six chiral foldamer-derived macrocycles. The strategy enables their synthesis in entirely sequence-defined manner when required, through synthesis of a linear precursor, or in a convenient semi-defined manner through generation of linear dimers which undergo spontaneous cyclisation upon coupling to form the tetramer. DFT indicates the trimeric macrocycles adopt a bowl-like shape with substituents in a *pseudo*-equatorial position, while tetramers adopt a planar conformation, as demonstrated by single crystal X-ray diffraction, and bind metal and ammonium cations. The high level of conformational control means these macrocycles are an excellent platform for the controlled positioning of sidechain groups. Current work is ongoing to examine applications of the macrocycles in molecular recognition and catalysis, and to develop a solid-supported second-generation synthetic approach.

## Data availability

Crystallographic data for 1a, 2a, 2d and 3a has been deposited at the CCDC under accession numbers 2057484, 2057483, 2057482 and 2057486 respectively, and can be obtained from http://www.ccdc.cam.ac.uk. DFT data for this paper can be found at https://pure.qub.ac.uk with DOI: 10.17034/8953afcf-c4b8-41f4-8b4b-176fd07a8956.

## Author contributions

TMCW: data curation; formal analysis; investigation; methodology; validation; visualization; writing – original draft; writing – review & editing. SR: data curation; formal analysis; investigation; methodology; writing – review & editing. MPF: investigation; writing – review & editing. CJS: formal analysis (XRD); writing – review & editing; PD: formal analysis; investigation (DFT); visualization; writing – review & editing. PCK: conceptualization; data curation; formal analysis; funding acquisition; methodology; project administration; resources; supervision; visualization; writing – original draft; writing – review & editing.

## Conflicts of interest

There are no conflicts to declare.

## Supplementary Material

SC-012-D1SC05021D-s001

SC-012-D1SC05021D-s002
