# Supplementary material for: Chiral, sequence-definable foldamer-derived macrocycles
Source: Chem Sci. 2021 Nov 10;12(47):15632–6. doi: 10.1039/d1sc05021d (PMC8654020; doi:10.1039/d1sc05021d)
Supplement: SC-012-D1SC05021D-s001 [file SC-012-D1SC05021D-s001.pdf]

Electronic Supplementary Material (ESI) for Chemical Science.
This journal is © The Royal Society of Chemistry 2021

Supplementary Information

Chiral, Sequence-Definable Foldamer-Derived Macrocycles

Toyah M. C. Warnock,[1] Sundaram Rajkumar,[2] Matthew P. Fitzpatrick,[1] Christopher J. Serpell,[3] Paul Dingwall[1] and Peter C. Knipe[1*]

1. *School of Chemistry and Chemical Engineering, Queen's University Belfast, David Keir Building, Belfast, BT9 5AG, UK*
2. *Almac Group Ltd., 20 Seagoe Industrial Estate, Craigavon, BT63 5QD, UK*
3. *School of Physical Sciences, Ingram Building, University of Kent, Canterbury, Kent, CT2 7NH, UK*

Contents

1 General Experimental 3
1.1 Naming and Numbering of Compounds 3
1.2 Solvents and Reagents 3
1.3 Chromatography 3
1.4 Spectroscopy 3
1.5 Crystallography 4
2 Experimental Procedures and Characterization Data 5
2.1 General Experimental Procedures 5
2.2 Overall Synthetic Scheme towards Dimers 1a-1e 6
2.3 Synthesis of Monomers 7
2.3.1 Isoleucine Series 7
2.3.2 Valine Series 9
2.3.3 Leucine Series 12
2.3.4 Alanine Series 14
2.4 Synthesis of Acyclic Dimers and Trimers 16
2.5 Synthesis of Macrocycles 30
3 Ammonium Salt NMR Titration 37
3.1 Host and Guest Solution Preparation 37
3.2 NMR Titrations 37
3.3 Fitting 38
4 X-Ray Crystallography 42

4.1 *t*-Bu Protected Dimer 1a (CCDC #2057484) 42
4.1.1 Crystal Data 42
4.1.2 Data Collection 43
4.1.3 Refinement 43
4.2 Dimer 2a (CCDC #2057483) 43
4.2.1 Crystal Data 44
4.2.2 Data Collection 44
4.2.3 Refinement 45
4.3 Dimer 2d (CCDC #2057482) 45
4.3.1 Crystal Data 45
4.3.2 Data Collection 46
4.3.3 Refinement 46
4.4 Macrocyclic Tetramer 3a (CCDC #2057486) 47
4.4.1 Crystal Data 47
4.4.2 Data Collection 48
4.4.3 Refinement 48
5 Density Functional Theory Calculations 49
5.1 Trimer Summary (See Figure 3B) 49
5.2 Tetramer Summary (See Figure 3a) 50
6 Supplementary References 51
7 NMR Spectra 52
8 Appendix – Coordinates for DFT-Optimized Geometries 162
8.1 Trimer Coordinates (Figure 3B) 162
8.2 Tetramer Coordinates (Figure 3A) 166

1 General Experimental

1.1 Naming and Numbering of Compounds

Systematic compound names are those generated by ChemBioDraw™ Ultra version 15.1.0.144 (Perkin Elmer) following IUPAC nomenclature.

1.2 Solvents and Reagents

Reactions were carried out under a nitrogen atmosphere in oven-dried glassware unless otherwise stated. Standard inert atmosphere techniques were used in handling all air- and moisture-sensitive reagents. Where necessary toluene and DMF (from commercial sources) were degassed prior to use by sparging with argon or nitrogen (15 min). Other solvents and reagents were used directly as received from commercial suppliers.

1.3 Chromatography

Flash column chromatography was carried out using Fluorochem 60 40-63 micron silica gel. Thin-layer chromatography was carried out using Merck Kieselgel 60 F254 (230-400 mesh) fluorescent treated silica, visualized under UV light (254 nm) or by staining with aqueous potassium permanganate solution, ninhydrin or ceric ammonium molybdate solutions.

1.4 Spectroscopy

^{1}H and ^{13}C NMR spectra were recorded using a Bruker 600, 400 or 300 MHz spectrometer running TopSpin™ software and are quoted in ppm for measurement against tetramethylsilane. Where no tetramethylsilane was present, spectra are referenced relative to the residual non-deuterated solvent peaks. Unless otherwise stated spectra were acquired at 298 K. Topspin™ was used for processing and viewing NMR data. Chemical shifts (δ) are given in parts per million (ppm), and coupling constants (*J*) are given in Hertz (Hz). The ^{1}H NMR spectra are reported as follows: δ / ppm (number of protons, multiplicity, coupling constant *J* / Hz (where appropriate), assignment (where known)). Multiplicity is abbreviated as follows: s = singlet, br = broad, d = doublet, t = triplet, q = quartet, m = multiplet, app = apparent. The numbering scheme used for NMR assignment is arbitrary and does not follow any particular convention. The ^{13}C NMR spectra are reported in δ / ppm. Where necessary or appropriate, two-dimensional (COSY, HSQC, HMBC, NOESY or ROESY) NMR experiments were used to assist the assignment of signals in the ^{1}H and ^{13}C NMR spectra. In some cases, complete assignment of spectra was not possible (in particular, aromatic CHs corresponding to multiple phenyl groups overlapped significantly); in these cases only a partial assignment is reported.

Infra-red (IR) spectra were recorded on a Perkin-Elmer Spectrum 100 FT-IR spectrometer equipped with a Perkin-Elmer Universal ATR Sampling Accessory. Samples were deposited on the ATR accessory as a thin film. Only selected maximum absorbances (ν_{max}) of the most intense peaks are reported (cm^{-1}).

High resolution mass spectra (HRMS) were recorded by Analytical Services and Environmental Projects (ASEP) at Queen's University Belfast on a Waters LCT Premier ToF mass spectrometer using the electrospray ionisation (ESI) technique.

Optical rotations were recorded at the sodium D-line (589 nm) using a Perkin Elmer 341 polarimeter at a temperature of 20 °C and are reported in degrees using concentrations (*c*) in g·100 mL^{-1}. Reported values are the average of eight readings.

1.5 Crystallography

Low temperature[1] single crystal X-ray diffraction studies for **1a**, **1d** and **2a** were carried out using CuK_{α} radiation on an Agilent Supernova diffractometer equipped with an area detector and graphite monochromator. X-ray diffraction studies for **3a** were conducted using CuK_{α} on a Rigaku 007HF equipped with Varimax confocal mirrors and an AFC11 goniometer and HyPix 6000 detector, at the National Crystallography Service in the University of Southampton. Raw frame data were reduced using CrysAlisPRO[2] solved using Superflip.[3] Full-matrix least-squares refinement of the structures were carried out using CRYSTALS.[4,5] Full refinement details are given in the supplementary material (CIF). CCDC 2057484 (**1a**), 2057483 (**2a**), 2057482 (**2d**), and 2057486 (**3a**) contain the supplementary crystallographic data for this paper. These data are provided free of charge by The Cambridge Crystallographic Data Centre and copies can be obtained free of charge *via* www.ccdc.cam.ac.uk/data_request/cif.

2 Experimental Procedures and Characterization Data

2.1 General Experimental Procedures

General Procedure A (*N*-Nosyl Deprotection)

To a stirred, room-temperature suspension of *N*-nosyl urea (1.0 eq.) and K_2CO_3 (3.0 eq.) in anhydrous *N*,*N*-DMF (*ca.* 0.1 M) was added thiophenol (1.5 eq.). The rapid development of a deep orange colour was invariably observed upon the addition of thiophenol. After complete consumption of the *N*-nosyl-protected starting material by TLC analysis, the reaction mixture was diluted with ethyl acetate and washed with $NaHCO_3$ (sat. aq., *ca*. 10 mL/mmol urea). The aqueous layer was extracted with ethyl acetate (*ca*. 2 x 10 mL/mmol urea). The combined organic extracts were washed well with water (*ca*. 4 x 10 mL/mmol urea) to remove any remaining *N*,*N*-DMF. The organic layer was dried over anhydrous magnesium sulfate, filtered, and concentrated *in vacuo*. The crude product was then purified by flash column chromatography.

General Procedure B (Palladium-Catalysed Coupling of Deprotected Ureas with Aryl Halides)

This reaction was carried out by analogy to a literature procedure.[6] To a sealed tube equipped with a magnetic stir bar was added deprotected urea (1.0 eq.), aryl halide (5.0-9.0 eq.), freshly recrystallized $Pd_2(dba)_3$ (5-10 mol%), and Xantphos (15-30 mol%). Anhydrous toluene (*ca.* 0.1 M) was added to the flask, and the resulting suspension was degassed by sparging with nitrogen gas for 15-30 min. Cs_2CO_3 or K_2CO_3 (2.5 eq.) was then added in one portion to the flask, and the reaction mixture was heated to reflux under a nitrogen atmosphere. If after 16 h the reaction had not reached completion, a second portion of $Pd_2(dba)_3$ (5.0 mol%) and Xantphos (15 mol%) were added. After complete consumption of the urea starting material by TLC analysis, the reaction was cooled to room temperature, diluted with dichloromethane (*ca*. 20 mL/mmol deprotected urea) and filtered over Celite®. The crude product was then purified by flash column chromatography.

2.2 Overall Synthetic Scheme towards Dimers 1a-1e

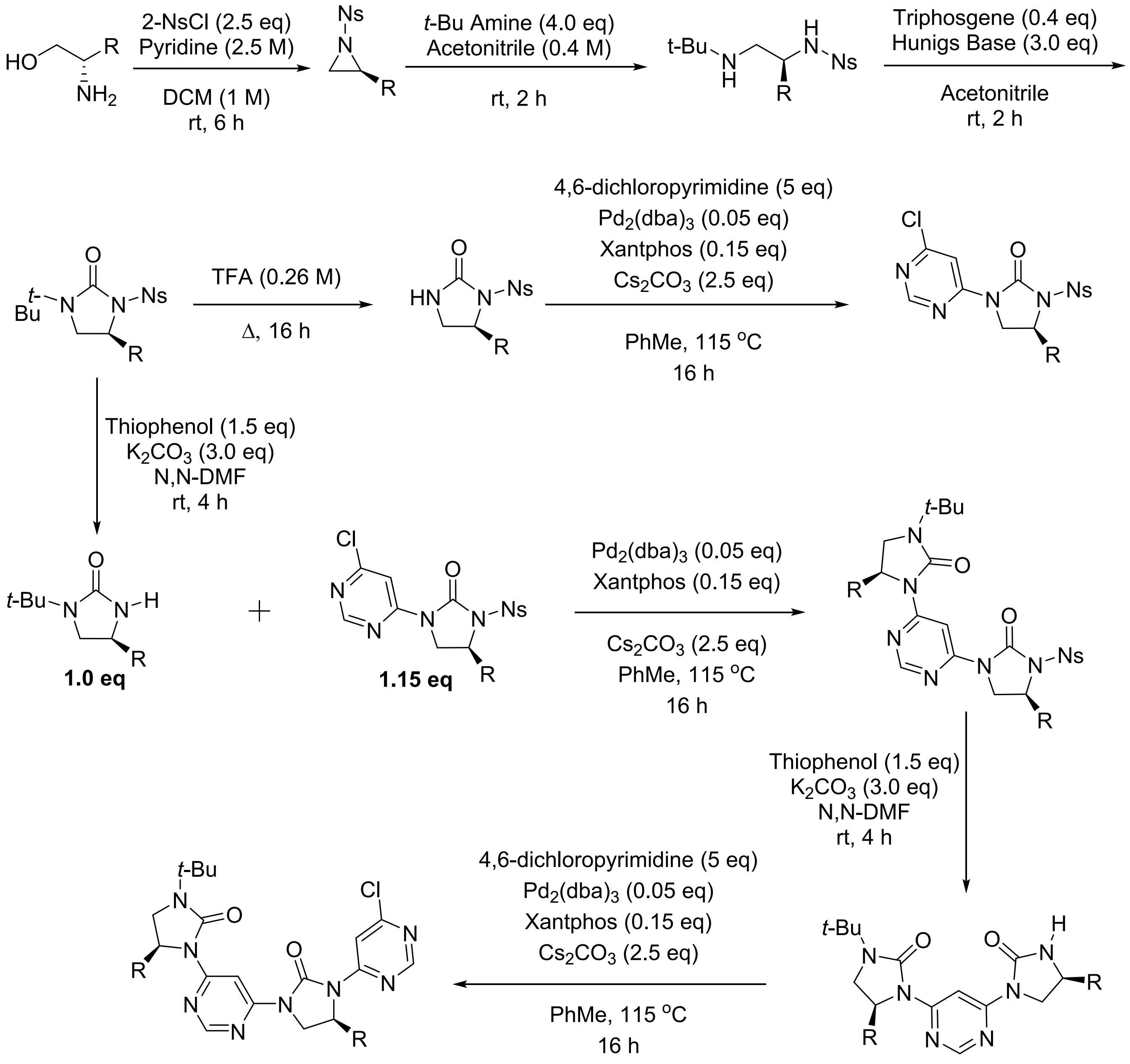

Scheme S1. General synthetic scheme towards dimers **1a-e**.

2.3 Synthesis of Monomers

2.3.1 Isoleucine Series

(*S*)-2-((*S*)-*sec*-butyl)-1-((2-nitrophenyl)sulfonyl)aziridine (S1)

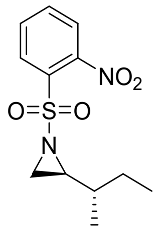

2-Nitrobenzenesulfonyl chloride (12.67 g, 57.18 mmol) was added in five portions to a stirred 0 °C solution of L-isoleucinol (3.00 g, 22.87 mmol) and pyridine (9.4 mL) in dichloromethane (23 mL). The reaction was stirred vigorously and then allowed to warm to room temperature. After 24 h, the volatiles were removed *in vacuo*. The concentrated reaction mixture was then taken up in diethyl ether (80 mL), and the organic layer was washed with HCl (1 M aq.) until the aqueous washings were acidic (approximately 6 x 40 mL). KOH (2 M aq., 160 mL) was added to the organic layer, and the resulting biphasic mixture was stirred vigorously for 6 h. The layers were separated, and the organic layer washed with KOH (2 M aq., 2 x 40 mL). The organic layer was dried over anhydrous magnesium sulfate, filtered, and concentrated *in vacuo*. Purification by flash column chromatography (silica gel, petrol:ethyl acetate, 5:1 → 2:1) afforded the aziridine **S1** (5.04 g, 78%) as a pale yellow oil.* δ_H (400 MHz, $CDCl_3$): 8.20 (1H, app. dd, *J* 7.0, 1.6), 7.78-7.70 (3H, m), 2.89-2.81 (2H, m), 2.30 (1H, d, *J* 4.7), 1.55-1.47 (1H, m), 1.40-1.31 (1H, m), 1.29-1.21 (1H, m), 0.93-0.87 (6H, m); δ_C (101 MHz, $CDCl_3$): 148.8, 134.5, 132.1, 132.0, 131.3, 124.3, 46.4, 36.8, 35.2, 27.2, 15.4, 11.0; HRMS (ESI+): found 323.0466; $C_{12}H_{16}N_2O_4SK$, $[M+K]^+$ requires 323.0468; v_{max} (thin film): 3093.7, 2963.2, 2929.7, 2877.5, 1591.6, 1543.1, 1461.1, 1405.2, 1367.9, 1330.7, 1237.5, 1162.9, 1058.6, 995.2, 931.8, 868.5, 779.0, 752.9, 693.3 cm^{-1}; $[\alpha]_D^{20}$ +105.2 (*c* = 1.1, $CHCl_3$).

***N*-((2*S*,3*S*)-1-(*tert*-butylamino)-3-methylpentan-2-yl)-2-nitrobenzenesulfonamide (S2)**

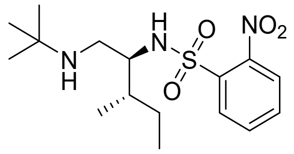

To a stirred, room temperature solution of *N*-nosyl-protected aziridine **S1** (5.04 g, 17.74 mmol) in acetonitrile (43 mL) was added *tert*-butylamine (7.5 mL, 70.96 mmol) in one portion. After 2 h the reaction was complete (TLC: petrol:ethyl acetate 1:1), and the volatiles were removed *in vacuo*. The crude residue was purified by flash column chromatography (silica gel, petrol:ethyl acetate 3:1 → 1:1 → pure ethyl acetate) to afforded the product **S2** (5.97 g, 94%) as a yellow oil crystalline oil. δ_H (400 MHz, $CDCl_3$): 8.14-8.12 (1H, m), 7.86-7.84 (1H, m), 7.74-7.68 (2H, m), 3.29-3.25 (1H, m), 2.55 (1H, dd, *J* 12.0, 6.8), 2.47 (1H, dd, *J* 12.0, 4.1), 1.70-1.60 (1H, m), 1.58-1.48 (1H, m), 1.18-1.07 (1H, m), 0.89-0.85 (15H, m); δ_C (101 MHz, $CDCl_3$): 149.0, 135.4, 133.2, 132.8, 130.7, 125.3, 60.2, 50.0, 42.5, 37.6, 29.0, 25.7, 15.0, 11.8; HRMS (ESI+): found 358.1786; $C_{16}H_{28}N_3O_4S$, $[M+H]^+$ requires 358.1801; v_{max} (thin film): 3306.1, 3101.1, 2959.5, 2870.1, 1595.3, 1543.1, 1461.1, 1364.2, 1297.1, 1215.1, 1170.4, 1073.5, 1021.3, 985.4, 853.6, 779.0, 738.0, 704.5 cm^{-1}; $[\alpha]_D^{20}$ –107.8 (*c* = 1.0, $CHCl_3$).

* It has been previously reported that *N*-nosyl-protected aziridines are prone to polymerisation upon standing.[22] Aziridine **S1**, however, was indefinitely stable when stored as a dilute solution in DCM.

(*S*)-4-((*S*)-*sec*-butyl)-1-(*tert*-butyl)-3-((2-nitrophenyl)sulfonyl)imidazolidin-2-one (S3)

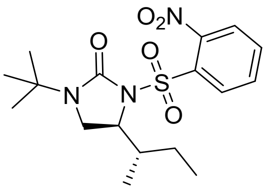

A solution of triphosgene (1.95 g, 6.56 mmol) in acetonitrile (50 mL) was added over 2 h *via* syringe pump to a stirred, room temperature solution of the diamine **S2** (5.97 g, 16.41 mmol) and Hünig's base (8.4 mL, 49.22 mmol) in acetonitrile (100 mL). After a further 30 min, the reaction mixture was concentrated *in vacuo*. The crude residue was taken up in dichloromethane (70 mL), and washed with HCl (30 mL, 1 M aq.). The layers were separated, and the aqueous phase was further extracted with dichloromethane (2 x 50 mL). The combined organic extracts were dried over anhydrous magnesium sulfate, filtered, and concentrated *in vacuo*. The crude residue was purified by trituration from hot hexane to afforded di-protected urea **S3** (5.55 g, 88%) as a brown crystalline solid. δ_H (400 MHz, $CDCl_3$): 8.43-8.41 (1H, m), 7.74-7.71 (3H, m), 4.28-4.26 (1H, m), 3.61 (1H, app. t, *J* 9.2), 3.27 (1H, app. dd, *J* 9.2, 1.7), 2.13-2.03 (1H, m), 1.48-1.38 (1H, m), 1.27 (9H, s), 1.23-1.16 (1H, m), 1.01 (3H, d, *J* 6.5), 0.98 (3H, d, *J* 7.3); δ_C (101 MHz, $CDCl_3$): 153.0, 147.9, 134.8, 134.2, 132.9, 132.0, 124.1, 57.8, 54.4, 42.1, 39.5, 27.4, 25.5, 12.2, 11.9; HRMS (ESI+): found 422.1159; $C_{17}H_{25}N_3O_5SK$, $[M+K]^+$ requires 422.1152; v_{max} (thin film): 3093.7, 2963.2, 2933.4, 2877.5, 1718.3, 1587.8, 1539.4, 1461.1, 1408.9, 1364.2, 1274.7, 1248.7, 1162.9, 1125.7, 1025.0, 998.9, 902.0, 756.6, 726.8 cm^{-1}; $[\alpha]_D^{20}$ +361.0 (c = 1.0, $CHCl_3$).

(*S*)-4-((*S*)-*sec*-butyl)-1-(*tert*-butyl)imidazolidin-2-one (S4)

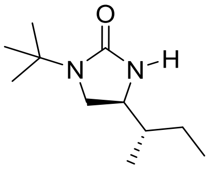

Prepared according to **General Procedure A** using di-protected urea **S3** (3.50 g, 9.13 mmol), thiophenol (1.29 mL, 13.69 mmol), K_2CO_3 (3.79 g, 27.39 mmol) and *N*,*N*-DMF (46 mL). Reaction time = 2 h. Purification by flash column chromatography (silica gel, petrol:ethyl acetate 10:1 → 5:1 → 2:1 (product)) afforded **S4** (1.61 g, 89%) as an off-white crystalline solid. Product **S4** is not visible under UV light and must be stained with ceric ammonium molybdate. δ_H (600 MHz, $CDCl_3$): 4.44 (1H, br. s, N-H), 3.46 (1H, app. t, *J* 8.6), 3.34-3.30 (1H, m), 3.08 (1H, app. t, *J* 8.3), 1.47-1.42 (2H, m), 1.35 (9H, s), 1.15-1.07 (1H, m), 0.90 (3H, t, *J* 7.1), 0.84 (3H, d, *J* 6.4); δ_C (151 MHz, $CDCl_3$): 162.3, 54.1, 52.9, 47.4, 39.4, 27.7, 25.6, 14.2, 11.0; HRMS (ESI+): found 199.1087; $C_{11}H_{23}N_2O$, $[M+H]^+$ requires 199.1810; v_{max} (thin film): 3209.2, 3086.2, 2959.5, 2870.1, 1684.8, 1483.5, 1416.4, 1360.5, 1293.4, 1259.8, 1230.0, 1148.0, 991.5, 928.1, 834.9, 767.8, 685.8 cm^{-1}; $[\alpha]_D^{20}$ +4.6 (c = 1.3, $CHCl_3$).

(*S*)-5-((*S*)-*sec*-butyl)-1-((2-nitrophenyl)sulfonyl)imidazolidin-2-one (S5)

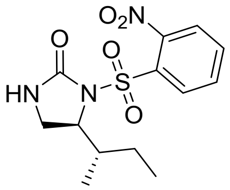

At room temperature, trifluoroacetic acid (30 mL) was added to di-protected urea **S3** (3.00 g, 7.82 mmol). The reaction mixture was stirred and then heated to reflux at 82°C for 16 h. When TLC analysis showed all starting material had been consumed, the reaction mixture was cooled to room temperature, and excess trifluoroacetic acid was removed by running a compressed air line over the solution for 1 h. The resulting crude brown residue was taken up in dichloromethane (60 mL) and washed with $NaHCO_3$ (sat. aq. 30 mL) The layers were separated, and the aqueous layer was further extracted with dichloromethane (2 x 40 mL). The combined organic extracts were dried over anhydrous magnesium sulfate, filtered, and concentrated *in vacuo*. The crude solid was purified by trituration from hot hexane to

afforded the *tert*-butyl deprotected product **S5** (2.31 g, 90%) as an orange-brown crystalline solid. δ_H (400 MHz, $CDCl_3$): 8.43 (1H, d, *J* 4.1), 7.75-7.73 (3H, m), 5.03 (1H, br. s, N-H), 4.57-4.55 (1H, m), 3.65 (1H, app. t, *J* 9.3), 3.30 (1H, dd, *J* 9.4, 2.2), 2.19-2.08 (1H, m), 1.47-1.38 (1H, m), 1.27-1.14 (1H, m), 1.06-0.97 (6H, m); δ_C (151 MHz, $CDCl_3$): 155.5, 148.0, 135.0, 134.6, 132.3, 131.9, 124.3, 61.8, 39.4, 39.2, 25.4, 12.2, 11.9; HRMS (ESI+): found 366.0506; $C_{13}H_{17}N_3O_5SK$, $[M+K]^+$ requires 366.0526; ν_{max} (thin film): 3369.5, 3063.9, 2963.2, 2929.7, 1751.8, 1714.6, 1587.8, 1550.6, 1453.7, 1420.1, 1356.8, 1300.8, 1259.8, 1211.4, 1162.9, 1125.7, 1073.5, 849.8, 793.9, 730.6 cm^{-1}; $[\alpha]_D^{20}$ +418.2 (*c* = 0.2, $CHCl_3$).

(*S*)-4-((*S*)-*sec*-butyl)-1-(6-chloropyrimidin-4-yl)-3-((2-nitrophenyl)sulfonyl)imidazolidin-2-one (S6)

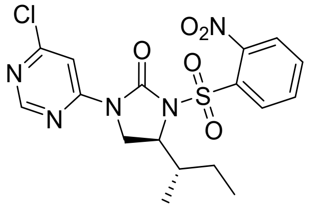

Prepared according to **General Procedure B** using deprotected urea **S5** (1.00 g, 3.05 mmol), 4,6-dichloropyrimidine (2.27 g, 15.25 mmol), $Pd_2(dba)_3$ (0.14 g, 0.15 mmol), Xantphos (0.26 g, 0.46 mmol), toluene (31 mL), and K_2CO_3 (1.05 g, 7.63 mmol). Reaction time = 16 h. Purification by flash column chromatography (silica gel, petrol:ethyl acetate, 10:1 → 8:1 → 1:1) afforded the chloropyrimidine **S6** (1.25 g, 93%) as a pale yellow solid. δ_H (600 MHz, $CDCl_3$): 8.66 (1H, s), 8.49-8.48 (1H, m), 8.09 (1H, s), 7.83-7.78 (3H, m), 4.61-4.60 (1H, m), 4.12-4.05 (2H, m), 2.22-2.18 (1H, m), 1.54-1.48 (1H, m), 1.35-1.29 (1H, m), 1.03 (3H, t, *J* 7.4), 0.99 (3H, d, *J* 6.9); δ_C (151 MHz, $CDCl_3$): 161.6, 158.1, 157.6, 151.3, 148.2, 135.4, 135.3, 132.3, 131.5, 124.8, 109.3, 58.8, 42.9, 39.3, 25.4, 11.9, 11.9; HRMS (ESI+): found 478.0355; $C_{17}H_{18}N_5O_5ClSK$, $[M+K]^+$ requires 478.0354; ν_{max} (thin film): 3108.6, 3145.9, 3049.0, 2963.2, 2873.8, 1736.9, 1565.5, 1535.7, 1487.2, 1457.4, 1397.8, 1356.8, 1289.7, 1230.0, 1166.7, 1107.0, 1069.7, 984.0, 902.0, 782.7, 734.3 cm^{-1}; $[\alpha]_D^{20}$ +157.0 (*c* = 1.2, $CHCl_3$).

2.3.2 Valine Series

(*S*)-2-Isopropyl-1-((2-nitrophenyl)sulfonyl)aziridine (S7)

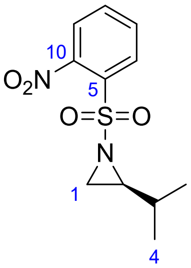

2-Nitrobenzenesulfonyl chloride (16.15 g, 72.9 mmol) was added in five portions to a stirred 0 °C solution of L-valinol (3.00 g, 29.1 mmol) and pyridine (12 mL) in dichloromethane (30 mL). The reaction was stirred vigorously and then allowed to warm to room temperature. After 24 h, the volatiles were removed *in vacuo*. The concentrated reaction mixture was then taken up in diethyl ether (80 mL), and the organic layer was washed with HCl (1 M aq.) until the aqueous washings were acidic (approximately 6 x 40 mL). KOH (2 M aq., 160 mL) was added to the organic layer, and the resulting biphasic mixture was stirred vigorously for 6 h. The layers were separated, and the organic layer washed with KOH (2 M aq., 2 x 40 mL). The organic layer was dried over anhydrous magnesium sulfate, filtered, and concentrated *in vacuo*. The crude residue was passed over a plug of silica (eluent: dichloromethane) and concentrated *in vacuo* to afford **S7** (4.93 g, 63%) as a pale yellow oil.* δ_H (400 MHz, $CDCl_3$): 8.24-8.15 (1H, m, H9), 7.82-7.67 (3H, m,

* It has been previously reported that *N*-nosyl-protected aziridines are prone to polymerisation upon standing.[22] Aziridine **S7**, however, was indefinitely stable when stored as a dilute solution in DCM.

H6, H7 & H8), 2.87-2.79 (2H, m, H1 & H2), 2.37-2.29 (1H, m, H1'), 1.66-1.51 (1H, m, H3), 0.96 (3H, d, *J* 3.8, H4), 0.95 (3H, d, *J* 3.7, H4'); δ_C (75 MHz, $CDCl_3$): 148.6 (C10), 134.6 (C6/C7/C8), 132.0 (C6/C7/C8), 131.5 (C5), 131.1 (C9), 124.2 (C6/C7/C8), 47.1 (C2), 35.0 (C1), 30.0 (C3), 19.4 & 18.8 (C4 & C4'); HRMS (ESI+): found 271.0784; $C_{11}H_{15}N_2O_4S$, $[M+H]^+$ requires 271.0753; v_{max} (thin film): 1542, 1331, 1163, 751, 605, 596 cm^{-1}; $[\alpha]_D^{20}$ +85.6 (*c* = 1.17, $CHCl_3$). These data are in agreement with previously reported values.[6]

(*S*)-*N*-(1-(*tert*-butylamino)-3-methylbutan-2-yl)-2-nitrobenzenesulfonamide (S8)

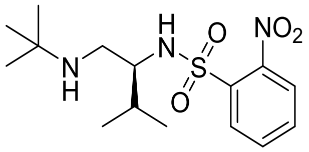

To a stirred, room temperature solution of *N*-nosyl-protected aziridine **S7** (8.01 g, 29.63 mmol) in acetonitrile (74 mL) was added *tert*-butylamine (12.24 mL, 118.52 mmol) in one portion. After 2 h the reaction was complete (TLC: petrol:ethyl acetate, 2:1), and the volatiles were removed *in vacuo*. The crude residue was purified by flash column chromatography (silica gel, petrol:ethyl acetate 2:1) to afforded the product **S8** (9.20 g, 90%) as a colourless solid. δ_H (600 MHz, $CDCl_3$): 8.14-8.12 (1H, m), 7.85-7.84 (1H, m), 7.73-7.68 (2H, m), 3.24-3.21 (1H, m), 2.56 (1H, dd, *J* 11.8, 6), 2.48 (1H, dd, *J* 11.9, 4.0), 1.91-1.85 (1H, m), 0.93 (3H, d, *J* 6.8), 0.90-0.85 (12H, m); δ_C (151 MHz, $CDCl_3$): 148.0, 135.6, 133.2, 132.9, 130.7, 61.4, 50.1, 43.3, 30.7, 29.0, 18.9, 18.7; HRMS (ESI+): found 344.1664; $C_{15}H_{26}N_3O_4S$, $[M+H]^+$ requires 344.1664; v_{max} (thin film): 2959.5, 2870.1, 1539.4, 1364.2, 1218.8, 779.0, 730.6, 685.8 cm^{-1}; $[\alpha]_D^{20}$ –116.5 (*c* = 1.0, $CHCl_3$).

(*S*)-1-(*tert*-butyl)-4-isopropyl-3-((2-nitrophenyl)sulfonyl)imidazolidin-2-one (S9)

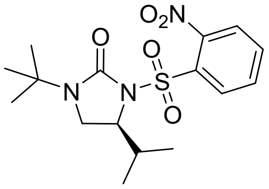

A solution of triphosgene (3.18 g, 10.72 mmol) in acetonitrile (80 mL) was added over 1 h *via* syringe pump to a stirred, room temperature solution of the diamine **S8** (9.20 g, 26.79 mmol) and Hünig's base (10.39 mL, 80.40 mmol) in acetonitrile (160 mL). After a further 30 min, the reaction mixture was concentrated *in vacuo*. The crude residue was taken up in dichloromethane (100 mL), and washed with HCl (40 mL, 1 M aq.). The layers were separated, and the aqueous phase was further extracted with dichloromethane (2 x 60 mL). The combined organic extracts were dried over anhydrous magnesium sulfate, filtered, and concentrated *in vacuo*. The crude residue was purified by flash column chromatography (silica gel, petrol:ethyl acetate 2:1) to afforded di-protected urea **S9** (8.85 g, 89%) as a pale yellow oil that solidified upon standing. δ_H (400 MHz, $CDCl_3$): 8.43-8.41 (1H, m), 7.76-7.69 (3H, m), 4.18-4.14 (1H, m), 3.65 (1H, app. t, *J* 9.1), 3.27 (1H, dd, *J* 9.3, 1.6), 2.34-2.22 (1H, m), 1.27 (9H, s), 1.03 (3H, d, *J* 6.8) 1.00 (3H, d, *J* 6.8); δ_C (101 MHz, $CDCl_3$): 152.9, 147.7, 134.8, 134.1, 132.7, 131.9, 124.0, 58.7, 54.3, 42.4, 32.7, 27.3, 17.9, 15.5; HRMS (ESI+): found 370.1424; $C_{16}H_{24}N_3O_5S$, $[M+H]^+$ requires 370.1437; v_{max} (thin film): 2967.0, 1718.3, 1543.1, 1408.9, 1364.2, 1248.7, 1166.7, 1121.9, 1095.8, 1028.7, 853.6, 752.9, 704.5 cm^{-1}; $[\alpha]_D^{20}$ +321.5 (*c* = 1.43, $CHCl_3$).

(*S*)-1-(*tert*-butyl)-4-isopropylimidazolidin-2-one (S10)

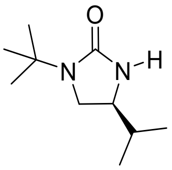

Prepared according to **General Procedure A** using di-protected urea **S9** (3.41 g, 9.23 mmol), thiophenol (1.3 mL,13.85 mmol), K_2CO_3 (3.83 g, 27.69 mmol) and *N*,*N*-DMF (92 mL). Reaction time = 4 h. Purification by flash

column chromatography (silica gel, petrol:ethyl acetate 10:1 → 5:1 → 1:1 (product)) afforded **S10** (1.52 g, 90%) as an off-white crystalline solid. Product **S10** is not visible under UV light and must be stained with ceric ammonium molybdate. δ_H (400 MHz, $CDCl_3$): 4.50 (1H, br s), 3.48 (1H, app. t, *J* 8.7), 3.22 (1H, app q, *J* 15.6, 7.5), 3.08 (1H, app. t, *J* 8.2), 1.62 (1H, m), 1.34 (9H, s), 0.91 (3H, d, *J* 6.8), 0.87 (3H, d, *J* 6.8); δ_C (101 MHz, $CDCl_3$): 162.3, 55.5, 52.9, 47.6, 33.1, 27.7, 18.6, 18.1; HRMS (ESI+): found 185.1636; $C_{10}H_{21}N_2O$, $[M+H]^+$ requires 185.1654; ν_{max} (thin film): 3227.9, 3086.2, 2959.5, 2877.5, 1681.0, 1479.8, 1412.7, 1360.5, 1237.5, 1148.0, 1092.1, 682.1 cm^{-1}; $[\alpha]_D^{20}$ +180.9 (*c* = 1.0, $CHCl_3$).

(*S*)-5-isopropyl-1-((2-nitrophenyl)sulfonyl)imidazolidin-2-one (S11)

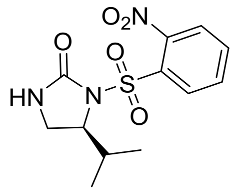

At room temperature, trifluoroacetic acid (52 mL) was added to di-protected urea **S9** (5.0 g, 13.53 mmol). The reaction mixture was stirred and then heated to reflux at 82°C for 16 h. When TLC analysis showed all starting material had been consumed, the reaction mixture was cooled to room temperature, and excess trifluoroacetic acid was removed by running a compressed air line over the solution for 1 h. The resulting crude brown residue was taken up in dichloromethane (60 mL) and washed with $NaHCO_3$ (sat. aq. 30 mL) The layers were separated, and the aqueous layer was further extracted with dichloromethane (2 x 40 mL). The combined organic extracts were dried over anhydrous magnesium sulfate, filtered, and concentrated *in vacuo*. The crude solid was purified by trituration in hot hexane to afford the *tert*-butyl deprotected product **S11** (3.72 g, 88 %) as an off-white crystalline solid. δ_H (400 MHz, $CDCl_3$): 8.43-8.41 (1H, m), 7.75-7.70 (3H, m), 4.95 (1H, br. s), 4.44-4.42 (1H, m), 3.67 (1H, app. t, *J* 9.2), 3.30 (1H, app. d, *J* 9.1), 2.38-2.30 (1H, m), 1.06 (3H, d, *J* 6.8), 1.00 (3H, d, *J* 6.8); δ_C (101 MHz, $CDCl_3$): 155.1, 147.9, 135.0, 134.5, 132.2, 131.8, 124.1, 62.7, 39.5, 32.5, 17.9, 15.3; HRMS (ESI+): found 314.0810; $C_{12}H_{16}N_3O_5S$, $[M+H]^+$ requires 314.0811; ν_{max} (thin film): 3365.8, 3063.9, 2955.8, 2926.0, 2870.1, 1751.8, 1707.1, 1535.7, 1490.9, 1356.8, 1252.4, 1125.7, 1073.5, 957.9, 853.6, 797.7, 723.1 cm^{-1}; $[\alpha]_D^{20}$ +408.5 (*c* = 1.0, $CHCl_3$). These data are in agreement with previously reported values.[6]

(*S*)-1-(6-chloropyrimidin-4-yl)-4-isopropyl-3-((2-nitrophenyl)sulfonyl)imidazolidin-2-one (S12)

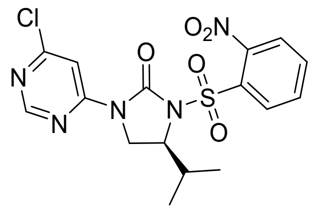

Prepared according to **General Procedure B** using deprotected urea **S11** (0.62 g, 1.99 mmol), 4,6-dichloropyrimidine (1.48 g, 9.93 mmol), $Pd_2(dba)_3$ (0.09 g, 0.10 mmol), Xantphos (0.17 g, 0.30 mmol), toluene (10 mL), and Cs_2CO_3 (1.62 g, 4.98 mmol). Reaction time = 16 h. Purification by flash column chromatography (silica gel, petrol:ethyl acetate, 7:1 → 5:1) afforded the chloropyrimidine **S12** (0.64 g, 75%) as a pale yellow solid. δ_H (600 MHz, $CDCl_3$): 8.67 (1H, s), 8.49-8.48 (1H, m), 8.08 (1H, s), 7.83-7.82 (2H, m), 7.79-7.77 (1H, m), 4.50-4.48 (1H, m), 4.13-4.07 (2H, m), 2.44-2.39 (1H, m), 1.07 (3H, d, *J* 6.8), 1.02 (3H, d, *J* 6.8); δ_C (151 MHz, $CDCl_3$): 161.4, 158.0, 157.5, 151.1, 148.0, 135.3, 135.2, 132.1, 131.3, 124.6, 109.2, 59.6, 43.0, 32.5, 17.8, 14.9; HRMS (ESI+): found 426.0644; $C_{16}H_{17}ClN_5O_5S$, $[M+H]^+$ requires 426.0639; ν_{max} (thin film): 3142.1, 3101.1, 3026.6, 2933.4, 2967.0, 1736.9, 1535.7, 1453.7, 1394.0, 1360.5, 1226.3, 1170.4, 1110.7, 980.3 cm^{-1}; $[\alpha]_D^{20}$ +186.1 (*c* = 1.68, $CHCl_3$). These data are in agreement with previously reported values.[6]

2.3.3 Leucine Series

(*S*)-2-isobutyl-1-((2-nitrophenyl)sulfonyl)aziridine (S13)

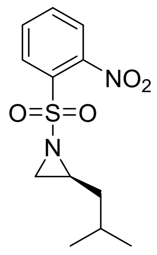

2-Nitrobenzenesulfonyl chloride (14.18 g, 64.00 mmol) was added in five portions to a stirred 0 °C solution of L-leucinol (3.00 g, 25.60 mmol) and pyridine (10.5 mL) in dichloromethane (30 mL). The reaction was stirred vigorously and then allowed to warm to room temperature. After 24 h, the volatiles were removed *in vacuo*. The concentrated reaction mixture was then taken up in diethyl ether (80 mL), and the organic layer was washed with HCl (1 M aq.) until the aqueous washings were acidic (approximately 6 x 40 mL). KOH (2 M aq., 160 mL) was added to the organic layer, and the resulting biphasic mixture was stirred vigorously for 6 h. The layers were separated, and the organic layer washed with KOH (2 M aq., 2 x 40 mL). The organic layer was dried over anhydrous magnesium sulfate, filtered, and concentrated *in vacuo*. Purification by flash column chromatography (silica gel, petrol:ethyl acetate, 10:1 → 5:1) afforded the aziridine **S13** (6.92 g, 95%) as a pale yellow oil.* δ_H (400 MHz, $CDCl_3$): 8.21-8.19 (1H, m), 7.78-7.71 (1H, m), 3.08-3.02 (1H, m), 2.90 (1H, app. d, *J* 7.1), 2.26 (1H, app. d, *J* 4.9), 1.83-1.72 (1H, m), 1.59-1.53 (1H, m), 1.37-1.30 (1H, m), 0.96 (3H, d, *J* 2.0) , 0.94 (3H, d, *J* 2.1); δ_C (101 MHz, $CDCl_3$): 148.7, 134.4, 132.3, 132.2, 131.2, 124.4, 41.0, 40.7, 36.7, 26.8, 22.8, 22.2; HRMS (ESI+): found 285.0901; $C_{12}H_{17}N_2O_4S$, $[M+H]^+$ requires 285.0909; ν_{max} (thin film): 2959.5, 2870.1, 1543.1, 1468.6, 1367.9, 1330.7, 1230.0, 1162.9, 1058.6, 931.8, 849.8, 775.3, 749.2, 678.4 cm^{-1}; $[\alpha]_D^{20}$ +61.0 (*c* = 1.4, $CHCl_3$).

(*S*)-*N*-(1-(*tert*-butylamino)-4-methylpentan-2-yl)-2-nitrobenzenesulfonamide (S14)

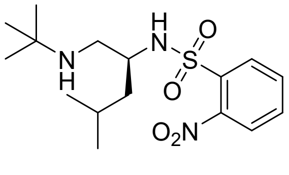

To a stirred, room temperature solution of *N*-nosyl-protected aziridine **S13** (1.0 g, 3.52 mmol) in acetonitrile (9 mL) was added *tert*-butylamine (1.5 mL, 14.08 mmol) in one portion. After 2 h the reaction was complete (TLC: dichloromethane), and the volatiles were removed *in vacuo*. The crude residue was purified by flash column chromatography (silica gel, dichloromethane:ethyl acetate 4:1) to afforded the product **S14** (1.13 g, 90%) as a yellow oil that solidifies upon standing. δ_H (400 MHz, $CDCl_3$): 8.16-8.14 (1H, m), 7.86-7.83 (1H, m), 7.74-7.67 (2H, m), 3.56-3.50 (1H, m), 2.54-2.46 (2H, m), 1.70-1.58 (1H, m), 1.48-1.41 (1H, m), 1.30-1.23 (1H, m), 0.89 (9H, s), 0.87 (3H, d, *J* 6.6), 0.84 (3H, d, *J* 6.6); δ_C (101 MHz, $CDCl_3$): 148.0, 135.7, 133.2, 132.8, 130.7, 125.3, 54.0, 40.1, 46.2, 43.1, 29.0, 24.6, 23.0, 22.3; HRMS (ESI+): found 715.3514; $C_{32}H_{55}N_6O_8S_2$, $[2M+H]^+$ requires 715.3523; ν_{max} (thin film): 3317.3, 2952.1, 2866.3, 1595.3, 1535.7, 1412.7, 1353.0 1233.7, 1170.4, 985.4, 853.6, 782.7, 734.3, 700.7 cm^{-1}; $[\alpha]_D^{20}$ -90.3 (*c* = 1.05, $CHCl_3$).

* It has been previously reported that *N*-nosyl-protected aziridines are prone to polymerisation upon standing.[22] Aziridine **S13**, however, was indefinitely stable when stored as a dilute solution in DCM.

(*S*)-1-(*tert*-butyl)-4-isobutyl-3-((2-nitrophenyl)sulfonyl)imidazolidin-2-one (S15)

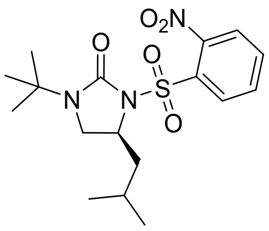

A solution of triphosgene (0.33 g, 1.12 mmol) in acetonitrile (8 mL) was added over 1 h *via* syringe pump to a stirred, room temperature solution of the diamine **S14** (1.00 g, 2.80 mmol) and Hünig's base (1.44 mL, 8.4 mmol) in acetonitrile (16 mL). After a further 30 min, the reaction mixture was concentrated *in vacuo*. The crude residue was taken up in dichloromethane (20 mL), and washed with HCl (10 mL, 1 M aq.). The layers were separated, and the aqueous phase was further extracted with dichloromethane (2 x 30 mL). The combined organic extracts were dried over anhydrous magnesium sulfate, filtered, and concentrated *in vacuo*. The crude residue was purified by flash column chromatography (silica gel, 1% triethylamine:dichloromethane) to afforded di-protected urea **S15** (1.04 g, 97%) as a pale yellow oil. δ_H (400 MHz, $CDCl_3$): 8.42-8.39 (1H, m), 7.76-7.69 (3H, m), 4.32-4.27 (1H, m), 3.72 (1H, app. t, *J* 8.7), 3.17 (1H, app. t, *J* 8.9, 2.2), 1.90-1.83 (1H, m), 1.76-1.65 (2H, m), 1.27 (9H, s), 1.00 (6H, d, *J* 6.3); δ_C (101 MHz, $CDCl_3$): 152.8, 147.9, 134.6, 134.3, 132.8, 132.0, 124.2, 54.3, 53.2, 46.9, 44.9, 27.4, 24.9, 23.5, 22.0; HRMS (ESI+): found 384.1588; $C_{17}H_{26}N_3O_5S$, $[M+H]^+$ requires 384.1593; ν_{max} (thin film): 2959.5, 2870.1, 1741.6, 1539.4, 1468.6, 1408.9, 1349.3, 1274.7, 1166.7, 1110.7, 969.1, 853.6, 782.7, 745.5 cm^{-1}; $[\alpha]_D^{20}$ –331.7 (*c* = 1.1, $CHCl_3$).

(*S*)-1-(*tert*-butyl)-4-isobutylimidazolidin-2-one (S16)

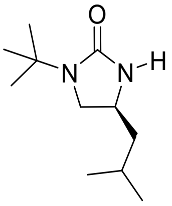

Prepared according to **General Procedure A** using di-protected urea **S15** (4.86 g, 12.66 mmol), thiophenol (1.79 mL, 18.99 mmol), K_2CO_3 (5.25 g, 37.99 mmol) and *N*,*N*-DMF (120 mL). Reaction time = 4 h. Purification by flash column chromatography (silica gel, petrol:ethyl acetate 10:1 → 5:1 → 2:1 (product)) afforded **S16** (2.31 g, 92%) as an off-white crystalline solid. Product **S16** is not visible under UV light and must be stained with ceric ammonium molybdate. δ_H (400 MHz, $CDCl_3$): 4.37 (1H, br. s, N-H), 3.63-3.55 (1H, m), 3.52 (1H, app. t, *J* 8.1), 2.99 (1H, app. t, *J* 7.6), 1.66-1.55 (1H, m), 1.49 (1H, m), 1.34 (9H, s), 1.32-1.28 (1H, m), 0.91 (6 H, app. t, *J* 6.7); δ_C (101 MHz, $CDCl_3$): 162.3, 52.9, 49.8, 47.8, 44.7, 27.7, 25.3, 23.1, 22.4; HRMS (ESI+): found 397.3532; $C_{22}H_{45}N_4O_2$, $[2M+H]^+$ requires 397.3543; ν_{max} (thin film): 3213.0, 3086.2, 2952.1, 2907.3, 2870.1, 1681.0, 1442.6, 1364.2, 1259.8, 1226.3, 1095.8, 1032.5, 931.8, 834.9, 767.8, 693.3 cm^{-1}; $[\alpha]_D^{20}$ -7.2 (*c* = 1.2, $CHCl_3$).

(*S*)-5-isobutyl-1-((2-nitrophenyl)sulfonyl)imidazolidin-2-one (6)

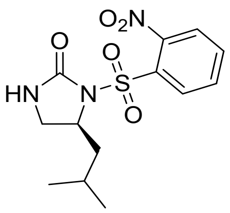

At room temperature, trifluoroacetic acid (10 mL) was added to di-protected urea **S15** (1.0 g, 2.61 mmol). The reaction mixture was stirred and then heated to reflux at 82°C for 16 h. When TLC analysis showed all starting material had been consumed, the reaction mixture was cooled to room temperature, and excess trifluoroacetic acid was removed by running a compressed air line over the solution for 1 h. The resulting crude brown residue was taken up in dichloromethane (60 mL) and washed with $NaHCO_3$ (sat. aq. 30 mL) The layers were separated, and the aqueous layer

was further extracted with dichloromethane (2 x 40 mL). The combined organic extracts were dried over anhydrous magnesium sulfate, filtered, and concentrated *in vacuo*. The crude solid was purified by trituration in hot hexane to afford the *tert*-butyl deprotected product **6** (0.85 g, 99%) as an off-white crystalline solid. δ_H (400 MHz, $CDCl_3$): 8.40-8.38 (1H, m), 7.75-7.69 (3H, m), 5.27 (1H, br. s, N-H), 4.56-4.50 (1H, m), 3.75 (1H, app. t, *J* 8.8), 3.20 (1H, app. dd, *J* 8.8,1.5), 1.92-1.86 (1H, m), 1.81-1.77 (1H, m), 1.73-1.66 (1H, m), 1.00 (6H, d, *J* 6.6); δ_C (101 MHz, $CDCl_3$): 155.1, 148.0, 134.7, 134.6, 132.3, 131.9, 124.3, 57.2, 44.9, 44.1, 24.9, 23.5, 21.; HRMS (ESI+): found 328.0948; $C_{13}H_{17}N_3O_5S$, $[M+H]^+$ requires 328.0967; v_{max} (thin film): 3101.1, 2959.5, 2873.8, 1740.7, 1539.4, 1457.4, 1395.8, 1364.2 1334.4, 1271.0, 1174.4, 1107.0, 984.0, 853.6, 741.7, 767.8, 700.7 cm^{-1}; $[\alpha]_D^{20}$ +477.5 (*c* = 1.2, $CHCl_3$).

(*S*)-1-(6-chloropyrimidin-4-yl)-4-isobutyl-3-((2-nitrophenyl)sulfonyl)imidazolidin-2-one (S17)

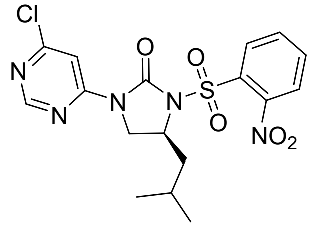

Prepared according to **General Procedure B** using deprotected urea **6** (0.73 g, 2.23 mmol), 4,6-dichloropyrimidine (1.66 g, 11.15 mmol), $Pd_2(dba)_3$ (0.10 g, 0.11 mmol), Xantphos (0.19 g, 0.33 mmol), toluene (11 mL), and Cs_2CO_3 (1.82 g, 5.58 mmol). Reaction time = 16 h. Purification by flash column chromatography (silica gel, petrol:ethyl acetate, 7:1 → 1:1) afforded the chloropyrimidine **S17** (0.84 g, 86%) as a pale yellow solid. δ_H (400 MHz, $CDCl_3$): 8.66 (1H, d, *J* 0.7), 8.50-8.46 (1H, m), 8.10 (1H, d, *J* 0.7), 7.84-7.77 (3H, m), 4.62.4.58 (1H, m), 4.22 (1H, app dd, *J* 10.9, 8.7), 4.01 (1H, app. dd, *J* 11.0, 1.9), 1.99-1.93 (1H, m), 1.83-1.74 (2H, m), 1.04 (6H, app. t, *J* 6.9); δ_C (101 MHz, $CDCl_3$): 161.6, 158.1, 157.9, 150.9, 148.1, 135.4, 135.0, 132.3, 131.5, 124.8, 109.5, 54.1, 47.5, 45.1, 24.9, 23.4, 21.7; HRMS (ESI+): found 440.0781; $C_{17}H_{19}N_5O_5SCl$, $[M+H]^+$ requires 440.0795; v_{max} (thin film): 3365.8, 3.71.3, 2959.5, 2922.2, 2870.1, 1751.8, 1714.6, 159.1, 1550.6, 1464.8, 1420.1 1356.8, 1267.1, 1248.7, 1162.9, 1121.9, 1066.0, 849.8, 797.7, 726.8 cm^{-1}; $[\alpha]_D^{20}$ +166.1 (*c* = 1.1, $CHCl_3$).

2.3.4 Alanine Series

(*S*)-2-methyl-1-((2-nitrophenyl)sulfonyl)aziridine (S18)

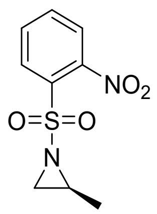

2-Nitrobenzenesulfonyl chloride (21.16 g, 100.0 mmol) was added in five portions to a stirred 0 °C solution of L-alaninol (3.00 g, 40.0 mmol) and pyridine (16 mL) in dichloromethane (40 mL). The reaction was stirred vigorously and then allowed to warm to room temperature. After 24 h, the volatiles were removed *in vacuo*. The concentrated reaction mixture was then taken up in diethyl ether (80 mL), and the organic layer was washed with HCl (1 M aq.) until the aqueous washings were acidic (approximately 6 x 40 mL). KOH (2 M aq., 160 mL) was added to the organic layer, and the resulting biphasic mixture was stirred vigorously for 6 h. The layers were separated, and the organic layer washed with KOH (2 M aq., 2 x 40 mL). The organic layer was dried over anhydrous magnesium sulfate, filtered, and concentrated *in vacuo*. Purification by flash column chromatography (silica gel, dichloromethane) afforded the aziridine **S18** (6.22 g, 64%) as a pale yellow oil.* δ_H (400 MHz, $CDCl_3$): 8.31-8.14 (1H, m), 7.86-7.62 (3H, m), 3.19-3.03 (1H, m), 2.90 (1H, d, *J* 7.0), 2.27 (1H, d, *J* 4.9), 1.37 (3H, d, *J* 5.6); δ_C (101 MHz, $CDCl_3$): 148.5, 134.3, 132.4, 132.2, 131.1,

* It has been previously reported that *N*-nosyl-protected aziridines are prone to polymerisation upon standing.[22] Aziridine **S18**, however, was indefinitely stable when stored as a dilute solution in DCM.

124.3, 38.6, 36.8, 16.9; HRMS (ESI+): found 243.0443; $C_9H_{11}N_2O_4S$, $[M+H]^+$ requires 242.0440; v_{max} (thin film): 3097.4, 2981.9, 1543.1, 1364.2, 1330.7, 1162.9, 1032.5, 849.8, 734.3 cm^{-1}; $[\alpha]_D^{20}$ -0.55 (*c* = 1.1, $CHCl_3$). This compound has previously been reported but only LCMS data were made available.[7]

(*S*)-*N*-(1-(*tert*-butylamino)propan-2-yl)-2-nitrobenzenesulfonamide (S19)

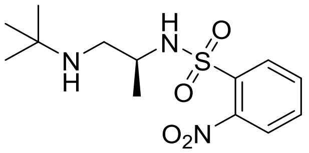

To a stirred, room temperature solution of *N*-nosyl-protected aziridine **S18** (4.15 g, 17.13 mmol) in acetonitrile (43 mL) was added *tert*-butylamine (7.2 mL, 68.52 mmol) in one portion. After 2 h the reaction was complete (TLC: 5% methanol/dichloromethane), and the volatiles were removed *in vacuo*. The crude residue was purified by recrystallisation from petrol and dichloromethane to afford the product **S19** (5.03 g, 93%) as a yellow solid. δ_H (400 MHz, $CDCl_3$): 8.21-8.07 (1H, m), 7.88-7.85 (1H, m), 7.73-7.71(2H, m), 3.57-3.20 (1H, m), 2.60 (1H, dd, *J* 12.1, 4.0), 2.45 (1H, dd, *J* 11.9, 7.4), 1.18 (3H, d, *J* 6.5), 0.97 (9H, s); δ_C (101 MHz, $CDCl_3$): 148.0, 134.7, 133.6, 132.6, 130.9, 125.2, 51.2, 50.1, 47.5, 29.0, 19.7; HRMS (ESI+): found 316.1305; $C_{13}H_{22}N_3O_4S$, $[M+H]^+$ requires 316.1331; v_{max} (thin film): 3321.1, 2967.0, 1539.4, 1364.2, 1170.4, 1125.7, 782.7, 741.7 cm^{-1}; $[\alpha]_D^{20}$ -74.1 (*c* = 1.4, $CHCl_3$).

(*S*)-1-(*tert*-butyl)-4-methyl-3-((2-nitrophenyl)sulfonyl)imidazolidin-2-one (S20)

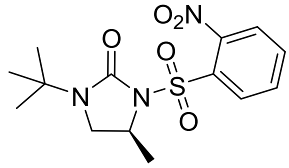

A solution of triphosgene (1.71 g, 14.43 mmol) in acetonitrile (30 mL) was added over 1 h *via* syringe pump to a stirred, room temperature solution of the diamine **S19** (4.55 g, 14.4 mmol) and Hünig's base (7.5 mL, 43.28 mmol) in acetonitrile (42 mL). After a further 30 min, the reaction mixture was concentrated *in vacuo*. The crude residue was taken up in dichloromethane (50 mL), and washed with HCl (20 mL, 1 M aq.). The layers were separated, and the aqueous phase was further extracted with dichloromethane (2 x 50 mL). The combined organic extracts were dried over anhydrous magnesium sulfate, filtered, and concentrated *in vacuo*. The crude residue was purified by flash column chromatography (silica gel, 1% triethylamine:dichloromethane) to afforded di-protected urea **S20** (4.93 g, 96%) as a pale yellow solid. δ_H (400 MHz, $CDCl_3$): 8.44-8.36 (1H, m), 7.78-7.65 (3H, m), 4.47-4.30 (1H, m), 3.74 (1H, t, *J* 8.7), 3.08 (1H, dd, *J* 8.9, 2.7), 1.54 (3H, d, *J* 6.3), 1.30 (9H, s); δ_C (101 MHz, $CDCl_3$): 152.4, 147.8, 134.2, 134.1, 132.5, 131.9, 124.0, 54.2, 50.4, 48.5, 27.3, 22.2; HRMS (ESI+): found 342.1121; $C_{14}H_{20}N_3O_5S$, $[M+H]^+$ requires 342.1124; v_{max} (thin film): 2978.1, 2933.4, 1718.3, 1539.4, 1405.2, 1360.5, 1274.7, 1162.9, 1125.7, 779.0, 738.0 cm^{-1}; $[\alpha]_D^{20}$ +317.9 (*c* = 1.4, $CHCl_3$).

(*S*)-5-methyl-1-((2-nitrophenyl)sulfonyl)imidazolidin-2-one (7)

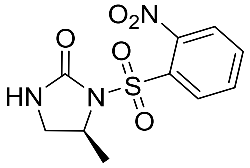

At room temperature, trifluoroacetic acid (29 mL) was added to di-protected urea **S20** (2.53 g, 7.41 mmol). The reaction mixture was stirred and then heated to reflux at 82°C for 16 h. When TLC analysis showed all starting material had been consumed, the reaction mixture was cooled to room temperature, and excess trifluoroacetic acid was removed by running a compressed air line over the solution for 1 h. The resulting crude brown residue was taken up in dichloromethane (60 mL) and washed with $NaHCO_3$ (sat. aq. 30 mL) The layers were separated, and the aqueous layer was further extracted with dichloromethane (2 x 40 mL). The combined organic extracts were dried over anhydrous magnesium sulfate, filtered, and concentrated *in vacuo*. The crude solid was purified by trituration in hot hexane to afford

the *tert*-butyl deprotected product **7** (2.11 g, >99.9%) as gray crystalline solid. δ_H (400 MHz, $CDCl_3$): 8.45-8.35 (1H, m), 7.79-7.65 (3H, m), 5.27 (1H, s), 4.67-4.56 (1H, m), 3.79 (1H, td, *J* 8.8), 3.10 (1H, ddd, *J* 8.8, 2.8, 0.9), 1.60 (3H, d, *J* 6.4); δ_C (101 MHz, $CDCl_3$): 154.7, 147.9, 134.6, 134.3, 132.1, 131.8, 124.1, 54.3, 45.8, 22.4; HRMS (ESI+): found 324.0047; $C_{10}H_{11}N_3O_5SK$, $[M+K]^+$ requires 324.0057; v_{max} (thin film): 3384.4, 1736.9, 1543.1, 1364.2, 1170.4, 1252.4, 1170.4, 1092.1, 853.6 cm^{-1}; $[\alpha]_D^{20}$ +484.5 (*c* = 0.4, $CHCl_3$).

2.4 Synthesis of Acyclic Dimers and Trimers

(*S*)-1-(*tert*-butyl)-3-(6-((*S*)-4-((*S*)-*sec*-butyl)-3-((2-nitrophenyl)sulfonyl)-2-oxoimidazolidin-1-yl)pyrimidin-4-yl)-4-isopropylimidazolidin-2-one (S21)

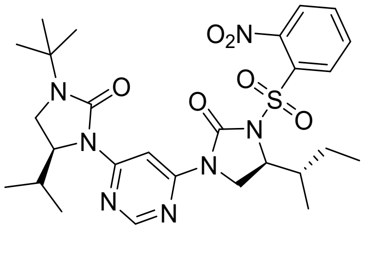

Prepared according to **General Procedure B** using chloropyrimidine **S6** (0.36 g, 0.81 mmol), imidazolidin-2-one **S10** (0.10 g, 0.54 mmol) [added in one portion at reaction time = 0], $Pd_2(dba)_3$ (25 mg, 27 μmol), Xantphos (50 mg, 81 μmol), toluene (5 mL), and Cs_2CO_3 (0.44 g, 1.35 mmol). Reaction time = 16 h. Purification by flash column chromatography (silica gel, firstly 10% ethyl acetate:dichloromethane to remove xantphos and xantphos oxide that co-elutes with the product in petrol:ethyl acetate. The column fractions containing the product were concentrated *in vacuo* and subjected to a second column, silica gel, petrol:ethyl acetate 10:1 → 3:1) afforded the di-protected dimer **S21** (0.16 g, 51%) as a pale yellow solid. δ_H (400 MHz, $CDCl_3$): 8.79 (1H, d, *J* 0.9), 8.56-8.56 (1H, dd, *J* 8.0, 1.1), 8.51 (1H, d, *J* 0.8), 7.79 (1H, app. td, *J* 7.1, 2.1), 7.75-7.71 (2H, m), 4.54 (1H, dt, *J* 8.6, 3.0), 4.47 (1H, dt, *J* 9.3, 3.1), 4.10-4.01 (2H, m), 3.40 (1H, app. t, *J* 9.4), 3.29 (1H, dd, *J* 9.3, 3.0), 2.50-2.42 (1H, m), 2.23-2.13 (1H, m), 1.55-1.44 (1H, m), 1.40 (9H, s), 1.35-1.28 (1H, m), 1.03-0.98 (6H, m), 0.92 (3H, d, *J* 7.1), 0.79 (3H, d, *J* 6.9); δ_C (101 MHz, $CDCl_3$): 158.8, 157.0, 156.8, 155.9, 150.9, 148.2, 135.6, 134.9, 132.4, 131.9, 124.4, 96.6, 58.4, 54.9, 54.0, 42.8, 40.4, 39.3, 28.4, 27.7, 25.4, 18.4, 14.5, 12.0, 11.9; HRMS (ESI+): found 626.2181; $C_{27}H_{37}N_7O_6SK$, $[M+K]^+$ requires 626.2163; v_{max} (thin film): 3093.7, 2963.2, 2922.2, 1736.9, 1572.9, 1539.4, 1435.0, 1390.3, 1360.5, 1274.7, 1215.1, 1162.9, 1110.7, 1069.7, 984.0, 916.9, 887.1, 808.8, 738.0, 678.4 cm^{-1}; $[\alpha]_D^{20}$ +109.0 (*c* = 1.1, $CHCl_3$).

(*S*)-1-(*tert*-butyl)-3-(6-((*S*)-4-((*S*)-*sec*-butyl)-2-oxoimidazolidin-1-yl)pyrimidin-4-yl)-4-isopropylimidazolidin-2-one (S22)

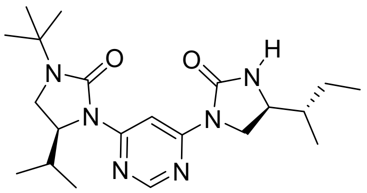

Prepared according to **General Procedure A** using di-protected dimer **S21** (0.48 g, 0.82 mmol), thiophenol (0.12 mL, 1.23 mmol), K_2CO_3 (0.34 g, 2.46 mmol) and *N,N*-DMF (10 mL). Reaction time = 3 h. Purification by flash column chromatography (silica gel, petrol:ethyl acetate 10:1 → 5:1 → 1:1 (product)) afforded **S22** (0.31 g, 94%) as a pale yellow crystalline solid. δ_H (600 MHz, $CDCl_3$): 9.00 (1H, s), 8.50 (1H, s), 5.70 (1H, br. s, N-H), 4.50 (1H, dt, *J* 9.2, 2.8), 4.11 (1H, app. t, *J* 10.0), 3.76 (1H, dd, *J* 10.5, 7.1), 3.61 (1H, app. q, *J* 14.9, 7.1), 3.43 (1H, app. t, *J* 9.1), 3.29 (1H, dd, *J* 8.9, 2.3), 2.50-2.45 (1H, m), 1.55-1.51 (1H, m), 1.41 (10H, app. s), 0.95-0.92 (9H, m), 0.81 (3H, d, *J* 6.8); δ_C (151 MHz, $CDCl_3$): 158.4, 158.2, 157.7, 156.9, 156.0, 96.0, 54.8, 53.9, 53.4, 47.3, 40.5, 39.6, 28.6, 27.7, 25.2, 18.4, 14.6, 13.9, 11.2; HRMS (ESI+): found 403.2825; $C_{21}H_{35}N_6O_2$, $[M+H]^+$ requires 403.2821; v_{max} (thin film): 3186.9, 3123.5, 2963.2, 2929.7, 2877.5, 1714.6, 1580.4, 1461.1, 1397.8, 1367.9, 1267.3, 1244.9, 1162.9, 1114.5, 1073.5, 984.0, 928.1, 883.4, 823.7, 756.6, 693.3 cm^{-1}; $[\alpha]_D^{20}$ +45.5 (*c* = 0.8, $CHCl_3$).

(*S*)-1-(*tert*-butyl)-3-(6-((*S*)-4-((*S*)-*sec*-butyl)-3-(6-chloropyrimidin-4-yl)-2-oxoimidazolidin-1-yl)pyrimidin-4-yl)-4-isopropylimidazolidin-2-one (1a)

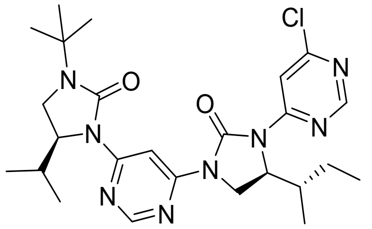

Prepared according to **General Procedure B** using *N*-nosyl deprotected dimer **S22** (0.26 g, 0.63 mmol), 4,6-dichloropyrimidine (0.65 g, 4.44 mmol), $Pd_2(dba)_3$ (29 mg, 32 μmol), Xantphos (54 mg, 95 μmol), toluene (10 mL), and Cs_2CO_3 (0.51 g, 1.50 mmol). Reaction time = 16 h. Then a second portion of $Pd_2(dba)_3$ (29 mg, 32 μmol) and Xantphos (72 mg, 95 μmol) was added. The reaction was heated at reflux for a further 5 h. Purification by flash column chromatography (silica gel, petrol:ethyl acetate, 7:1) afforded the chloropyrimidine **1a** (0.23 g, 71%) as a yellow crystalline solid. δ_H (600 MHz, $CDCl_3$): 9.17 (1H, d, *J* 1.0), 8.66 (1H, d, *J*, 0.9), 8.57 (1H, d, *J* 0.9), 8.56 (1H, d, *J* 1.0), 4.86-4.84 (1H, m), 4.54-4.52 (1H, m), 4.08 (1H, dd, *J* 11.2, 2.4), 3.96 (1H, app. t, *J* 10.2), 3.47 (1H, app. t, *J* 9.3), 3.33 (1H, dd, *J* 9.1, 2.5), 2.54-2.51 (1H, m), 2.32-2.31 (1H, m), 1.54 (10H, app. s), 1.34-1.28 (1H, m), 1.01 (3H, app. t, *J* 7.3), 0.96 (3H, app. t, *J* 7.3), 0.96 (3H, d, *J* 7.0), 0.83 (3H, d, *J* 6.9), 0.76 (3H, d, *J* 6.9); δ_C (151 MHz, $CDCl_3$): 161.5, 158.8, 158.3, 157.9, 157.1, 157.1, 156.0, 153.4, 109.9, 69.5, 54.9, 54.5, 54.1, 41.5, 40.5, 34.9, 28.5, 27.8, 25.6, 18.4, 14.6, 12.0, 11.6; HRMS (ESI+): found 1067.4789; $C_{50}H_{70}N_{16}O_4Cl_2K$, $[2M+K]^+$ requires 1067.4780; v_{max} (thin film): 3138.4, 2952.1, 2870.1, 1714.6, 1561.8, 1528.2, 1490.9, 1435.0, 1382.8, 1274.7, 1241.2, 1200.2, 1110.7, 980.3, 931.8, 745.5, 678.4 cm^{-1}; $[\alpha]_D^{20}$ +24.8 (*c* = 1.3, $CHCl_3$).

(*S*)-4-((*S*)-*sec*-butyl)-3-(6-chloropyrimidin-4-yl)-1-(6-((*S*)-5-isopropyl-2-oxoimidazolidin-1-yl)pyrimidin-4-yl)imidazolidin-2-one (2a)

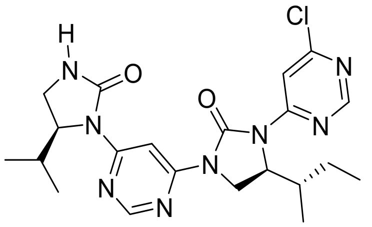

At room temperature, trifluoroacetic acid (5 mL) was added to the chloropyrimidine **1a** (0.18 g, 0.34 mmol). The reaction mixture was stirred and trifluoromethanesulfonic acid (0.6 mL) was subsequently added. When TLC analysis showed all starting material had been consumed, excess trifluoroacetic acid and trifluoromethanesulfonic acid was removed by running a compressed air line over the solution for 1 h. The resulting crude brown residue was taken up in dichloromethane and washed with $NaHCO_3$. The layers were separated, and the aqueous layer was further extracted with dichloromethane (2 x 10 mL). The combined organic extracts were dried over anhydrous magnesium sulfate, filtered, and concentrated *in vacuo*. The crude solid was purified by trituration from hot hexane to afford the *tert*-butyl deprotected dimer **2a** (85 mg, 0.19 mmol, 81%)* as an off-white crystalline solid. δ_H (600 MHz, $CDCl_3$): 9.1 (1H, s), 8.67 (1H, s), 8.59 (1H, s), 8.53 (1H, s), 4.93 (1H, br. s, N-H), 4.86-4.84 (1H, m), 4.77-4.75 (1H, m), 4.08 (1H, dd, *J* 11.1, 2.5), 3.97 (1H, app. t, *J* 10.0), 3.55 (1H, app. t, *J* 9.2), 3.36 (1H, dd, *J* 8.7, 2.0), 2.60 (1H, app. oct, *J* 20.3, 17.1, 13.5, 10.7, 6.8), 2.34-2.33 (1H, m), 1.48-1.44 (1H, m), 1.34-1.28 (1H, m), 1.02 (3H, app. t, *J* 7.24), 0.96 (3H, d, *J* 6.9), 0.88 (3H, d, *J* 6.9), 0.77 (3H, d, *J* 6.7); δ_C (151 MHz, $CDCl_3$): 161.5, 158.6, 158.3, 158.2, 157.9, 157.3, 157.2, 153.5, 109.9, 96.8, 59.0, 54.6, 41.4, 37.7, 34.9, 28.5, 25.6, 18.3, 14.5, 12.0, 11.6; HRMS (ESI+): found 511.1736; $C_{22}H_{29}N_8O_2ClK$, $[M+K]^+$ requires 511.1739; v_{max} (thin film): 3242.8, 3164.5, 2959.5, 2870.1, 1722.0, 1565.5, 1528.2, 1483.5, 1427.6, 1285.9, 1252.4, 1155.5, 1114.5, 984.0, 924.4, 887.1, 745.5, 674.0 cm^{-1}; $[\alpha]_D^{20}$ +12.9 (*c* = 0.4, $CHCl_3$).

---

* Based on recovered starting material. The starting material was soluble in hot hexane.

(*S*)-4-((*S*)-*sec*-butyl)-1-(*tert*-butyl)-3-(6-((*S*)-4-((*S*)-*sec*-butyl)-3-((2-nitrophenyl)sulfonyl)-2-oxoimidazolidin-1-yl)pyrimidin-4-yl)imidazolidin-2-one (S23)

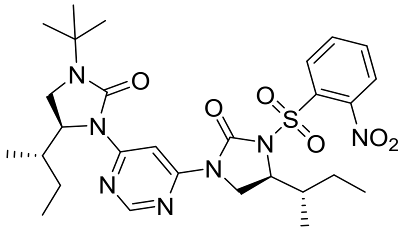

Prepared according to **General Procedure B** using chloropyrimidine **S6** (0.92 g, 2.10 mmol), imidazolidin-2-one **S4** (0.32 g, 1.61 mmol) [added in 2 portions at reaction time = 0 h and at 3 h], $Pd(dba)_2$ (46 mg, 80 μmol), Xantphos (0.14 g, 0.24 mmol), toluene (8 mL), and K_2CO_3 (0.56 g, 4.03 mmol). Reaction time = 16 h. Purification by flash column chromatography (silica gel, firstly 10% ethyl acetate:dichloromethane to remove xantphos and xantphos oxide that co-elutes with the product in petrol:ethyl acetate. The column fractions containing the product were concentrated *in vacuo* and subjected to a second column, silica gel, petrol:ethyl acetate 7:1 → 3:1) afforded the di-protected dimer **S23** (0.73 g, 75%) as a pale yellow solid. δ_H (600 MHz, $CDCl_3$):* 8.79 (1H, s), 8.55 (1H, d, *J* 7.7), 8.51 (1H, s), 7.79 (1H, t, *J* 7.0), 7.76-7.72 (2H, m), 4.55 (2H, dd, *J* 20.0, 9.1), 4.09-4.02 (1H, m), 3.40 (1H, t, *J* 9.6), 3.27 (1H, dd, *J* 9.1, 2.1), 2.21-2.18 (2H, m), 1.56-1.52 (1H, m), 1.39 (9H, s), 1.33-1.27 (2H, m), 1.23-1.18 (1H, m), 1.02-0.95 (6H, m), 0.85-0.81 (3H, m), 0.77 (3H, d, *J* 6.8); δ_C (151 MHz, $CDCl_3$): 158.5, 156.9, 156.6, 155.8, 150.8, 148.0, 135.4, 134.7, 132.2, 131.7, 124.2, 96.5, 58.2, 53.8, 53.7, 42.6, 41.3, 39.1, 34.9, 27.5, 25.6, 25.3, 11.82, 11.80, 11.7, 11.6; HRMS (ESI+): found 602.2754; $C_{28}H_{40}N_7O_6S$, $[M+H]^+$ requires 602.2761; ν_{max} (thin film): 2959.5, 2873.8, 1736.9, 1707.1, 1580.4, 1539.4, 1435.0, 1360.5, 1274.7, 1218.8, 1166.7, 1114.5, 1069.7, 984.0, 875.9, 730.6 cm^{-1}; $[\alpha]_D^{20}$ +76.2 (*c* = 0.7, $CHCl_3$).

(*S*)-4-((*S*)-*sec*-butyl)-1-(*tert*-butyl)-3-(6-((*S*)-4-((*S*)-*sec*-butyl)-2-oxoimidazolidin-1-yl)pyrimidin-4-yl)imidazolidin-2-one (S24)

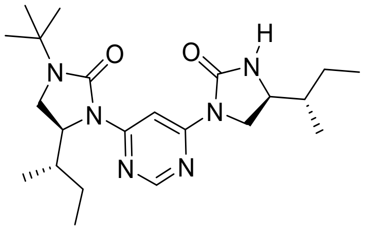

Prepared according to **General Procedure A** using di-protected dimer **S23** (0.41 g, 0.68 mmol), thiophenol (0.1 mL, 1.02 mmol), K_2CO_3 (0.23 g, 1.69 mmol) and *N*,*N*-DMF (7 mL). Reaction time = 8 h. Purification by flash column chromatography (silica gel, petrol:ethyl acetate 10:1 → 2:1(product)) afforded **S24** (0.24 g, 85%) as a pale yellow crystalline solid. δ_H (400 MHz, $CDCl_3$): 8.90 (1H, d, *J* 1.2), 8.50 (1H, d, *J* 1.1), 5.44 (1H, br. s, N-H), 4.59 (1H, dt, *J* 9.3, 3.3), 4.11 (1H, dd, *J* 10.7, 9.2), 3.76 (1H, dd, *J* 10.9, 6.9), 3.61 (1H, app. q, *J* 15.5, 6.6), 3.42 (1H, app. t, *J* 9.3), 3.30 (1H, dd, *J* 9.2, 3.4), 2.35-2.18 (1H, m), 1.57-1.48 (3H, m), 1.40 (10H, s), 1.25-1.23 (1H, m), 0.98-0.89 (9H, m), 0.78 (3H, d, *J* 6.9); δ_C (151 MHz, $CDCl_3$): 158.3, 158.1, 157.7, 156.9, 156.0, 96.2, 53.8, 53.4, 47.3, 40.5, 39.6, 35.3, 27.7, 25.8, 25.2, 17.4, 13.9, 12.0, 11.8, 11.2; HRMS (ESI+): found 455.2520; $C_{22}H_{36}N_6O_2K$, $[M+K]^+$ requires 455.2537; ν_{max} (thin film): 3235.3, 2959.5, 2873.8, 1714.6, 1580.4, 1438.8, 1394.0, 1237.5, 1166.7, 1077.2, 1028.7, 984.0, 883.4, 820.0, 752.9, 670.9 cm^{-1}; $[\alpha]_D^{20}$ +54.3 (*c* = 0.7, $CHCl_3$).

* The product was isolated along with a hydrocarbon grease-type contaminant. This led to measured ^1H integrals in the ~0.5-1.5 ppm region being greater than expected, and some additional ^{13}C peaks in the low ppm range. The reported peaks are those assigned to the product structure on the basis of 2D COSY, HSQC and HMBC experiments.

(*S*)-4-((*S*)-*sec*-butyl)-1-(*tert*-butyl)-3-(6-((*S*)-4-((*S*)-*sec*-butyl)-3-(6-chloropyrimidin-4-yl)-2-oxoimidazolidin-1-yl)pyrimidin-4-yl)imidazolidin-2-one (1b)

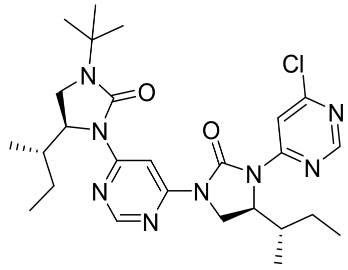

Prepared according to **General Procedure B** using *N*-nosyl deprotected dimer **S24** (0.11 g, 0.25 mmol), 4,6-dichloropyrimidine (0.27 g, 1.78 mmol), $Pd_2(dba)_2$ (12 mg, 13 μmol), Xantphos (22 mg, 38 μmol), toluene (3 mL), and K_2CO_3 (90 mg, 0.64 mmol). Reaction time = 16 h. Purification by flash column chromatography (silica gel, dichloromethane → (impurity) → 10% ethyl acetate:dichloromethane (product) afforded the chloropyrimidine **1b** (0.11 g, 82%) as a yellow crystalline solid. δ_H (600 MHz, $CDCl_3$): 9.16 (1H, s), 8.66 (1H, s), 8.56 (2H, app. d, *J* 3.47), 4.85 (1H, dt, *J* 9.3, 2.8), 4.63 (1H, dt, *J* 9.5, 2.8), 4.07 (1H, dd, *J* 11.0, 2.1), 3.96 (1H, app. t, *J* 10.3), 3.46 (1H, app. t, *J* 9.4), 3.31 (1H, dd, *J* 9.1, 2.3), 2.34-2.26 (2H, m), 1.44 (11H, app. s), 1.33-1.28 (1H, m), 1.23-1.19 (1H, m), 1.00 (6H, app. dt, *J* 14.6, 7.4), 0.81 (3H, d, *J* 6.8), 0.76 (3H, d, *J* 6.8); δ_C (151 MHz, $CDCl_3$): 161.5, 158.7, 158.3, 157.8, 157.1, 157.1, 156.1, 153.4, 109.9, 96.5, 54.5, 53.9, 41.5, 40.5, 35.2, 34.9, 27.8, 25.8, 25.3, 12.1, 12.0, 11.8, 11.5; HRMS (ESI+): found 529.2803; $C_{26}H_{38}N_8O_2Cl$, $[M+H]^+$ requires 529.2806; v_{max} (thin film): 2955.8, 2922.2, 2855.1, 1714.6, 1565.5, 1531.9, 1435.0, 1386.6, 1274.7, 1241.2, 1162.9, 1114.5, 980.3, 928.1, 875.9, 745.5, 678.4 cm^{-1}; $[\alpha]_D^{20}$ +9.1 (*c* = 1.3, $CHCl_3$).

(*S*)-4-((*S*)-*sec*-butyl)-1-(6-((*S*)-5-((*S*)-*sec*-butyl)-2-oxoimidazolidin-1-yl)pyrimidin-4-yl)-3-(6-chloropyrimidin-4-yl)imidazolidin-2-one (2b)

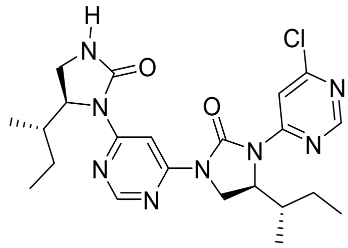

At room temperature, trifluoroacetic acid (mL) was added to the chloropyrimidine **1b** (1.1 g, 0.20 mmol). The reaction mixture was stirred and trifluoromethanesulfonic acid (0.5 mL) was subsequently added. When TLC analysis showed all starting material had been consumed, excess trifluoroacetic acid and trifluoromethanesulfonic acid was removed by running a compressed air line over the solution for 1 h. The resulting crude brown residue was taken up in dichloromethane and washed with $NaHCO_3$. The layers were separated, and the aqueous layer was further extracted with dichloromethane (2 x 10 mL). The combined organic extracts were dried over anhydrous magnesium sulfate, filtered, and concentrated *in vacuo*. The crude solid was purified by trituration from hot hexane to afford the *tert*-butyl deprotected dimer **2b** (76 mg, 80%) as a yellow powder. δ_H (600 MHz, $CDCl_3$): 9.14 (1H, s), 8.67 (1H, s), 8.59 (1H, s), 8.53 (1H, s), 5.03 (1H, br. s, N-H), 4.87-4.85 (2H, m), 4.08 (1H, dd, *J* 11.3, 2.3), 3.97 (1H, app.t, *J* 10.1), 3.54 (1H, app. t, *J* 9.1), 3.35 (1H, dd, *J* 8.7, 2.0), 2.39-2.31 (2H, m), 1.46 (1H, app. oct, 27.8, 20.8, 13.8, 7.5), 1.39 (1H, app. oct, *J* 24.8, 18.1, 11.3, 4.0), 1.32 (1H, app. oct, *J* 26.2, 19.8, 16.3, 4.7), 1.22 (1H, app. oct, *J* 29.8, 23.2, 15.3, 7.7), 1.03-0.98 (6H, m), 0.86 (3H, d, *J* 6.8), 0.77 (3H, d, *J* 6.8); δ_C (151 MHz, $CDCl_3$): 161.5, 158.5, 158.3, 158.3, 157.9, 157.3, 157.2, 153.5, 109.9, 96.9, 58.0, 54.6, 41.4, 37.8, 35.2, 34.9, 25.7, 25.6, 12.0, 12.0, 11.8, 11.6; HRMS (ESI+): found 511.1736; $C_{22}H_{29}N_8O_2ClK$, $[M+K]^+$ requires 511.1739; v_{max} (thin film): 3235.3, 3149.6, 2967.0, 2933.4, 2877.5, 1729.5, 1561.8, 1479.8, 1431.3, 1382.8, 1252.4, 1155.5, 1118.2, 1088.4, 984.0, 879.7, 752.6, 674.6 cm^{-1}; $[\alpha]_D^{20}$ +6.4 (*c* = 1.1, $CHCl_3$).

(*S*)-1-(*tert*-butyl)-4-isobutyl-3-(6-((*S*)-4-isobutyl-3-((2-nitrophenyl)sulfonyl)-2-oxoimidazolidin-1-yl)pyrimidin-4-yl)imidazolidin-2-one (S25)

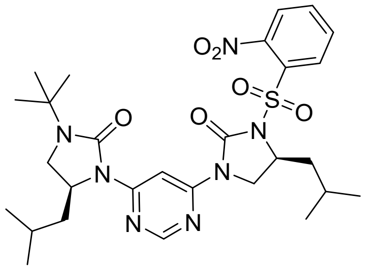

Prepared according to **General Procedure B** using chloropyrimidine **S17** (0.49 g, 1.11 mmol), imidazolidin-2-one **6** (0.20 g, 1.01 mmol), $Pd_2(dba)_3$ (50 mg, 55 µmol), Xantphos (0.09 g, 0.15 mmol), toluene (10 mL), and Cs_2CO_3 (0.81 g, 2.5 mmol). Reaction time = 16 h. Purification by flash column chromatography (silica gel, petrol:ethyl acetate 5:1 → 3:1) afforded the di-protected dimer **S25** (0.55 g, 91%) as a white solid. δ_H (600 MHz, $CDCl_3$): 8.73 (1H, d, *J* 1.0), 8.54 (1H, app. d, *J* 7.9), 8.51 (1H, app. s), 7.78-7.71 (3H, m), 4.55-4.51 (2H, m), 4.20 (1H, app. t, *J* 9.9), 3.96 (1H, app. d, *J* 10.7), 3.51 (1H, app. t, *J* 8.7), 3.18 (1H, app. dd, *J* 8.8, 2.0), 1.92-1.89 (1H, m), 1.79-1.73 (2H, m), 1.71-1.69 (1H, m), 1.40 (10H, s), 1.03-0.98 (9H, m), 0.93 (3H, d, *J* 6.2); δ_C (151 MHz, $CDCl_3$): 158.6, 157.1, 155.7, 150.6, 148.1, 135.4, 134.9, 132.4, 131.9, 124.4, 96.8, 53.8, 53.8, 49.9, 47.5, 45.7, 45.0, 41.6, 29.9, 27.7, 25.1, 24.9, 23.9, 23.6, 21.9, 21.7; HRMS (ESI+): found 602.2771; $C_{28}H_{40}N_7O_6S$, $[M+H]^+$ requires 602.2761; ν_{max} (thin film): 2959.5, 2870.1, 1744.4, 1699.7, 1580.4, 1550.6, 1468.6, 1435.0, 1364.2, 1278.5, 1222.6, 1170.4, 1118.2, 868.5, 782.7, 745.5, 670.9 cm^{-1}; $[\alpha]_D^{20}$ +94.1 (*c* = 1.1, $CHCl_3$).

(*S*)-1-(*tert*-butyl)-4-isobutyl-3-(6-((S)-4-isobutyl-2-oxoimidazolidin-1-yl)pyrimidin-4-yl)imidazolidin-2-one (S26)

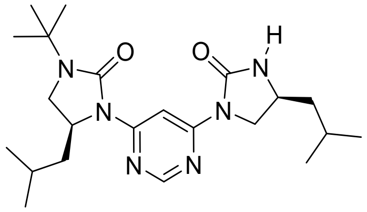

Prepared according to **General Procedure A** using di-protected dimer **S25** (1.52 g, 2.53 mmol), thiophenol (0.36 mL, 3.79 mmol), K_2CO_3 (1.05 g, 7.59 mmol) and *N,N*-DMF (30 mL). Reaction time = 2 h. Purification by flash column chromatography (silica gel, petrol:ethyl acetate 7:1 → 4:1(product)) afforded **S26** (1.03 g, 98 %) as a pale yellow crystalline solid. δ_H (400 MHz, $CDCl_3$): 8.90 (1H, d, *J* 0.8), 8.50 (1H, d, *J* 0.8), 5.37 (1H, br. s, N-H), 4.59-4.54 (1H, m), 4.19 (1H, app. t, *J* 9.6), 3.83 (1H, app. quint, *J* 21.6, 13.8, 6.8), 3.70-3.65 (1H, m), 3.54 (1H, t, *J* 8.6), 3.18 (1H, dd, *J* 8.8, 2.7), 1.77-1.69 (2H, m), 1.67-1.60 (1H, m), 1.58-1.51 (1H, m), 1.47-1.41 (1H, m), 1.41 (10H, app. s), 1.01-0.93 (12H, m); δ_C (101 MHz, $CDCl_3$): 158.2, 158.2, 157.6, 157.0, 155.9, 96.1, 53.7, 50.0, 49.7, 47.4, 45.7, 45.4, 41.8, 27.7, 25.1, 25.1, 23.9, 23.0, 22.5, 21.9; HRMS (ESI+): found 417.2984; $C_{22}H_{37}N_6O_2$, $[M+H]^+$ requires 417.2978; ν_{max} (thin film): 3235.3, 3123.5, 2955.8, 2929.7, 2870.1, 1718.3, 1572.9, 1535.7, 1438.8, 1390.3, 1364.2, 1259.8, 1230.0, 1162.9, 1073.5, 980.3, 752.9, 670.9 cm^{-1}; $[\alpha]_D^{20}$ +56.0 (*c* = 1.1, $CHCl_3$).

(*S*)-1-(*tert*-butyl)-3-(6-((*S*)-3-(6-chloropyrimidin-4-yl)-4-isobutyl-2-oxoimidazolidin-1-yl)pyrimidin-4-yl)-4-isobutylimidazolidin-2-one (1c)

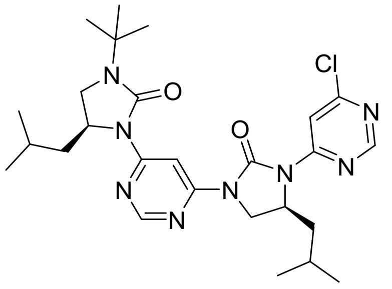

Prepared according to **General Procedure B** using *N*-nosyl deprotected dimer **S26** (0.13 g, 0.31 mmol), 4,6-dichloropyrimidine (0.32 g, 2.15 mmol), $Pd_2(dba)_3$ (14 mg, 15 μmol), Xantphos (27 mg, 0.05 mmol), toluene (3 mL), and Cs_2CO_3 (0.25 g, 0.77 mmol). Reaction time = 16 h. Purification by flash column chromatography (silica gel, petrol:ethyl acetate, 10:1 → 5:1) afforded the chloropyrimidine **1c** (0.14 g, 86%) as a yellow crystalline solid. δ_H (600 MHz, $CDCl_3$): 9.09 (1H, d, *J* 0.9), 8.67 (1H, s), 8.56 (1H, d, *J* 0.8), 8.51 (1H, s), 4.81-4.78 (1H, m) 4.62-4.59 (1H, m), 4.08 (1H, app. t, *J* 10.1), 4.03 (1H, app. dd, *J* 10.7, 1.7), 3.58 (1H, app. t, *J* 8.7), 3.23 (1H, app. dd, *J* 8.6, 1.7), 1.84-1.80 (1H, m), 1.76-1.72 (2H, m), 1.70-1.65 (1H), 1.45 (12H, app. s), 1.05 (3H, d, *J* 6.3), 1.02 (3H, d, *J* 6.3), 0.96 (6H, app. d, *J* 6.4); δ_C (151 MHz, $CDCl_3$): 161.4, 158.5, 158.2, 157.9, 157.4, 157.2, 155.8, 152.9, 109.8, 96.6, 53.9, 50.5, 49.9, 46.4, 45.7, 41.9, 41.6, 27.7, 25.2, 25.0, 24.0, 23.9, 21.9, 21.6; HRMS (ESI+): found 529.2795; $C_{26}H_{38}N_8O_2Cl$, $[M+H]^+$ requires 529.2806; ν_{max} (thin film): 2955.8, 2870.1, 1736.9, 1707.1, 1561.8, 1431.3, 1386.6, 1274.7, 1222.6, 1162.9, 1110.7, 984.0, 864.7, 745.5, 670.9 cm^{-1}; $[\alpha]_D^{20}$ +18.1 (*c* = 0.9, $CHCl_3$).

(*S*)-3-(6-chloropyrimidin-4-yl)-4-isobutyl-1-(6-((*S*)-5-isobutyl-2-oxoimidazolidin-1-yl)pyrimidin-4-yl)imidazolidin-2-one (2c)

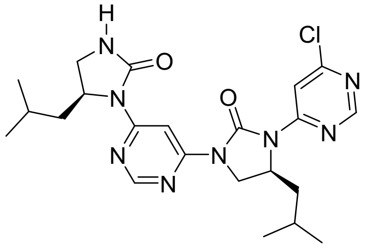

At room temperature, trifluoroacetic acid (27 mL) was added to the chloropyrimidine **1c** (1.43 g, 2.70 mmol). The reaction mixture was stirred and trifluoromethanesulfonic acid (4.3 mL) was subsequently added. When TLC analysis showed all starting material had been consumed, excess trifluoroacetic acid and trifluoromethanesulfonic acid was removed by running a compressed air line over the solution for 1 h. The resulting crude brown residue was taken up in dichloromethane and washed with $NaHCO_3$. The layers were separated, and the aqueous layer was further extracted with dichloromethane (2 x 10 mL). The combined organic extracts were dried over anhydrous magnesium sulfate, filtered, and concentrated *in vacuo*. The crude solid was purified by flash column chromatography (silica gel, petrol:ethyl acetate, 5:1 → 2:1) afforded the *tert*-butyl deprotected dimer **2c** (0.76 g, 60%) as an off-white crystalline solid. δ_H (600 MHz, $CDCl_3$): 9.06 (1H, d, *J* 0.8), 8.67 (1H, d, *J* 0.8), 8.58 (1H, d, *J* 0.8), 8.46 (1H, d, *J* 0.8), 5.47 (1H, br. s, N-H), 4.82-4.78 (2H, m), 4.10 (1H, app. t, *J* 9.9), 4.02 (1H, app. dd, *J* 10.7, 1.8), 3.68 (1H, app. t, *J* 8.7), 3.28 (1H, app. d, *J* 8.4), 1.85-1.73 (3H, m), 1.72-1.69 (1H, m), 1.58-1.54 (1H, m), 1.49-1.45 (1H, m), 1.03 (6H, app. t, *J* 7.1), 0.96 (6H, app. d, *J* 6.2); δ_C (151 MHz, $CDCl_3$): 161.4, 158.4, 158.2, 158.0, 158.0, 157.5, 157.3, 153.0, 109.7, 96.7, 53.8, 50.5, 46.4, 43.0, 41.9, 41.6, 25.0, 25.0, 23.9, 23.9, 21.8, 21.6; HRMS (ESI+): found 473.2195; $C_{22}H_{30}N_8O_2Cl$, $[M+H]^+$ requires 473.2180; ν_{max} (thin film): 3250.2, 3138.4, 2870.1, 2955.8, 2626.0, 2870.1, 1722.0, 1561.8, 1531.9, 1431.3, 1364.2, 1282.2, 1244.9, 1222.6, 1114.5, 984.0, 864.7, 745.5, 667.2 cm^{-1}; $[\alpha]_D^{20}$ +5.9 (*c* = 1.1, $CHCl_3$).

(*S*)-1-(*tert*-butyl)-4-isopropyl-3-(6-((*S*)-4-isopropyl-3-((2-nitrophenyl)sulfonyl)-2-oxoimidazolidin-1-yl)pyrimidin-4-yl)imidazolidin-2-one (S27)

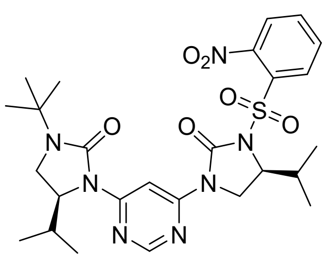

Prepared according to **General Procedure B** using chloropyrimidine **S12** (0.86 g, 2.02 mmol), imidazolidin-2-one **S10** (0.27 g, 1.47 mmol) [added in 2 portions at reaction time = 0 h and at 3 h], $Pd_2(dba)_3$ (70 mg, 80 μmol), Xantphos (0.13 g, 0.23 mmol), toluene (8 mL), and K_2CO_3 (0.54 g, 3.88 mmol). Reaction time = 16 h. Purification by flash column chromatography (silica gel, firstly 10% ethyl acetate:dichloromethane to remove xantphos and xantphos oxide that co-elutes with the product in petrol:ethyl acetate. The column fractions containing the product were concentrated *in vacuo* and subjected to a second column, silica gel, petrol:ethyl acetate 7:1 → 1:1) afforded the di-protected dimer **S27** (0.70 g, 83%) as a pale yellow solid. δ_H (600 MHz, $CDCl_3$): 8.79 (1H, s), 8.55-8.34 (1H, m), 8.51 (1H, s), 7.80-7.71 (3H, m), 4.47-4.46 (1H, m), 4.43-4.41 (1H, m), 4.12-4.03 (2H, m), 3.40 (1H, app. t, *J* 9.4), 3.29 (1H, dd, *J* 9.1, 2.4), 2.47-2.45 (1H, m), 2.41-2.38 (1H, m), 1.39 (9H, s), 1.02 (3H, d, *J* 6.8), 0.95 (3H, d, *J* 6.8), 0.83 (3H, d, *J* 6.8), 0.78 (3H, d, *J* 6.8); δ_C (151 MHz, $CDCl_3$): 158.8, 157.1, 156.8, 155.9, 150.9, 148.2, 135.7, 134.9, 132.4, 131.9, 124.4, 96.6, 59.4, 54.9, 54.0, 43.1, 40.4, 32.6, 28.5, 27.7, 18.4, 18.0, 15.0, 14.5; HRMS (ESI+): found 574.2438; $C_{26}H_{36}N_7O_6S$, $[M+H]^+$ requires 574.2448; ν_{max} (thin film): 2959.5, 1736.9, 1707.1, 1580.4, 1546.8, 1371.7, 1207.7, 1174.1, 1066.0, 872.2, 775.3, 715.6 cm^{-1}; $[\alpha]_D^{20}$ +88.7 (*c* = 1.0, $CHCl_3$).

(*S*)-1-(*tert*-butyl)-4-isopropyl-3-(6-((*S*)-4-isopropyl-2-oxoimidazolidin-1-yl)pyrimidin-4-yl)imidazolidin-2-one (S28)

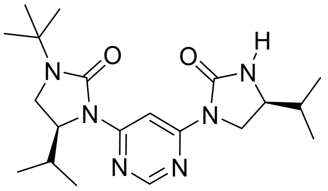

Prepared according to General Procedure A using di-protected dimer **S27** (0.69 g, 1.21 mmol), thiophenol (0.17 mL,1.82 mmol), K_2CO_3 (0.50 g, 3.63 mmol) and *N*,*N*-DMF (12 mL). Reaction time = 2 h. Purification by flash column chromatography (silica gel, petrol:ethyl acetate 7:1 → 3:1 → 1:1 (product)) afforded **S28** (0.32 g, 69 %) as a yellow crystalline solid. δ_H (400 MHz, $CDCl_3$): 9.01 (1H, s), 8.50 (1H, s), 5.71 (1H, br. s), 4.51-4.47 (1H, s), 4.13 (1H, app. t, *J* 10.2), 3.76 (1H, app. dd, *J* 10.65, 6.7), 3.51 (1H, app. q, *J* 15.5, 7.3), 3.43 (1H, app. t, *J* 9.2), 3.29 (1H, app. dd, *J* 9.1, 2.7), 2.52-2.44 (1H, m), 1.78-1.70 (1H, m), 1.41 (9H, s), 0.99 (3H, d, *J* 6.8), 0.94 (6H, app. dd, *J* 10.2, 6.8), 0.80 (3H, d, *J* 6.8); δ_C (101 MHz, $CDCl_3$): 158.5, 158.3, 157.9, 157.0, 156.0, 96.2, 54.9, 53.9, 47.8, 40.6, 33.4, 28.7, 27.8, 18.5, 18.3,17.9, 14.7; HRMS (ESI+): found 389.2660; $C_{20}H_{33}N_6O_2$, $[M+H]^+$ requires 389.2665; ν_{max} (thin film): 3213.0, 3112.3, 2959.5, 2873.8, 1707.1, 1569.2, 1461.1, 1438.8, 1386.6, 1244.9, 1162.9, 980.3, 883.4, 756.6, 670.9 cm^{-1}; $[\alpha]_D^{20}$ +54.7 (*c* = 1.0, $CHCl_3$).

(*S*)-1-(*tert*-butyl)-3-(6-((*S*)-3-(6-chloropyrimidin-4-yl)-4-isopropyl-2-oxoimidazolidin-1-yl)pyrimidin-4-yl)-4-isopropylimidazolidin-2-one (1d)

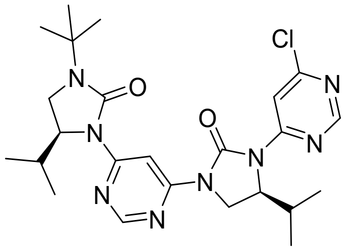

Prepared according to **General Procedure B** using *N*-nosyl deprotected dimer **S28** (0.32 g, 0.82 mmol), 4,6-dichloropyrimidine (0.87 g, 5.82 mmol), $Pd_2(dba)_3$ (38 mg, 42 μmol), Xantphos (72 mg, 0.12 mmol), toluene (5 mL), and K_2CO_3 (0.29 g, 2.08 mmol). Reaction time = 16 h. Then a second portion of $Pd_2(dba)_3$ (38 mg, 42 μmol) and Xantphos (72 mg, 0.12 mmol) was added. The reaction was heated at reflux for a further 5 h. Purification by flash column chromatography (silica gel, petrol:ethyl acetate, 7:1 → 2:1) afforded the chloropyrimidine **1d** (0.19 g, 46%) as an off-white foam. δ_H (400 MHz, $CDCl_3$): 9.17 (1H, s), 8.65 (1H, s), 8.56 (1H, s), 4.76-4.73 (1H, m), 4.54-4.52 (1H, m), 4.10 (1H, dd, *J* 11.2, 2.4), 3.95 (1H, app. t, *J* 10.3), 3.47 (1H, app. t, *J* 9.3), 3.33 (1H, dd, *J* 9.3, 2.4), 2.60-2.49 (2H, m), 1.45 (9H, s), 1.02 (3H, d, *J* 7.0), 0.96 (3H, d, *J* 7.0), 0.83 (3H, d, *J* 7.0), 0.78 (3H, d, *J* 7.0); δ_C (101 MHz, $CDCl_3$):161.5, 158.8, 158.4, 157.8, 157.1, 157.1, 156.0, 153.3, 109.9, 96.5, 55.6, 54.9, 54.0, 41.4, 40.5, 28.5, 28.3, 27.8, 18.4, 18.2, 14.6, 14.1; HRMS (ESI+): found 501.2498; $C_{24}H_{34}N_8O_2SCl$, $[M+H]^+$ requires 501.2493; ν_{max} (thin film): 3145.9, 2959.5, 2873.8, 1710.8, 1561.8, 1531.9, 1431.3, 1386.6, 1237.5, 1207.7, 1114.5, 984.0, 864.7, 678.4 cm^{-1}; $[\alpha]_D^{20}$ +19 (*c* = 1.0, $CHCl_3$).

(*S*)-3-(6-chloropyrimidin-4-yl)-4-isopropyl-1-(6-((*S*)-5-isopropyl-2-oxoimidazolidin-1-yl)pyrimidin-4-yl)imidazolidin-2-one (2d)

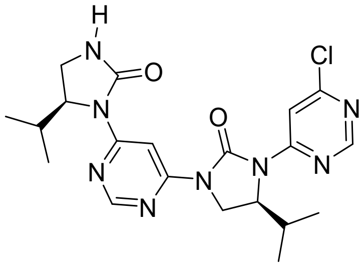

At room temperature, trifluoroacetic acid (21 mL) was added to the chloropyrimidine **1d** (0.76 g, 1.51 mmol). The reaction mixture was stirred and trifluoromethanesulfonic acid (2 mL) was subsequently added. When TLC analysis showed all starting material had been consumed, excess trifluoroacetic acid and trifluoromethanesulfonic acid was removed by running a compressed air line over the solution for 1 h. The resulting crude brown residue was taken up in dichloromethane and washed with $NaHCO_3$. The layers were separated, and the aqueous layer was further extracted with dichloromethane (2 x 10 mL). The combined organic extracts were dried over anhydrous magnesium sulfate, filtered, and concentrated *in vacuo*. The crude solid was purified by trituration in hot hexane to afford the *tert*-butyl deprotected dimer **2d** (0.54 g, 80 %) as an off-white crystalline solid. δ_H (400 MHz, $CDCl_3$): 9.13 (1H, s), 8.66 (1H, s), 8.60 (1H, s), 8.52 (1H, s), 5.17 (1H, br. s), 4.77-4.74 (2H, m), 4.11 (1H, dd, *J* 11.4, 2.4), 3.97 (1H, app. t, *J* 10.1), 3.56 (1H, app. t, *J* 9.3), 3.37 (1H, dd, *J* 9.0, 2.2), 2.63-2.54 (2H, m), 1.06 (3H, d, *J* 6.9), 0.96 (3H, d, *J* 6.9), 0.88 (3H, d, *J* 6.8), 0.79 (3H, d, *J* 6.8); δ_C (101 MHz, $CDCl_3$): 161.4, 158.4, 158.2, 158.1, 157.7, 157.2, 157.0, 153.2, 109.7, 96.6, 58.9, 55.5, 41.2, 37.6, 28.4, 28.2, 18.1, 18.0, 14.4, 14.0; HRMS (ESI+): found 445.1857; $C_{20}H_{26}N_8O_2Cl$, $[M+H]^+$ requires 445.1867; ν_{max} (thin film): 3231.6, 3142.1, 2959.5, 1729.5, 1561.8, 1427.6, 1382.8, 1252.4, 1114.5, 984.0, 872.2, 764.1, 674.6 cm^{-1}; $[\alpha]_D^{20}$ +9.13 (*c* = 1.0, $CHCl_3$).

(*S*)-1-(*tert*-butyl)-4-isobutyl-3-(6-((*S*)-4-isobutyl-3-(6-((*S*)-4-isobutyl-3-((2-nitrophenyl)sulfonyl)-2-oxoimidazolidin-1-yl)pyrimidin-4-yl)-2-oxoimidazolidin-1-yl)pyrimidin-4-yl)imidazolidin-2-one (S29)

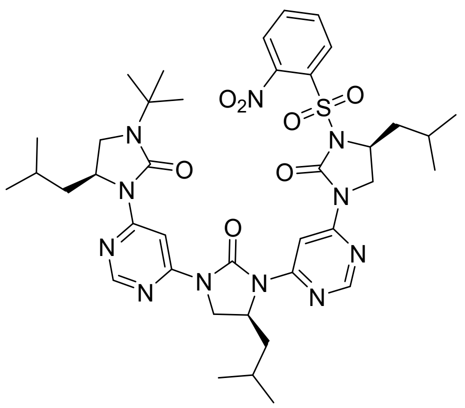

Prepared according to **General Procedure B** using chloropyrimidine **1c** (0.12 g, 0.22 mmol), imidazolidin-2-one **6** (93 mg, 0.28 mmol) [added in one portion at reaction time = 0], $Pd_2(dba)_3$ (10 mg, 11 µmol), Xantphos (19 mg, 33 µmol), toluene (2 mL), and K_2CO_3 (80 mg, 0.55 mmol). Reaction time = 16 h. Purification by flash column chromatography (silica gel, petrol:ethyl acetate 5:1 → 2:1) afforded the di-protected trimer **S29** (98 mg, 73%) as a pale yellow solid. δ_H (400 MHz, $CDCl_3$):* 8.95 (1H, d, *J* 1.2), 8.81 (1H, d, *J* 1.2), 8.58 (1H, d, *J* 0.9), 8.54 (1H, d, *J* 0.9), 8.53-8.50 (1H, m), 7.82-7.71 (3H, m), 4.78-4.71 (1H, m), 4.63-4.52 (2H, m), 4.22 (1H, dd, *J* 10.6, 8.5), 4.03 (1H, dd, *J* 10.6, 8.4), 3.99-3.91 (2H, m), 3.57 (1H, t, *J* 8.6), 3.23 (1H, dd, *J* 8.8, 2.5), 1.97-1.91 (1H, m), 1.85-1.64 (6H, m), 1.47 (9H, s), 1.45-1.39 (2H, m), 1.06-1.00 (12H, m), 0.96 (3H, d, *J* 6.4), 0.93 (3H, d, *J* 6.4); δ_C (151 MHz, $CDCl_3$): 158.3, 157.8, 157.6, 157.5, 157.0, 156.9, 155.6, 152.7, 150.4, 147.9, 135.5, 134.8, 132.3, 131.5, 124.3, 98.0, 97.0, 53.72, 53.68, 50.4, 49.8, 47.5, 46.3, 45.6, 45.0, 41.8, 41.5, 27.6, 25.0, 24.9, 24.8, 23.83, 23.76, 23.4, 21.8, 21.7, 21.6; HRMS (ESI+): found 858.3474; $C_{39}H_{53}N_{11}O_7K$, $[M+K]^+$ requires 858.3487; v_{max} (thin film): 2955.8, 2922.2, 2855.1, 1714.6, 1576.7, 1543.1, 1427.6, 1364.2, 1215.1, 1170.4, 1110.7, 875.9, 745.5, 670.9 cm^{-1}; $[\alpha]_D^{20}$ +1.8 (*c* = 1.1, $CHCl_3$).

(*S*)-1-(*tert*-butyl)-4-isobutyl-3-(6-((*S*)-4-isobutyl-3-(6-((*S*)-4-isobutyl-2-oxoimidazolidin-1-yl)pyrimidin-4-yl)-2-oxoimidazolidin-1-yl)pyrimidin-4-yl)imidazolidin-2-one (S30)

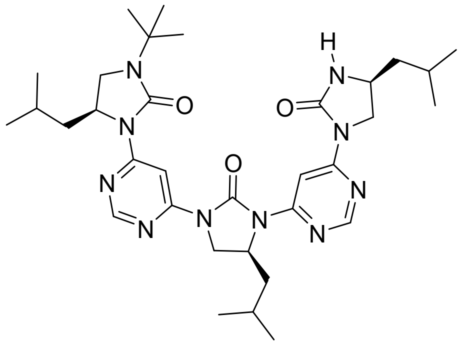

Prepared according to **General Procedure A** using di-protected trimer **S29** (0.50 g, 0.61 mmol), thiophenol (90 µL, 0.91 mmol), K_2CO_3 (0.25 g, 1.83 mmol) and *N*,*N*-DMF (6 mL). Reaction time = 5 h. Purification by flash column chromatography (silica gel, petrol:ethyl acetate 7:1 → 5:1 → 3:1→ 1:1 (product)) afforded **S30** (0.35 g, 90 %) as a pale yellow crystalline solid. δ_H (400 MHz, $CDCl_3$): 9.00 (1H, d, *J* 1.2), 8.94 (1H, d, *J* 1.1), 8.58 (1H, d, *J* 1.2), 8.54 (1H, d, *J* 1.2), 5.01 (1H, br s), 4.85-4.77 (1H, m), 4.60-4.53 (1H, m), 4.21 (1H, dd, *J* 10.5, 8.5), 4.08 (1H, dd, *J* 10.5, 8.7), 3.94 (1H, dd, *J* 10.7, 3.1), 3.86 (1H, quintet, *J* 7.2), 3.70 (1H, dd, *J* 10.5, 6.7), 3.55 (1H, t, *J* 8.6), 3.19 (1H, dd, *J* 8.8, 2.6), 1.86-1.62 (5H, m), 1.59-1.43 (5H, m), 1.41 (9H, s), 1.02 (6H, app. t, *J* 6.1), 0.98-0.92 (12H, m); δ_C (101 MHz, $CDCl_3$):

* A trace impurity was observed in the aromatic region with an integration of 0.08 relative to the title compound, e.g. δ = 9.08 (0.08H, d, *J* 1.2).

158.5, 158.3, 157.7, 157.3, 157.2, 156.9, 156.8, 152.9, 97.3, 96.9, 53.7, 50.1, 49.9, 49.8, 47.3, 46.3, 45.6, 45.2, 42.1, 41.5, 27.6, 25.0,* 24.8, 23.81, 23.77, 22.8, 22.4, 21.8, 21.6; HRMS (ESI+): found 673.3727; $C_{33}H_{50}N_{10}O_3K$, $[M+K]^+$ requires 673.3704; v_{max} (thin film): 2922.2, 2955.8 1718.3, 1580.4, 1423.8, 1356.8, 1215.1, 984.0, 752.9, 670.9 cm^{-1}; $[\alpha]_D^{20}$ +40.4 (*c* = 1.4, $CHCl_3$).

(*S*)-1-(*tert*-butyl)-3-(6-((*S*)-3-(6-((*S*)-3-(6-chloropyrimidin-4-yl)-4-isobutyl-2-oxoimidazolidin-1-yl)pyrimidin-4-yl)-4-isobutyl-2-oxoimidazolidin-1-yl)pyrimidin-4-yl)-4-isobutylimidazolidin-2-one (S31)

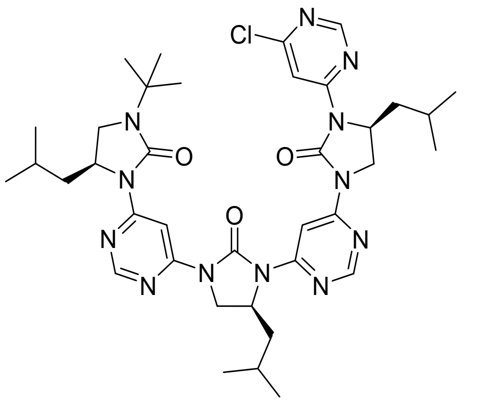

Prepared according to **General Procedure B** using *N*-nosyl deprotected trimer **S30** (72 mg, 0.11 mmol), 4,6-dichloropyrimidine (0.15 g, 1.02 mmol), $Pd_2(dba)_3$ (5 mg, 6 μmol), Xantphos (12 mg, 20 μmol), toluene (2 mL), and K_2CO_3 (39 mg, 0.28 mmol). Reaction time = 16 h. Purification by flash column chromatography (silica gel, petrol:ethyl acetate, 7:1 → 3:1) afforded the chloropyrimidine **S31** (51 mg, 62%) as a yellow crystalline solid. δ_H (400 MHz, $CDCl_3$): 9.12 (1H, d, *J* 1.0), 8.99 (1H, d, *J* 1.0), 8.67 (1H, d, *J* 0.8), 8.63 (1H, d, *J* 1.0), 8.55 (1H, d, *J* 1.0), 8.49 (1H, d, *J* 0.8), 4.85-4.76 (2H, m), 4.60-4.52 (1H, m), 4.16-4.07 (2H, m), 4.03 (1H, dd, *J* 10.7, 2.4), 3.98 (1H, dd, *J* 10.7, 2.5), 3.56 (1H, t, *J* 8.5), 3.21 (1H, dd, *J*, 8.7, 2.3), 1.88-1.63 (6H, m), 1.56-1.46 (3H, m), 1.43 (9H, s), 1.08-1.00 (9H, m), 0.99-0.92 (9H, m); δ_C (101 MHz, $CDCl_3$): 161.3, 158.4, 158.0, 157.82, 157.79, 157.7, 157.6, 157.1, 156.9, 155.6, 152.9, 152.7, 109.7, 97.9, 97.2, 53.7, 50.42, 50.37, 50.0, 46.38, 46.36, 45.6, 41.9, 41.7, 41.4, 27.6, 25.0, 24.9,† 23.83, 23.79, 23.7, 21.8, 21.6, 21.5; HRMS (ESI+): found 785.3542; $C_{37}H_{51}N_{12}O_3ClK$, $[M+K]^+$ requires 785.3533; v_{max} (thin film): 2955.8, 2870.1, 1722.0, 1565.5, 1438.8, 1360.5, 1218.8, 984.0, 875.9, 745.5, 670.9 cm^{-1}; $[\alpha]_D^{20}$ -5.5 (*c* = 3.1, $CHCl_3$).

* HMBC experiment indicated this peak corresponds to two carbon atoms.

† An HSQC experiment indicates that this peak corresponds to two separate ^{13}C positions.

(*S*)-3-(6-chloropyrimidin-4-yl)-4-isobutyl-1-(6-((*S*)-5-isobutyl-3-(6-((*S*)-5-isobutyl-2-oxoimidazolidin-1-yl)pyrimidin-4-yl)-2-oxoimidazolidin-1-yl)pyrimidin-4-yl)imidazolidin-2-one (4a)

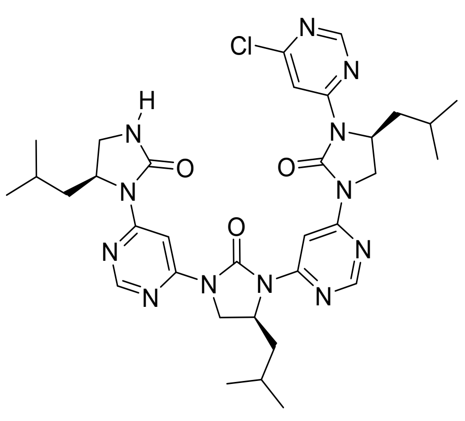

At room temperature, trifluoroacetic acid (2 mL) was added to the chloropyrimidine **S31** (47 mg, 63 μmol). The reaction mixture was stirred and trifluoromethanesulfonic acid (0.5 mL) was subsequently added. When TLC analysis showed all starting material had been consumed, excess trifluoroacetic acid and trifluoromethanesulfonic acid was removed by running a compressed air line over the solution for 1 h. The resulting crude brown residue was taken up in dichloromethane and washed with $NaHCO_3$. The layers were separated, and the aqueous layer was further extracted with dichloromethane (2 x 10 mL). The combined organic extracts were dried over anhydrous magnesium sulfate, filtered, and concentrated *in vacuo*. The crude solid was used without any further purification. The *tert*-butyl deprotected trimer **4a** (0.44 g, >99%) was obtained as a pale yellow crystalline solid. δ_H (600 MHz, $CDCl_3$): 9.11 (1H, s), 8.99 (1H, s), 8.66 (1H, s), 8.62 (1H, s), 8.56 (1H, s), 8.44 (1H, s), 5.42 (1H, br. s, N-H), 4.82-4.77 (3H, m), 4.11 (1H, app. t, *J* 9.3), 4.03 (1H, d, *J* 10.2), 3.99 (1H, d, *J* 10.2), 3.70 (1H, app. t, *J* 8.1), 3.27 (1H, d, *J* 8.1), 1.85-1.80 (3H, m), 1.80-1.77 (3H, m), 1.56-1.52 (1H, m), 1.48-1.46 (2H, m), 1.04 (6H, app. br. s), 1.00 (3H, d, *J* 6.9), 0.95 (9H, dd, *J* 14.0, 6.0); δ_C (151 MHz, $CDCl_3$): 161.5, 158.3, 158.1, 158.0, 157.9, 157.9, 157.8, 157.8, 157.2, 157.1, 153.0, 152.8, 109.7, 109.7, 97.7, 97.0, 53.9, 50.5, 46.5, 46.5, 43.1, 42.1, 41.9, 41.5, 25.1, 25.0, 25.0, 23.9, 23.9, 22.7, 21.8, 21.7, 21.6, 19.6; HRMS (ESI+): found 729.2920; $C_{33}H_{43}N_{12}O_3ClK$, $[M+K]^+$ requires 729.2907; ν_{max} (thin film): 3138.4, 2952.1, 2866.3, 2363.1, 1722.0, 1561.8, 1531.9, 1420.1, 1356.8, 1203.9, 1110.7, 1058.6, 984.0, 864.7, 745.5, 663.5 cm^{-1}; $[\alpha]_D^{20}$ -42.4 (*c* = 1.8, $CHCl_3$).

(*S*)-4-((*S*)-*sec*-butyl)-1-(*tert*-butyl)-3-(6-((*S*)-4-isopropyl-3-((2-nitrophenyl)sulfonyl)-2-oxoimidazolidin-1-yl)pyrimidin-4-yl)imidazolidin-2-one (S32)

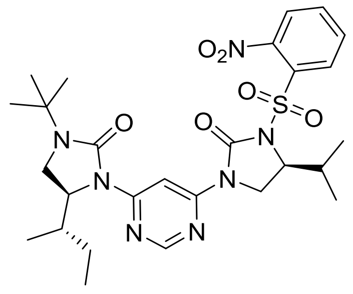

Prepared according to **General Procedure B** using chloropyrimidine **S12** (0.50 g, 1.17 mmol), *N*-nosyl deprotected imidazolidin-2-one **S4** (0.19 g, 0.96 mmol) [added in 2 portions at reaction time = 0 h and at 3 h], $Pd(dba)_2$ (44 mg, 49 μmol), Xantphos (84 mg, 0.15 mmol), toluene (9.7 mL), and Cs_2CO_3 (0.79 g, 2.43 mmol). Reaction time = 16 h. Then a second portion of $Pd_2(dba)_3$ (44 mg, 49 μmol) and Xantphos (84 mg, 0.15 mmol) was added. The reaction was heated at reflux for a further 5 h. Purification by flash column chromatography (silica gel, firstly 10% ethyl acetate:dichloromethane to remove xantphos and xantphos oxide that co-elutes with the product in petrol:ethyl acetate. The column fractions containing the product were concentrated *in vacuo* and subjected to a second column, silica gel, petrol:ethyl acetate 7:1 → 3:1) afforded the di-protected dimer **S32** (0.37 g, 65%) as a pale yellow solid. δ_H (600 MHz, $CDCl_3$): 8.78 (1H, s), 8.55 (1H, d, *J* 8.1), 8.52 (1H, s), 7.81-7.78 (1H, m), 7.76-7.72 (2H, m), 4.58-4.56 (1H, m), 4.42 (1H, m), 4.09 (1H, app. t, *J* 10.5), 4.05 (1H, app. dd, *J* 10.7, 1.7), 3.40 (1H, app. t, *J* 8.9), 3.27 (1H, dd, *J* 9.2, 2.7), 2.39 (1H, app. oct, *J* 20.3, 17.1, 13.6, 10.8, 7.0), 2.24-2.19 (1H, m), 1.39 (10H, app. s), 1.21-1.16 (1H, m), 1.05 (3H, d, *J* 7.0), 1.01

(3H, d, *J* 6.8), 0.96 (3H, t, *J* 7.1), 0.77 (3H, d, *J* 6.8); δ_C (151 MHz, $CDCl_3$): 158.6, 157.0, 156.7, 155.9, 150.8, 148.0, 135.5, 134.8, 132.3, 131.8, 124.3, 96.6, 59.3, 53.8, 53.7, 42.9, 40.3, 35.0, 32.4, 27.6, 25.7, 17.9, 14.9, 11.9, 11.6; HRMS (ESI+): found 626.2164; $C_{27}H_{37}N_7O_6SK$, $[M+K]^+$ requires 626.2163; v_{max} (thin film): 2959.5, 2877.5, 1710.8, 1580.4, 1543.1, 1464.8, 1435.0, 1360.5, 1274.7, 1215.1, 1170.4, 1114.5, 1073.0, 984.0, 875.9, 760.4, 730.6 cm^{-1}; $[\alpha]_D^{20}$ +110.0 (*c* = 0.7, $CHCl_3$).

(*S*)-4-((*S*)-*sec*-butyl)-1-(*tert*-butyl)-3-(6-((*S*)-4-isopropyl-2-oxoimidazolidin-1-yl)pyrimidin-4-yl)imidazolidin-2-one (S33)

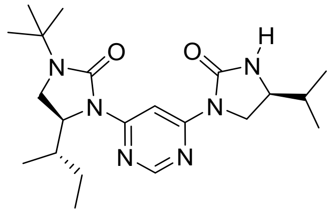

Prepared according to **General Procedure A** using di-protected dimer **S32** (0.58 g, 0.99 mmol), thiophenol (140 µL, 1.48 mmol), K_2CO_3 (0.41 g, 2.97 mmol) and *N*,*N*-DMF (10 mL). Reaction time = 4 h. Purification by flash column chromatography (silica gel, petrol:ethyl acetate 7:1 → 4:1 → 2:1 (product)) afforded **S33** (0.32 g, 80%) as a pale yellow crystalline solid. δ_H (600 MHz, $CDCl_3$): 8.98 (1H, s), 8.50 (1H, s), 5.31 (1H, br. s, N-H), 4.60-4.58 (1H, m), 4.13 (1H, app. t, *J* 10.0), 3.77 (1H, dd, *J* 10.5, 6.8), 3.50 (1H, app. q, *J* 14.7, 7.2), 3.43 (1H, app. t, *J* 9.3), 3.27 (1H, dd, *J* 8.5, 2.3), 2.27-2.21 (1H, m), 1.73 (1H, app. oct, *J* 33.0, 27.1, 20.0, 13.6, 6.8), 1.41 (9H, s), 1.40-1.35 (1H, m), 1.21-1.16 (1H, m), 0.98 (6H, d, *J* 7.4), 0.95 (3H, d, *J* 7.0), 0.79 (3H, d, *J* 6.8); δ_C (151 MHz, $CDCl_3$): 158.3, 158.1, 157.6, 156.9, 156.1, 96.2, 54.8, 53.9, 47.6, 40.5, 35.3, 33.3, 27.7, 25.8, 18.2, 17.8, 12.0, 11.8; HRMS (ESI+): found 403.2805; $C_{21}H_{35}N_6O_2$, $[M+H]^+$ requires 403.2821; v_{max} (thin film): 3339.7, 2963.2, 2929.7, 2873.8, 1729.5, 1576.7, 1535.7, 1442.5, 139.4, 1364.2, 1323.2, 1237.5, 1077.2, 1047.4, 972.8, 875.9, 846.1, 805.1, 756.6 cm^{-1}; $[\alpha]_D^{20}$ +49.8 (*c* = 1.5, $CHCl_3$).

(*S*)-4-((*S*)-*sec*-butyl)-1-(*tert*-butyl)-3-(6-((*S*)-3-(6-chloropyrimidin-4-yl)-4-isopropyl-2-oxoimidazolidin-1-yl)pyrimidin-4-yl)imidazolidin-2-one (2e)

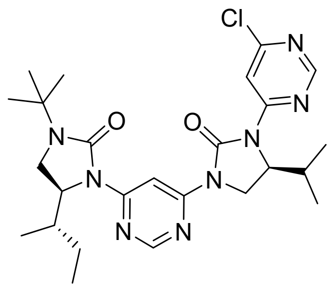

Prepared according to **General Procedure B** using *N*-nosyl deprotected dimer **S33** (0.32 g, 0.79 mmol), 4,6-dichloropyrimidine (0.82 g, 5.53 mmol), $Pd_2(dba)_3$ (40 mg, 40 µmol), Xantphos (70 mg, 0.12 mmol), toluene (10 mL), and Cs_2CO_3 (0.64 g, 1.98 mmol). Reaction time = 16 h. Purification by flash column chromatography (silica gel, petrol:ethyl acetate, 10:1 → 5:1) afforded the chloropyrimidine **2e** (0.33 g, 82%) as a yellow crystalline solid. δ_H (600 MHz, $CDCl_3$): 9.16 (1H, s), 8.65 (1H, s), 8.56 (2H, s), 4.74 (1H, dt, *J* 9.1, 2.7), 4.62 (1H, dt, *J* 9.3, 3.0), 4.10 (1H, dd, *J* 11.2, 2.3), 3.95 (1H, app. t, *J* 10.2), 3.46 (1H, app. t, *J* 9.4), 3.31 (1H, dd, *J* 9.2, 2.3), 2.57-2.54 (1H, m), 2.30-2.25 (1H, m), 1.44 (9H, s), 1.41-1.37 (1H, m), 1.23-1.21 (1H, m), 1.01 (3H, d, *J* 7.0), 0.99 (3H, app. t, *J* 7.4), 0.81 (3H, d, *J* 6.8), 0.77 (3H, d, *J* 6.8); δ_C (151 MHz, $CDCl_3$): 161.5, 158.7, 158.4, 157.8, 157.1, 157.1, 156.1, 153.3, 109.9, 96.6, 55.6, 54.0, 53.9, 41.4, 40.5, 35.2, 28.3, 27.8, 25.8, 18.2, 14.1, 12.1, 11.8; HRMS (ESI+): found 553.2205; $C_{25}H_{35}N_8O_2ClK$, $[M+K]^+$ requires 553.2209; v_{max} (thin film): 2959.5, 2873.8, 1714.6, 1565.5, 1531.9, 1461.1, 1435.0, 1386.6, 1274.7, 1241.2, 1200.2, 1110.7, 984.0, 875.9, 812.6, 764.1, 678.4 cm^{-1}; $[\alpha]_D^{20}$ +10.7 (*c* = 2.1, $CHCl_3$).

(*S*)-4-((*S*)-*sec*-butyl)-1-(*tert*-butyl)-3-(6-((*S*)-4-isopropyl-3-(6-((*S*)-4-methyl-3-((2-nitrophenyl)sulfonyl)-2-oxoimidazolidin-1-yl)pyrimidin-4-yl)-2-oxoimidazolidin-1-yl)pyrimidin-4-yl)imidazolidin-2-one (S34)

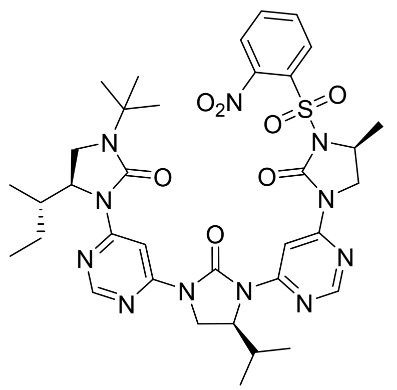

Prepared according to **General Procedure B** using chloropyrimidine **2e** (0.33 g, 0.65 mmol), imidazolidin-2-one **7** (0.28 g, 0.97 mmol) [added in 2 portions at reaction time = 0 h and at 1 h], $Pd_2(dba)_3$ (30 mg, 30 μmol), Xantphos (60 mg, 0.10 mol), toluene (10 mL), and K_2CO_3 (0.22 g, 1.63 mmol). Reaction time = 16 h. Purification by flash column chromatography (silica gel, dichloromethane → dichloromethane:ethyl acetate 6:1) afforded the di-protected trimer **S34** (0.47 g, 95 %) as a pale yellow solid. δ_H (600 MHz, $CDCl_3$): 9.04 (1H, s), 8.88 (1H, s), 8.58 (1H, s), 8.55-8.54 (2H, m), 7.79 (1H, t, *J* 7.3), 7.75-7.71 (2H, m), 4.72 (1H, dt, *J* 9.2, 6.0), 4.68-4.66 (1H, m), 4.61 (1H, dt, *J* 9.4, 2.8), 4.26 (1H, t, *J* 9.7), 4.02 (1H, dd, *J* 11.1, 2.0), 3.90 (1H, t, *J* 10.0), 3.86 (1H, dd, *J* 10.6, 1.9), 3.46 (1H, t, *J* 9.3), 3.31 (1H, dd, 8.9, 2.2), 2.54-2.49 (1H, m), 2.31-2.24 (1H, m), 1.65 (3H, d, *J* 6.3), 1.46 (9H, s), 1.41-1.36 (1H, m), 1.23-1.18 (1H, m), 0.99 (6H, app. t, *J* 6.7), 0.82 (3H, d, *J* 6.8), 0.75 (3H, d, *J* 6.8); δ_C (151 MHz, $CDCl_3$): 158.6, 158.1, 157.7, 157.4, 157.1, 157.0, 156.0, 153.2, 150.4, 148.0, 135.5, 135.0, 132.4, 131.5, 124.3, 98.4, 97.0, 55.6, 54.0, 53.9, 51.1, 49.4, 41.3, 40.5, 35.2, 28.4, 27.8, 25.8, 22.9, 18.1, 14.2, 12.1, 11.8; HRMS (ESI+): found 802.2885; $C_{35}H_{45}N_{11}O_7SK$, $[M+K]^+$ requires 802.2861; ν_{max} (thin film): 2992.2, 2851.4, 1733.2, 1580.4, 1539.4, 1446.2, 1367.9, 1278.5, 1215.1, 1174.1, 1110.7, 875.9, 749.2 cm^{-1}; $[\alpha]_D^{20}$ +5.5 (*c* = 2.1, $CHCl_3$).

(*S*)-4-((*S*)-*sec*-butyl)-1-(*tert*-butyl)-3-(6-((*S*)-4-isopropyl-3-(6-((*S*)-4-methyl-2-oxoimidazolidin-1-yl)pyrimidin-4-yl)-2-oxoimidazolidin-1-yl)pyrimidin-4-yl)imidazolidin-2-one (S35)

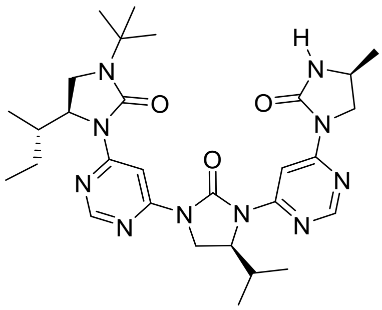

Prepared according to **General Procedure A** using di-protected trimer **S34** (0.47 g, 0.62 mmol), thiophenol (90 μL, 0.93 mmol), K_2CO_3 (0.26 g, 1.86 mmol) and *N*,*N*-DMF (10 mL). Reaction time = 20 h. Purification by flash column chromatography (silica gel, dichloromethane → 1% methanol:dichloromethane) afforded **S35** (41 g, 52 %) as a pale yellow crystalline solid. δ_H (600 MHz, $CDCl_3$): 9.09 (1H, s), 9.01 (1H, s), 8.54 (1H, s), 8.53 (1H, s), 6.18 (1H, br. s), 4.76 (1H, dt, *J* 9.0, 2.9), 4.59 (1H, dt, *J* 9.3, 2.8), 4.22 (1H, dd, *J* 10.5, 8.7), 4.03 (1H, dd, *J* 11.0, 3.5), 4.0-3.9 (2H, m), 3.69 (1H, dd, *J* 10.1, 6.6), 3.43 (1H, t, *J* 9.3), 3.28 (1H, dd, *J* 8.5, 1.6), 2.54-2.49 (1H, m), 2.28-2.23 (1H, m), 1.40 (10H, app. s), 1.36 (3H, d, *J* 5.8), 1.22-1.17 (1H, m), 0.99-0.96 (6H, m), 0.79 (3H, d, *J* 6.8), 0.77 (3H, d, *J* 6.8); δ_C (151 MHz, $CDCl_3$): 158.8, 158.6, 157.6, 157.6, 157.2, 156.9, 156.9, 155.9, 153.3, 97.8, 97.1, 55.4, 54.0, 53.9, 51.2, 44.8, 41.3, 40.5, 35.2, 28.5, 27.7, 25.9, 21.9, 18.2, 14.3, 12.1, 11.8; HRMS (ESI+): found 617.3064; $C_{29}H_{42}N_{10}O_3K$, $[M+K]^+$ requires 617.3078; ν_{max} (thin film): 3321.1, 2959.5, 2922.2, 2873.8, 2359.4, 1718.3, 1576.7, 1446.2, 1378.1, 1274.7, 1237.5, 1274.7, 1110.7, 1073.5, 879.7, 756.6, 670.9 cm^{-1}; $[\alpha]_D^{20}$ +63.7 (*c* = 0.6, $CHCl_3$).

(*S*)-4-((*S*)-*sec*-butyl)-1-(*tert*-butyl)-3-(6-((*S*)-3-(6-((*S*)-3-(6-chloropyrimidin-4-yl)-4-methyl-2-oxoimidazolidin-1-yl)pyrimidin-4-yl)-4-isopropyl-2-oxoimidazolidin-1-yl)pyrimidin-4-yl)imidazolidin-2-one (S36)

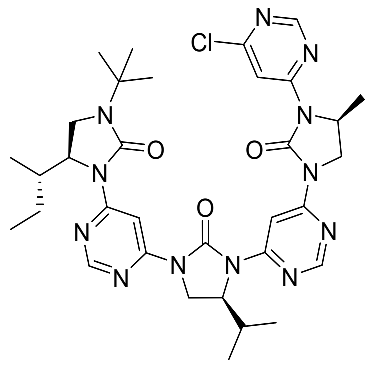

Prepared according to **General Procedure B** using *N*-nosyl deprotected trimer **S35** (0.26 g, 0.46 mol), 4,6-dichloropyrimidine (0.61 g, 4.11 mmol), $Pd_2(dba)_3$ (21 mg, 23 µmol), Xantphos (40 mg, 70 µmol), toluene (5 mL), and K_2CO_3 (0.16 g, 1.14 mmol). Reaction time = 16 h. After 16 h the reaction had not reached completion, a second portion of $Pd_2(dba)_3$ (10 mg, 12 µmol) and Xantphos (20 mg, 35 µmol) was added. Reaction time = 7 h. Purification by flash column chromatography (silica gel, petrol:ethyl acetate, 10:1 → 5:1 → 1:1) afforded the chloropyrimidine **S36** (0.28 g, 90%) as a pale yellow crystalline solid. δ_H (600 MHz, $CDCl_3$): 9.17 (1H, d, 1.0), 9.09 (1H, d, *J* 0.9), 8.67 (1H, d, *J* 0.8), 8.62 (1H, d, *J* 1.0), 8.56 (1H, d, *J* 0.9), 8.49 (1H, d, 0.8), 4.86 (1H, pent. d, *J* 27.6, 21.3, 15.2, 12.5, 6.1, 3.0), 4.77 (1H, dt, *J* 9.5, 3.4), 4.60 (1H, dt, *J* 9.6, 3.2), 4.18 (1H, dd, *J* 10.6, 9.0), 4.07 (1H, dd, *J* 10.9, 3.2). 4.00 (1H, dd, *J* 10.7, 9.5), 3.93 (1H, dd, *J* 10.6, 2.8), 3.45 (1H, t, *J* 9.2), 3.30 (1H, dd, *J* 9.1, 3.2), 2.60-2.53 (1H, m), 2.32-2.25 (1H, m), 1.50 (3H, d, *J* 6.3), 1.43 (9H, s), 1.22-1.19 (1H, m), 1.02 (3H, d, *J* 7.0), 0.99 (3H, t, *J* 7.5), 0.80 (6H, t, *J* 7.1); δ_C (151 MHz, $CDCl_3$): 161.4, 158.7, 158.2, 158.1, 158.0, 157.8, 157.4, 157.1, 156.9, 155.9, 153.3, 152.6, 109.8, 98.2, 97.2, 55.6, 54.0, 54.0, 48.3, 48.0, 41.4, 40.5, 35.2, 28.5, 27.7, 25.8, 20.2, 18.2, 14.2, 12.1, 11.8; HRMS (ESI+): found 729.2830; $C_{33}H_{43}N_{12}O_3ClK$, $[M+K]^+$ requires 729.2907; ν_{max} (thin film): 2922.2, 2959.5, 1714.6, 1561.8, 1528.2, 1438.8, 1394.0, 1364.2, 1278.5, 1237.5, 1207.7, 1110.7, 984.0, 875.9, 741.7, 670.9 cm^{-1}; $[\alpha]_D^{20}$ +28.9 (*c* = 2.0, $CHCl_3$).

(*S*)-1-(6-((*S*)-5-((*S*)-sec-butyl)-2-oxoimidazolidin-1-yl)pyrimidin-4-yl)-3-(6-((*S*)-3-(6-chloropyrimidin-4-yl)-4-methyl-2-oxoimidazolidin-1-yl)pyrimidin-4-yl)-4-isopropylimidazolidin-2-one (4b)

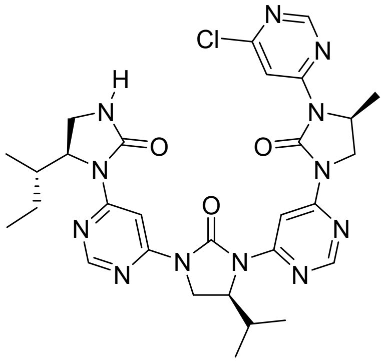

At room temperature, trifluoroacetic acid (5 mL) was added to the chloropyrimidine **S36** (37 mg, 54 µmol). The reaction mixture was stirred and trifluoromethanesulfonic acid (1 mL) was subsequently added. When TLC analysis showed all starting material had been consumed, excess trifluoroacetic acid and trifluoromethanesulfonic acid was removed by running a compressed air line over the solution for 1 h. The resulting crude brown residue was taken up in dichloromethane and washed with $NaHCO_3$. The layers were separated, and the aqueous layer was further extracted with dichloromethane (2 x 10 mL). The combined organic extracts were dried over anhydrous magnesium sulfate, filtered, and concentrated *in vacuo*. The crude solid was used without any further purification due to the reaction size and inherent loss of mass during trituration. The *tert*-butyl deprotected trimer **4b** (34 mg, >99%) was obtained as a pale yellow crystalline solid. δ_H (400 MHz, $CDCl_3$): 9.17 (1H, s), 9.06 (1H, s), 8.66 (1H, s), 8.62 (1H, d, *J* 0.9), 8.58 (1H, s), 8.46 (1H, s), 5.36 (1H, br. s, N-H), 4.89-4.86 (1H, m), 4.83 (1H, dt, *J* 9.5, 3.4), 4.78 (1H, dt, *J* 9.4, 3.3), 4.19 (1H, dd, *J* 10.5, 9.0), 4.08 (1H, dd, *J* 11.0, 3.1), 4.0 (1H, dd, 10.8, 9.7), 3.93 (1H, dd, *J* 10.7, 2.6), 3.55 (1H, t, *J* 9.3), 3.35 (1H, dd,

J 9.0, 3.3), 2.59-2.53 (1H, m), 2.35-2.29 (1H, m), 1.50 (3H, d, *J* 6.3), 1.39-1.34 (1H, m), 1.23-1.15 (1H, m), 1.01 (3H, d, *J* 7.0), 0.97 (3H, t, *J* 7.4), 0.84 (3H, d, *J* 6.9), 0.79 (3H, d, *J* 6.9) ; δ_C (101 MHz, $CDCl_3$): 161.6, 158.5, 158.3, 158.2, 158.1, 157.9, 157.6, 157.2, 157.1, 153.4, 152.6, 109.8, 98.1, 97.3, 58.1, 55.6, 48.3, 48.0, 41.4, 37.9, 35.2, 28.5, 25.7, 20.3, 18.2, 14.3, 12.0, 11.8; HRMS (ESI+): found 673.2280; $C_{29}H_{35}N_{12}O_3ClK$, $[M+K]^+$ requires 673.2281; v_{max} (thin film): 2959.5, 2922.2, 2855.1, 1729.5, 1565.5, 1531.9, 1423.8, 1367.9, 1207.7, 1110.7, 984.0, 875.9, 771.6, 741.7, 708.2 cm^{-1}; $[\alpha]_D^{20}$ +6.1 (*c* = 1.8, $CHCl_3$).

2.5 Synthesis of Macrocycles

Macrocyclic Tetramer 3a

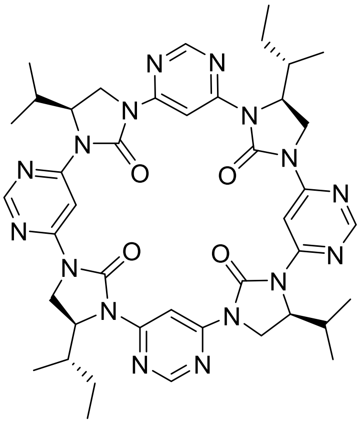

To a sealed tube equipped with a magnetic stir bar was added dimer **2a** (72 mg, 0.16 mmol), freshly recrystallized $Pd_2(dba)_3$ (4 mg, 4 μmol), and Xantphos (7 mg, 12 μmol). Anhydrous toluene (10 mL) was added to the flask, and the resulting suspension was degassed by sparging with nitrogen gas for 30 min. Cs_2CO_3 (60 mg, 0.20 mmol) was then added in one portion to the flask, and the reaction mixture was heated to reflux under a nitrogen atmosphere. Reaction time = 16 h. After complete consumption of the urea starting material by TLC analysis (petrol:ethyl acetate, 1:1), the reaction was cooled to room temperature, diluted with dichloromethane (20 mL) and filtered over Celite®. The crude diluted product was then washed in distilled water for 4 h to displace any Cs^+ that was bound inside the macrocycle cavity. The layers were then separated and the aqueous layers extracted with dichloromethane (2 x 30 mL). The combined organic extracts were dried over anhydrous magnesium sulfate, filtered, and concentrated *in vacuo*. The crude solid was purified by trituration in hot diethyl ether to afford the macrocyclic tetramer **3a** (37 mg, 56%) as an off-white crystalline solid. δ_H (600 MHz, $CDCl_3$): 9.97 (4H, app. d, *J* 2.4), 8.60 (4H, d, *J* 3.9), 4.83 (2H, dt, *J* 9.9, 3.1), 4.73 (2H, dt, *J* 8.9, 2.6), 4.06-3.98 (8H, m), 2.68-2.62 (2H, m), 2.44-2.39 (2H, m), 1.50-1.41 (2H, m), 1.35-1.27 (2H, m), 1.01 (12H, dd, *J* 6.8, 2.5), 0.78 (12H, dd, *J* 12.2, 7.0); δ_C (151 MHz, $CDCl_3$): 158.2, 158.1, 157.9, 157.8, 157.2, 157.1, 153.6, 153.5, 97.9, 97.8, 55.4, 54.3, 40.8, 40.7, 35.2, 28.5, 25.7, 18.2, 14.0, 12.0, 11.4; HRMS (ESI+): found 883.3961; $C_{42}H_{52}N_{16}O_4K$, $[M+K]^+$ requires 883.3995; v_{max} (thin film): 3190.6, 2959.5, 2873.8, 1729.5, 1580.4, 1476.0, 1423.8, 1364.2, 1259.8, 1215.1, 1114.5, 987.7, 902.0, 723.1, 682.1 cm^{-1}; $[\alpha]_D^{20}$ +6.5 (*c* = 0.2, $CHCl_3$). Diffraction-quality crystals were grown by the vapour diffusion method ($CHCl_3$/MeOH).

Macrocyclic Tetramer 3b

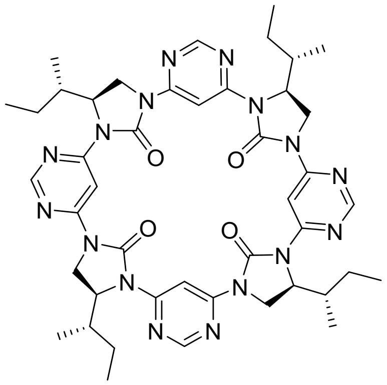

To a sealed tube equipped with a magnetic stir bar was added dimer **2b** (50 mg, 0.11 mmol), freshly recrystallized $Pd_2(dba)_3$ (5 mg, 5 μmol), and Xantphos (9 mg, 16 μmol). Anhydrous toluene (5 mL) was added to the flask, and the resulting suspension was degassed by sparging with nitrogen gas for 30 min. K_2CO_3 (40 mg, 0.27 mmol) was then added in one portion to the flask, and the reaction mixture was heated to reflux under a nitrogen atmosphere. Reaction time = 16 h. After complete consumption of the urea starting material by TLC analysis (petrol:ethyl acetate, 1:1), the reaction was cooled to room temperature, diluted with dichloromethane (20 mL) and filtered over Celite®. The crude diluted product was then washed in distilled water for 4 h to displace any Cs^+ that was bound inside the macrocycle cavity. The layers were then separated and the aqueous layers extracted with dichloromethane (2 x 30 mL). The combined organic extracts were dried over anhydrous magnesium sulfate, filtered, and concentrated *in vacuo*. The crude solid was purified by trituration in hot diethyl ether to afford the macrocyclic tetramer **3b** (28.4 mg, 61%) as an off-white crystalline solid. δ_H (600 MHz, $CDCl_3$): 9.97 (4H, s, H3), 8.61 (4H, s, H1), 4.84 (4H, dt, *J* 9.1, 3.0, H7), 4.03 (4H, dd, *J* 10.8, 2.7, H6), 3.98 (4H, t, *J* 10.0, H6'), 2.45-2.38 (4H, m, H8), 1.48 (4H, ddq, *J* 13.5, 7.3, 6.9, H9), 1.36-1.28 (4H, m, H9'), 1.02 (12H, t, *J* 7.4, H10), 0.78 (12H, d, *J* 6.9, H11); δ_C (151 MHz, $CDCl_3$): 158.0, 157.7, 157.0 (C1), 153.5, 97.8 (C3), 54.2 (C7), 40.7 (C6), 35.0 (C8), 25.5 (C9), 11.9 (C10), 11.3 (C11); HRMS (ESI+): found 911.4267; $C_{44}H_{56}N_{16}O_4K$, $[M+K]^+$ requires 911.4308; ν_{max} (thin film): 3183.1, 2963.2, 1736.9, 1584.1, 1431.3, 1394.0, 1263.6, 1222.6, 987.7, 887.1, 752.9, 685.8 cm^{-1}; $[\alpha]_D^{20}$ +16.5 (*c* = 0.3, $CHCl_3$). **Note:** The ^{1}H NMR spectrum of **3b** indicated the presence of a second, C_n-symmetrical species (Figure S1) in a ~6:1 ratio relative to the major compound. For example, two pyrimidine peaks at 9.44 and 8.78 ppm were observed, and the fact only two peaks were observed is indicative of a symmetrical molecule (as opposed to residual starting material or open-chain oligomers, which would lack this symmetry).

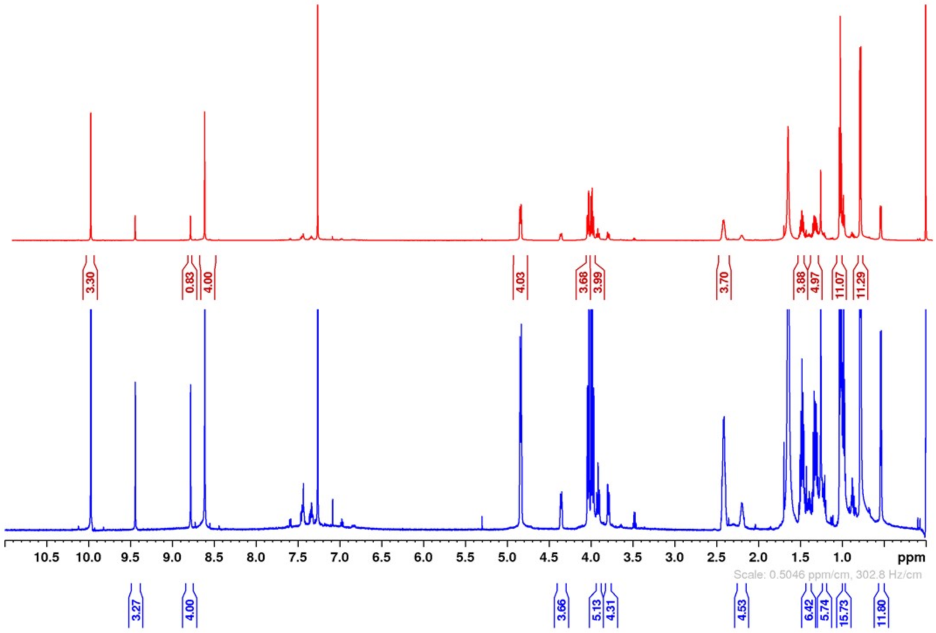

Figure S1. The spectrum of **3b** (600 MHz, $CDCl_3$) with major (top) and minor (bottom) resonances integrated.

NOESY analysis of the mixture showed in-phase cross-peaks between equivalent ^{1}H resonances in the major and minor components, indicating that these molecules undergo *chemical* exchange on the timescale of the NOESY experiment (Figure S2 below).

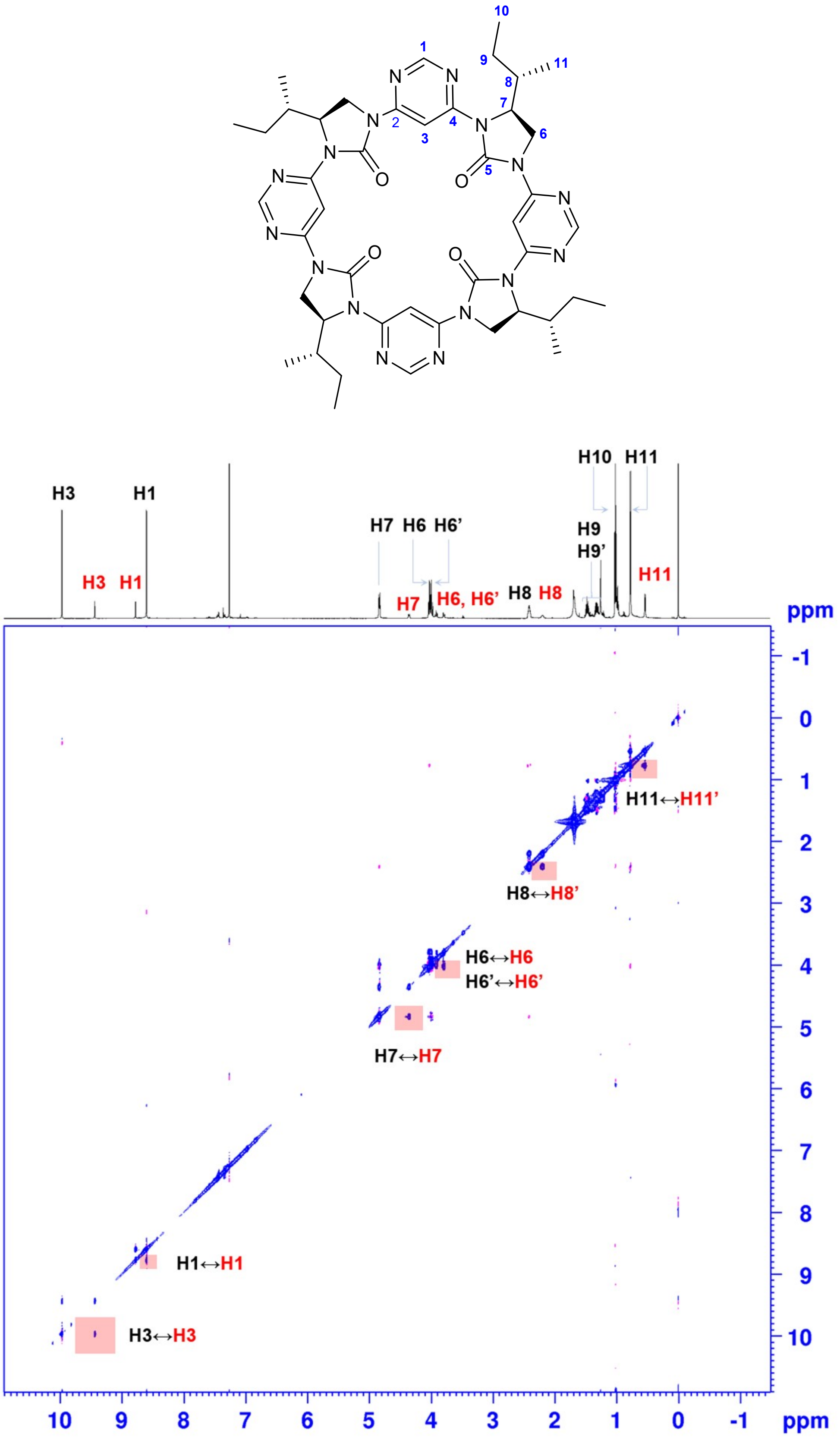

Figure S2. NOESY spectrum of **3b** (600 MHz, $CDCl_3$, t_{mix} 0.30 s). Structural assignments for peaks corresponding to the major (black) and minor (red) components are labelled on the 1D projection. Cross-peaks indicating chemical exchange processes are highlighted.

The observed chemical exchange may be due to the molecule populating two low energy interconverting conformers, or due to a reversible binding interaction between the macrocycle and an unknown guest. To rule out the latter, the macrocycle was placed in a much more competitive (Lewis basic) NMR solvent – d_5-pyridine. If an unknown guest were bound, it would be expected that the ratio of unoccupied:occupied macrocycle would increase. However, the minor component was still present in the same proportion when the ^{1}H NMR spectrum was acquired in a more competitive d_5-pyridine NMR solvent (Figure S3).

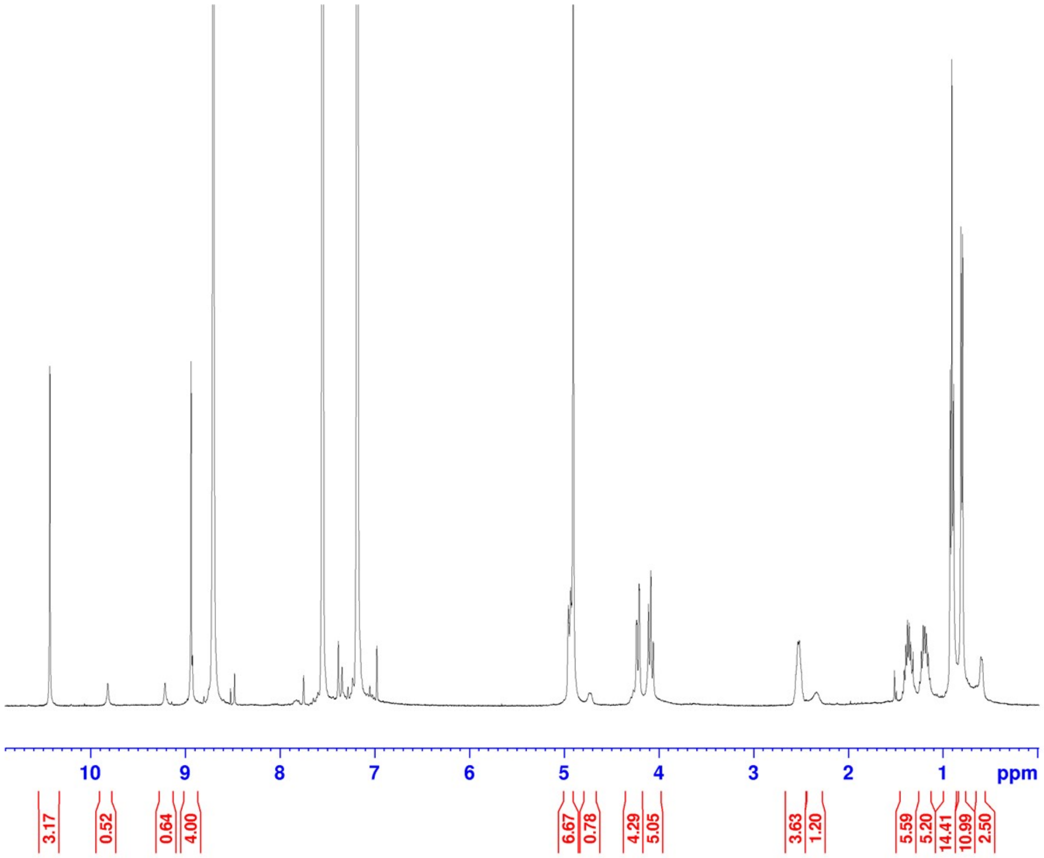

Figure S3. ^{1}H NMR spectrum of **3b** in d_5-pyridine (400 MHz).

In combination with the NOESY experiment, these data are consistent with the presence of two low energy conformations of the macrocycle.

Macrocyclic Tetramer 3c

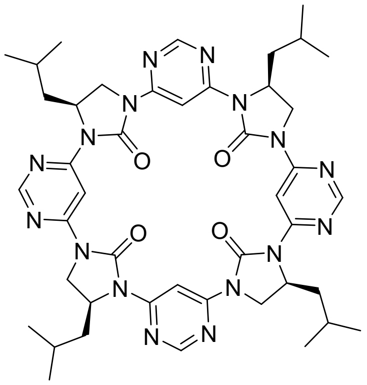

To a sealed tube equipped with a magnetic stir bar was added dimer **2c** (0.56 g, 1.18 mmol), freshly recrystallized $Pd_2(dba)_3$ (30 mg, 30 μmol), and Xantphos (50 mg, 90 μmol). Anhydrous toluene (59 mL) was added to the flask, and the resulting suspension was degassed by sparging with nitrogen gas for 30 min. Cs_2CO_3 (0.48 g, 1.48 mmol) was then added in one portion to the flask, and the reaction mixture was heated to reflux under a nitrogen atmosphere. Reaction

time = 16 h. After complete consumption of the urea starting material by TLC analysis (petrol:ethyl acetate, 1:1), the reaction was cooled to room temperature, diluted with dichloromethane (20 mL) and filtered over Celite®. The crude diluted product was then washed in distilled water for 4 h to displace any Cs^+ that was bound inside the macrocycle cavity. The layers were then separated and the aqueous layers extracted with dichloromethane (2 x 30 mL). The combined organic extracts were dried over anhydrous magnesium sulfate, filtered, and concentrated *in vacuo*. The crude solid was purified by trituration in hot diethyl ether to afford the macrocyclic tetramer **3c** (0.38 g, 74%) as an off-white crystalline solid. δ_H (600 MHz, $CDCl_3$): 9.89 (4H, d, *J* 0.8), 8.60 (4H, d, *J* 0.7), 4.81-4.78 (4H, m), 4.11 (4H, app. t, *J* 10.0), 4.01 (4H, dd, *J* 10.4, 2.2), 1.93-1.89 (4H, m), 1.82-1.75 (4H, m), 1.53-1.48 (4H, m), 1.05 (12H, d, *J* 6.5), 0.97 (12H, d, *J* 6.6); δ_C (151 MHz, $CDCl_3$): 158.4, 157.8, 157.2, 153.3, 97.9, 50.1, 45.9, 42.4, 25.0, 23.9, 21.8; HRMS (ESI+): found 890.5020; $C_{44}H_{60}N_{17}O_4$, $[M+NH_4]^+$ requires 890.50147; v_{max} (thin film): 2959.5, 2873.8, 1736.9, 1580.4, 1423.8, 1386.6, 1259.8, 1218.8, 1110.7, 987.7, 771.6, 670.9 cm^{-1}; $[\alpha]_D^{20}$ +3.2 (*c* = 0.7 , $CHCl_3$).

Macrocyclic Tetramer 3d

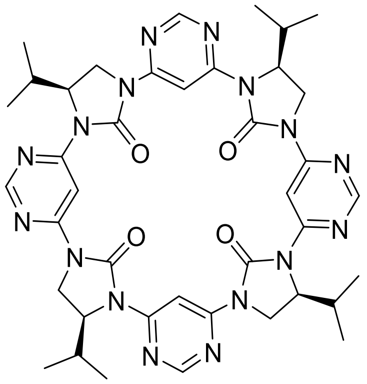

To a sealed tube equipped with a magnetic stir bar was added dimer **2d** (0.12 g, 0.26 mmol), freshly recrystallized $Pd_2(dba)_3$ (6 mg, 6 µmol), and Xantphos (11 mg, 20 µmol). Anhydrous toluene (13 mL) was added to the flask, and the resulting suspension was degassed by sparging with nitrogen gas for 15-30 min. Cs_2CO_3 (0.11 g, 0.33 mmol) was then added in one portion to the flask, and the reaction mixture was heated to reflux under a nitrogen atmosphere. Reaction time = 16 h. After complete consumption of the urea starting material by TLC analysis (petrol:ethyl acetate, 1:1), the reaction was cooled to room temperature, diluted with dichloromethane (20 mL) and filtered over Celite®. The crude diluted product was then washed in distilled water for 4 h to displace any Cs^+ that was bound inside the macrocycle cavity. The layers were then separated and the aqueous layers extracted with dichloromethane (2 x 20 mL). The combined organic extracts were dried over anhydrous magnesium sulfate, filtered, and concentrated *in vacuo*. The crude solid was purified by trituration in hot diethyl ether to afford the macrocyclic tetramer **3d** (91 mg, 85%) as an off-white crystalline solid. δ_H (600 MHz, $CDCl_3$): 9.98 (4H, s), 8.62 (4H, s), 4.74 (4H, dt, *J* 9.5, 3.0), 4.06 (4H, dd, *J* 10.9, 2.5), 3.97 (4H, app. t, *J* 10.2), 2.68-2.63 (4H, m), 1.04 (12H, d, *J* 7.0), 0.81 (12H, d, *J* 6.9); δ_C (151 MHz, $CDCl_3$): 158.2, 157.9, 157.2, 153.6, 97.9, 55.4, 40.7, 28.5, 18.2, 14.1; HRMS (ESI+): found 855.3653 $C_{40}H_{48}N_{16}O_4K$, $[M+K]^+$ requires 855.3682; v_{max} (thin film): 3183.1, 2959.5, 2873.8, 1729.5, 1580.4, 1423.8, 1386.6, 1282.2, 1259.8, 1218.8, 1114.5, 987.7, 730.6, 685.8 cm^{-1}; $[\alpha]_D^{20}$ +33.6 (*c* = 0.28, $CHCl_3$).

Macrocyclic Trimer 5a

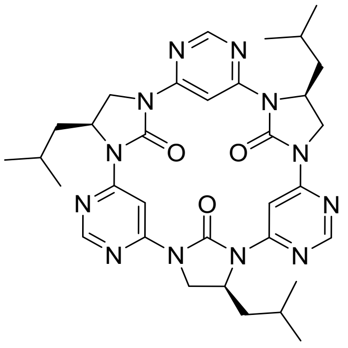

To a sealed tube equipped with a magnetic stir bar was added trimer **4a** (60 mg, 0.09 mmol), freshly recrystallized $Pd_2(dba)_3$ (4 mg, 3 µmol), and Xantphos (6 mg, 10 µmol). Anhydrous toluene (21 mL) was added to the flask, and the resulting suspension was degassed by sparging with nitrogen gas for 30 min. K_2CO_3 (22 mg, 0.16 mmol) was then added in one portion to the flask, and the reaction mixture was heated to reflux under a nitrogen atmosphere. Reaction time = 16 h. After complete consumption of the urea starting material by TLC analysis (petrol:ethyl acetate, 1:1), the reaction was cooled to room temperature, diluted with dichloromethane (20 mL) and filtered over Celite®. The crude diluted product was then washed in distilled water for 4 h to displace any Cs^+ that was bound inside the macrocycle cavity. The layers were then separated and the aqueous layers extracted with dichloromethane (2 x 30 mL). The combined organic extracts were dried over anhydrous magnesium sulfate, filtered, and concentrated *in vacuo*. Purification by flash column chromatography (silica gel, 1% methanol:dichloromethane) to afford the macrocyclic trimer **5a** (3.5 mg, 6%) as an off-white crystalline solid. δ_H (600 MHz, $CDCl_3$): 8.69 (3H, d, *J* 1.0), 8.50 (3H, d, *J* 0.9), 4.71-4.67 (3H, m), 4.18 (3H, app. dd, *J* 10.4, 8.8), 3.91 (3H, dd, *J* 10.6, 5.7), 1.91-1.87 (3H, m), 1.78-1.71 (3H, m), 1.01 (9H, d, *J* 6.6), 0.95 (9H, d, *J* 6.7); δ_C (151 MHz, $CDCl_3$): 158.6, 158.0, 157.4, 153.3, 101.3, 51.3, 47.1, 42.1, 24.9, 23.9, 21.8; HRMS (ESI+): found 655.3581; $C_{33}H_{43}N_{12}O_3$, $[M+H]^+$ requires 665.3576; ν_{max} (thin film): 2955.8, 2929.7, 2870.1, 1736.9, 1576.7, 1435.0, 1364.2, 1241.2, 1211.4, 987.7, 749.2, 682.1 cm^{-1}; $[\alpha]_D^{20}$ +23.7 (*c* = 0.4, $CHCl_3$).

Macrocyclic Trimer 5b

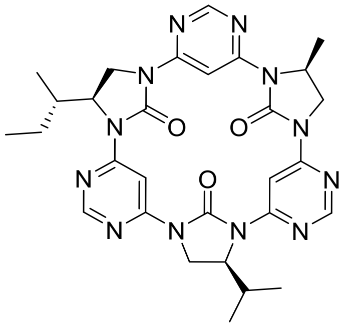

To a sealed tube equipped with a magnetic stir bar was added trimer **4b** (40 mg, 63 µmol), freshly recrystallized $Pd_2(dba)_3$ (3 mg, 3 µmol), and Xantphos (6 mg, 10 µmol). Anhydrous toluene (21 mL) was added to the flask, and the resulting suspension was degassed by sparging with nitrogen gas for 30 min. Cs_2CO_3 (50 mg, 0.16 mmol) was then added in one portion to the flask, and the reaction mixture was heated to reflux under a nitrogen atmosphere. Reaction time = 16 h. After complete consumption of the urea starting material by TLC analysis (ethyl acetate), the reaction was cooled to room temperature, diluted with dichloromethane (20 mL) and filtered over Celite®. The crude diluted product was then washed in distilled water for 4 h to displace any Cs^+ that was bound inside the macrocycle cavity. The layers were then separated and the aqueous layers extracted with dichloromethane (2 x 30 mL). The combined organic extracts were dried over anhydrous magnesium sulfate, filtered, and concentrated *in vacuo*. Purification by flash column chromatography (silica gel, 5% methanol:dichloromethane) to afford the macrocyclic trimer **5b** (11 mg, 29%) as an off-white crystalline solid. δ_H (400 MHz, $CDCl_3$): 8.72-8.71 (3H, m), 8.66 (1H, d, *J* 1.1), 8.49 (1H, d, *J* 1.1), 8.44 (1H, d, *J* 1.0), 4.81-4.76 (1H, m), 4.69-4.64 (2H, m), 4.20 (1H, app. dd, *J* 10.4, 8.6), 4.04-3.94 (4H, m), 3.83 (1H, dd, *J* 10.7, 7.2), 2.44-2.34 (1H, m), 2.24-2.17 (1H, m), 1.47-1.42 (4H, m), 1.30-1.21 (3H, m), 0.98 (6H, app. t, *J* 7.0), 0.79 (6H, app. dd,

J 6.9, 2.0); δ_C (151 MHz, $CDCl_3$): 158.5, 158.40, 158.38, 157.8, 157.7, 157.6, 157.41, 157.39, 157.3, 153.7, 153.5, 153.1, 101.8, 101.5, 101.3, 56.0, 55.0, 48.7, 48.5, 41.4, 41.3, 34.5, 27.5, 25.3, 19.2, 17.7, 14.0, 11.9, 11.5; HRMS (ESI+): found 599.2960; $C_{33}H_{43}N_{12}O_3$, $[M+H]^+$ requires 599.2950; ν_{max} (thin film): 2959.5, 2877.5, 1736.9, 1576.7, 1435.0, 1248.7, 987.7, 872.2, 730.6, 685.8 cm^{-1}; $[\alpha]_D^{20}$ +25.4 (*c* = 0.5, $CHCl_3$).

3 NMR Titration

3.1 Hexadecylammonium Chloride Binding

3.1.1 Host and Guest Solution Preparation

Macrocycle **3d** (2.30 mg, 2.8 μmol) was weighed accurately into a gas chromatography vial which was then fitted with a screw-top lid featuring a rubber septum to prevent evaporation of solvent. Deuterated chloroform (1.07 mL) was then added to the vial and sonicated briefly to ensure the macrocycle had been fully dissolved. The solution was split into two parts, the host solution (0.66 mL) which was transferred using a micro syringe into an NMR tube leaving the guest solution (0.41 mL) in the sealed gas chromatography vial. Hexadecyltrimethylammonium chloride (11.5 mg, 31.6 μmol) was added to the guest solution and sonicated.

3.1.2 NMR Titrations

An NMR of the free host solution was carried out to obtain a reference spectrum. The guest solution (2 μmL) was then added to the NMR tube and a second spectrum obtained. This process was repeated according to the Table S1 below to acquire a range of data points. All spectra were referenced to tetramethylsilane and the chemical shifts of the two pyrimidine C-H residues were measured.

Table S1: NMR Guest Titration Values

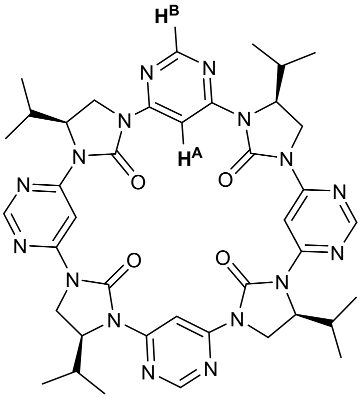

Vol Guest (mL)	Conc. Guest (M)	δ(H^A) / ppm	δ(H^B) / ppm
0.002	0.0002335	10.00185	8.63103
0.004	0.00046559	9.99813	8.63223
0.006	0.0006963	9.99462	8.6347
0.008	0.00092562	9.99027	8.63574
0.01	0.00115359	9.98596	8.63671
0.012	0.0013802	9.98153	8.63641
0.014	0.00160547	9.97726	8.63606
0.016	0.00182942	9.97512	8.63794
0.018	0.00205205	9.9739	8.63971
0.02	0.00227337	9.97018	8.63917
0.022	0.0024934	9.96773	8.64006
0.024	0.00271215	9.96449	8.63939
0.03	0.0033608	9.96243	8.63977
0.04	0.00441727	9.9567	8.64143
0.06	0.00644247	9.94844	8.64234
0.08	0.00835855	9.93626	8.64421
0.1	0.0101741	9.92784	8.64647
0.2	0.01798872	9.91795	8.64493
0.3	0.02417935	9.89376	8.64581
0.4	0.02920457	9.87964	8.64533

3.1.3 Fitting

Job's plot analysis of the binding between hexadecyltrimethylammonium chloride and the macrocycle **3d** suggested a simple 1:1 binding isotherm was not appropriate (Figure S4).

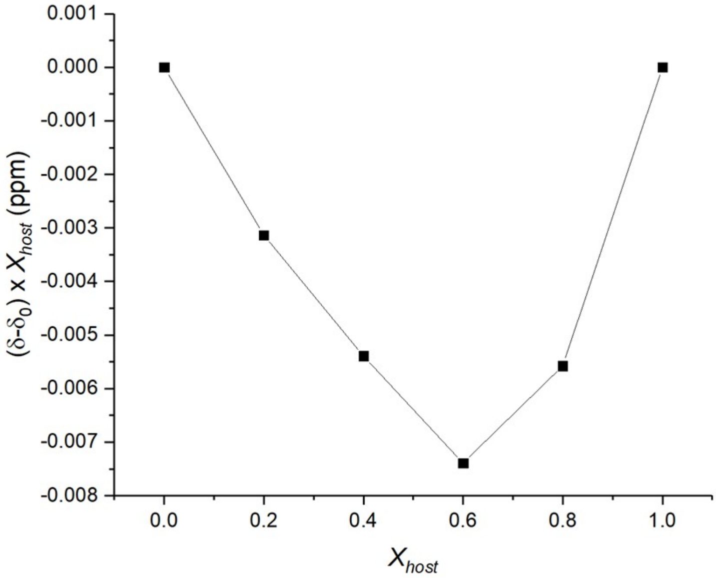

Figure S4. Job's plot for the binding of hexadecyltrimethylammonium chloride to macrocycle **3a**, determined by ^{1}H NMR ($CDCl_3$, 400 MHz).

NMR chemical shift data for H^A and H^B were fitted using Bindfit.[8,9] Fitting curves and residuals are shown in Figure S5.

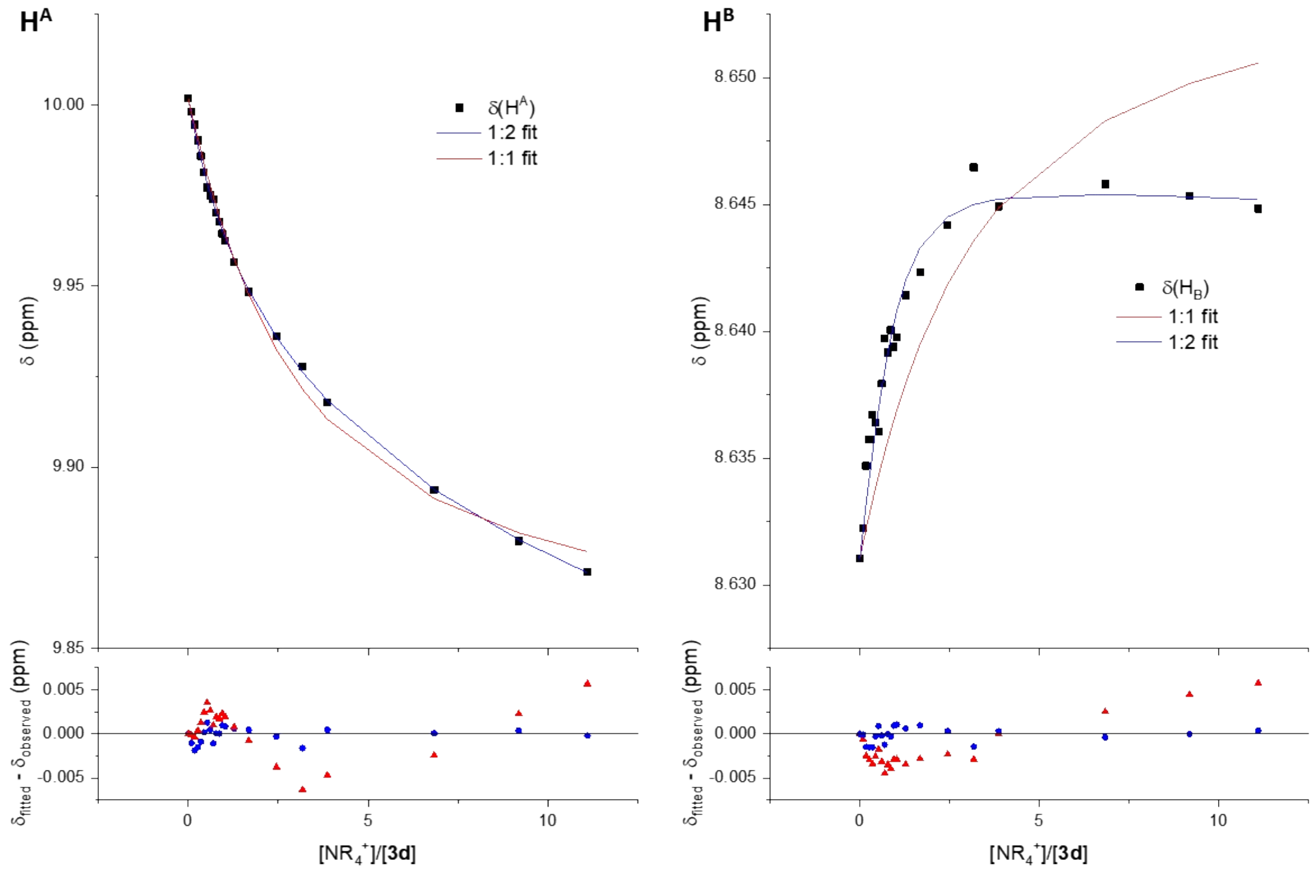

Figure S5. *Top*: ^{1}H NMR chemical shift values of H^A (left) and H^B (right) in macrocycle **3d** upon addition of dodecyltrimethylammonium chloride as described above. Measured chemical shifts are indicated by black squares. Fitted curves are overlaid for 1:1 (red) and 1:2 (blue) **3a**:NR_4^+ isotherm models. *Bottom*: residual curves for 1:1 (red triangle) and 1:2 (blue circle) binding isotherm models.

This analysis is suggestive of a 1:2 host:guest binding mode between **3a** and the ammonium salt, since when a 1:2 binding isotherm model is applied, the residuals (δ_{fitted}-$\delta_{observed}$) more closely resemble a random distribution around y = 0, as proposed by Jurczak.[10] Application of the 1:2 binding model leads to a first binding constant K_{11} = 1550 ± 120 M^{-1} and second K_{12} = 28 ± 1 M^{-1}. The robustness of these fitted parameters was verified by conducting the fit from a range of different initial guess values for K_{11} and K_{12}, and using both the Nelder-Mead and Limited-Memory Broyden-Fletcher-Goldfarb-Shanno with Box contstraint (L-BFGS-B) fitting algorithms (Table S2). In all cases, the algorithm converged on the same fitted binding constants.

Table S2. Fitted binding constants generated within Bindfit from a range of initial guesses and using different algorithmic methods. Raw outputs for each case are shown below.

	Initial Guess (M^{-1})		Fit Values (M^{-1})		Error (%)	
**Method**	**K_{11}**	**K_{12}**	**K_{11}**	**K_{12}**	**K_{11}**	**K_{12}**
Nelder-Mead	1	100	1554	28	7.7	2.6
Nelder-Mead	1	1	1554	28	7.7	2.6
Nelder-Mead	100	1	1554	28	7.7	2.6
L-BFGS-B	1	100	1554	28	7.7	2.6
L-BFGS-B	1	1	1554	28	7.7	2.6
L-BFGS-B	100	1	1554	28	7.7	2.6

Nelder-Mead output

Details

Time to fit 1.7940 s
SSR 2.8899e-5
Fitted datapoints 42
Fitted params 6

Parameters

Parameter (bounds)	Optimised	Error	Initial
K_{11} (0 → ∞)	1554.11 M^{-1}	± 7.6950 %	1.00 M^{-1}
K_{12} (0 → ∞)	28.12 M^{-1}	± 2.5941 %	100.00 M^{-1}

Details

Time to fit 1.4107 s
SSR 2.8899e-5
Fitted datapoints 42
Fitted params 6

Parameters

Parameter (bounds)	Optimised	Error	Initial
K_{11} (0 → ∞)	1554.11 M^{-1}	± 7.6950 %	1.00 M^{-1}
K_{12} (0 → ∞)	28.12 M^{-1}	± 2.5941 %	1.00 M^{-1}

Details

Time to fit 1.4531 s
SSR 2.8899e-5
Fitted datapoints 42
Fitted params 6

Parameters

Parameter (bounds)	Optimised	Error	Initial
K_{11} (0 → ∞)	1554.11 M^{-1}	± 7.6950 %	100.00 M^{-1}
K_{12} (0 → ∞)	28.12 M^{-1}	± 2.5941 %	1.00 M^{-1}

L-BFGS-B output

Details

Time to fit 1.0942 s
SSR 2.8899e-5
Fitted datapoints 42
Fitted params 6

Parameters

Parameter (bounds)	Optimised	Error	Initial
K_{11} (0 → ∞)	1553.78 M^{-1}	± 7.6940 %	1.00 M^{-1}
K_{12} (0 → ∞)	28.12 M^{-1}	± 2.5941 %	100.00 M^{-1}

Details

Time to fit 1.4351 s
SSR 2.8899e-5
Fitted datapoints 42
Fitted params 6

Parameters

Parameter (bounds)	Optimised	Error	Initial
K_{11} (0 → ∞)	1554.17 M^{-1}	± 7.6952 %	1.00 M^{-1}
K_{12} (0 → ∞)	28.12 M^{-1}	± 2.5941 %	1.00 M^{-1}

Details

Time to fit 1.2042 s
SSR 2.8899e-5
Fitted datapoints 42
Fitted params 6

Parameters

Parameter (bounds)	Optimised	Error	Initial
K_{11} (0 → ∞)	1554.30 M^{-1}	± 7.6956 %	100.00 M^{-1}
K_{12} (0 → ∞)	28.12 M^{-1}	± 2.5941 %	1.00 M^{-1}

The binding of the cationic ammonium salt by the macrocycle is believed to be mediated by C-H···O hydrogen bonds formed with between the acidified α-hydrogens of the ammonium salt and the imidazolidin-2-one carbonyl groups. Evidence for specific binding of the ammonium head-group (as opposed to e.g. hydrophobic effects) is found through examination of the chemical shift changes of the hexadecyltrimethylammonium salt itself. In the following discussion we follow the naming convention of Magid,[11] where the ^{1}H NMR chemical shift environments are divided into the head group (*hg*), *α*, *β*, *γ*', main chain (*mc*) and *ω* positions (Figure S6).

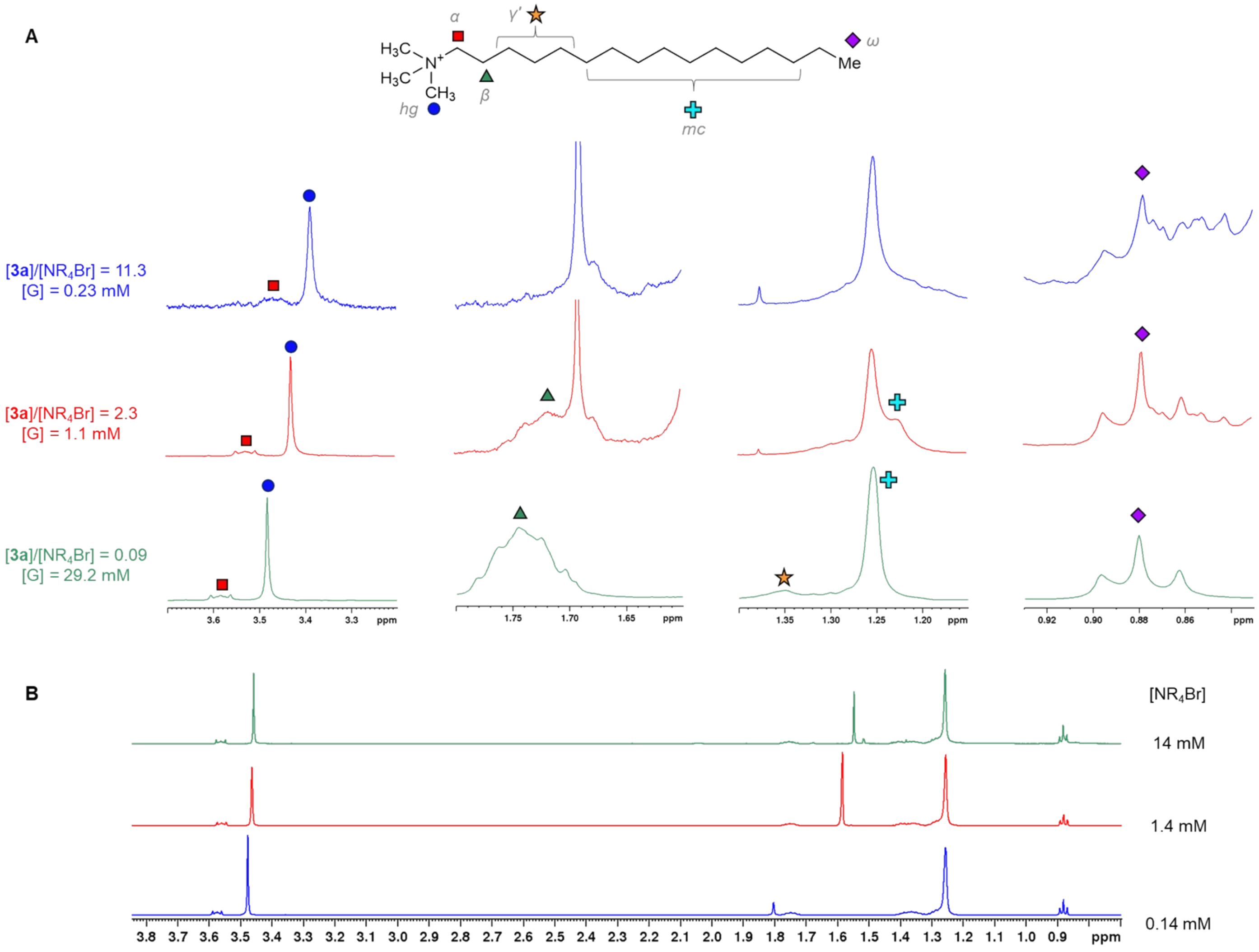

Figure S6. **A.** Evidence for interaction *via* N⁺C-H···O interactions. Peak regions within the hexadecylammonium salt (NR_4Br) are denoted as follows: head group (*hg*, blue circle), *α* (red square), *β* (green triangle), *γ'* (yellow star), *mc* (teal star), *ω* (purple diamond). **B**. Serial dilution of a solution of hexadecyltrimethylammonium bromide in $CDCl_3$ showing minimal change in chemical shift across a 100-fold change in concentration.

A upon addition of the host **3a**, a strong upfield shift is observed in the *hg*- and *α*-environments, with overall Δδ values across the concentration range investigated of 0.093 and 0.115 ppm respectively. While the precise chemical shifts of the *β*- and *γ'*-hydrogens are not discernible in the [H]/[G] = 11.3 sample, they display much smaller Δδ shifts than the *hg*- and *α*-hydrogens in the [H]/[G] = 2.3 relative to the [H]/[G] = 0.09 spectrum, as do the *mc*- and *ω*-hydrogens. These data are consistent with binding occurring primarily through hydrogen bonding at the *hg*- and *α*-hydrogens, which has previously been demonstrated to induce an upfield shift in the hydrogens adjacent to the tetrasubstituted nitrogen both in molecular recognition[12–16] and more recently in asymmetric catalysis[17,18] applications. Spectral excerpts are taken directly from the **3a**/NR_4^+Br titration described above, so host concentration is constant for all spectra while guest concentration is varied. The validity of this approach was confirmed by a dilution study on pure ammonium salt, which indicated minimal chemical shift change as a function of concentration (with the exception of the residual water peak) in $CDCl_3$ (Figure S6B). Small upfield shifts in *hg*- and *α*-hydrogens were observed upon increasing concentration (Δδ ≈ 0.02 ppm), but these changes are dwarfed by those seen in the titration experiment so can be disregarded.

3.2 Dibenzoyl Tartaric Acid (DBTA) Binding

3.2.1 Host and Guest Solution Preparation

Macrocycle **3b** (910 μg, 0.312 μmol) was dissolved in $CDCl_3$, made up to a total volume of 1.000 mL (0.312 μM), and the volumetric flask was sealed with parafilm and refrigerated to prevent evaporation.

Dibenzoyl-*L*-tartaric acid (500 mg, 1.40 mmol) was dissolved in $CDCl_3$, made up to a total volume of 10.00 mL (0.140 M), and the volumetric flask was sealed with parafilm and refrigerated to prevent evaporation. An identical method was used to prepare the stock solution of dibenzoyl-D-tartaric acid.

3.2.2 NMR Titrations

The ^{1}H NMR spectra of the macrocyles display no measurable change with concentration in the range 0 – 1 μM, so titrations were carried out on a single sample with aliquots of guest added incrementally.

The stock solution of **3b** (0.30 mL, 0.0936 μmol) was placed in an NMR tube and further diluted with $CDCl_3$ (0.30 mL) to a total volume of 0.60 mL. The dibenzoyl tartaric acid stock solutions were added without further dilution according to the schedule below.

Entry	Vol DBTA stock added (μL)	Total Vol DBTA stock added (μL)	Total amount DBTA added (μmol)	[DBTA]/[3b]
1	0	0	0	0
2	2.24	2.24	0.313	3.339658
3	2.24	4.48	0.625	6.679316
4	2.24	6.72	0.938	10.01897
5	2.24	8.96	1.250	13.35863
6	2.24	11.2	1.563	16.69829
7	2.24	13.44	1.876	20.03795
8	2.24	15.68	2.188	23.37761
9	2.24	17.92	2.501	26.71726
10	2.24	20.16	2.813	30.05692
11	2.24	22.4	3.126	33.39658
12	11.2	33.6	4.689	50.09487
13	11.2	44.8	6.252	66.79316
14	22.4	67.2	9.378	100.1897

The results of these titrations are displayed in figure S7 below. These indicate that the macrocycle does not differentiate between enantiomers of DBTA to any significant extent, with binding constants of 51 M^{-1} and 56 M^{-1} (for D and L-enantiomers respectively) the same within experimental error.

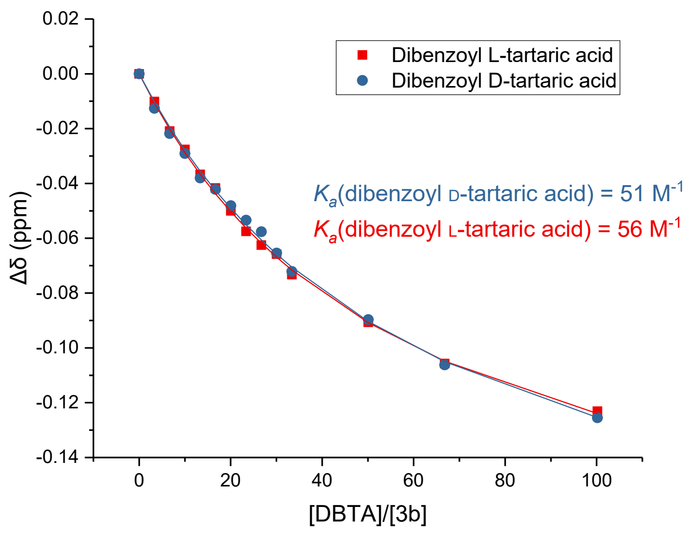

Figure S7. ^{1}H NMR titration data indicating chemical shift change in the inward-pointing pyrimidine hydrogen of **3b** (*cf.* H^A in Figure S1), upon addition of both enantiomers of dibenzoyl tartaric acid (DBTA). Individual datapoints represent raw data, and lines indicate fitting curves as produced by Bindfit according to a 1:1 binding model.

4 X-Ray Crystallography

Data for compounds **1a**, **2a**, **2d** and **3a** were collected as described in the General Experimental section. For full details on the collection and refinement of crystal data, please refer to the cif files, accessible from the ccdc using the accession numbers given below.

4.1 *t*-Bu Protected Dimer 1a (CCDC #2057484)

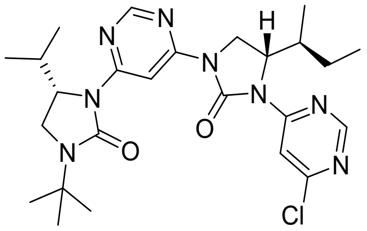

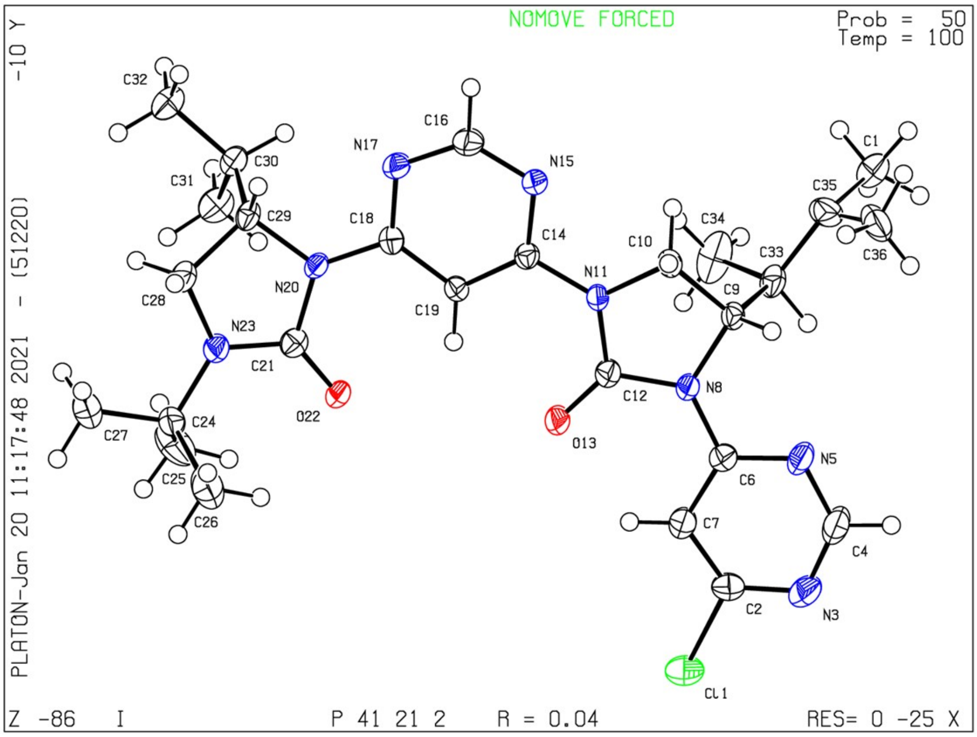

4.1.1 Crystal Data

a = 12.05260(10) Å α = 90°
b = 12.05260(10) Å β = 90°
c = 37.0309(5) Å γ = 90°

Volume	5379.30(12) Å³		Crystal Class	tetragonal
Space group	P 4_1 2_1 2		Z =	8
Formula	C_{25} H_{34} Cl N_8 O_2		M_r	514.05
Cell determined from	14408 reflections		Cell θ range =	5 - 71°
Temperature	100K			
Pressure	100 kPa	Shape	blocks	
Colour	clear_pale_colourless		Size	0.60 × 0.50 × 0.50 mm
D_x	1.27 Mg m^{-3}		F000	2184.000
μ	1.562 mm^{-1}			
Absorption correction	multi-scan			

T_{min}	0.26	T_{max}	0.39

4.1.2 Data Collection

Diffractometer	multi-scan
Scan type	ω scans
Reflections measured	22811
Independent reflections	5157
Rint	0.0306
θ_{max}	71.2738
h =	-14 → 14
k =	-9 → 14
l =	-45 → 35

4.1.3 Refinement

$\Delta\rho_{min}$ =	-0.22 e Å^{-3}	
$\Delta\rho_{max}$ =	0.34 e Å^{-3}	
Reflections used	5135	
Cutoff: I >	-3.00σ(I)	
Parameters refined	334	
S =	1.17	
R-factor	0.044	
weighted R-factor	0.058	
Δ/σ_{max}	0.0093	
Flack parameter	0.032(5)	Parsons, Flack & Wagner (2013)
Refinement on	F^2	

4.2 Dimer 2a (CCDC #2057483)

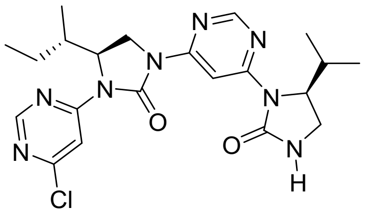

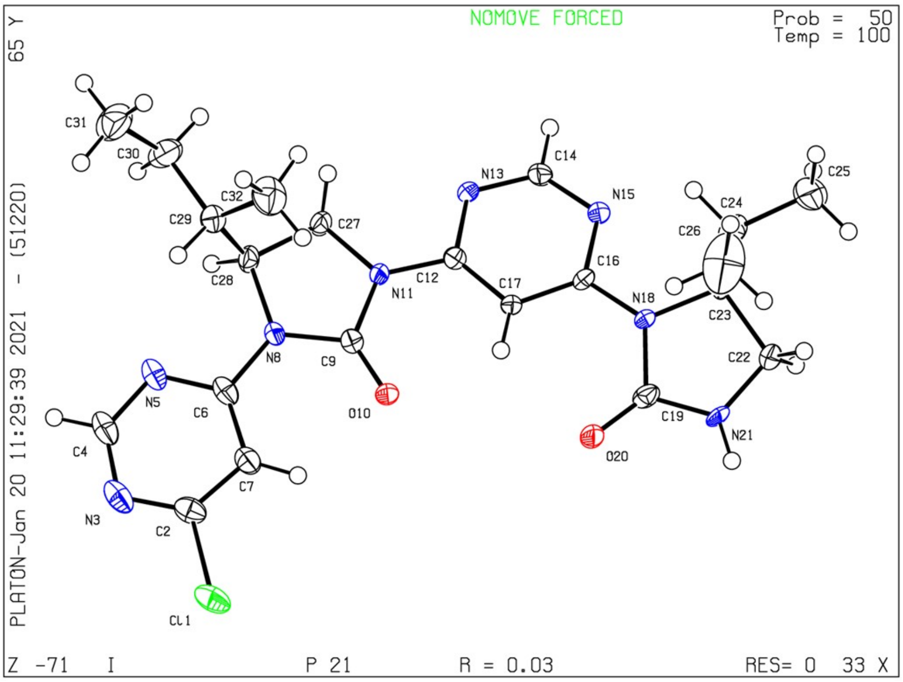

4.2.1 Crystal Data

a = 9.69330(10) Å   α = 90°
b = 9.01790(10) Å   β = 90.0868(8)°
c = 13.41980(10) Å γ = 90°

Volume	1173.07(2) Å³	Crystal Class	monoclinic
Space group	P 2_1	Z =	2
Formula	$C_{21} H_{27} Cl N_8 O_2$	M_r	458.9497
Cell determined from	9280 reflections	Cell θ range =	6 - 71°
Temperature	100K		
Pressure	100 kPa	Shape	needle
Colour	clear_pale_colourless	Size	0.50 × 0.40 × 0.40 mm
D_x	1.30 Mg m^{-3}	F000	484.000
μ	1.728 mm^{-1}		
Absorption correction	multi-scan		
T_{min}	0.32	T_{max}	0.42

4.2.2 Data Collection

Diffractometer	multi-scan
Scan type	ω scans
Reflections measured	10892
Independent reflections	3858
Rint	0.0271
θ_{max}	71.3843
h =	-11 → 11
k =	-10 → 7
l =	-16 → 16

4.2.3 Refinement

$\Delta\rho_{min}$ =	-0.20 e $Å^{-3}$
$\Delta\rho_{max}$ =	0.22 e $Å^{-3}$
Reflections used	3842

Cutoff: I >	-3.00σ(I)	
Parameters refined	294	
S =	1.95	
R-factor	0.034	
weighted R-factor	0.064	
Δ/σ_{max}	0.0099	
Flack parameter	0.015(5)	Parsons, Flack & Wagner (2013)
Refinement on	F^2	

4.3 Dimer 2d (CCDC #2057482)

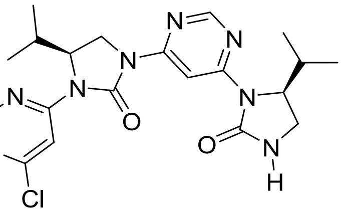

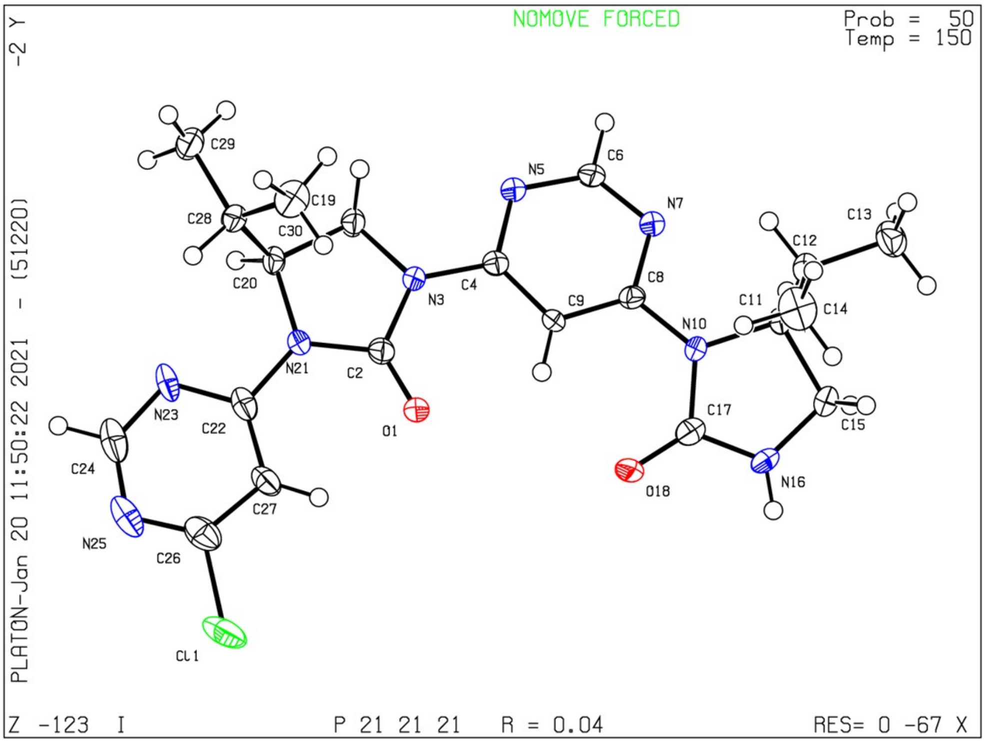

4.3.1 Crystal Data

a = 8.9540(2) Å α = 90°
b = 14.9196(3) Å β = 90°
c = 16.0420(3) Å γ = 90°

Volume	2143.05(8) Å³	Crystal Class	orthorhombic
Space group	P 2_1 2_1 2_1	Z =	4
Formula	C_{20} H_{25} Cl N_8 O_8	M_r	444.93
Cell determined from	4064 reflections	Cell θ range =	6 - 71°
Temperature	150K		
Pressure	100 kPa	Shape	needles

Colour	clear_pale_colourless	Size	0.40 × 0.15 × 0.10 mm
D_x	1.38 Mg m^{-3}	F000	936.000
μ	1.875 mm^{-1}		
Absorption correction	multi-scan		
T_{min}	0.36	T_{max}	0.47

4.3.2 Data Collection

Diffractometer	multi-scan
Scan type	ω scans
Reflections measured	6522
Independent reflections	4053
Rint	0.0265
θ_{max}	71.3775
h =	-7 → 10
k =	-17 → 18
l =	-18 → 19

4.3.3 Refinement

$\Delta\rho_{min}$ =	-0.20 e $Å^{-3}$	
$\Delta\rho_{max}$ =	0.31 e $Å^{-3}$	
Reflections used	4053	
Cutoff: I >	-3.00σ(I)	
Parameters refined	285	
S =	1.87	
R-factor	0.038	
weighted R-factor	0.043	
Δ/σ_{max}	0.0014	
Flack parameter	-0.003(10)	Parsons, Flack & Wagner (2013)
Refinement on	F^2	

4.4 Macrocyclic Tetramer 3a (CCDC #2057486)

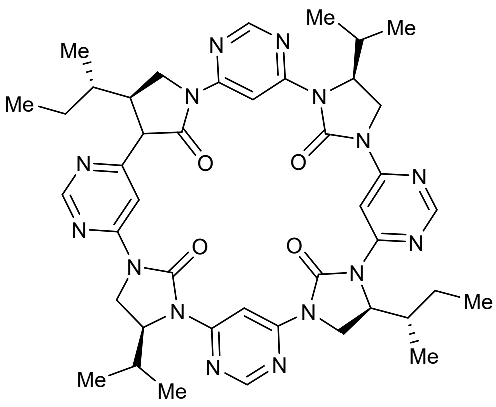

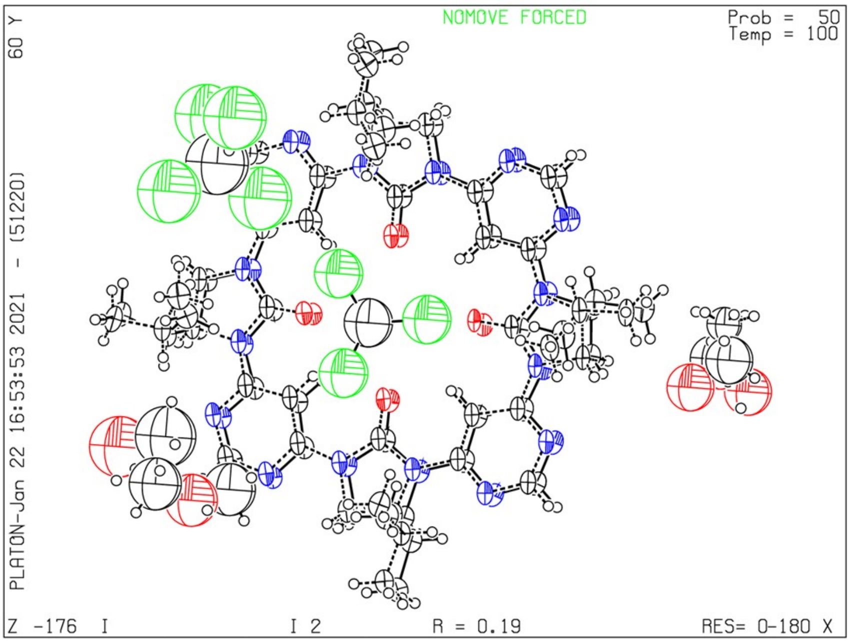

4.4.1 Crystal Data

a = 12.00486(4) Å	α = 90°		
b = 15.58877(3) Å	β = 96.150(5)°		
c = 23.79439(4) Å	γ = 90°		
Volume	5279.29(6) Å³	Crystal Class	monoclinic
Space group	I 2	Z =	4
Formula	$C_{42.64}$ $H_{56.23}$ $Cl_{3.11}$ N_{16} $O_{5.46}$	M_r	990.44
Cell determined from	4657 reflections	Cell θ range =	3 - 68°
Temperature	293K		
Pressure	100 kPa	Shape	plates
Colour	clear_pale_colourless	Size	0.22 × 0.14 × 0.005 mm
D_x	1.246 Mg m^{-3}	F000	2082.261
μ	2.098 mm^{-1}		
Absorption correction	multi-scan		
T_{min}	0.14	T_{max}	0.99

4.4.2 Data Collection

Diffractometer	multi-scan
Scan type	ω scans
Reflections measured	31995
Independent reflections	9165
Rint	0.114
θ_{max}	68.242
h =	-13 → 14
k =	-22 → 22
l =	-28 → 28

4.4.3 Refinement

$\Delta\rho_{min}$ =	-0.69 e $Å^{-3}$
$\Delta\rho_{max}$ =	0.71 e $Å^{-3}$

Reflections used	6744	
Cutoff: I >	-3.00σ(I)	
Parameters refined	406	
S =	1.7645	
R-factor	0.225	
weighted R-factor	0.498	
Δ/σ_{max}	0.037	
Flack parameter	0.22(4)	Parsons, Flack & Wagner (2013)
Refinement on	F^2	

Portions of the macrocyclic skeleton were found in the raw structure solution from Superflip. With the exception of the side chains, the rest of the structure was resolved by Fourier cycles. The refinement was not stable, and restraints were applied to bond distances following measurements from a previous structure (CCDC #1824642) corresponding to the repeating motif in the present structure. The first carbon of the side chains was then resolvable in the Fourier map, and in agreement with the expected chirality. Refinement of the anisotropic ADPs revealed that there was extensive disorder in the macrocycle position along the vector perpendicular to its plane. The molecule was then modelled over two positions, and with competitively refined occupancy. Further side chain structure then became visible, although it was not possible to distinguish between valine and isoleucine-derived monomers, with the additional methyl unit failing to appear or to refine to physically reasonable positions even when restrained. The anisotropic ADPs of both sets of atoms for the macrocycle were still prolate beyond physically reasonable limits, and so restrained refinement from isotropic ADPs was performed to account for electron density, before fixing the ADPs. The macrocycle could feasibly be modelled over more than two locations, but the qualitative description of the structure would not be more precise - there is extensive disorder in position perpendicular to the plane of the macrocycle. Hydrogen atoms were added geometrically, and the solvent was modelled by examination of slant Fourier maps. While we are confident of the location and orientation of the chloroform molecules, the ethanol molecules are more speculative. Solvent molecules were refined isotropically and with partial occupancies. Weights were selected and the structure was refined to convergence.

This is a low-quality structure and only information about connectivity, relative molecular position and orientation, and gross conformation of the main residue should be taken with any level of confidence. There are no fully ordered portions.

5 Density Functional Theory Calculations

All optimisation, frequency, and single-point calculations were performed with *Gaussian 16*, rev A.03.[19] Structures are displayed with CYLView.[20] The B3LYP functional was used for all geometry optimisations with the 6-31G(d,p) basis set on all atoms unless otherwise stated. Grimme's DFT-D3BJ correction was included in the optimisation procedure as well as single point calculations.[21] All optimised structures were confirmed as minima by the absence of imaginary harmonic frequencies. Further refinements to the electronic energies were made through single point calculations on the optimised geometries using the B3LYP functional with the 6-311+G(d,p) basis set for all atoms. Grimme's DFT-D3BJ correction and further corrections for bulk solvation through a polarisable continuum model (PCM) with description of toluene in all calculations were also incorporated. Free energies were determined from thermochemical corrections of the geometries applied to electronic energies. Coordinates for all minimized geometries are given below (§8, pg. 162)

5.1 Trimer Summary (See Figure 3B)

Two conformations of the trimer were found, both bowl shaped, with the R group sidechains in either pseudo-axial or equatorial positions. No other conformations, including a flat or staggered arrangement of each monomeric unit, could be found. The pseudo-equatorial conformation was consistently more stable. This was found to hold true for a number of different functionals employed during optimisation (Table S3) with relative energies very similar in each case.

Table S3. Relative energies of pseudo-axial and equatorial conformations R=Me trimer using different functionals during optimisation. B3LYP and B3PW91 include D3BJ corrections, M062X and ωb97xd do not. Single point energy calculations performed at B3LYP-D3BJ-$PCM_{Toluene}$/6-311+G(d,p) throughout.

Optimisation Functional	ddG (kcal mol^{-1})
B3LYP	5.8
B3PW91	6.0
M062X	6.6
ωb97xd	5.8

For the B3LYP functional, the effects of solvation and dispersion during singled point energy calculations were examined (Table S4). While the inclusion of solvation makes only a minor change in the relative energy, inclusion of dispersion corrections prompts a very large decrease in relative energies of the conformers, 4.2 kcal mol^{-1} for R=Me. Notably, the stabilisation offered by the inclusion of dispersion corrections increases as the size of the sidechain R groups increases and the dispersion level contacts between ends of the sidechain become more numerous.

Table S4. Effect of solvation and dispersion corrections on relative energies of pseudo-axial and equatorial conformations of trimers of various R groups.

	R=Me	R=ABC	R=iPr
SP	10.3	16.5	17.1
SP/$PCM_{Toluene}$	10.0	16.0	16.5
SP/D3BJ	6.2	6.4	3.2
SP/$PCM_{Toluene}$/D3BJ	5.8	6.0	2.6

5.2 Tetramer Summary (See Figure 3a)

A significant degree of conformational flexibility was observed, with many essentially isoenergetic conformations found, although all retained the shallow bowl conformation when R=Me, two examples are shown in Figure S8. The conformation when one carbonyl is on a different face to the other three is 0.17 kcal mol^{-1} higher in energy compared to when all are pointing towards the same face. All attempts at finding the pseudo-axial conformation of the tetramer optimised to the pseudo-equatorial.

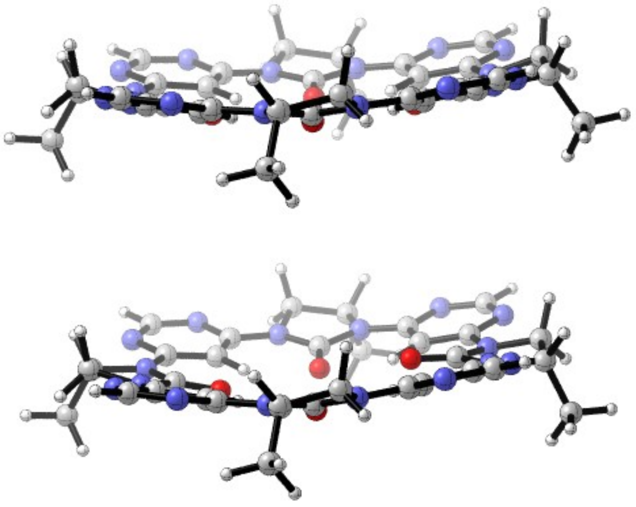

Figure S8. Examples of different shallow bowl conformations found when R=Me.

When R=H, one conformation was found which was entirely flat (Figure S9). O-O distances are slightly shorted in this conformation at 3.6 and 5.0 Å. However, this was 3.9 kcal mol^{-1} higher in energy than any other shallow bowl conformations, making this conformation highly unfavoured.

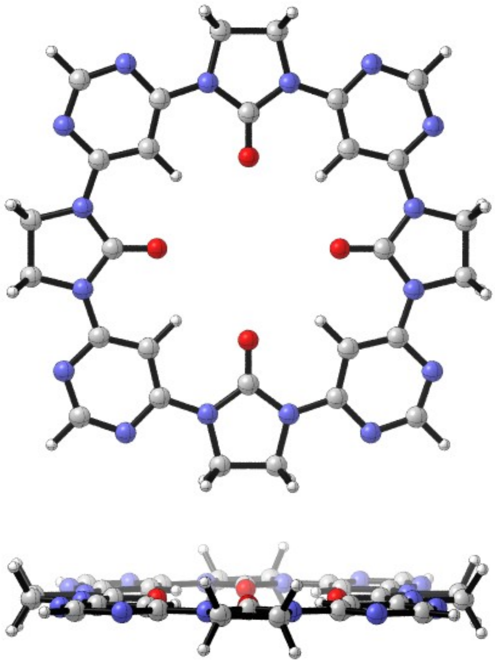

Figure S9. Tetramer flat conformation when R=H.

6 Supplementary References

[1] J. Cosier, A. M. Glazer, *J. Appl. Crystallogr.* **1986**, *19*, 105–107.

[2] *CrysAlisPRO*, Oxford Diffraction/Agilent Technologies UK Ltd, Yarnton, England, **n.d.**

[3] L. Palatinus, G. Chapuis, *J. Appl. Crystallogr.* **2007**, *40*, 786–790.

[4] P. W. Betteridge, J. R. Carruthers, R. I. Cooper, K. Prout, D. J. Watkin, *J. Appl. Crystallogr.* **2003**, *36*, 1487.

[5] R. I. Cooper, A. L. Thompson, D. J. Watkin, *J. Appl. Crystallogr.* **2008**, *43*, 1100–1107.

[6] Z. Lockhart, P. C. Knipe, *Angew. Chem. Int. Ed.* **2018**, *57*, 8478–8482.

[7] P. Jain, I. O. Raji, S. Chamakuri, K. R. MacKenzie, B. T. Ebright, C. Santini, D. W. Young, *J. Org. Chem.* **2019**, *84*, 6040–6064.

[8] P. Thordarson, *Chem. Soc. Rev.* **2011**, *40*, 1305–1323.

[9] www.supramolecular.org

[10] F. Ulatowski, K. Dabrowa, T. Bałakier, J. Jurczak, *J. Org. Chem.* **2016**, *81*, 1746–1756.

[11] P. J. Kreke, L. J. Magid, J. C. Gee, *Langmuir* **1996**, *12*, 699–705.

[12] D. A. Stauffer, D. A. Dougherty, *Tetrahedron Lett.* **1988**, *29*, 6039–6042.

[13] L. Garel, B. Lozach, J. P. Dutasta, A. Collet, *J. Am. Chem. Soc.* **1993**, *115*, 11652–11653.

[14] A. Arduini, W. M. McGregor, D. Paganuzzi, A. Pochini, A. Secchi, F. Ugozzoli, R. Ungaro, *J. Chem. Soc. Perkin Trans. 2* **1996**, 839–846.

[15] R. Arnecke, V. Böhmer, R. Cacciapaglia, A. Dalla Cort, L. Mandolini, *Tetrahedron* **1997**, *53*, 4901–4908.

[16] S. Kubik, *J. Am. Chem. Soc.* **1999**, *121*, 5846–5855.

[17] S. Shirakawa, S. Liu, S. Kaneko, Y. Kumatabara, A. Fukuda, Y. Omagari, K. Maruoka, *Angew. Chem. Int. Ed.* **2015**, *54*, 15767–15770.

[18] Y. Kumatabara, S. Kaneko, S. Nakata, S. Shirakawa, K. Maruoka, *Chem. – An Asian J.* **2016**, *11*, 2126–2129.

[19] M. J. Frisch, G. W. Trucks, H. B. Schlegel, G. E. Scuseria, M. A. Robb, J. R. Cheeseman, G. Scalmani, V. Barone, B. Mennucci, G. A. Petersson, H. Nakatsuji, M. Caricato, X. Li, H. P. Hratchian, A. F. Izmaylov, J. Bloino, G. Zheng, J. L. Sonnenberg, M. Hada, M. Ehara, K. Toyota, R. Fukuda, J. Hasegawa, M. Ishida, T. Nakajima, Y. Honda, O. Kitao, H. Nakai, T. Vreven, J. A. Montgomery Jr., J. E. Peralta, F. Ogliaro, M. Bearpark, J. J. Heyd, E. Brothers, K. N. Kudin, V. N. Staroverov, R. Kobayashi, J. Normand, K. Raghavachari, A. Rendell, J. C. Burant, S. S. Iyengar, J. Tomasi, M. Cossi, N. Rega, J. M. Millam, M. Klene, J. E. Knox, J. B. Cross, V. Bakken, C. Adamo, J. Jaramillo, R. Gomperts, R. E. Stratmann, O. Yazyev, A. J. Austin, R. Cammi, C. Pomelli, J. W. Ochterski, R. L. Martin, K. Morokuma, V. G. Zakrzewski, G. A. Voth, P. Salvador, J. J. Dannenberg, S. Dapprich, A. D. Daniels, O. Farkas, J. B. Foresman, J. V Ortiz, J. Cioslowski, D. J. Fox, *Gaussian16 (Revision A.03)* **2016**.

[20] C. Y. Legault, *Univ. Sherbrooke* **2009**.

[21] S. Grimme, S. Ehrlich, L. Goerigk, *J. Comput. Chem.* **2011**, *32*, 1456–1465.

[22] P. C. Knipe, S. Thompson, A. D. Hamilton, *Chem. Commun.* **2016**, *52*, 6521–6524.

7 NMR Spectra

S1

^{1}H NMR

400 MHz

$CDCl_3$

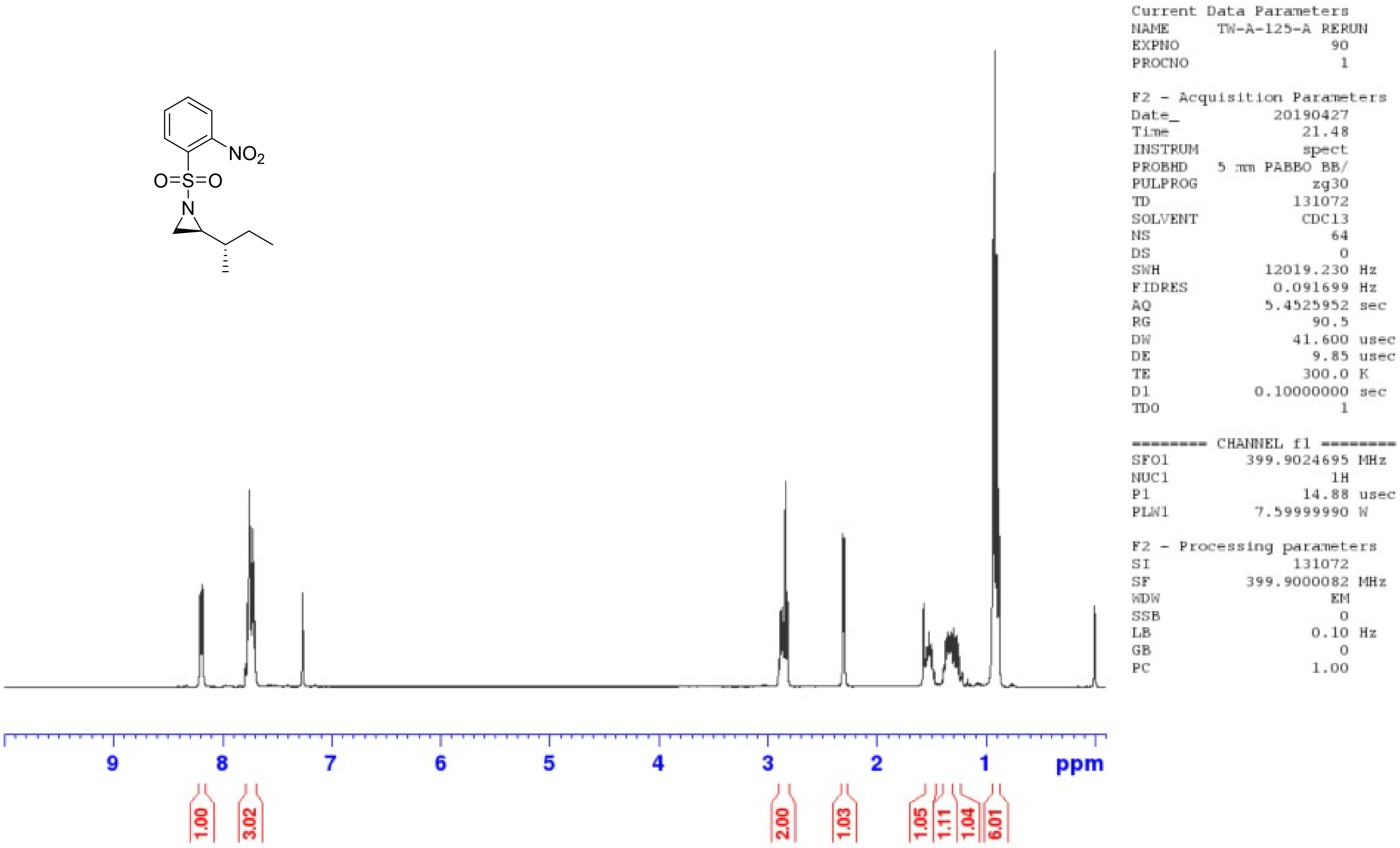

S1

^{13}C NMR

101 MHz

$CDCl_3$

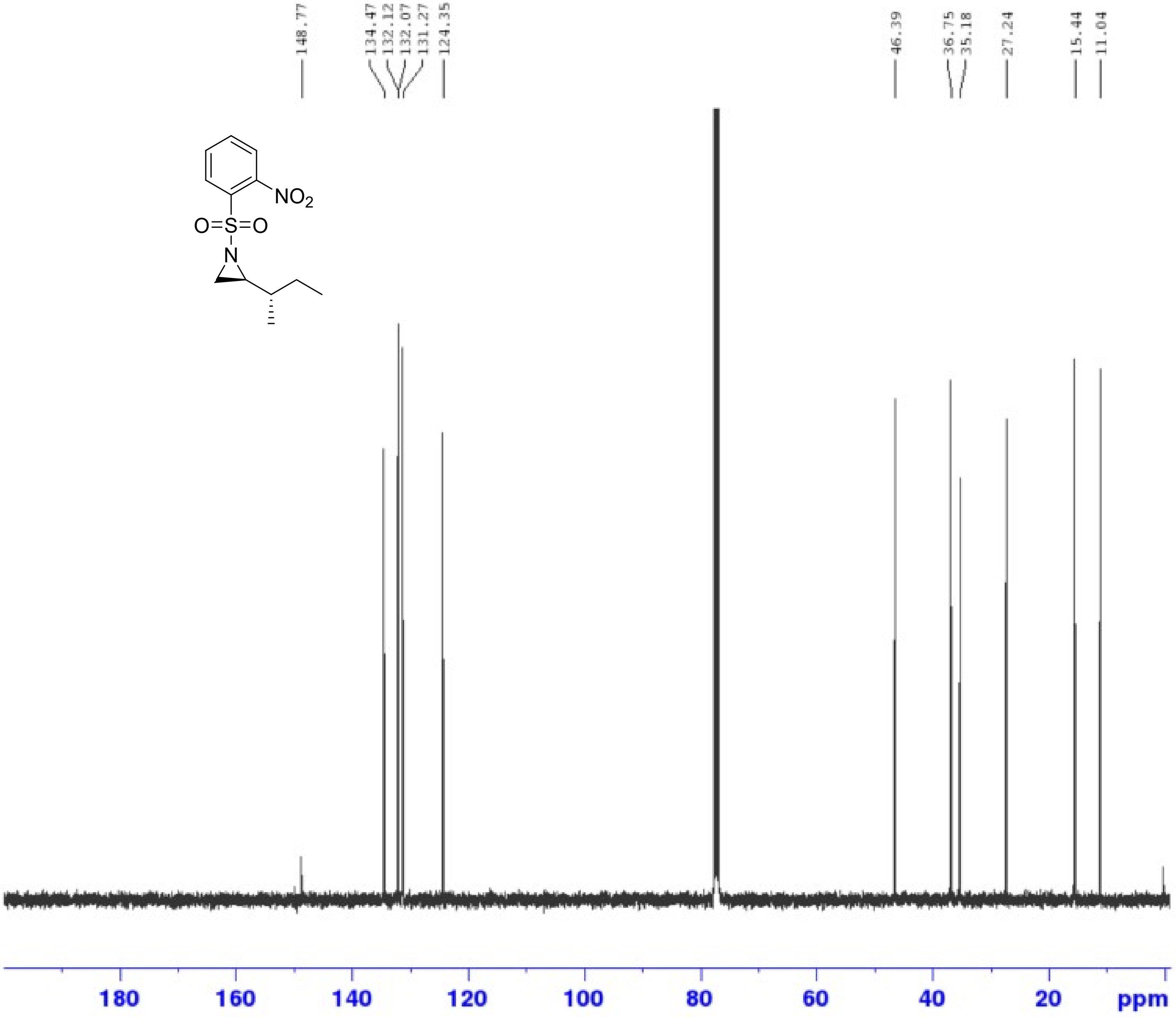


```
Current Data Parameters
NAME        TW-A-125-A RERUN
EXPNO                     92
PROCNO                     1

F2 - Acquisition Parameters
Date_               20190428
Time                    4.32
INSTRUM                spect
PROBHD      5 mm PABBO BB/
PULPROG              zgpg30
TD                     65536
SOLVENT                CDCl3
NS                      1024
DS                         4
SWH                24038.461 Hz
FIDRES              0.366798 Hz
AQ                 1.3631488 sec
RG                       181
DW                    20.800 usec
DE                      6.50 usec
TE                     300.0 K
D1                2.00000000 sec
D11               0.03000000 sec
TD0                        1

======== CHANNEL f1 ========
SFO1             100.5649900 MHz
NUC1                     13C
P1                     10.00 usec
PLW1             44.46300125 W

======== CHANNEL f2 ========
SFO2             399.9015996 MHz
NUC2                      1H
CPDPRG[2             waltz16
PCPD2                  90.00 usec
PLW2              7.59999990 W
PLW12             0.20774999 W
PLW13             0.16827001 W

F2 - Processing parameters
SI                     32768
SF               100.5549220 MHz
WDW                       EM
SSB                        0
LB                      1.00 Hz
GB                         0
PC                      1.40
```

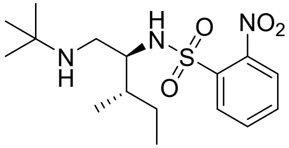

S2

^{1}H NMR

400 MHz

$CDCl_3$

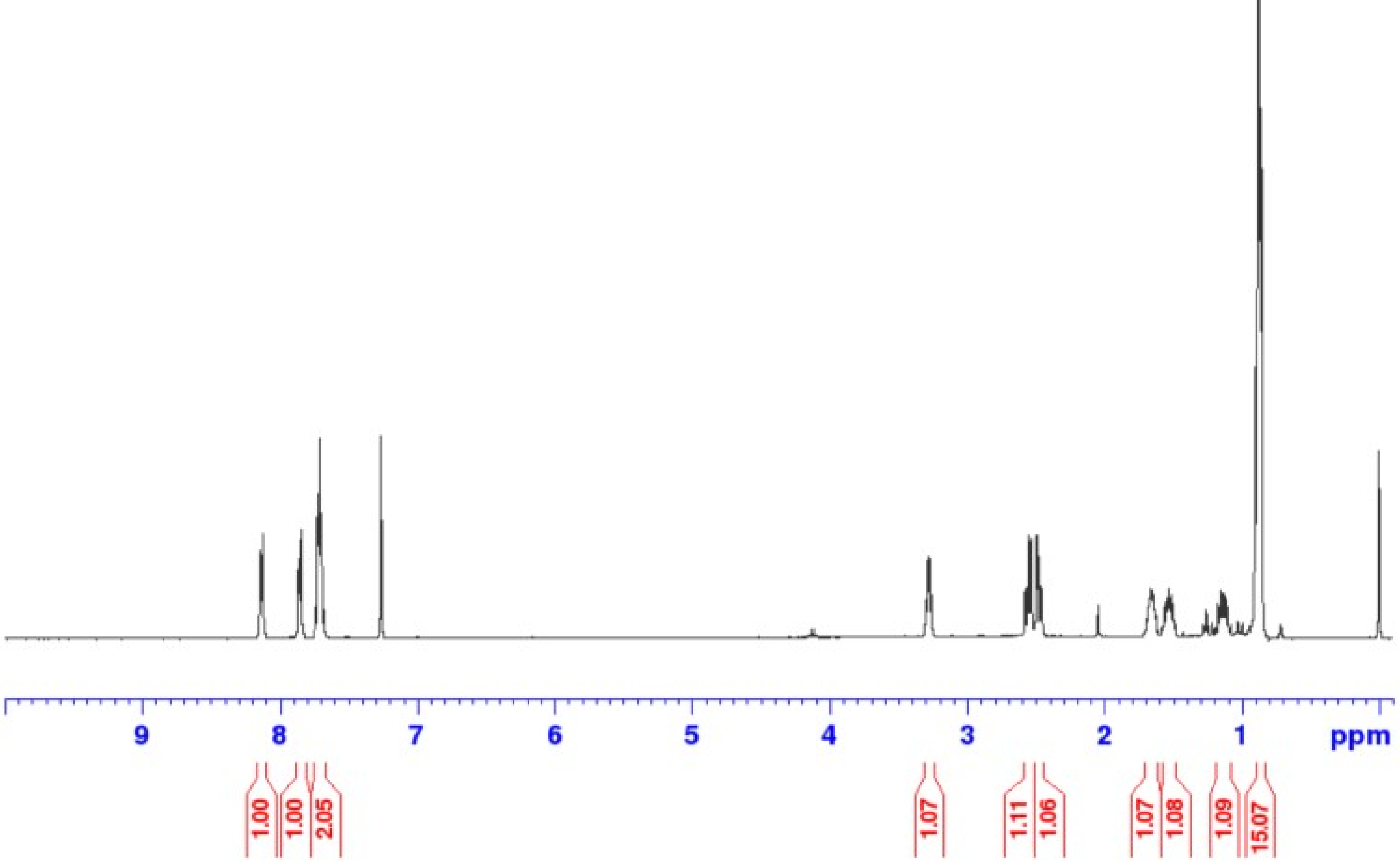


```
Current Data Parameters
NAME       TW-A-197-A RERUN
EXPNO                    10
PROCNO                    1

F2 - Acquisition Parameters
Date_              20190430
Time                  20.53
INSTRUM               spect
PROBHD     5 mm PABBO BB/
PULPROG                zg30
TD                   131072
SOLVENT               CDCl3
NS                       64
DS                        0
SWH            12019.230 Hz
FIDRES          0.091699 Hz
AQ            5.4525952 sec
RG                      101
DW               41.600 usec
DE                 9.85 usec
TE                  300.0 K
D1           0.10000000 sec
TD0                       1

======== CHANNEL f1 ========
SFO1         399.9024695 MHz
NUC1                     1H
P1                14.88 usec
PLW1            7.59999990 W

F2 - Processing parameters
SI                   131072
SF           399.9000085 MHz
WDW                      EM
SSB                       0
LB                  0.10 Hz
GB                        0
PC                     1.00
```

S2

^{13}C NMR

101 MHz

$CDCl_3$

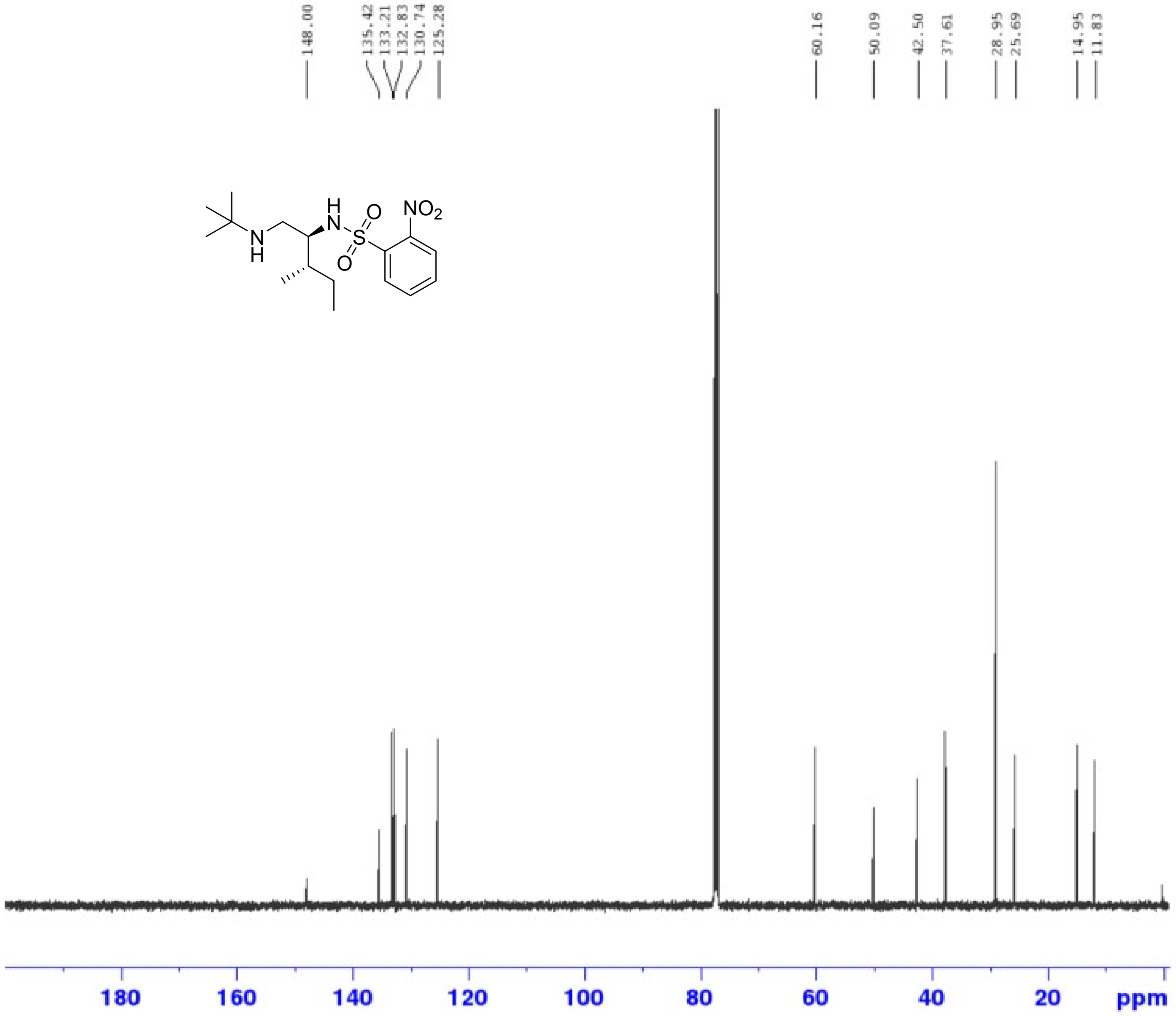


```
Current Data Parameters
NAME       TW-A-197-A RERUN
EXPNO                    12
PROCNO                    1

F2 - Acquisition Parameters
Date_             20190430
Time                 21.54
INSTRUM              spect
PROBHD     5 mm PABBO BB/
PULPROG             zgpg30
TD                   65536
SOLVENT              CDCl3
NS                    1024
DS                       4
SWH              24038.461 Hz
FIDRES            0.366798 Hz
AQ               1.3631488 sec
RG                     181
DW                  20.800 usec
DE                    6.50 usec
TE                   300.0 K
D1              2.00000000 sec
D11             0.03000000 sec
TD0                      1

======== CHANNEL f1 ========
SFO1           100.5649900 MHz
NUC1                   13C
P1                   10.00 usec
PLW1           44.46300125 W

======== CHANNEL f2 ========
SFO2           399.9015996 MHz
NUC2                    1H
CPDPRG[2           waltz16
PCPD2                90.00 usec
PLW2            7.59999990 W
PLW12           0.20774999 W
PLW13           0.16827001 W

F2 - Processing parameters
SI                   32768
SF             100.5549213 MHz
WDW                     EM
SSB                      0
LB                    1.00 Hz
GB                       0
PC                    1.40
```

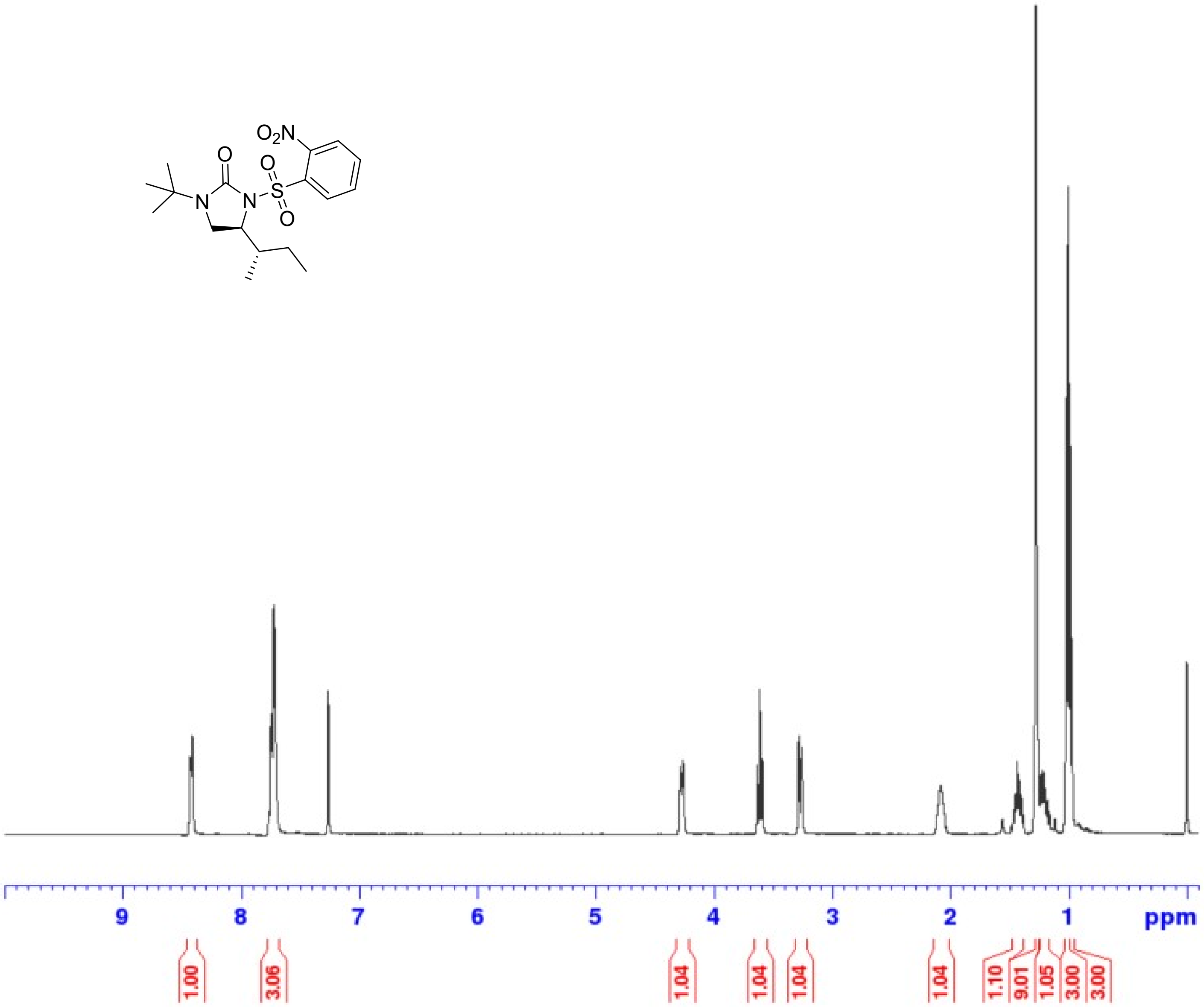

S3

^{1}H NMR

400 MHz

$CDCl_3$

Current Data Parameters
NAME TW-A-130-A
EXPNO 50
PROCNO 1

F2 - Acquisition Parameters
Date_ 20190427
Time 17.09
INSTRUM spect
PROBHD 5 mm PABBO BB/
PULPROG zg30
TD 131072
SOLVENT CDCl3
NS 64
DS 0
SWH 12019.230 Hz
FIDRES 0.091699 Hz
AQ 5.4525952 sec
RG 101
DW 41.600 usec
DE 9.85 usec
TE 300.0 K
D1 0.10000000 sec
TD0 1

======== CHANNEL f1 ========
SFO1 399.9024695 MHz
NUC1 1H
P1 14.88 usec
PLW1 7.59999990 W

F2 - Processing parameters
SI 131072
SF 399.9000095 MHz
WDW EM
SSB 0
LB 0.10 Hz
GB 0
PC 1.00

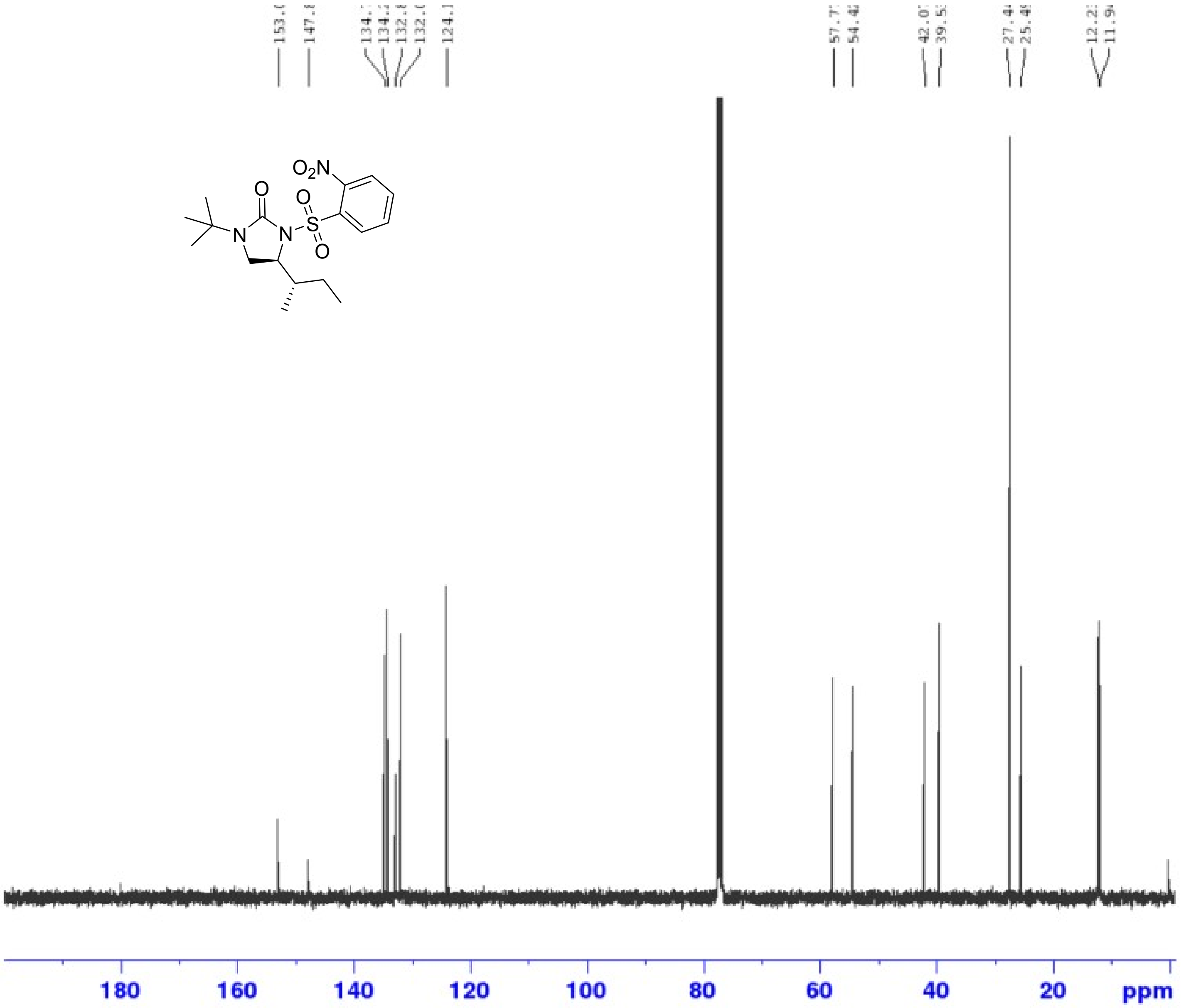

S3

^{13}C NMR

101 MHz

$CDCl_3$

```
Current Data Parameters
NAME          TW-A-130-A
EXPNO                 52
PROCNO                 1

F2 - Acquisition Parameters
Date_           20190428
Time               20.29
INSTRUM            spect
PROBHD   5 mm PABBO BB/
PULPROG          zgpg30
TD                 65536
SOLVENT            CDCl3
NS                  1024
DS                     4
SWH            24038.461 Hz
FIDRES          0.366798 Hz
AQ             1.3631488 sec
RG                   203
DW                20.800 usec
DE                  6.50 usec
TE                 300.0 K
D1            2.00000000 sec
D11           0.03000000 sec
TD0                    1

======== CHANNEL f1 ========
SFO1         100.5649900 MHz
NUC1                 13C
P1                 10.00 usec
PLW1         44.46300125 W

======== CHANNEL f2 ========
SFO2         399.9015996 MHz
NUC2                  1H
CPDPRG[2         waltz16
PCPD2              90.00 usec
PLW2          7.59999990 W
PLW12         0.20774999 W
PLW13         0.16827001 W

F2 - Processing parameters
SI                 32768
SF           100.5549213 MHz
WDW                   EM
SSB                    0
LB                  1.00 Hz
GB                     0
PC                  1.40
```

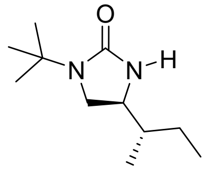

S4

^{1}H NMR

600 MHz

$CDCl_3$

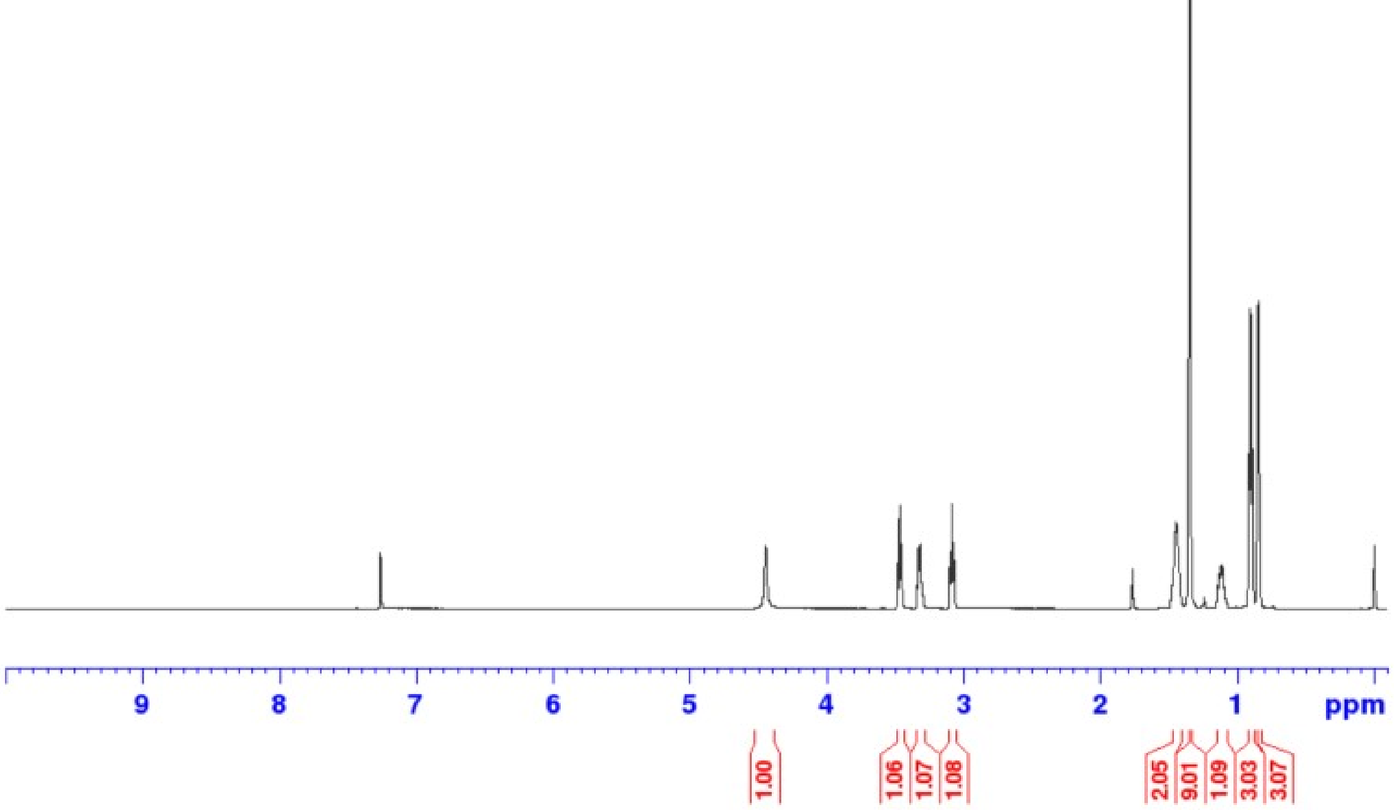


```
Current Data Parameters
NAME      TW-A-169-A 600
EXPNO                 80
PROCNO                 1

F2 - Acquisition Parameters
Date_           20190831
Time                9.15
INSTRUM            spect
PROBHD    5 mm PABBO BB/
PULPROG             zg30
TD                 65536
SOLVENT            CDCl3
NS                    16
DS                     2
SWH            12019.230 Hz
FIDRES          0.183399 Hz
AQ             2.7262976 sec
RG                 83.95
DW                41.600 usec
DE                  6.50 usec
TE                 300.0 K
D1            1.00000000 sec
TD0                    1

======== CHANNEL f1 ========
SFO1         600.1337060 MHz
NUC1                  1H
P1                 10.00 usec
PLW1         26.60000038 W

F2 - Processing parameters
SI                 65536
SF           600.1300145 MHz
WDW                   EM
SSB                    0
LB                  0.30 Hz
GB                     0
PC                  1.00
```

S4

^{13}C NMR

151 MHz

$CDCl_3$

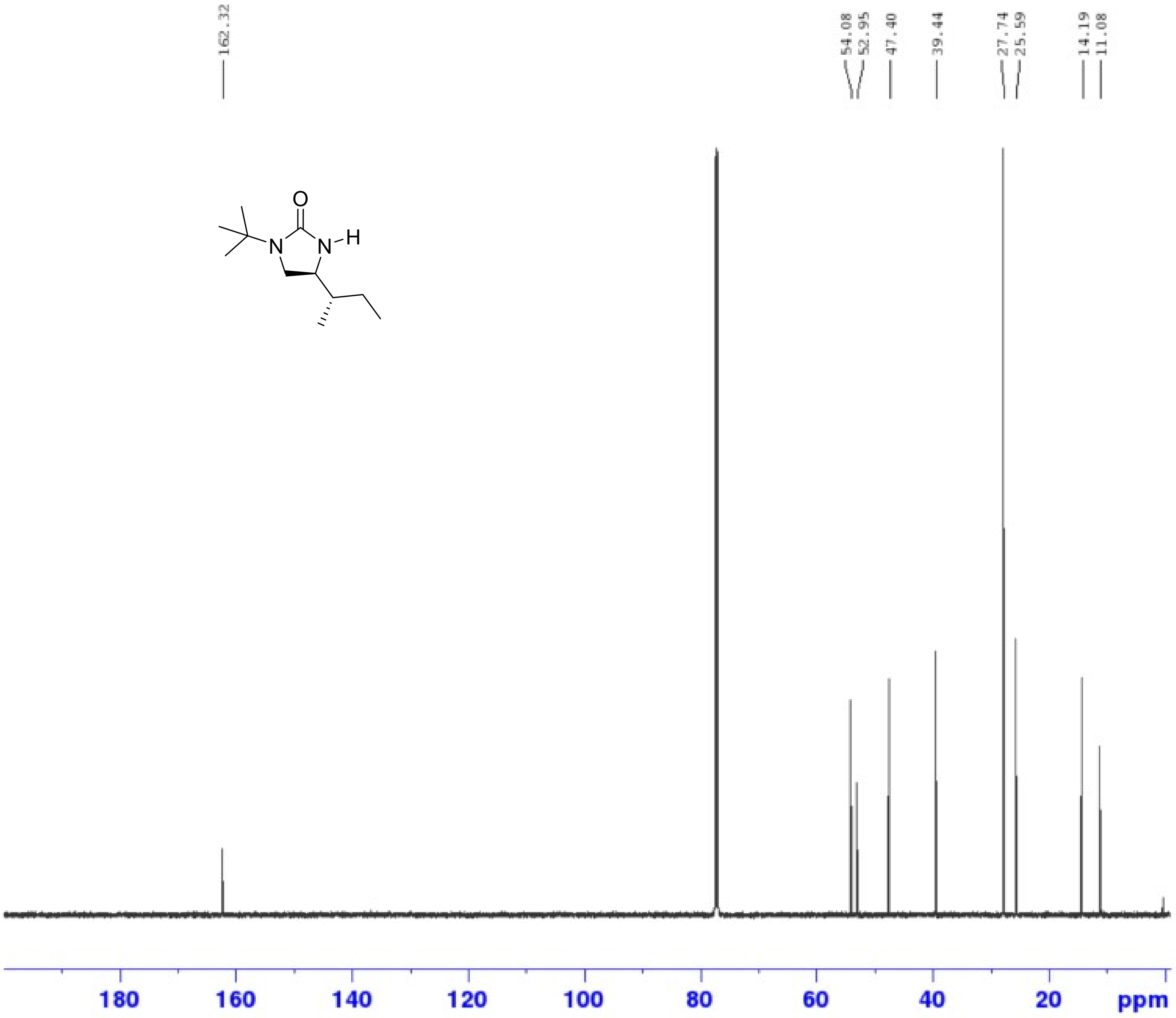


```
Current Data Parameters
NAME        TW-A-169-A 600
EXPNO                   82
PROCNO                   1

F2 - Acquisition Parameters
Date_             20190831
Time                 10.06
INSTRUM              spect
PROBHD     5 mm PABBO BB/
PULPROG             zgpg30
TD                   65536
SOLVENT              CDCl3
NS                    1024
DS                       4
SWH              36057.691 Hz
FIDRES            0.550197 Hz
AQ               0.9087659 sec
RG                  186.92
DW                  13.867 usec
DE                    6.50 usec
TE                   300.0 K
D1              2.00000000 sec
D11             0.03000000 sec
TD0                      1

======== CHANNEL f1 ========
SFO1           150.9178981 MHz
NUC1                   13C
P1                   11.80 usec
PLW1           85.00000000 W

======== CHANNEL f2 ========
SFO2           600.1324005 MHz
NUC2                    1H
CPDPRG[2           waltz16
PCPD2                80.00 usec
PLW2           27.00000000 W
PLW12           0.43891999 W
PLW13           0.28090999 W

F2 - Processing parameters
SI                   32768
SF             150.9027883 MHz
WDW                     EM
SSB                      0
LB                    1.00 Hz
GB                       0
PC                    1.40
```

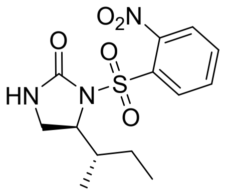

S5

^{1}H NMR

400 MHz

$CDCl_3$

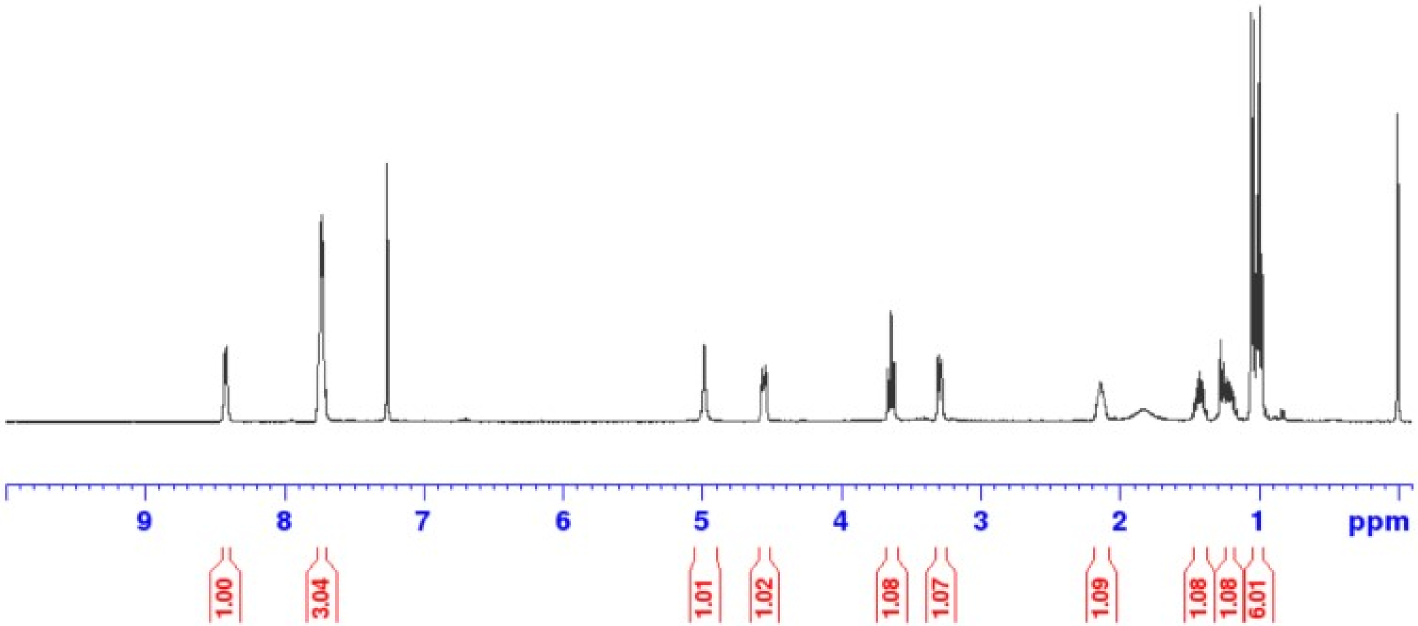


```
Current Data Parameters
NAME       TW-A-168-A COSY1
EXPNO                    10
PROCNO                    1

F2 - Acquisition Parameters
Date_              20190828
Time                  16.41
INSTRUM               spect
PROBHD     5 mm PABBO BB/
PULPROG                zg30
TD                   131072
SOLVENT               CDCl3
NS                       16
DS                        0
SWH             12019.230 Hz
FIDRES           0.091699 Hz
AQ              5.4525952 sec
RG                      203
DW                 41.600 usec
DE                   9.85 usec
TE                  299.7 K
D1             0.10000000 sec
TD0                       1

======== CHANNEL f1 ========
SFO1          399.9024695 MHz
NUC1                     1H
P1                  14.88 usec
PLW1           7.59999990 W

F2 - Processing parameters
SI                   131072
SF            399.9000095 MHz
WDW                      EM
SSB                       0
LB                     0.10 Hz
GB                        0
PC                     1.00
```

S5

^{13}C NMR

151 MHz

$CDCl_3$

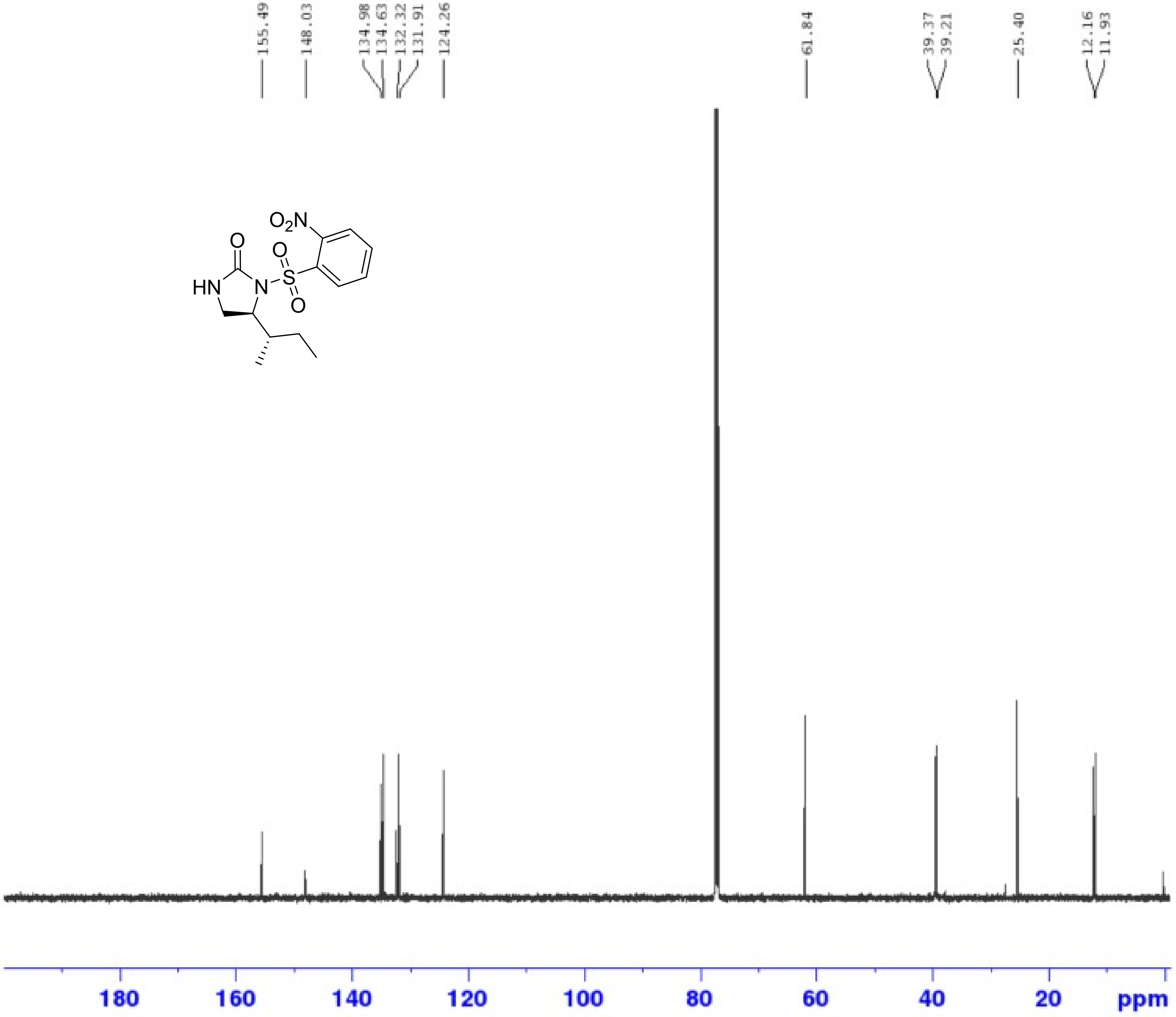

Current Data Parameters
NAME TW-A-168-A
EXPNO 11
PROCNO 1

F2 - Acquisition Parameters
Date_ 20190906
Time 11.59
INSTRUM spect
PROBHD 5 mm PABBO BB/
PULPROG zgpg30
TD 65536
SOLVENT CDCl3
NS 1024
DS 4
SWH 36057.691 Hz
FIDRES 0.550197 Hz
AQ 0.9087659 sec
RG 186.92
DW 13.867 usec
DE 6.50 usec
TE 300.0 K
D1 2.00000000 sec
D11 0.03000000 sec
TD0 1

======== CHANNEL f1 ========
SFO1 150.9178981 MHz
NUC1 13C
P1 11.80 usec
PLW1 85.00000000 W

======== CHANNEL f2 ========
SFO2 600.1324005 MHz
NUC2 1H
CPDPRG[2 waltz16
PCPD2 80.00 usec
PLW2 27.00000000 W
PLW12 0.43891999 W
PLW13 0.28090999 W

F2 - Processing parameters
SI 32768
SF 150.9028085 MHz
WDW EM
SSB 0
LB 1.00 Hz
GB 0
PC 1.40

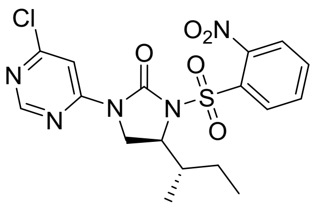

S6

^{1}H NMR

600 MHz

$CDCl_3$

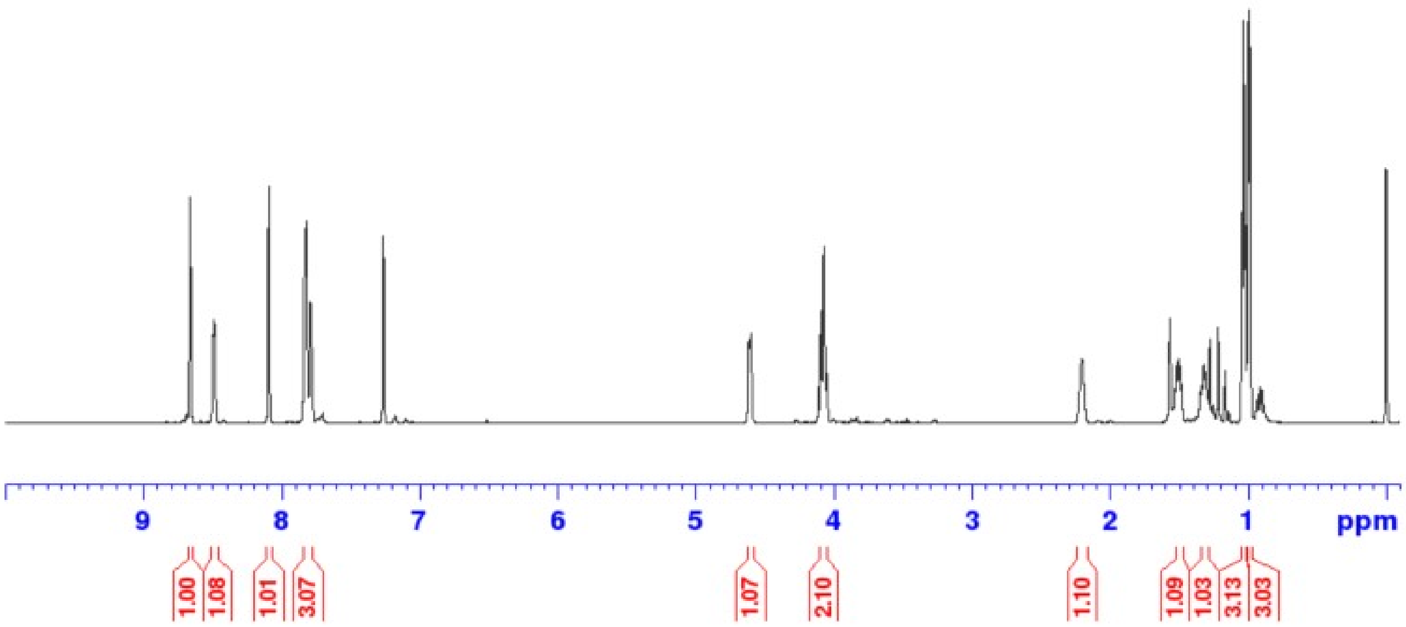

S6

^{13}C NMR

151 MHz

$CDCl_3$

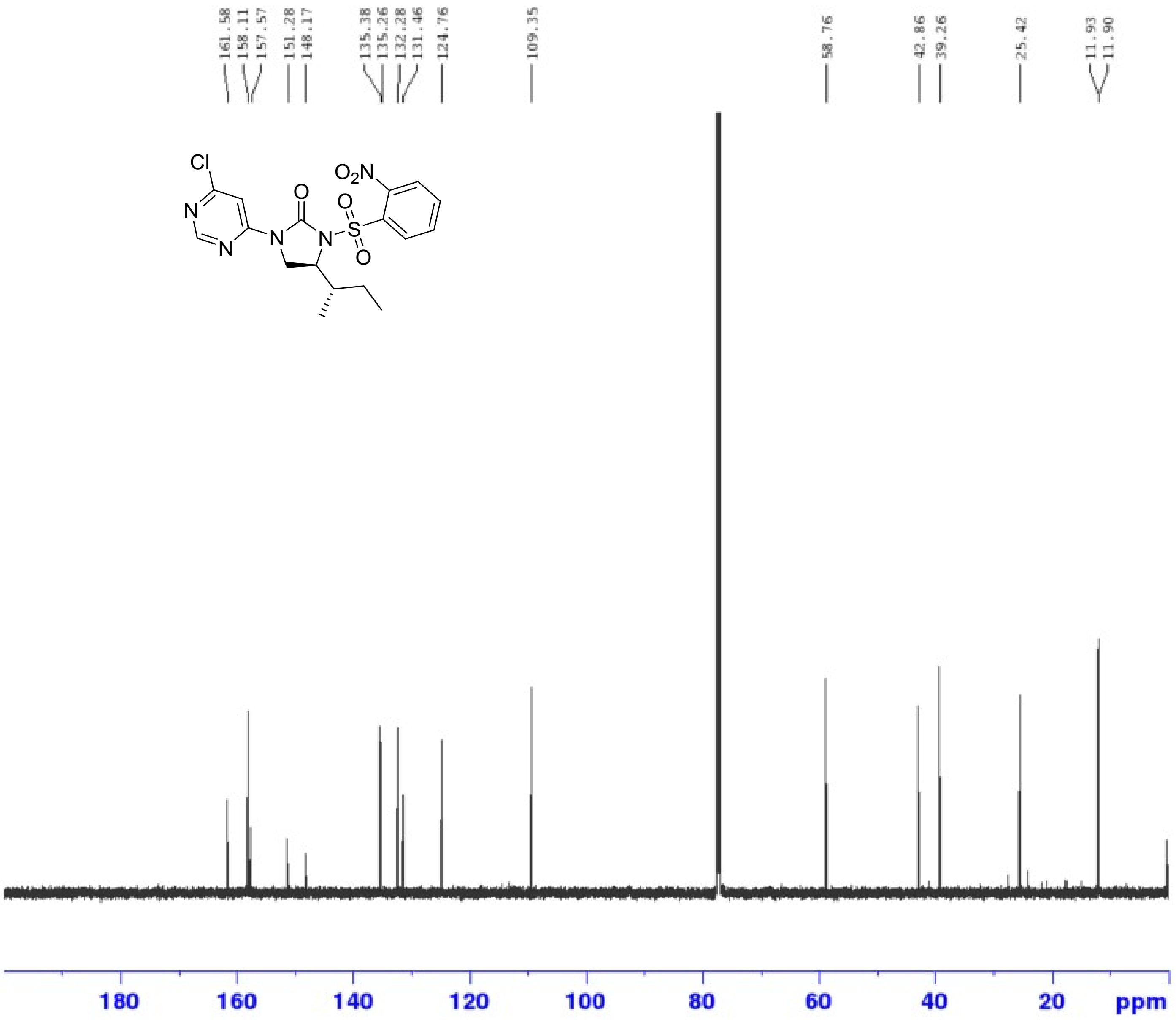

Current Data Parameters
NAME TW-B-215
EXPNO 11
PROCNO 1

F2 - Acquisition Parameters
Date_ 20190905
Time 19.57
INSTRUM spect
PROBHD 5 mm PABBO BB/
PULPROG zgpg30
TD 65536
SOLVENT CDCl3
NS 1024
DS 4
SWH 36057.691 Hz
FIDRES 0.550197 Hz
AQ 0.9087659 sec
RG 186.92
DW 13.867 usec
DE 6.50 usec
TE 300.0 K
D1 2.00000000 sec
D11 0.03000000 sec
TD0 1

======== CHANNEL f1 ========
SFO1 150.9178981 MHz
NUC1 13C
P1 11.80 usec
PLW1 85.00000000 W

======== CHANNEL f2 ========
SFO2 600.1324005 MHz
NUC2 1H
CPDPRG[2 waltz16
PCPD2 80.00 usec
PLW2 27.00000000 W
PLW12 0.43891999 W
PLW13 0.28090999 W

F2 - Processing parameters
SI 32768
SF 150.9027875 MHz
WDW EM
SSB 0
LB 1.00 Hz
GB 0
PC 1.40

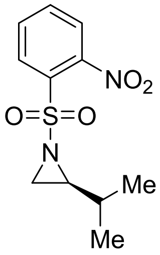

S7

^{1}H NMR

600 MHz

$CDCl_3$

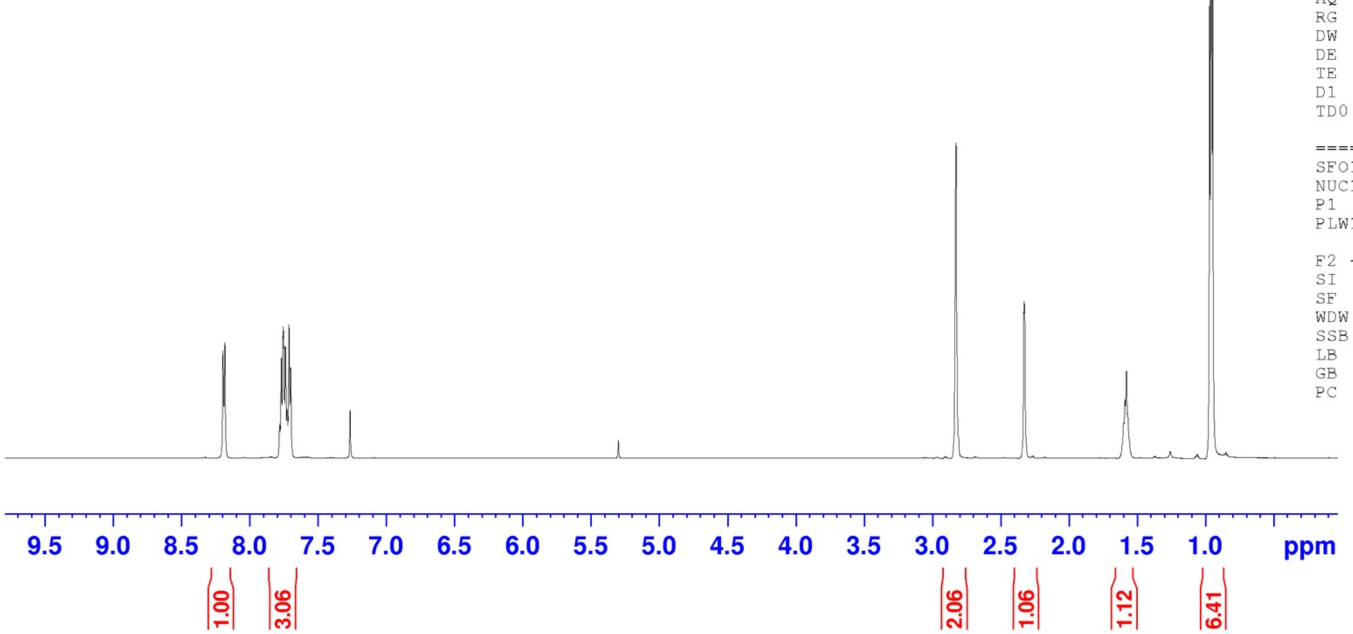


```
Current Data Parameters
NAME      TW-B-203-A 600
EXPNO               10
PROCNO               1

F2 - Acquisition Parameters
Date_         20190911
Time             19.04
INSTRUM          spect
PROBHD   5 mm PABBO BB/
PULPROG           zg30
TD               65536
SOLVENT          CDCl3
NS                  16
DS                   2
SWH          12019.230 Hz
FIDRES        0.183399 Hz
AQ           2.7262976 sec
RG               74.91
DW              41.600 usec
DE                6.50 usec
TE               300.0 K
D1          1.00000000 sec
TD0                  1

======== CHANNEL f1 ========
SFO1       600.1337060 MHz
NUC1                1H
P1               10.00 usec
PLW1       26.60000038 W

F2 - Processing parameters
SI               65536
SF         600.1300112 MHz
WDW                 EM
SSB                  0
LB                0.30 Hz
GB                   0
PC                1.00
```

S7

^{13}C NMR

151 MHz

$CDCl_3$

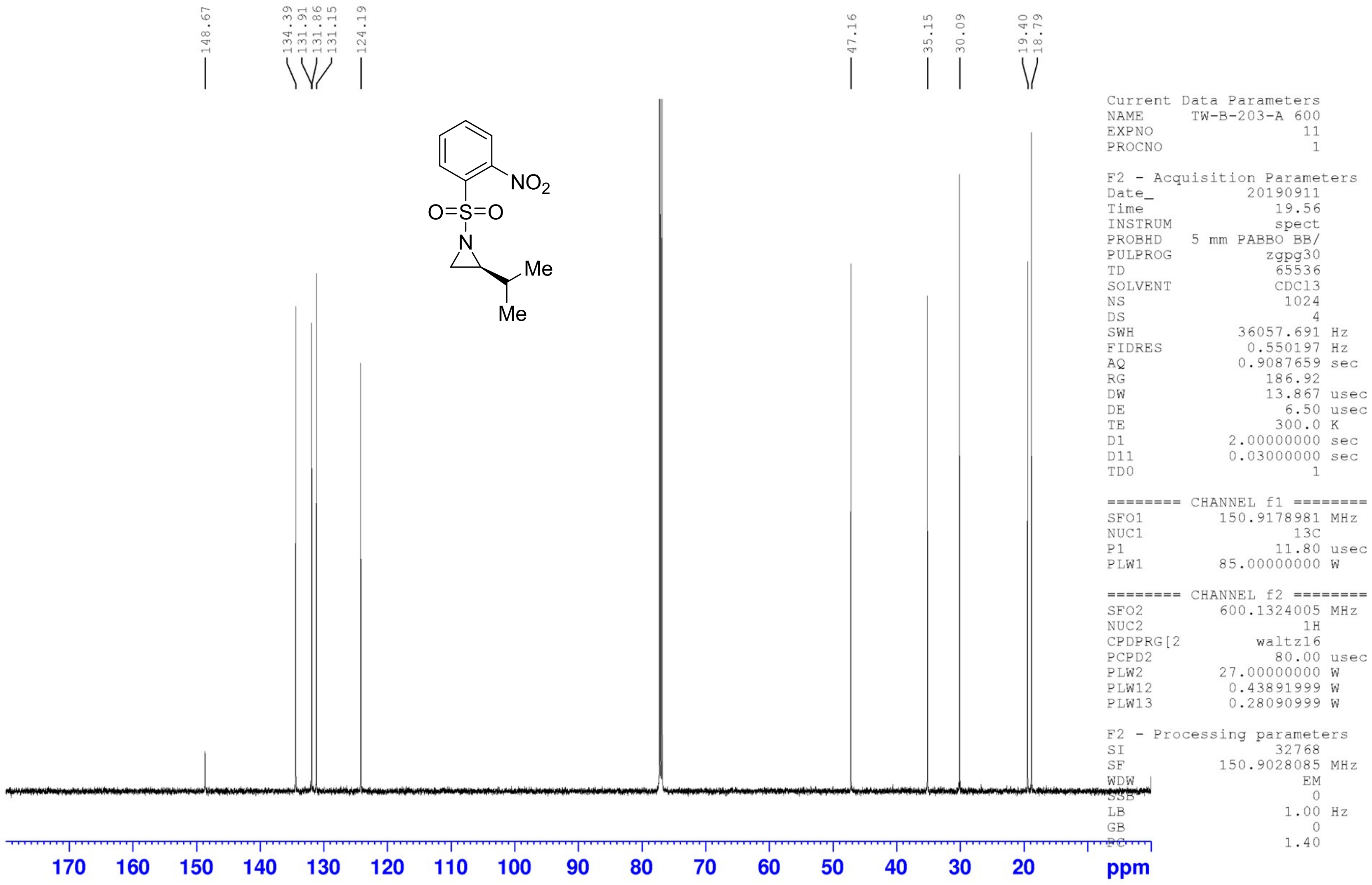

S8

^1H NMR

600 MHz

$CDCl_3$

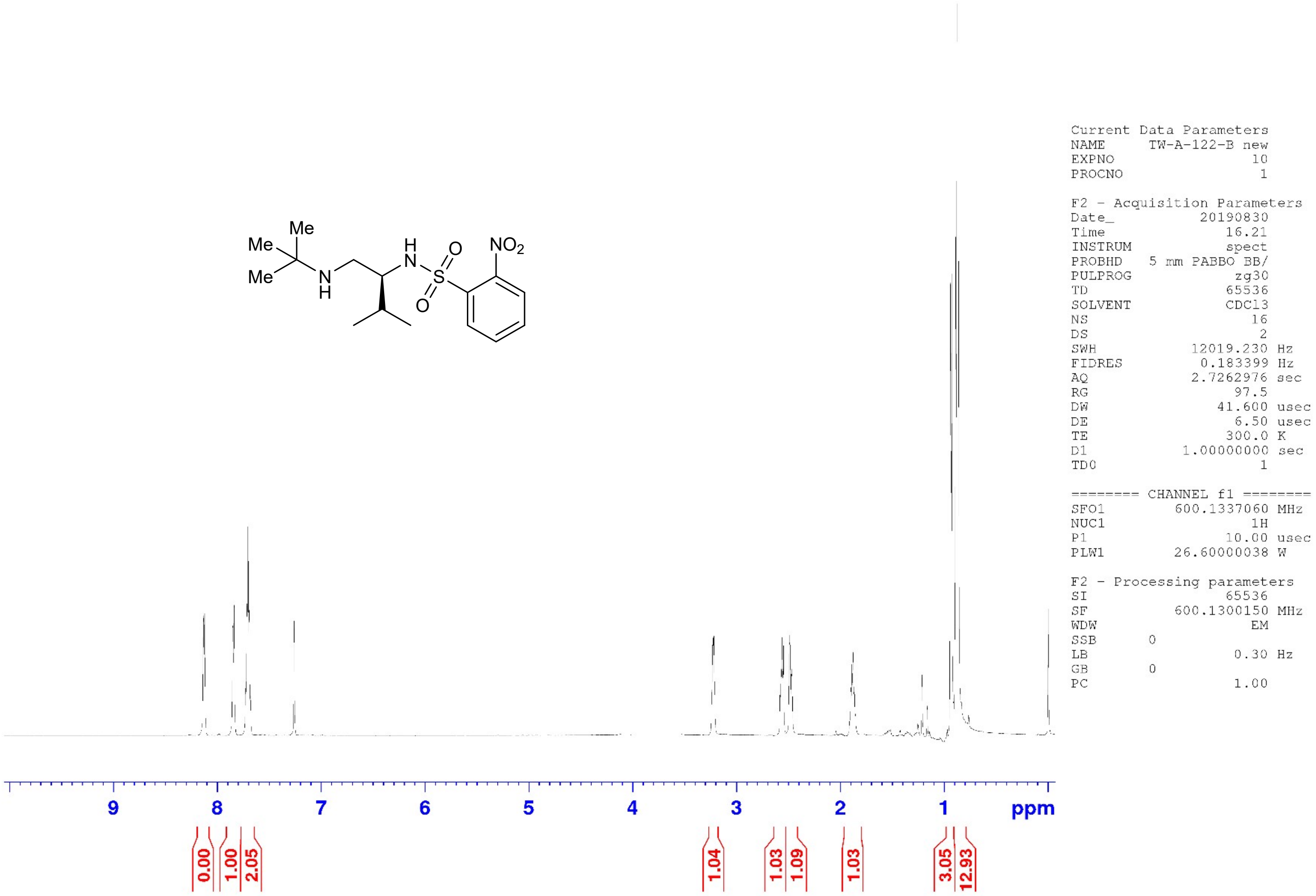

S8

^{13}C NMR

151 MHz

$CDCl_3$

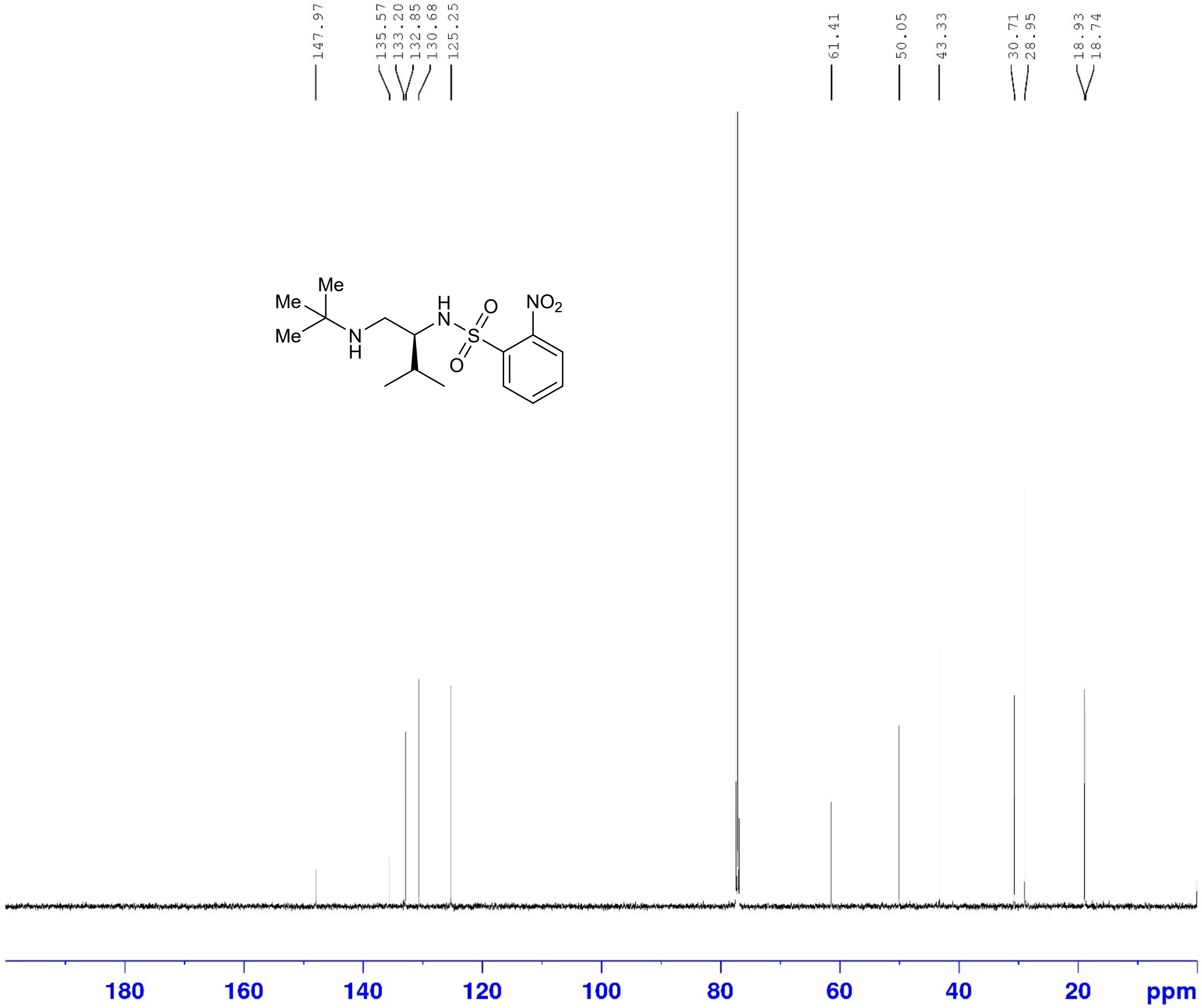


```
Current Data Parameters
NAME      TW-A-122-B new
EXPNO                 12
PROCNO                 1

F2 - Acquisition Parameters
Date_           20190830
Time               17.12
INSTRUM            spect
PROBHD   5 mm PABBO BB/
PULPROG          zgpg30
TD                 65536
SOLVENT            CDCl3
NS                  1024
DS                     4
SWH            36057.691 Hz
FIDRES          0.550197 Hz
AQ             0.9087659 sec
RG                186.92
DW                13.867 usec
DE                  6.50 usec
TE                 300.0 K
D1            2.00000000 sec
D11           0.03000000 sec
TD0                    1

======== CHANNEL f1 ========
SFO1         150.9178981 MHz
NUC1                 13C
P1                 11.80 usec
PLW1         85.00000000 W

======== CHANNEL f2 ========
SFO2         600.1324005 MHz
NUC2                  1H
CPDPRG[2         waltz16
PCPD2              80.00 usec
PLW2         27.00000000 W
PLW12         0.43891999 W
PLW13         0.28090999 W

F2 - Processing parameters
SI                 32768
SF           150.9027886 MHz
WDW                   EM
SSB      0
LB                  1.00 Hz
GB       0
PC                  1.40
```

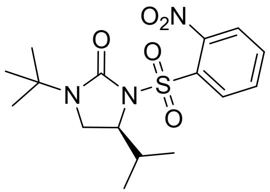

S9

^{1}H NMR

400 MHz

$CDCl_3$

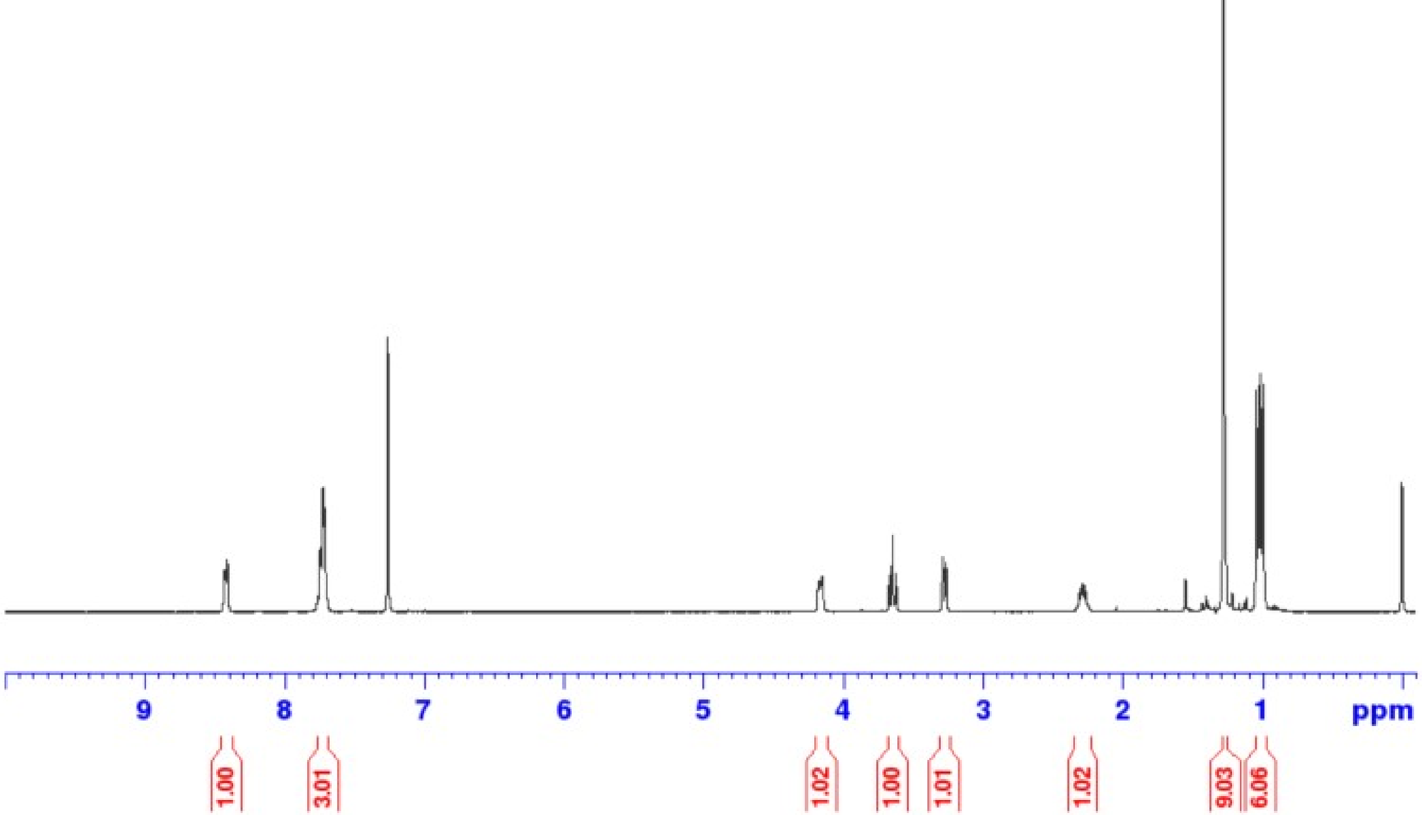


```
Current Data Parameters
NAME      TW-A-123-A RERUN
EXPNO                   20
PROCNO                   1

F2 - Acquisition Parameters
Date_             20190427
Time                 16.40
INSTRUM              spect
PROBHD    5 mm PABBO BB/
PULPROG               zg30
TD                  131072
SOLVENT              CDCl3
NS                      64
DS                       0
SWH              12019.230 Hz
FIDRES            0.091699 Hz
AQ               5.4525952 sec
RG                     203
DW                  41.600 usec
DE                    9.85 usec
TE                   300.0 K
D1              0.10000000 sec
TD0                      1

======== CHANNEL f1 ========
SFO1           399.9024695 MHz
NUC1                    1H
P1                   14.88 usec
PLW1            7.59999990 W

F2 - Processing parameters
SI                  131072
SF             399.9000100 MHz
WDW                     EM
SSB                      0
LB                    0.10 Hz
GB                       0
PC                    1.00
```

S9

^{13}C NMR

101 MHz

$CDCl_3$

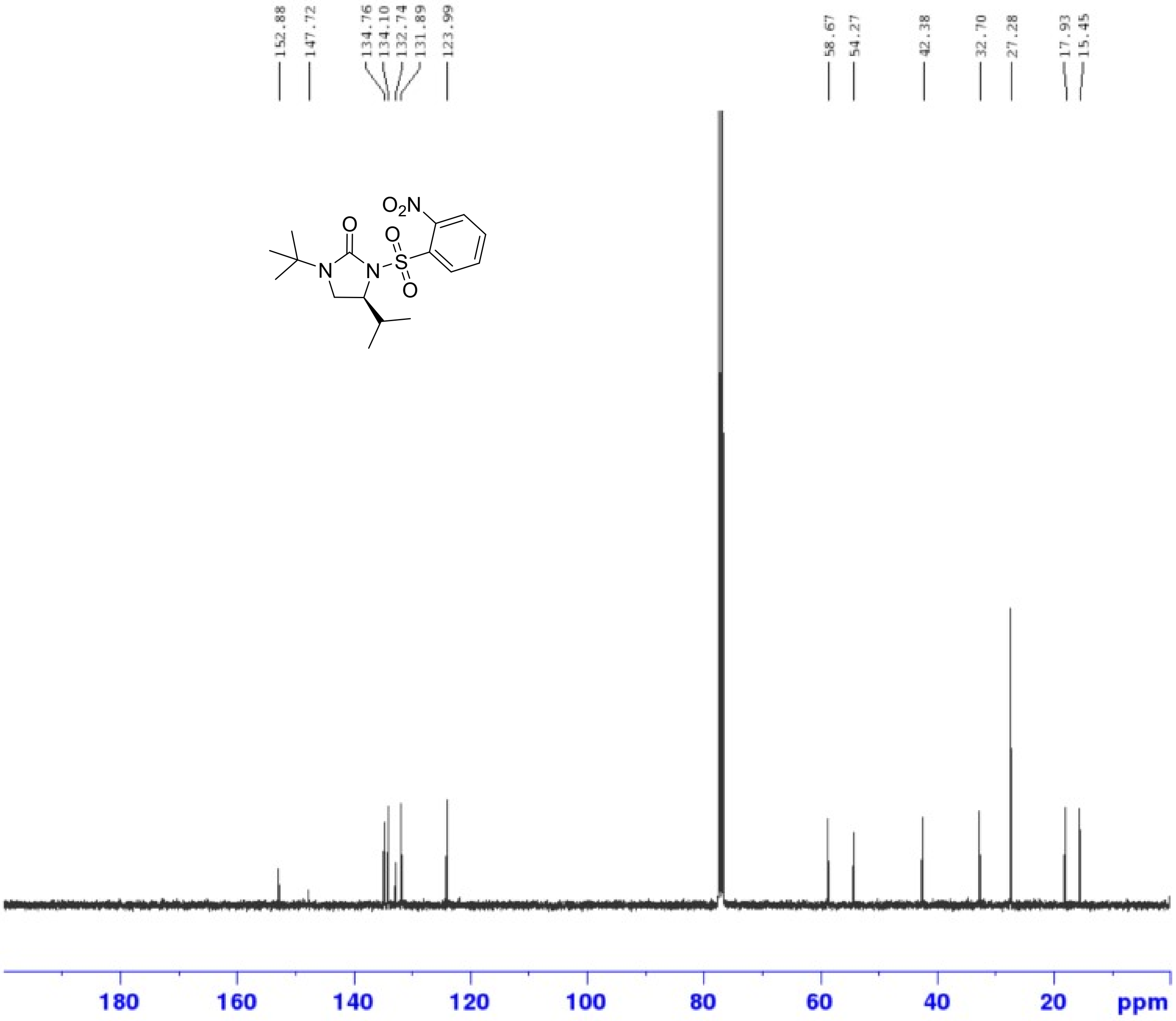


```
Current Data Parameters
NAME        TW-A-123-A RERUN
EXPNO                     23
PROCNO                     1

F2 - Acquisition Parameters
Date_               20190428
Time                   10.12
INSTRUM                spect
PROBHD     5 mm PABBO BB/
PULPROG               zgpg30
TD                     65536
SOLVENT                CDCl3
NS                      1024
DS                         4
SWH              24038.461 Hz
FIDRES            0.366798 Hz
AQ               1.3631488 sec
RG                       203
DW                    20.800 usec
DE                      6.50 usec
TE                     300.0 K
D1                2.00000000 sec
D11               0.03000000 sec
TD0                        1

======== CHANNEL f1 ========
SFO1          100.5649900 MHz
NUC1                     13C
P1                     10.00 usec
PLW1            44.46300125 W

======== CHANNEL f2 ========
SFO2          399.9015996 MHz
NUC2                      1H
CPDPRG[2             waltz16
PCPD2                  90.00 usec
PLW2              7.59999990 W
PLW12             0.20774999 W
PLW13             0.16827001 W

F2 - Processing parameters
SI                     32768
SF            100.5549350 MHz
WDW                       EM
SSB                        0
LB                      1.00 Hz
GB                         0
PC                      1.40
```

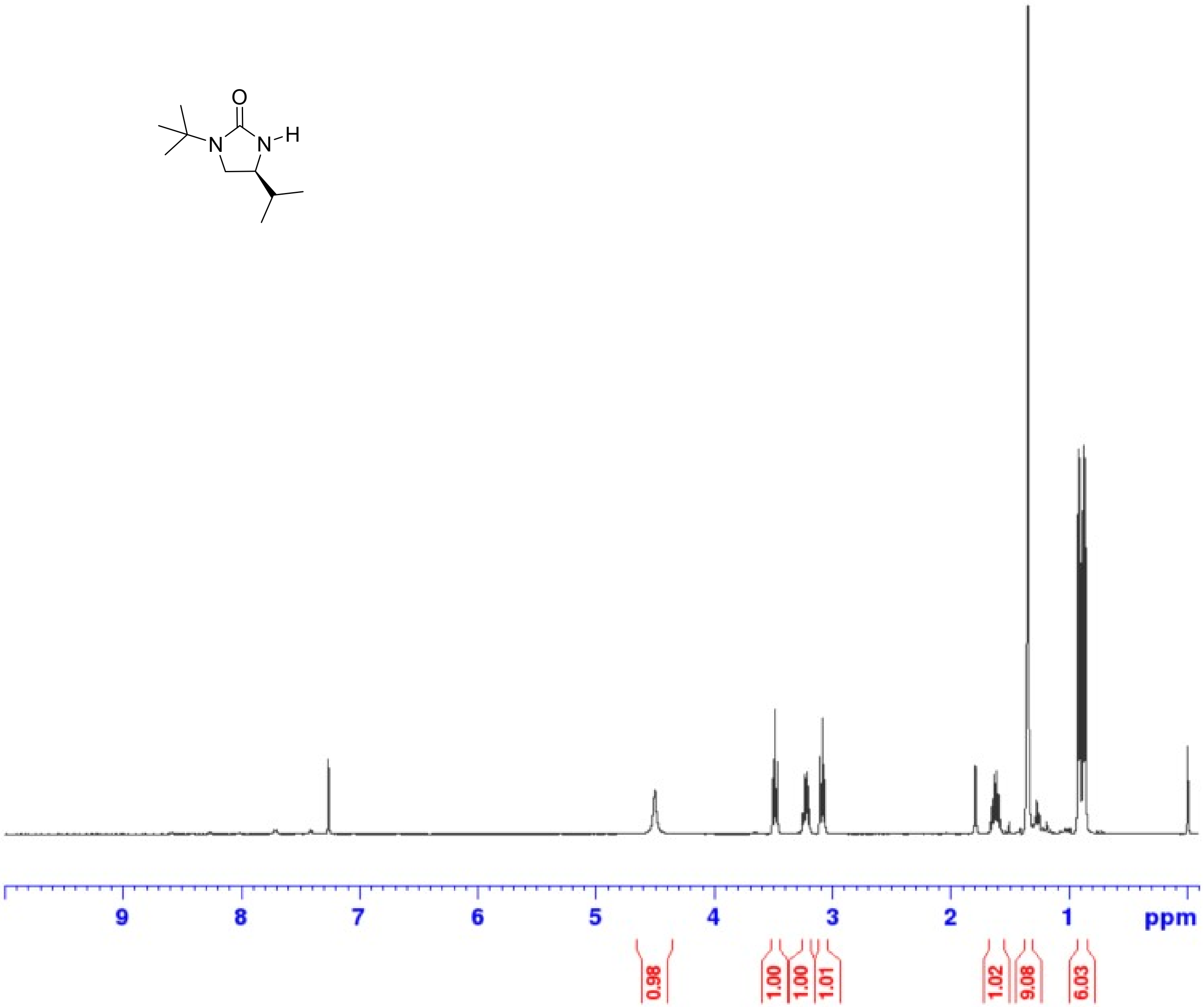

S10

^{1}H NMR

400 MHz

$CDCl_3$

```
Current Data Parameters
NAME      TW-A-194-A RERUN
EXPNO                   20
PROCNO                   1

F2 - Acquisition Parameters
Date_             20190501
Time                  0.02
INSTRUM              spect
PROBHD    5 mm PABBO BB/
PULPROG               zg30
TD                  131072
SOLVENT              CDCl3
NS                      64
DS                       0
SWH           12019.230 Hz
FIDRES         0.091699 Hz
AQ            5.4525952 sec
RG                    80.6
DW               41.600 usec
DE                 9.85 usec
TE                300.0 K
D1           0.10000000 sec
TD0                      1

======== CHANNEL f1 ========
SFO1        399.9024695 MHz
NUC1                    1H
P1                 14.88 usec
PLW1          7.59999990 W

F2 - Processing parameters
SI                  131072
SF          399.9000098 MHz
WDW                     EM
SSB                      0
LB                    0.10 Hz
GB                       0
PC                    1.00
```

S10

^{13}C NMR

101 MHz

$CDCl_3$

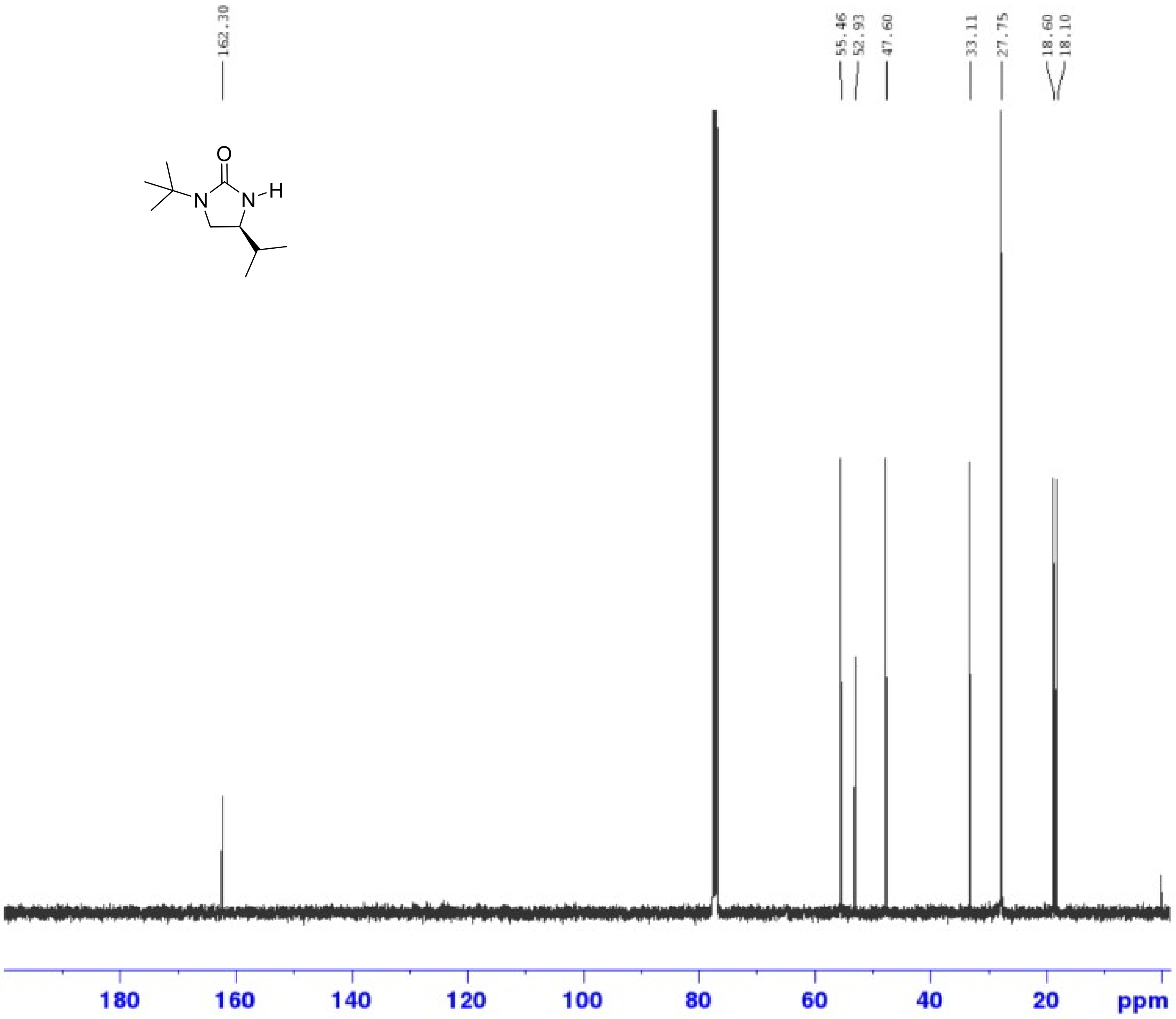


```
Current Data Parameters
NAME       TW-A-194-A RERUN
EXPNO                    22
PROCNO                    1

F2 - Acquisition Parameters
Date_              20190501
Time                   1.02
INSTRUM               spect
PROBHD     5 mm PABBO BB/
PULPROG             zgpg30
TD                   65536
SOLVENT              CDCl3
NS                    1024
DS                       4
SWH              24038.461 Hz
FIDRES            0.366798 Hz
AQ               1.3631488 sec
RG                     203
DW                  20.800 usec
DE                    6.50 usec
TE                   300.0 K
D1              2.00000000 sec
D11             0.03000000 sec
TD0                      1

======== CHANNEL f1 ========
SFO1           100.5649900 MHz
NUC1                   13C
P1                   10.00 usec
PLW1           44.46300125 W

======== CHANNEL f2 ========
SFO2           399.9015996 MHz
NUC2                    1H
CPDPRG[2           waltz16
PCPD2                90.00 usec
PLW2            7.59999990 W
PLW12           0.20774999 W
PLW13           0.16827001 W

F2 - Processing parameters
SI                   32768
SF             100.5549216 MHz
WDW                     EM
SSB                      0
LB                    1.00 Hz
GB                       0
PC                    1.40
```

S11

^{1}H NMR

400 MHz

$CDCl_3$

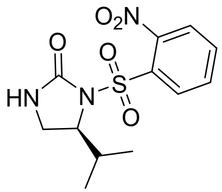

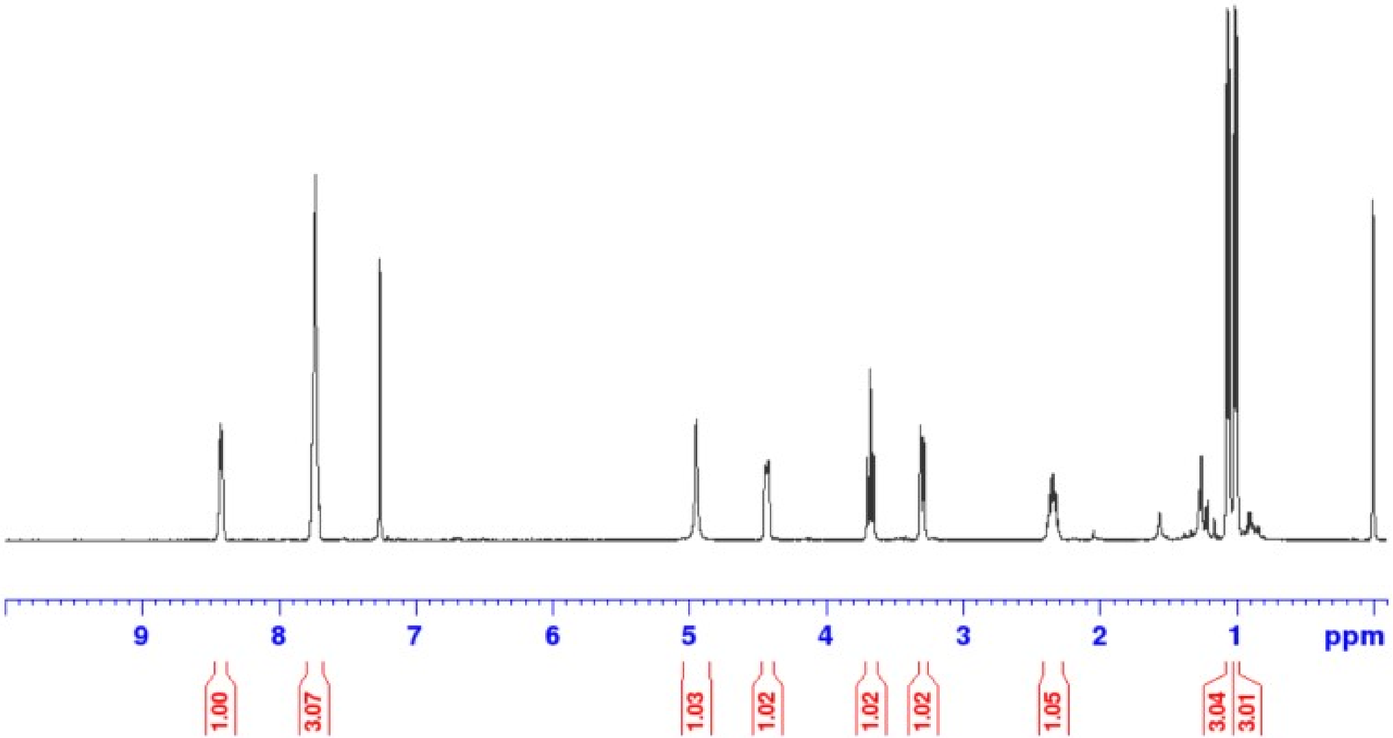


```
Current Data Parameters
NAME       TW-A-137-A RERUN
EXPNO                    10
PROCNO                    1

F2 - Acquisition Parameters
Date_              20190427
Time                  20.59
INSTRUM               spect
PROBHD    5 mm PABBO BB/
PULPROG                zg30
TD                   131072
SOLVENT               CDCl3
NS                       64
DS                        0
SWH              12019.230 Hz
FIDRES            0.091699 Hz
AQ               5.4525952 sec
RG                      203
DW                   41.600 usec
DE                     9.85 usec
TE                    300.0 K
D1               0.10000000 sec
TD0                       1

======== CHANNEL f1 ========
SFO1           399.9024695 MHz
NUC1                     1H
P1                    14.88 usec
PLW1             7.59999990 W

F2 - Processing parameters
SI                   131072
SF             399.9000099 MHz
WDW                      EM
SSB                       0
LB                     0.10 Hz
GB                        0
PC                     1.00
```

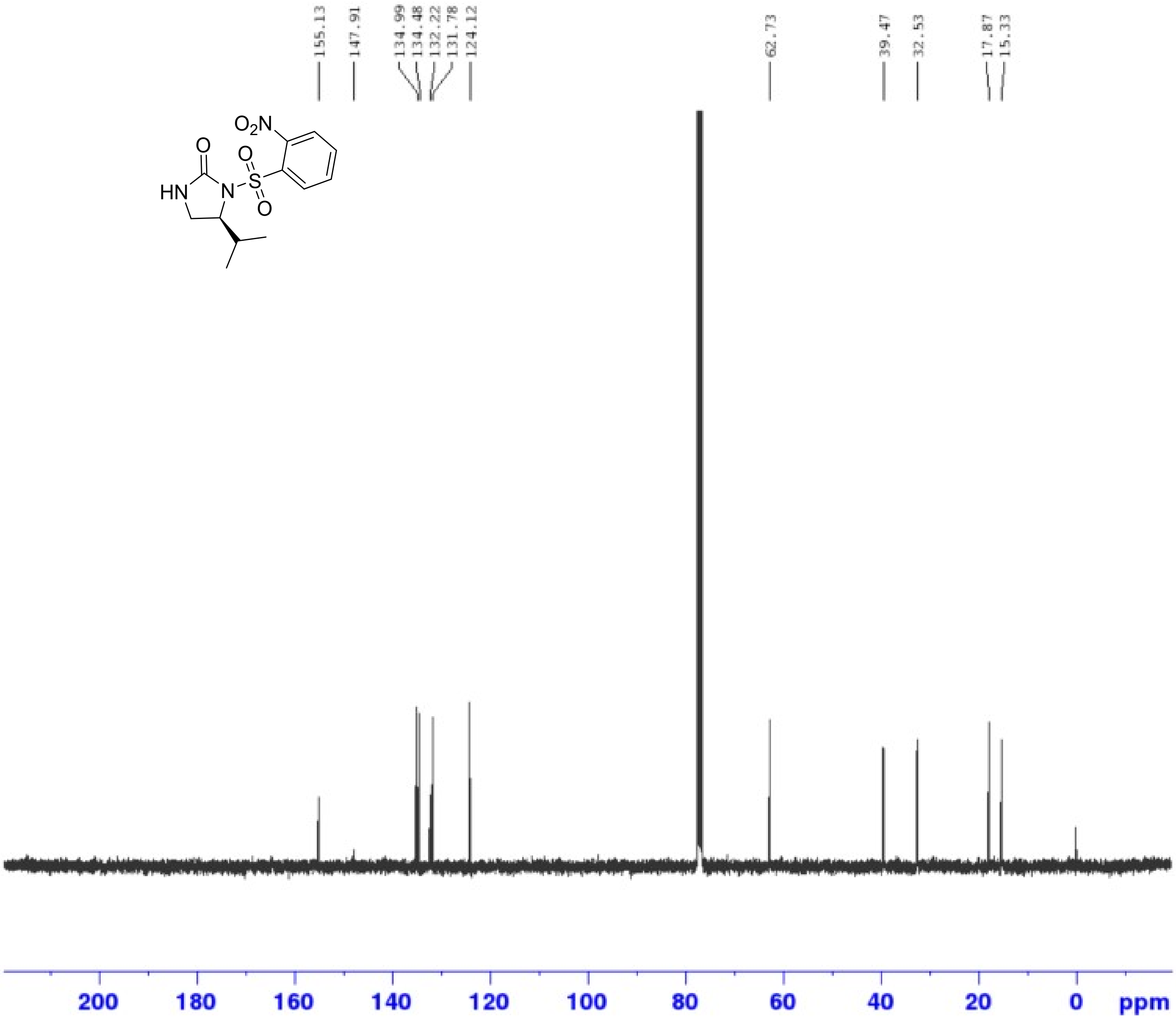

S11

^{13}C NMR

101 MHz

$CDCl_3$

```
Current Data Parameters
NAME        TW-A-137-A RERUN
EXPNO                     11
PROCNO                     1

F2 - Acquisition Parameters
Date_               20190429
Time                    2.09
INSTRUM                spect
PROBHD     5 mm PABBO BB/
PULPROG              zgpg30
TD                     65536
SOLVENT                CDC13
NS                      1024
DS                         4
SWH              24038.461 Hz
FIDRES            0.366798 Hz
AQ               1.3631488 sec
RG                       203
DW                    20.800 usec
DE                      6.50 usec
TE                     300.0 K
D1                2.00000000 sec
D11               0.03000000 sec
TD0                        1

======== CHANNEL f1 ========
SFO1          100.5649900 MHz
NUC1                     13C
P1                     10.00 usec
PLW1             44.46300125 W

======== CHANNEL f2 ========
SFO2          399.9015996 MHz
NUC2                      1H
CPDPRG[2             waltz16
PCPD2                  90.00 usec
PLW2              7.59999990 W
PLW12             0.20774999 W
PLW13             0.16827001 W

F2 - Processing parameters
SI                     32768
SF            100.5549350 MHz
WDW                       EM
SSB                        0
LB                      1.00 Hz
GB                         0
PC                      1.40
```

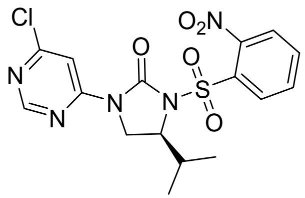

S12

^{1}H NMR

600 MHz

$CDCl_3$

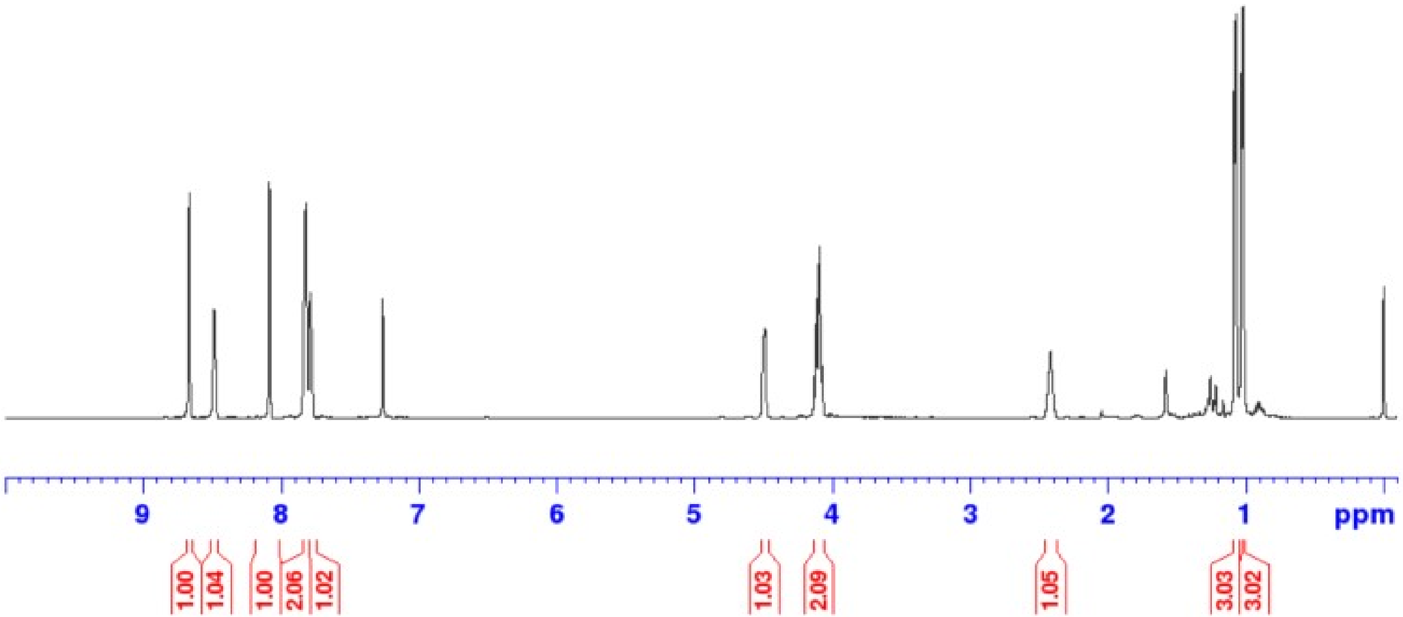

Current Data Parameters
NAME TW-B-196-A 600
EXPNO 27
PROCNO 1

F2 - Acquisition Parameters
Date_ 20190830
Time 18.57
INSTRUM spect
PROBHD 5 mm PABBO BB/
PULPROG zg30
TD 65536
SOLVENT CDCl3
NS 16
DS 2
SWH 12019.230 Hz
FIDRES 0.183399 Hz
AQ 2.7262976 sec
RG 134.29
DW 41.600 usec
DE 6.50 usec
TE 300.0 K
D1 1.00000000 sec
TD0 1

======== CHANNEL f1 ========
SFO1 600.1337060 MHz
NUC1 1H
P1 10.00 usec
PLW1 26.60000038 W

F2 - Processing parameters
SI 65536
SF 600.1300150 MHz
WDW EM
SSB 0
LB 0.30 Hz
GB 0
PC 1.00

S12

^{13}C NMR

151 MHz

$CDCl_3$

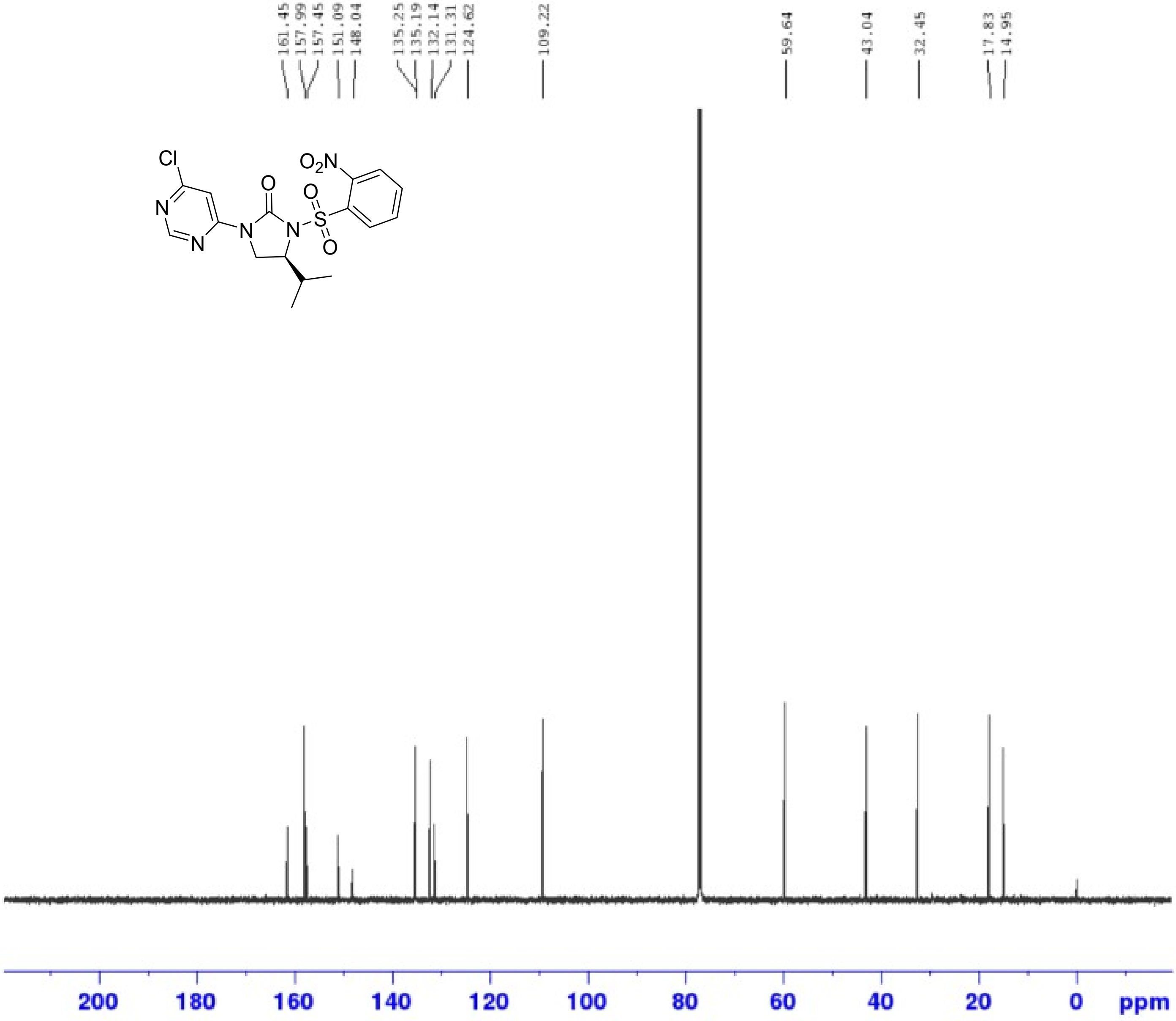


```
Current Data Parameters
NAME        TW-B-196-A 600
EXPNO                   28
PROCNO                   1

F2 - Acquisition Parameters
Date_             20190830
Time                 19.48
INSTRUM              spect
PROBHD    5 mm PABBO BB/
PULPROG            zgpg30
TD                  65536
SOLVENT             CDCl3
NS                   1024
DS                      4
SWH             36057.691 Hz
FIDRES           0.550197 Hz
AQ              0.9087659 sec
RG                 186.92
DW                 13.867 usec
DE                   6.50 usec
TE                  300.0 K
D1             2.00000000 sec
D11            0.03000000 sec
TD0                     1

======== CHANNEL f1 ========
SFO1         150.9178981 MHz
NUC1                 13C
P1                 11.80 usec
PLW1         85.00000000 W

======== CHANNEL f2 ========
SFO2         600.1324005 MHz
NUC2                  1H
CPDPRG[2         waltz16
PCPD2              80.00 usec
PLW2         27.00000000 W
PLW12         0.43891999 W
PLW13         0.28090999 W

F2 - Processing parameters
SI                  32768
SF            150.9028085 MHz
WDW                    EM
SSB                     0
LB                   1.00 Hz
GB                      0
PC                   1.40
```

S13

^1H NMR

400 MHz

$CDCl_3$

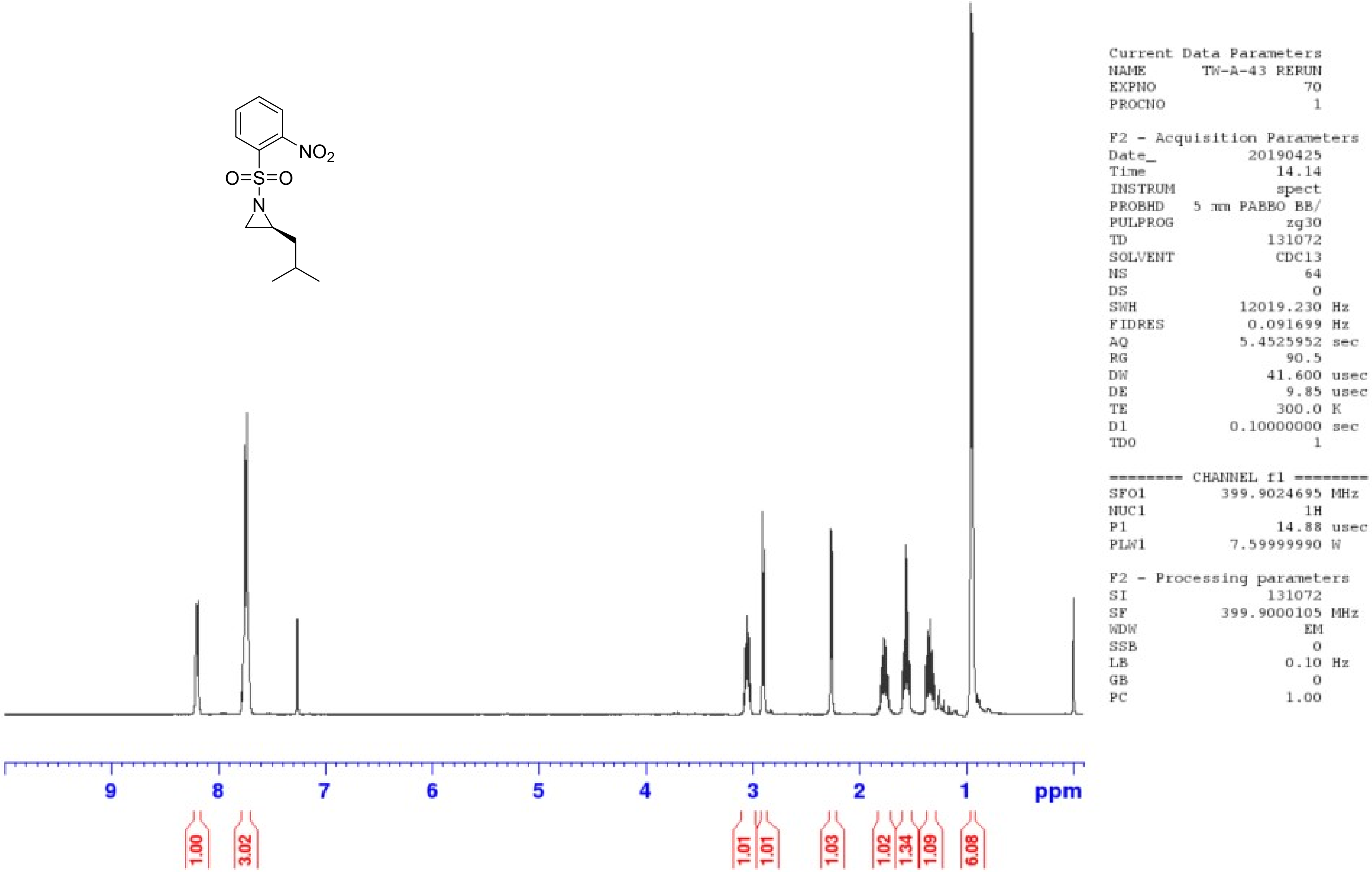

S13

^{13}C NMR

101 MHz

$CDCl_3$

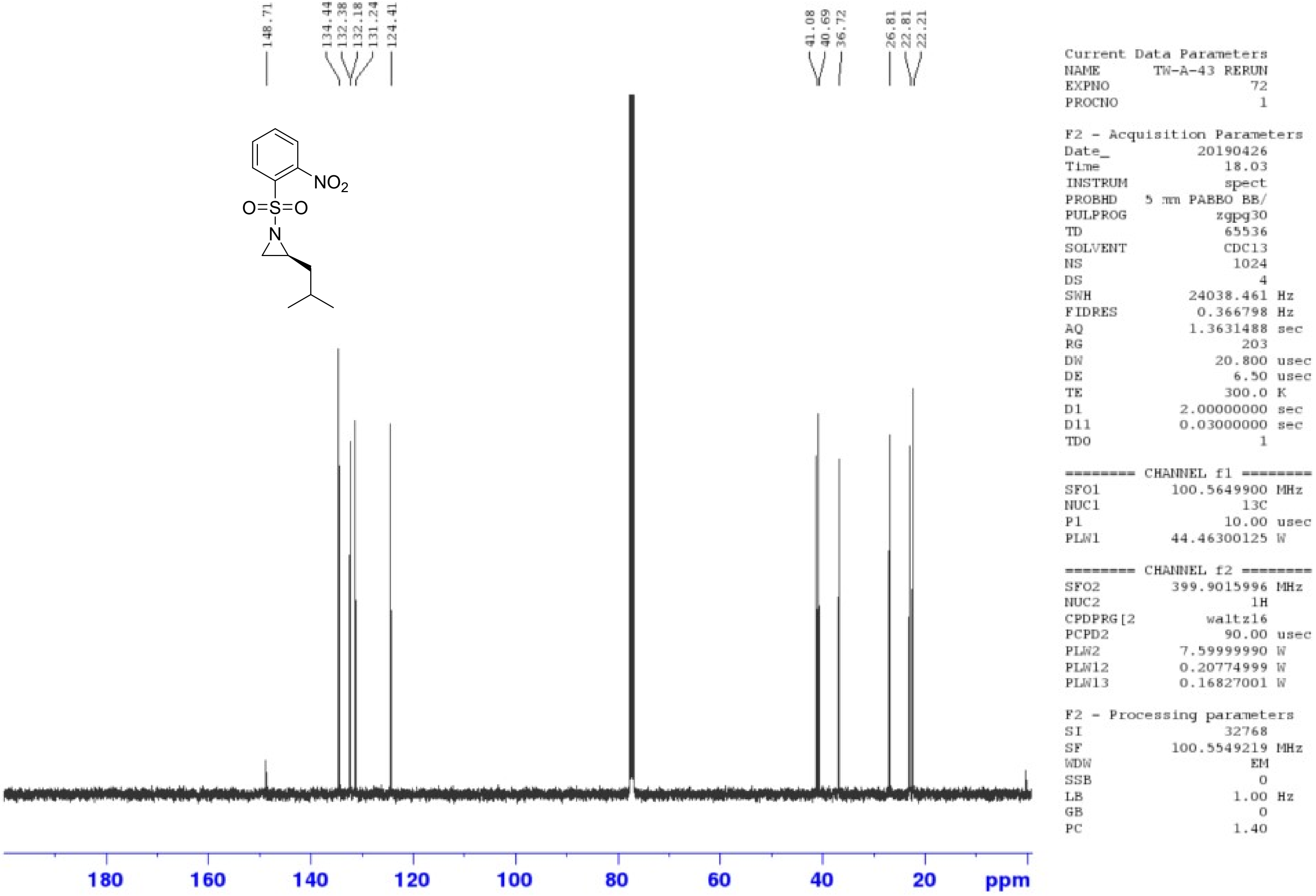

S14

^{1}H NMR

400 MHz

$CDCl_3$

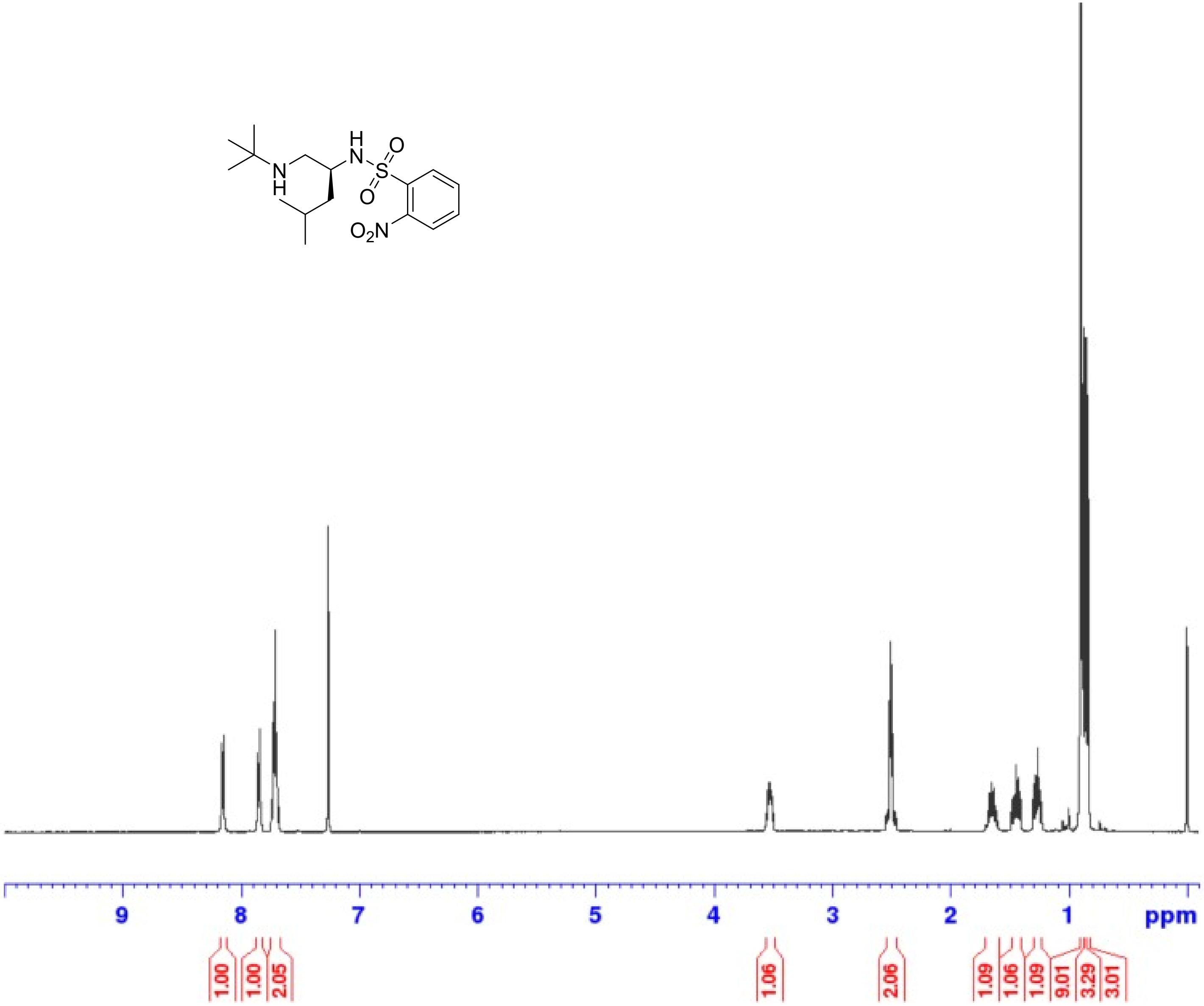


```
Current Data Parameters
NAME        TW-A-48 RERUN
EXPNO                  10
PROCNO                  1

F2 - Acquisition Parameters
Date_            20190425
Time                12.06
INSTRUM             spect
PROBHD     5 mm PABBO BB/
PULPROG              zg30
TD                 131072
SOLVENT             CDCl3
NS                     64
DS                      0
SWH             12019.230 Hz
FIDRES           0.091699 Hz
AQ              5.4525952 sec
RG                    203
DW                 41.600 usec
DE                   9.85 usec
TE                  300.0 K
D1             0.10000000 sec
TD0                     1

======== CHANNEL f1 ========
SFO1          399.9024695 MHz
NUC1                   1H
P1                  14.88 usec
PLW1           7.59999990 W

F2 - Processing parameters
SI                 131072
SF            399.9000097 MHz
WDW                    EM
SSB                     0
LB                   0.10 Hz
GB                      0
PC                   1.00
```

S14

^{13}C NMR

101 MHz

$CDCl_3$

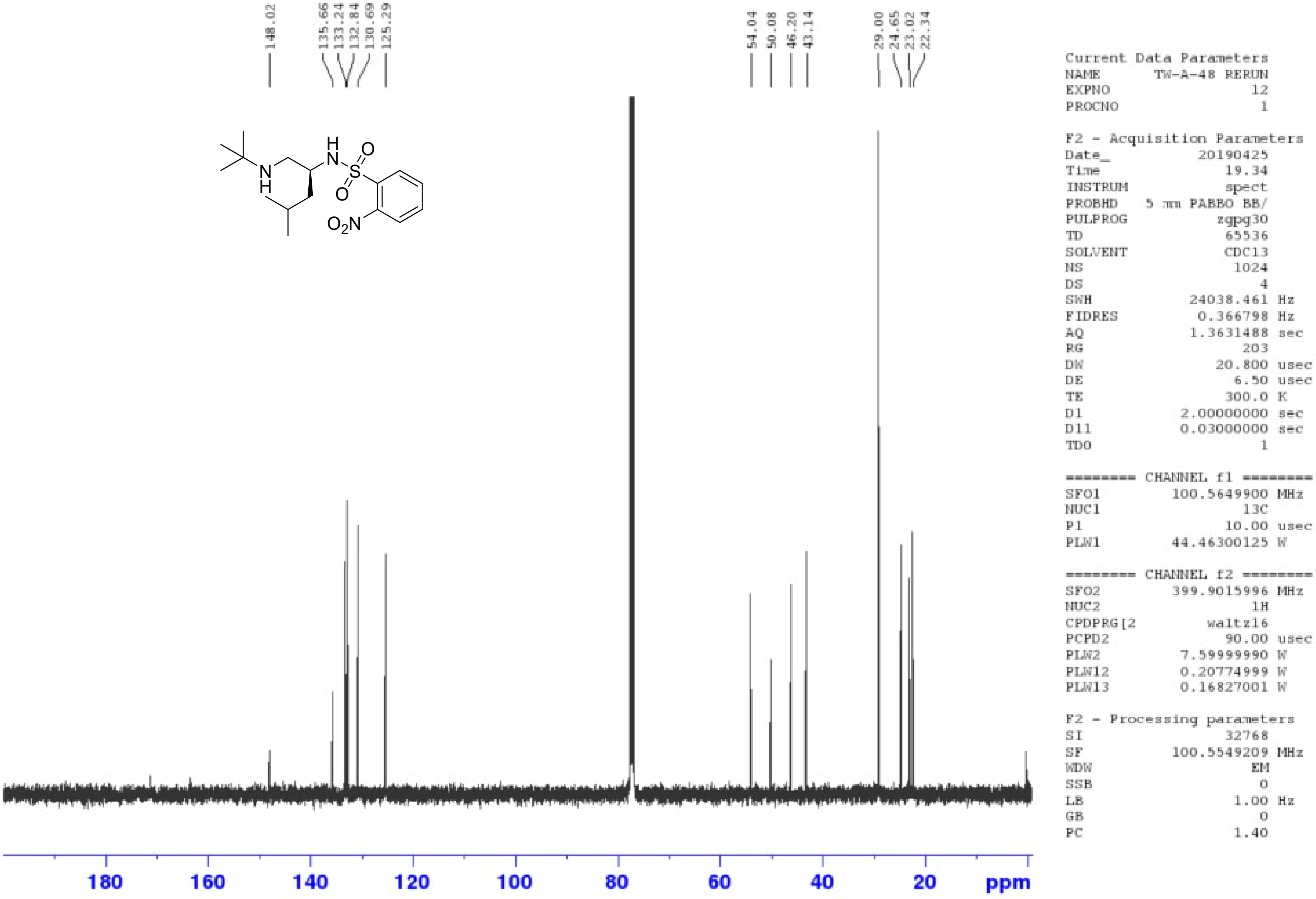

S15

^{1}H NMR

400 MHz

$CDCl_3$

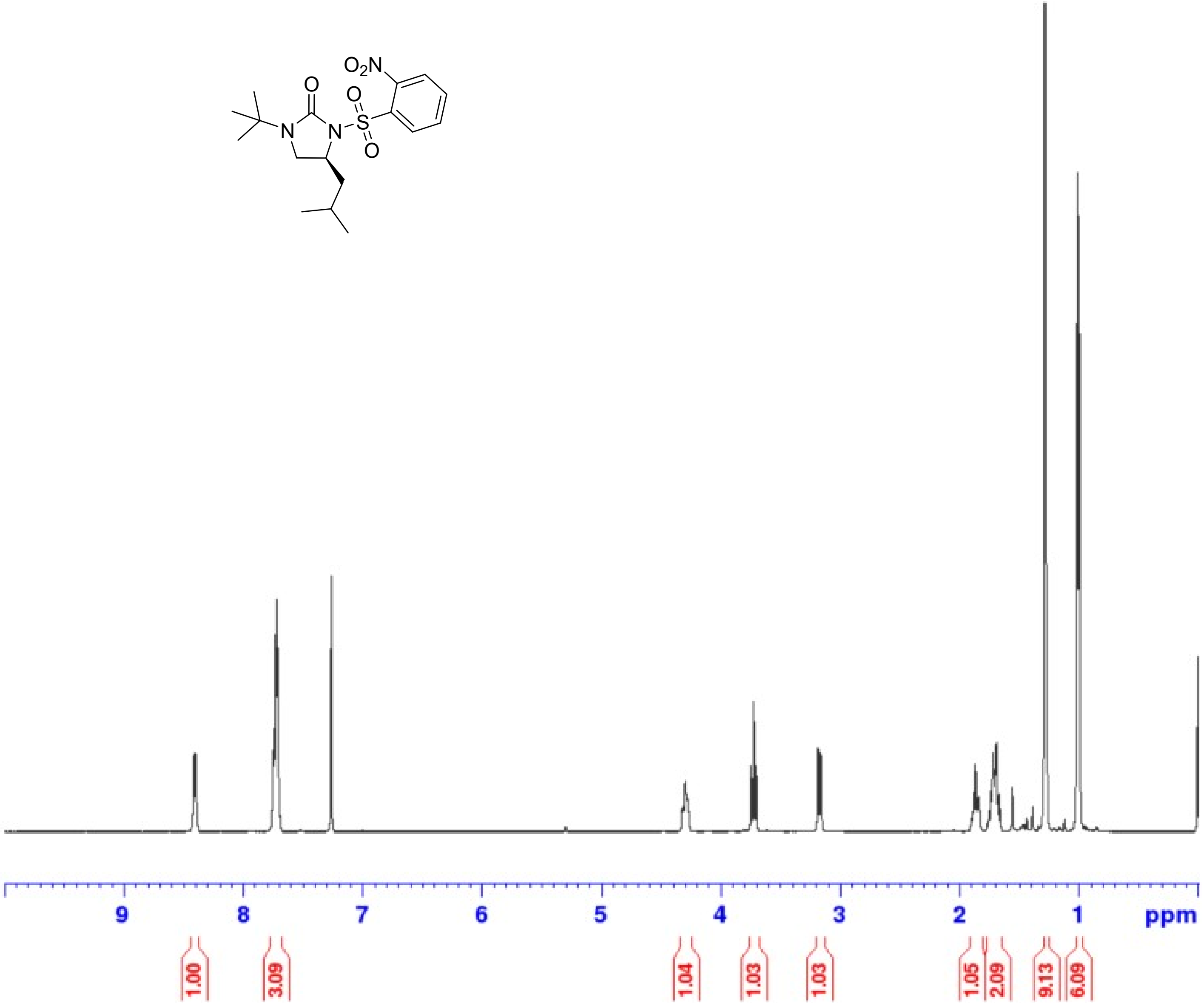


```
Current Data Parameters
NAME        TW-A-49-A RERUN
EXPNO                    20
PROCNO                    1

F2 - Acquisition Parameters
Date_              20190425
Time                  12.26
INSTRUM               spect
PROBHD     5 mm PABBO BB/
PULPROG                zg30
TD                   131072
SOLVENT               CDCl3
NS                       64
DS                        0
SWH            12019.230 Hz
FIDRES          0.091699 Hz
AQ             5.4525952 sec
RG                      203
DW               41.600 usec
DE                 9.85 usec
TE                  300.0 K
D1           0.10000000 sec
TD0                       1

======== CHANNEL f1 ========
SFO1         399.9024695 MHz
NUC1                     1H
P1                14.88 usec
PLW1          7.59999990 W

F2 - Processing parameters
SI                   131072
SF           399.9000099 MHz
WDW                      EM
SSB                       0
LB                  0.10 Hz
GB                        0
PC                     1.00
```

S15

^{13}C NMR

101 MHz

$CDCl_3$

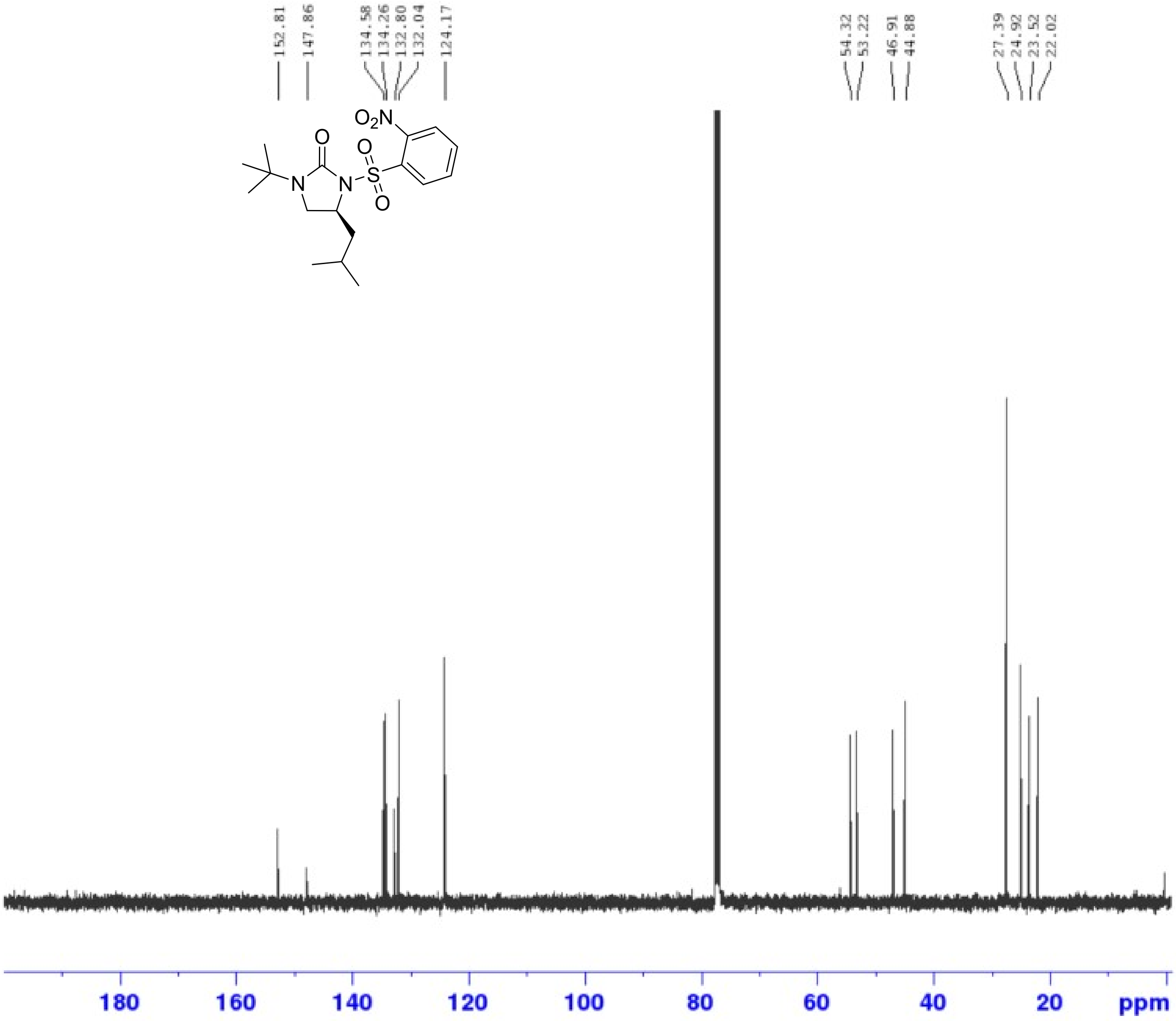


```
Current Data Parameters
NAME        TW-A-49-A RERUN
EXPNO                    23
PROCNO                    1

F2 - Acquisition Parameters
Date_              20190425
Time                  22.38
INSTRUM               spect
PROBHD     5 mm PABBO BB/
PULPROG             zgpg30
TD                   65536
SOLVENT              CDCl3
NS                    1024
DS                       4
SWH              24038.461 Hz
FIDRES            0.366798 Hz
AQ               1.3631488 sec
RG                     203
DW                  20.800 usec
DE                    6.50 usec
TE                   300.0 K
D1              2.00000000 sec
D11             0.03000000 sec
TD0                      1

======== CHANNEL f1 ========
SFO1           100.5649900 MHz
NUC1                   13C
P1                   10.00 usec
PLW1           44.46300125 W

======== CHANNEL f2 ========
SFO2           399.9015996 MHz
NUC2                    1H
CPDPRG[2            waltz16
PCPD2                90.00 usec
PLW2            7.59999990 W
PLW12           0.20774999 W
PLW13           0.16827001 W

F2 - Processing parameters
SI                   32768
SF             100.5549209 MHz
WDW                     EM
SSB                      0
LB                    1.00 Hz
GB                       0
PC                    1.40
```

S16

^{1}H NMR

400 MHz

$CDCl_3$

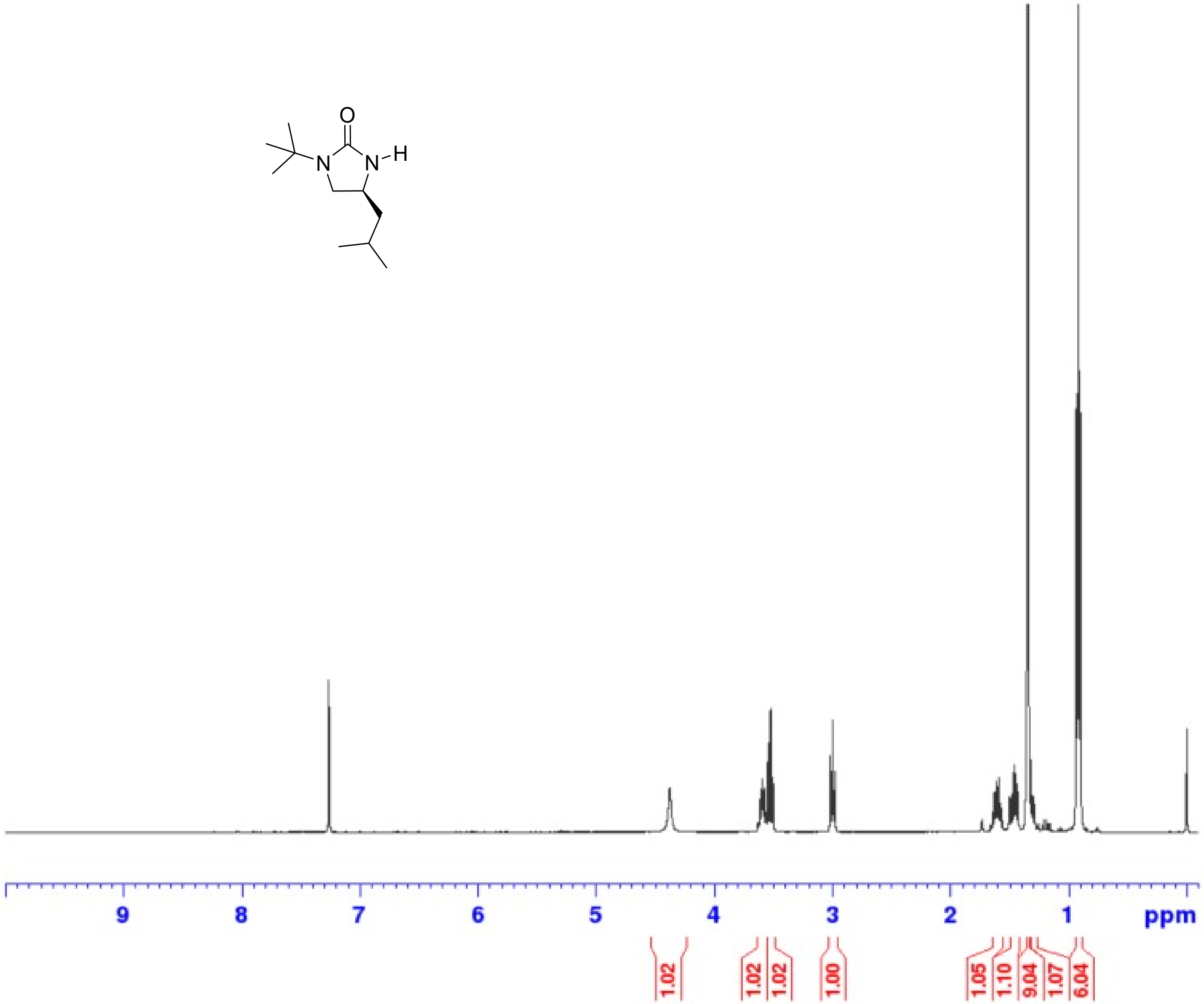


```
Current Data Parameters
NAME      TW-A-79-A RERUN
EXPNO                  30
PROCNO                  1

F2 - Acquisition Parameters
Date_            20190425
Time                12.16
INSTRUM             spect
PROBHD   5 mm PABBO BB/
PULPROG              zg30
TD                 131072
SOLVENT             CDCl3
NS                     64
DS                      0
SWH            12019.230 Hz
FIDRES          0.091699 Hz
AQ             5.4525952 sec
RG                   90.5
DW                 41.600 usec
DE                   9.85 usec
TE                  300.0 K
D1             0.10000000 sec
TD0                     1

======== CHANNEL f1 ========
SFO1         399.9024695 MHz
NUC1                   1H
P1                  14.88 usec
PLW1           7.59999990 W

F2 - Processing parameters
SI                 131072
SF            399.9000098 MHz
WDW                    EM
SSB                     0
LB                   0.10 Hz
GB                      0
PC                   1.00
```

S16

^{13}C NMR

101 MHz

$CDCl_3$

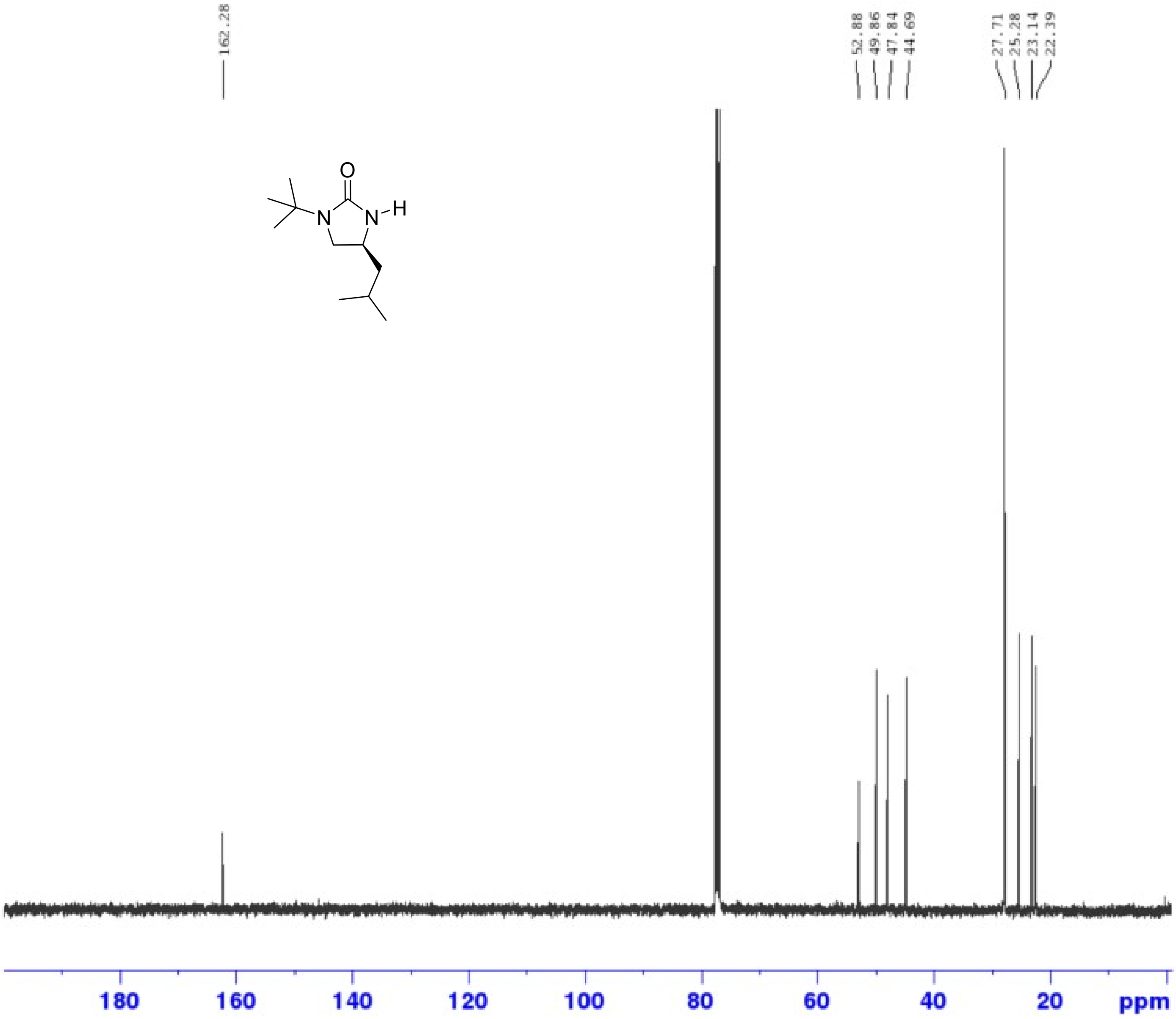


```
Current Data Parameters
NAME        TW-A-79-A RERUN
EXPNO                    33
PROCNO                    1

F2 - Acquisition Parameters
Date_              20190426
Time                   1.42
INSTRUM               spect
PROBHD    5 mm PABBO BB/
PULPROG             zgpg30
TD                    65536
SOLVENT               CDCl3
NS                     1024
DS                        4
SWH              24038.461 Hz
FIDRES            0.366798 Hz
AQ               1.3631488 sec
RG                      144
DW                   20.800 usec
DE                     6.50 usec
TE                    300.0 K
D1               2.00000000 sec
D11              0.03000000 sec
TD0                       1

======== CHANNEL f1 ========
SFO1           100.5649900 MHz
NUC1                   13C
P1                    10.00 usec
PLW1            44.46300125 W

======== CHANNEL f2 ========
SFO2           399.9015996 MHz
NUC2                    1H
CPDPRG[2            waltz16
PCPD2                 90.00 usec
PLW2             7.59999990 W
PLW12            0.20774999 W
PLW13            0.16827001 W

F2 - Processing parameters
SI                    32768
SF             100.5549213 MHz
WDW                      EM
SSB                       0
LB                     1.00 Hz
GB                        0
PC                     1.40
```

6

^{1}H NMR

400 MHz

$CDCl_3$

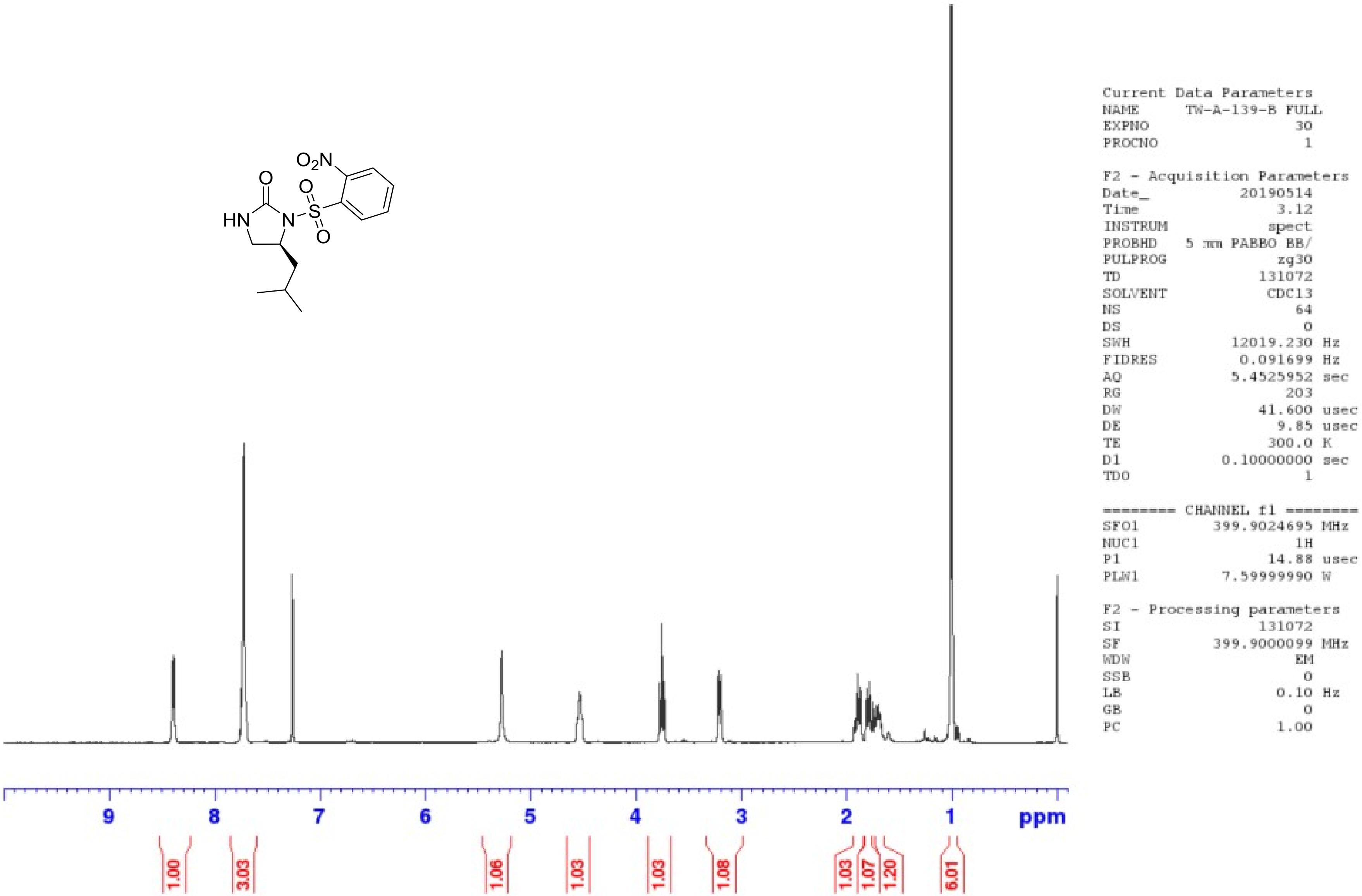

6

^{13}C NMR

101 MHz

$CDCl_3$

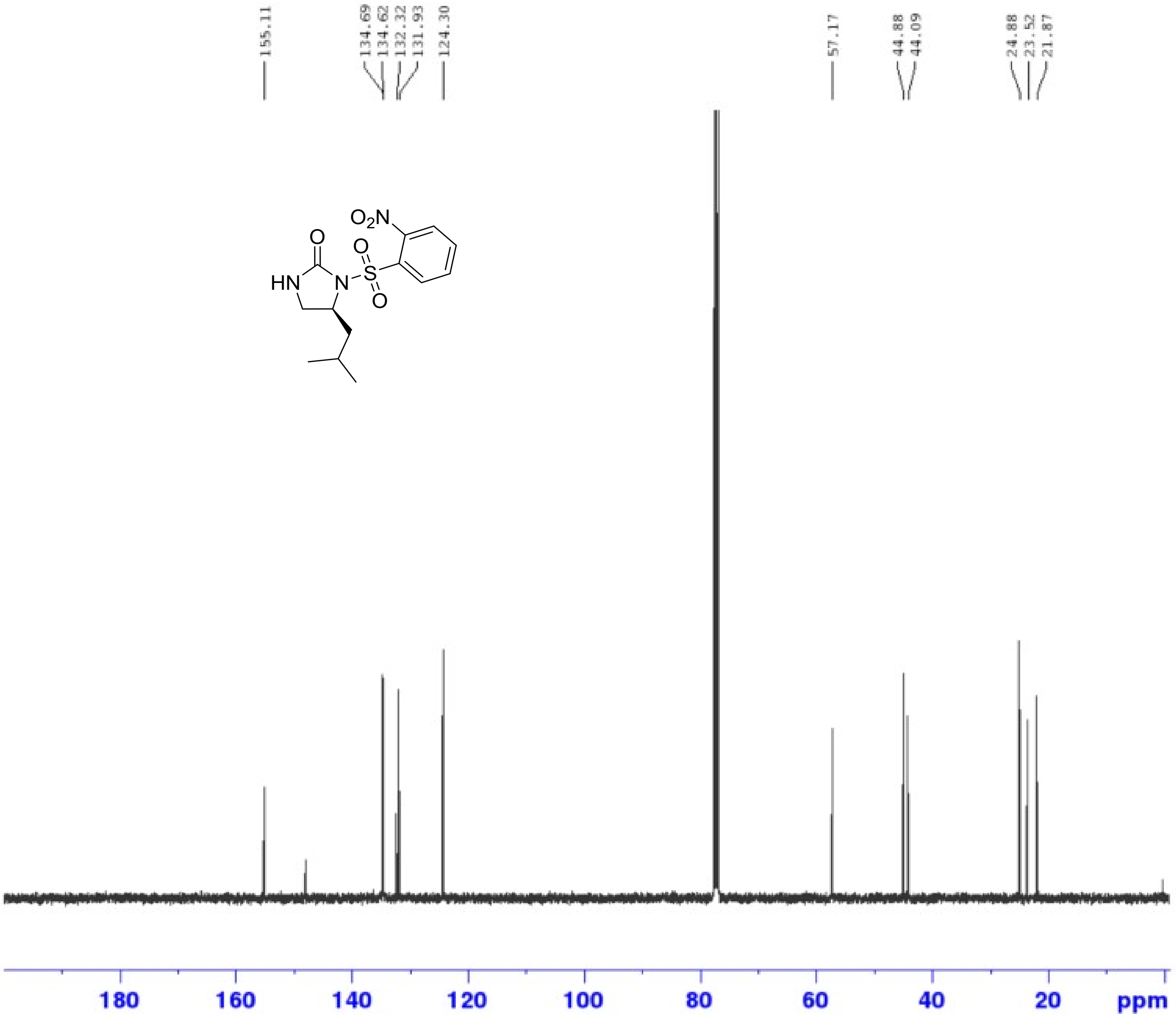

Current Data Parameters
NAME TW-A-139-B FULL
EXPNO 32
PROCNO 1

F2 - Acquisition Parameters
Date_ 20190514
Time 4.12
INSTRUM spect
PROBHD 5 mm PABBO BB/
PULPROG zgpg30
TD 65536
SOLVENT CDCl3
NS 1024
DS 4
SWH 24038.461 Hz
FIDRES 0.366798 Hz
AQ 1.3631488 sec
RG 203
DW 20.800 usec
DE 6.50 usec
TE 300.0 K
D1 2.00000000 sec
D11 0.03000000 sec
TD0 1

======== CHANNEL f1 ========
SFO1 100.5649900 MHz
NUC1 13C
P1 10.00 usec
PLW1 44.46300125 W

======== CHANNEL f2 ========
SFO2 399.9015996 MHz
NUC2 1H
CPDPRG[2 waltz16
PCPD2 90.00 usec
PLW2 7.59999990 W
PLW12 0.20774999 W
PLW13 0.16827001 W

F2 - Processing parameters
SI 32768
SF 100.5549219 MHz
WDW EM
SSB 0
LB 1.00 Hz
GB 0
PC 1.40

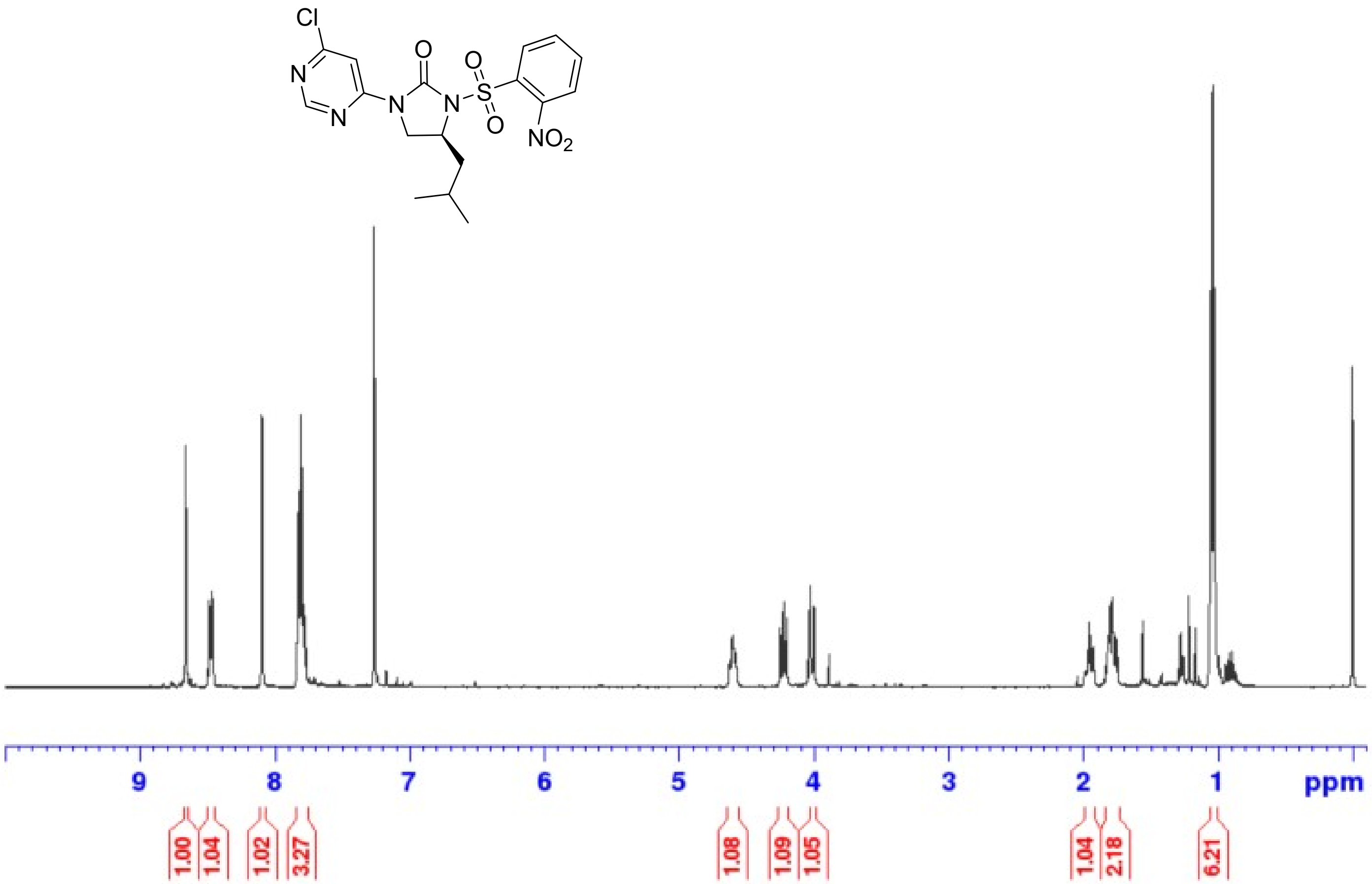

S17

^{1}H NMR

400 MHz

$CDCl_3$


```
Current Data Parameters
NAME              TW-A-195-A
EXPNO                     10
PROCNO                     1

F2 - Acquisition Parameters
Date_               20190424
Time                   14.02
INSTRUM                spect
PROBHD    5 mm PABBO BB/
PULPROG                 zg30
TD                    131072
SOLVENT                CDCl3
NS                        16
DS                         0
SWH              12019.230 Hz
FIDRES            0.091699 Hz
AQ               5.4525952 sec
RG                       203
DW                  41.600 usec
DE                    9.85 usec
TE                   300.0 K
D1              0.10000000 sec
TD0                        1

======== CHANNEL f1 ========
SFO1          399.9024695 MHz
NUC1                      1H
P1                     14.88 usec
PLW1              7.59999990 W

F2 - Processing parameters
SI                    131072
SF            399.9000096 MHz
WDW                       EM
SSB                        0
LB                      0.10 Hz
GB                         0
PC                      1.00
```

S17

^{13}C NMR

101 MHz

$CDCl_3$

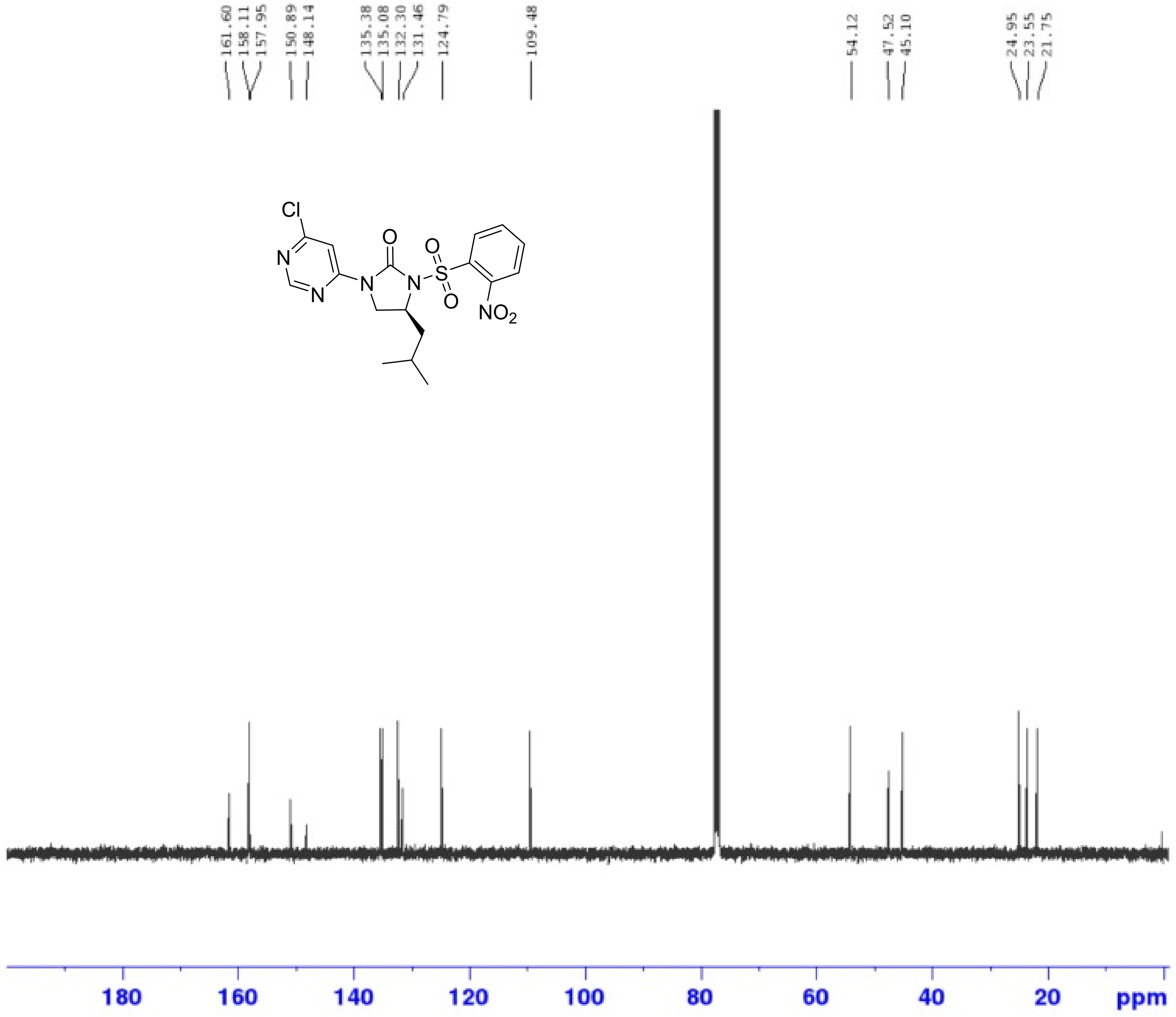

Current Data Parameters
NAME TW-A-195-A
EXPNO 14
PROCNO 1

F2 - Acquisition Parameters
Date_ 20190424
Time 20.04
INSTRUM spect
PROBHD 5 mm PABBO BB/
PULPROG zgpg30
TD 65536
SOLVENT CDCl3
NS 1024
DS 4
SWH 24038.461 Hz
FIDRES 0.366798 Hz
AQ 1.3631488 sec
RG 203
DW 20.800 usec
DE 6.50 usec
TE 300.0 K
D1 2.00000000 sec
D11 0.03000000 sec
TD0 1

======== CHANNEL f1 ========
SFO1 100.5649900 MHz
NUC1 13C
P1 10.00 usec
PLW1 44.46300125 W

======== CHANNEL f2 ========
SFO2 399.9015996 MHz
NUC2 1H
CPDPRG[2 waltz16
PCPD2 90.00 usec
PLW2 7.59999990 W
PLW12 0.20774999 W
PLW13 0.16827001 W

F2 - Processing parameters
SI 32768
SF 100.5549209 MHz
WDW EM
SSB 0
LB 1.00 Hz
GB 0
PC 1.40

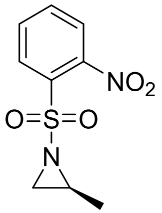

S18

^{1}H NMR

400 MHz

$CDCl_3$

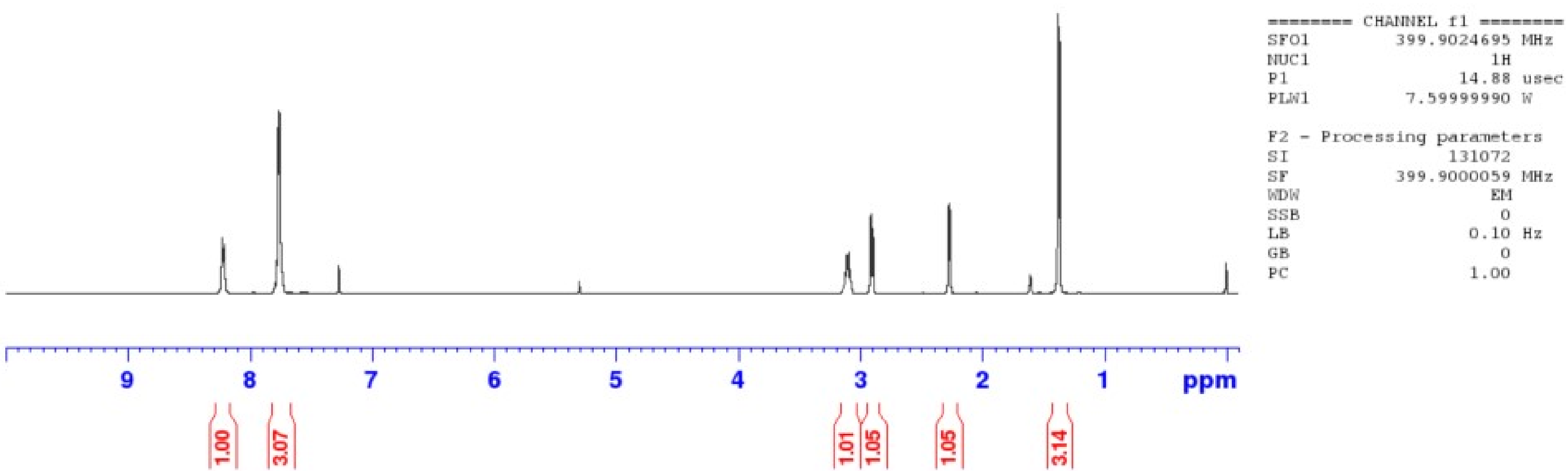

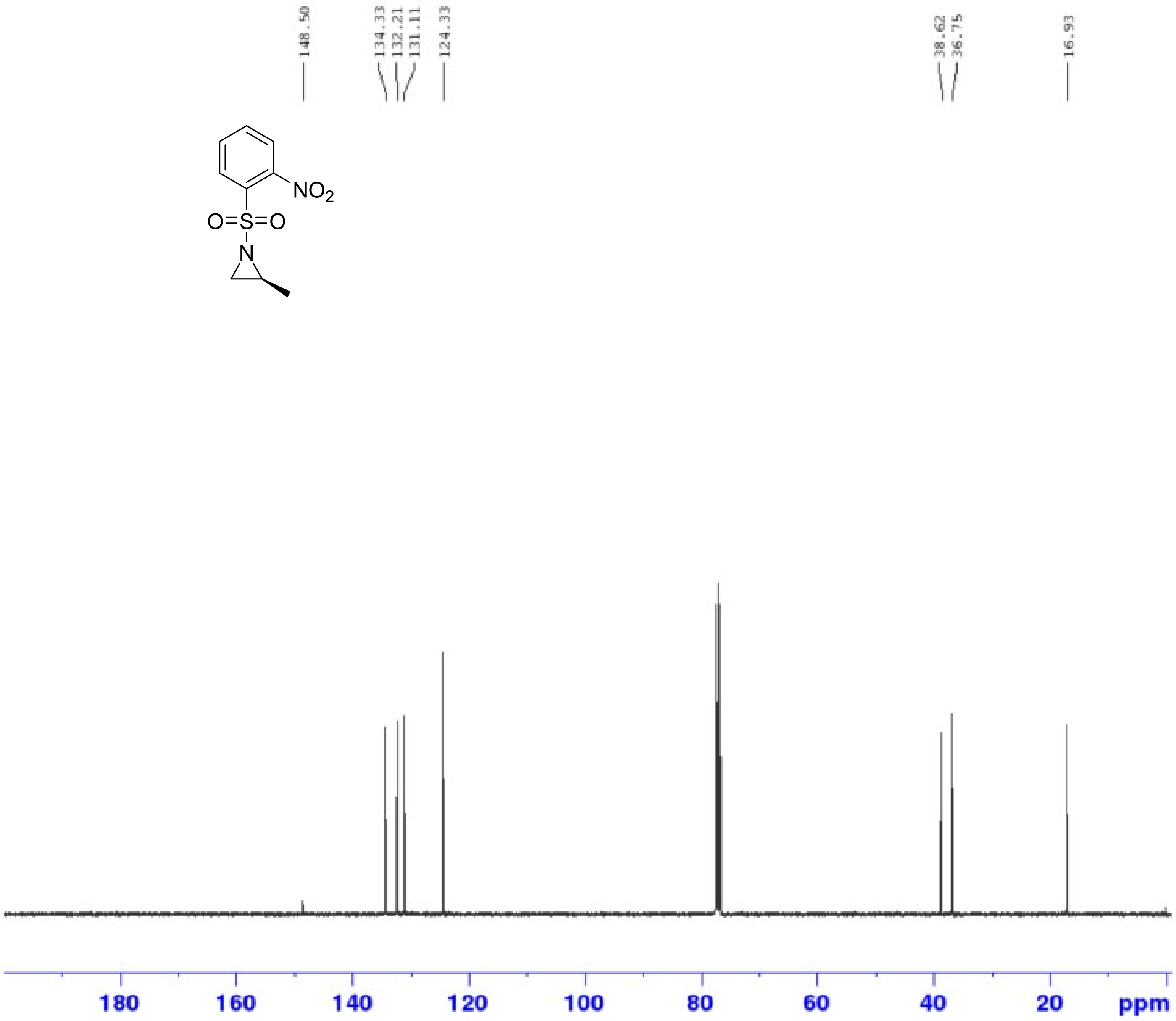

S18

^{13}C NMR

101 MHz

$CDCl_3$

```
Current Data Parameters
NAME              RK1-83-1
EXPNO                   11
PROCNO                   1

F2 - Acquisition Parameters
Date_             20181128
Time                 21.17
INSTRUM              spect
PROBHD   5 mm PABBO BB/
PULPROG            zgpg30
TD                   65536
SOLVENT              CDCl3
NS                    1024
DS                       4
SWH              24038.461 Hz
FIDRES            0.366798 Hz
AQ               1.3631488 sec
RG                     203
DW                  20.800 usec
DE                    6.50 usec
TE                   298.6 K
D1              2.00000000 sec
D11             0.03000000 sec
TD0                      1

======== CHANNEL f1 ========
SFO1          100.5649900 MHz
NUC1                  13C
P1                  10.00 usec
PLW1          44.46300125 W

======== CHANNEL f2 ========
SFO2          399.9015996 MHz
NUC2                   1H
CPDPRG[2          waltz16
PCPD2               90.00 usec
PLW2           7.59999990 W
PLW12          0.20774999 W
PLW13          0.16827001 W

F2 - Processing parameters
SI                  32768
SF            100.5549353 MHz
WDW                    EM
SSB                     0
LB                   1.00 Hz
GB                      0
PC                   1.40
```

S19

^{1}H NMR

400 MHz

$CDCl_3$

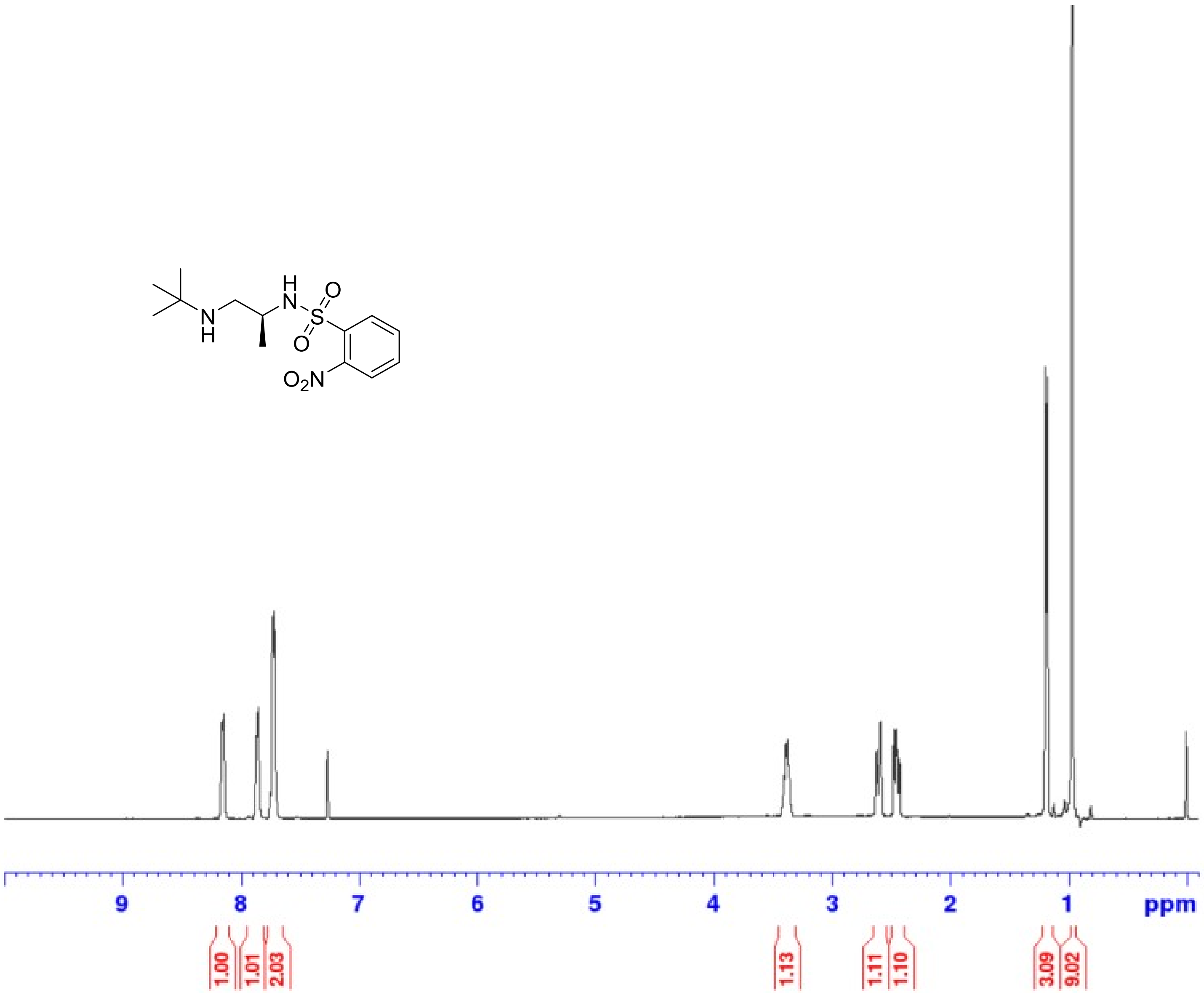


```
Current Data Parameters
NAME        RK1-89-recrys
EXPNO                  10
PROCNO                  1

F2 - Acquisition Parameters
Date_            20190108
Time                17.53
INSTRUM             spect
PROBHD    5 mm PABBO BB/
PULPROG              zg30
TD                 131072
SOLVENT             CDCl3
NS                     16
DS                      0
SWH             12019.230 Hz
FIDRES           0.091699 Hz
AQ              5.4525952 sec
RG                   90.5
DW                 41.600 usec
DE                   9.85 usec
TE                  300.0 K
D1             0.10000000 sec
TD0                     1

======== CHANNEL f1 ========
SFO1          399.9024695 MHz
NUC1                   1H
P1                  14.88 usec
PLW1           7.59999990 W

F2 - Processing parameters
SI                 131072
SF            399.9000070 MHz
WDW                    EM
SSB                     0
LB                   0.10 Hz
GB                      0
PC                   1.00
```

S19

^{13}C NMR

101 MHz

$CDCl_3$

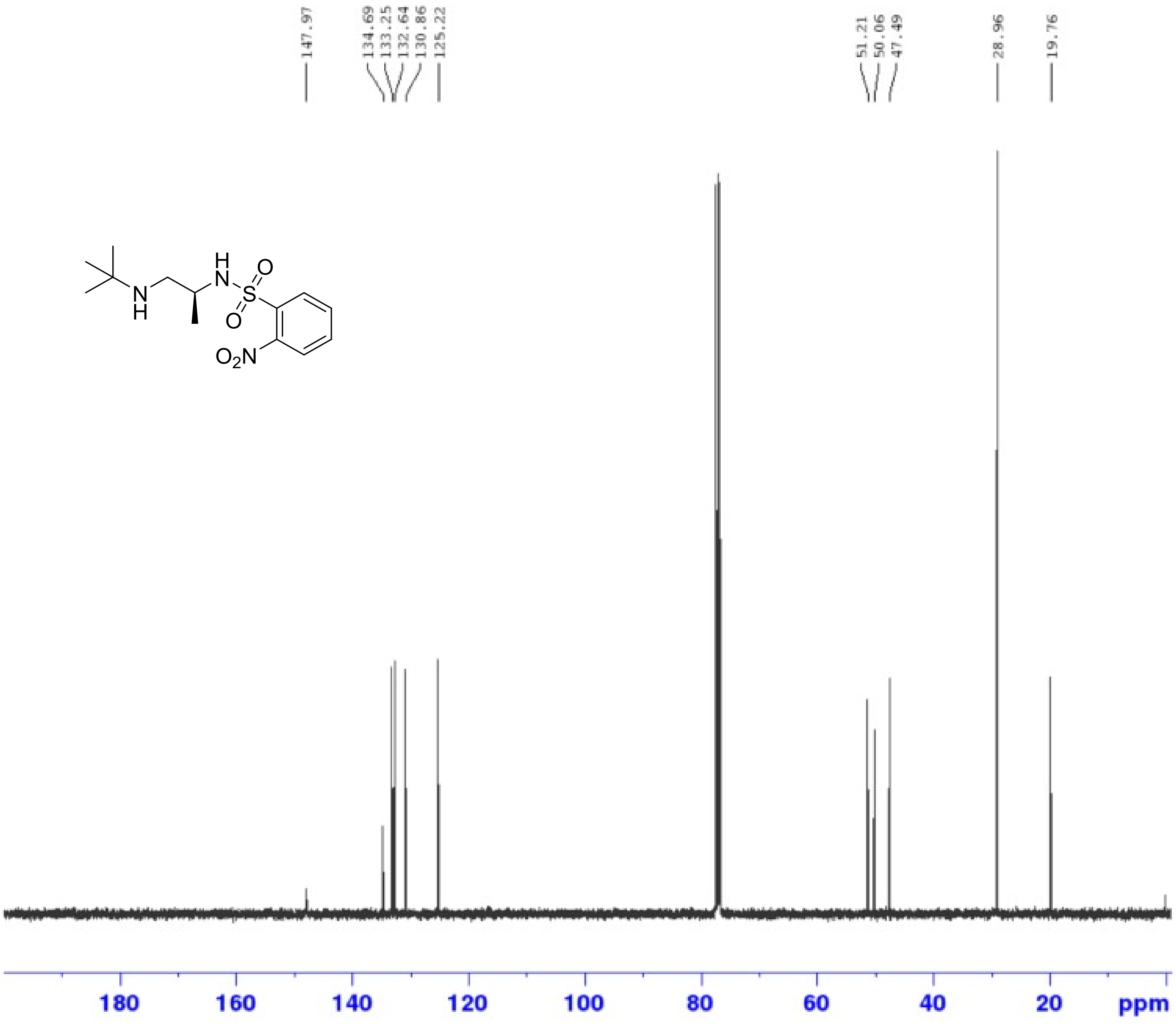


```
Current Data Parameters
NAME        RK1-89-recrys
EXPNO                  14
PROCNO                  1

F2 - Acquisition Parameters
Date_            20190108
Time                20.49
INSTRUM             spect
PROBHD    5 mm PABBO BB/
PULPROG           zgpg30
TD                119044
SOLVENT            CDCl3
NS                    512
DS                      4
SWH          25000.000 Hz
FIDRES        0.210006 Hz
AQ           2.3808801 sec
RG                   2050
DW              20.000 usec
DE                9.12 usec
TE               300.0 K
D1          1.00000000 sec
D11         0.03000000 sec
TD0                     1

======== CHANNEL f1 ========
SFO1       100.5659947 MHz
NUC1                  13C
P1              10.00 usec
PLW1        44.46300125 W

======== CHANNEL f2 ========
SFO2       399.9015996 MHz
NUC2                   1H
CPDPRG[2          waltz64
PCPD2           90.00 usec
PLW2         7.59999990 W
PLW12        0.20774999 W
PLW13        0.16827001 W

F2 - Processing parameters
SI                 131072
SF         100.5549350 MHz
WDW                    EM
SSB                     0
LB                1.00 Hz
GB                      0
PC                   1.40
```

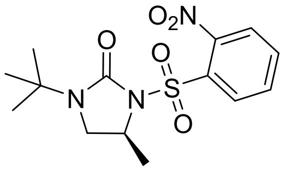

S20

^{1}H NMR

400 MHz

$CDCl_3$

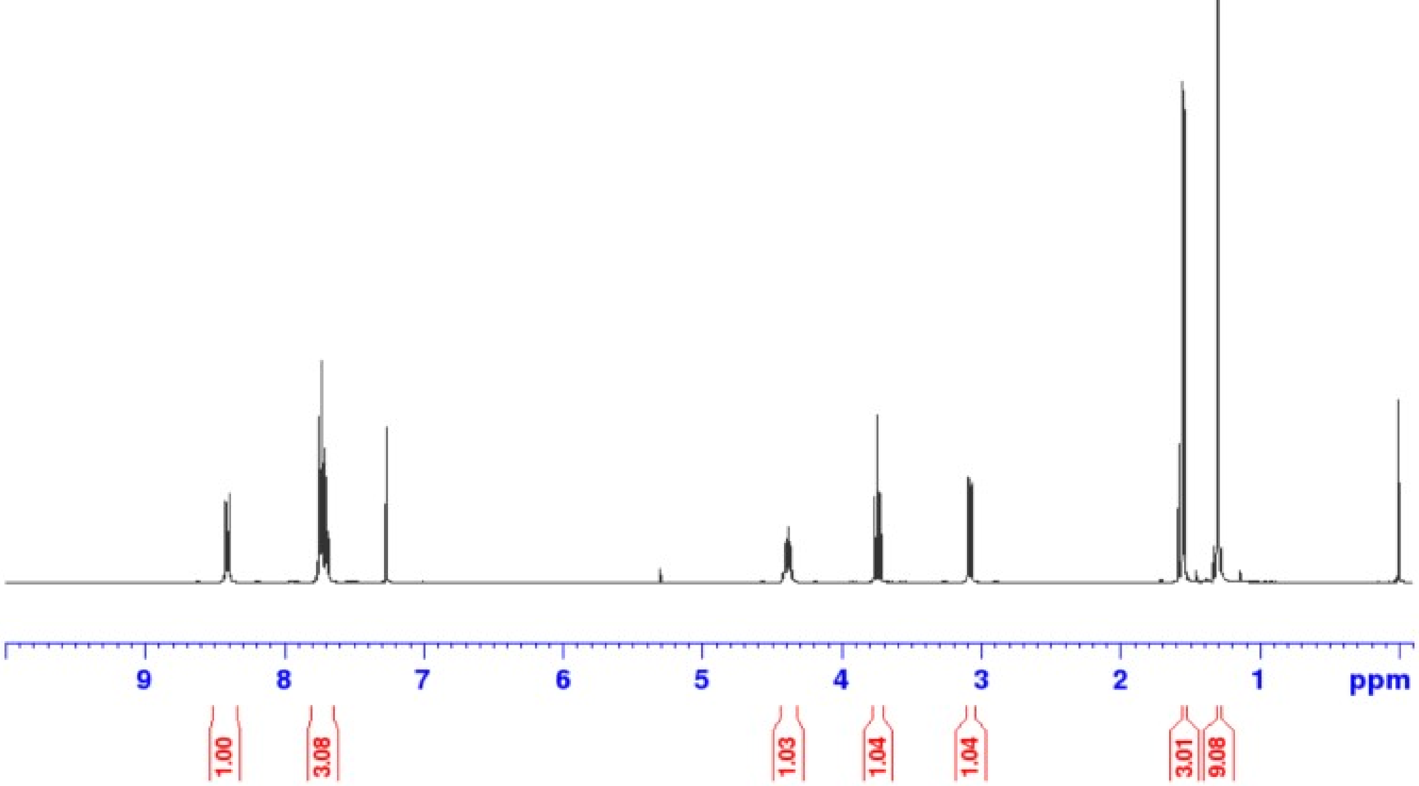

Current Data Parameters
NAME RK1-108-1
EXPNO 10
PROCNO 1

F2 - Acquisition Parameters
Date_ 20190321
Time 13.27
INSTRUM AVIII_400
PROBHD 5 mm PABBO BB-
PULPROG zg30
TD 65536
SOLVENT CDCl3
NS 16
DS 2
SWH 8223.685 Hz
FIDRES 0.125483 Hz
AQ 3.9845889 sec
RG 144
DW 60.800 usec
DE 16.82 usec
TE 300.0 K
D1 1.00000000 sec
TD0 1

======== CHANNEL f1 ========
SFO1 400.1124708 MHz
NUC1 1H
P1 15.00 usec
PLW1 17.29199982 W

F2 - Processing parameters
SI 32768
SF 400.1100077 MHz
WDW EM
SSB 0
LB 0.30 Hz
GB 0
PC 1.00

S20

^{13}C NMR

101 MHz

$CDCl_3$

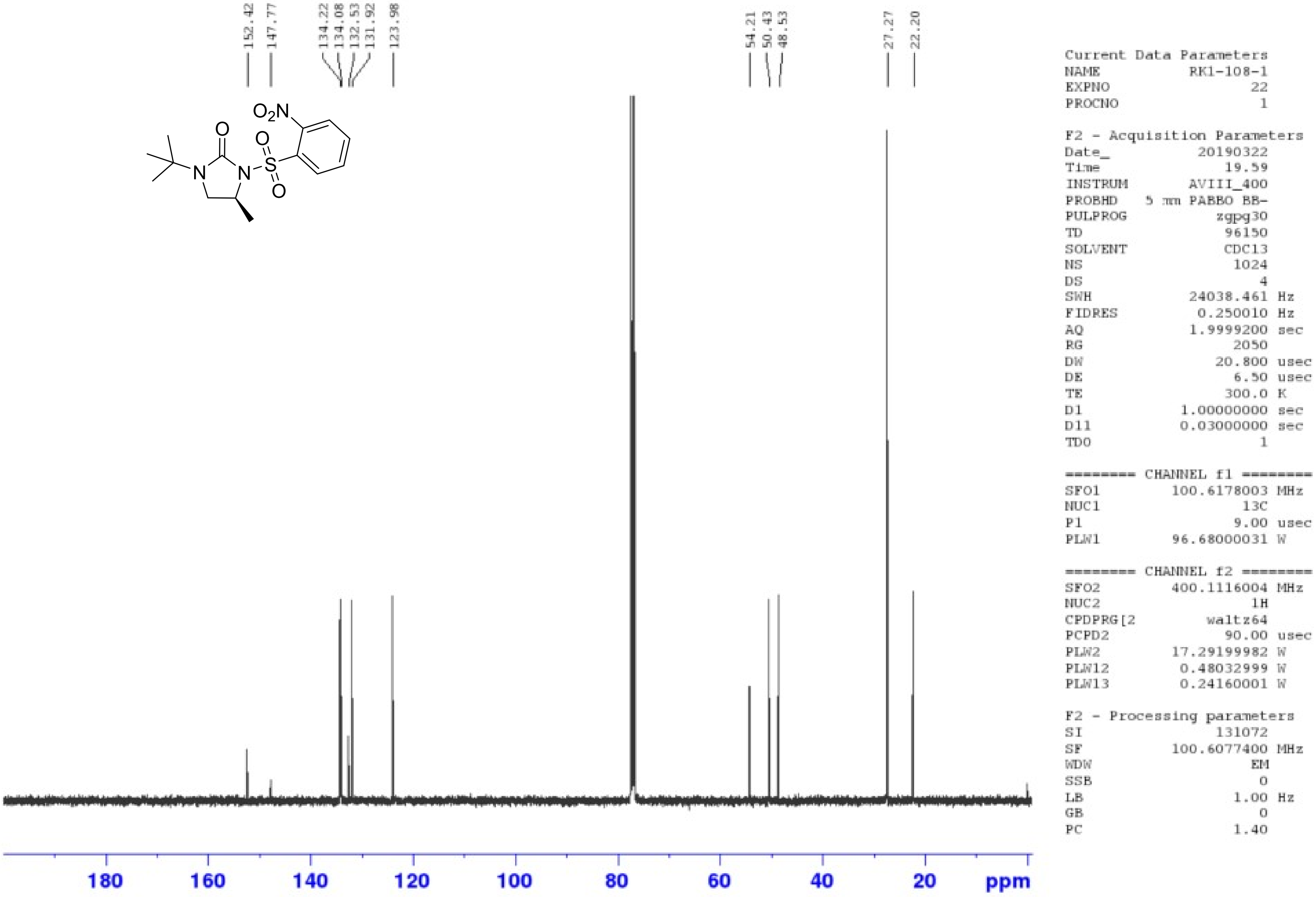

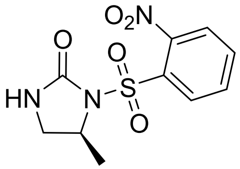

7

^{1}H NMR

400 MHz

$CDCl_3$

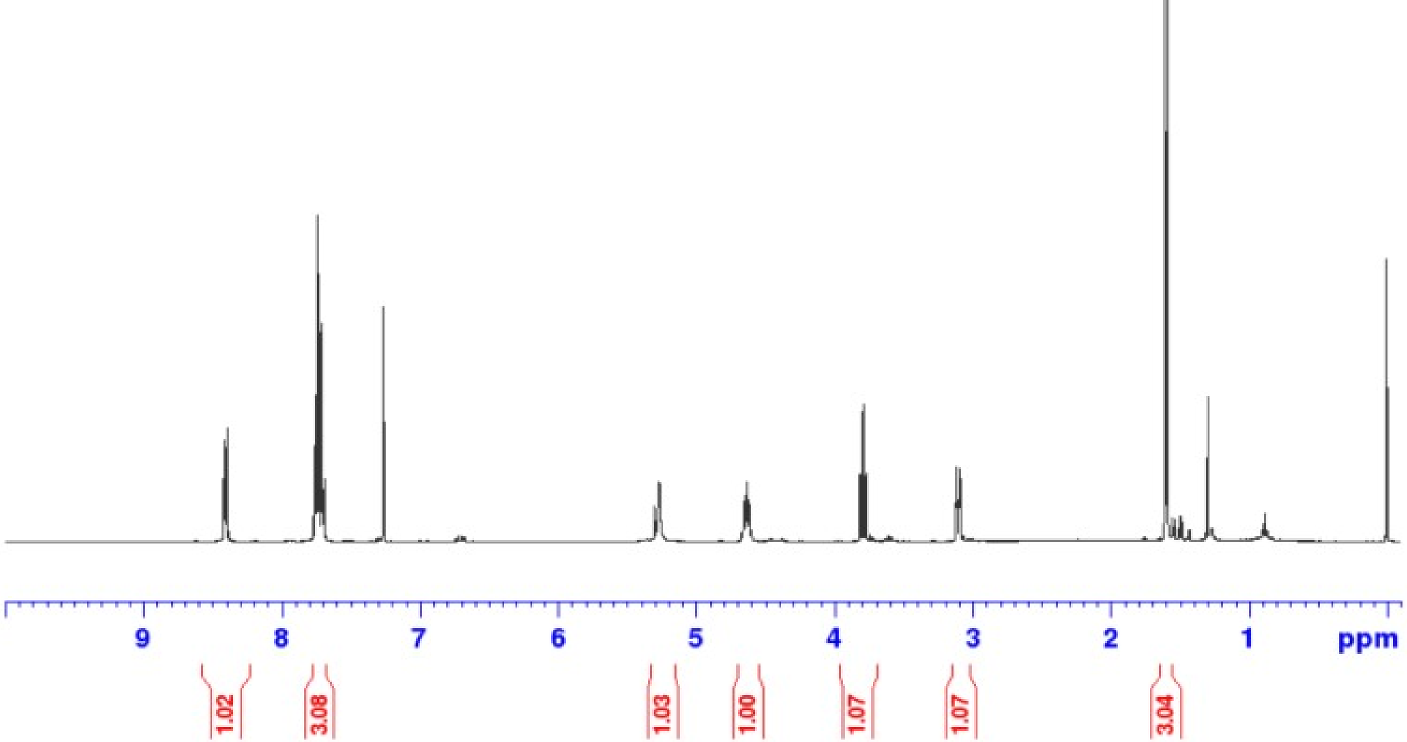


```
Current Data Parameters
NAME            RK1-112-1
EXPNO                  10
PROCNO                  1

F2 - Acquisition Parameters
Date_          20190328
Time              12.24
INSTRUM       AVIII_400
PROBHD   5 mm PABBO BB-
PULPROG            zg30
TD                65536
SOLVENT           CDCl3
NS                   16
DS                    2
SWH            8223.685 Hz
FIDRES         0.125483 Hz
AQ            3.9845889 sec
RG                  181
DW               60.800 usec
DE                16.82 usec
TE                300.0 K
D1           1.00000000 sec
TD0                   1

======== CHANNEL f1 ========
SFO1        400.1124708 MHz
NUC1                 1H
P1                15.00 usec
PLW1        17.29199982 W

F2 - Processing parameters
SI                32768
SF          400.1100083 MHz
WDW                  EM
SSB                   0
LB                 0.30 Hz
GB                    0
PC                 1.00
```

7

^{13}C NMR

101 MHz

$CDCl_3$

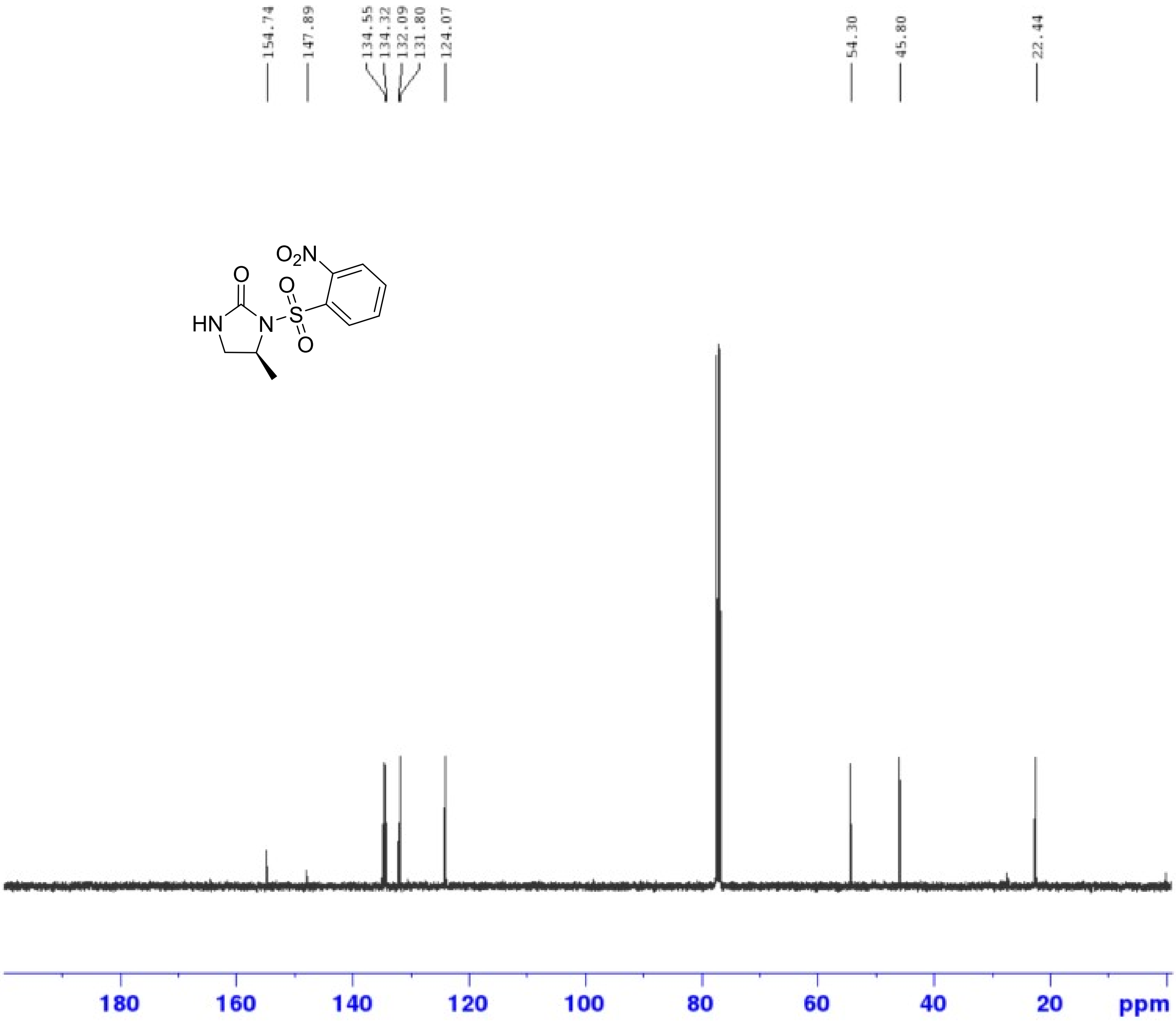


```
Current Data Parameters
NAME              RK1-112-1
EXPNO                    11
PROCNO                    1

F2 - Acquisition Parameters
Date_              20190328
Time                  12.51
INSTRUM           AVIII_400
PROBHD     5 mm PABBO BB-
PULPROG             zgpg30
TD                   96150
SOLVENT              CDCl3
NS                     512
DS                       4
SWH           24038.461 Hz
FIDRES         0.250010 Hz
AQ            1.9999200 sec
RG                    2050
DW              20.800 usec
DE               6.50 usec
TE                300.0 K
D1           1.00000000 sec
D11          0.03000000 sec
TD0                      1

======== CHANNEL f1 ========
SFO1       100.6178003 MHz
NUC1                   13C
P1                9.00 usec
PLW1         96.68000031 W

======== CHANNEL f2 ========
SFO2       400.1116004 MHz
NUC2                    1H
CPDPRG[2           waltz64
PCPD2            90.00 usec
PLW2         17.29199982 W
PLW12         0.48032999 W
PLW13         0.24160001 W

F2 - Processing parameters
SI                  131072
SF         100.6077400 MHz
WDW                     EM
SSB                      0
LB                 1.00 Hz
GB                       0
PC                    1.40
```

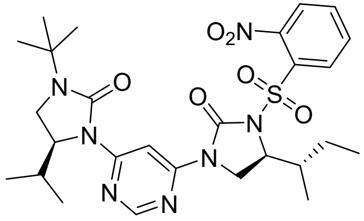

S21

^{1}H NMR

400 MHz

$CDCl_3$

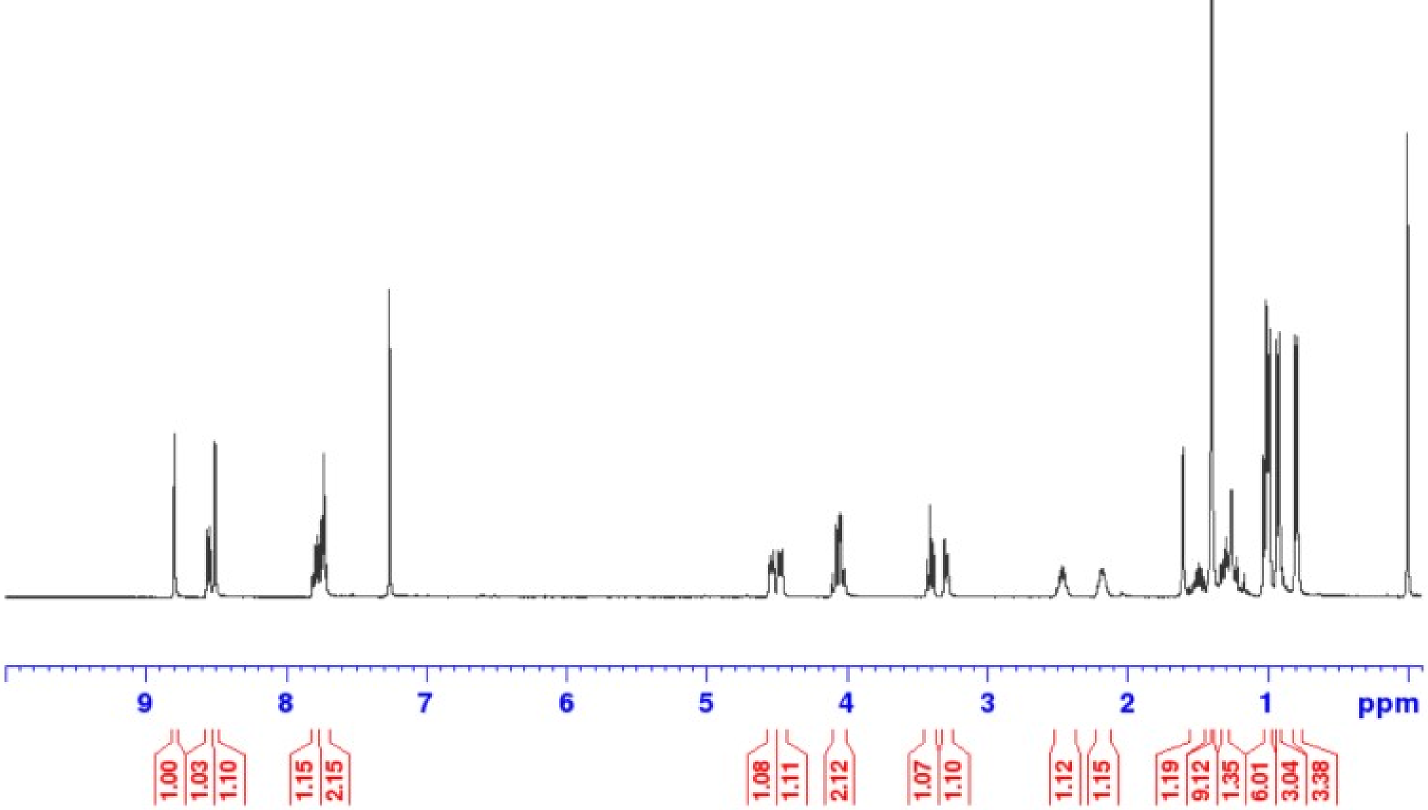


```
Current Data Parameters
NAME      TW-A-177-A RERUN
EXPNO                   10
PROCNO                   1

F2 - Acquisition Parameters
Date_             20190426
Time                 20.14
INSTRUM              spect
PROBHD     5 mm PABBO BB/
PULPROG               zg30
TD                  131072
SOLVENT              CDCl3
NS                      64
DS                       0
SWH              12019.230 Hz
FIDRES            0.091699 Hz
AQ               5.4525952 sec
RG                     203
DW                  41.600 usec
DE                    9.85 usec
TE                   300.0 K
D1              0.10000000 sec
TD0                      1

======== CHANNEL f1 ========
SFO1           399.9024695 MHz
NUC1                    1H
P1                   14.88 usec
PLW1            7.59999990 W

F2 - Processing parameters
SI                  131072
SF             399.9000097 MHz
WDW                     EM
SSB                      0
LB                    0.10 Hz
GB                       0
PC                    1.00
```

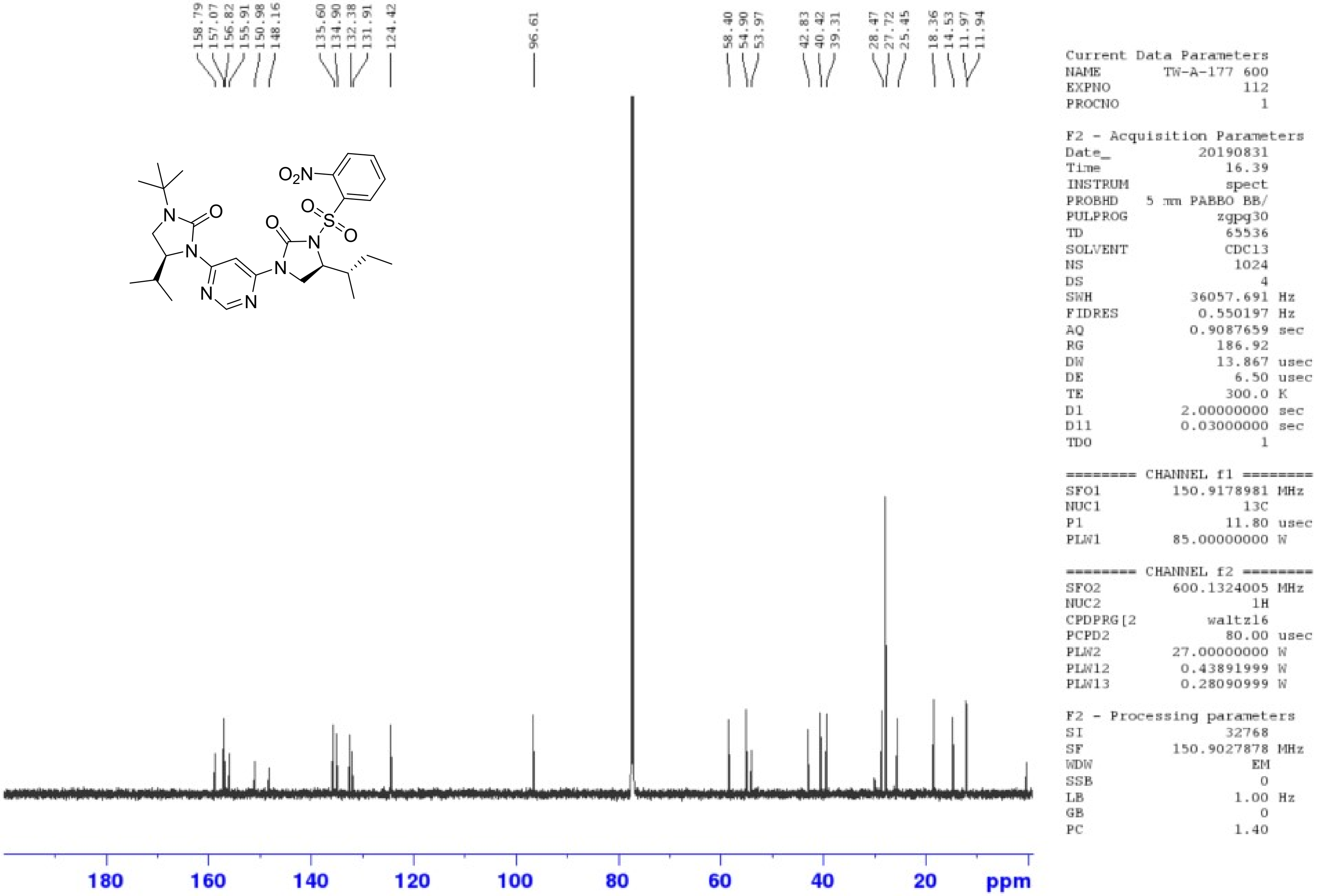

S21

^{13}C NMR

101 MHz

$CDCl_3$

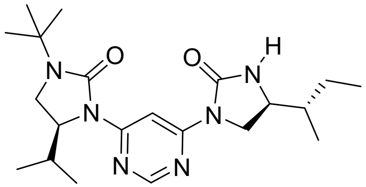

S22

^1H NMR

600 MHz

$CDCl_3$

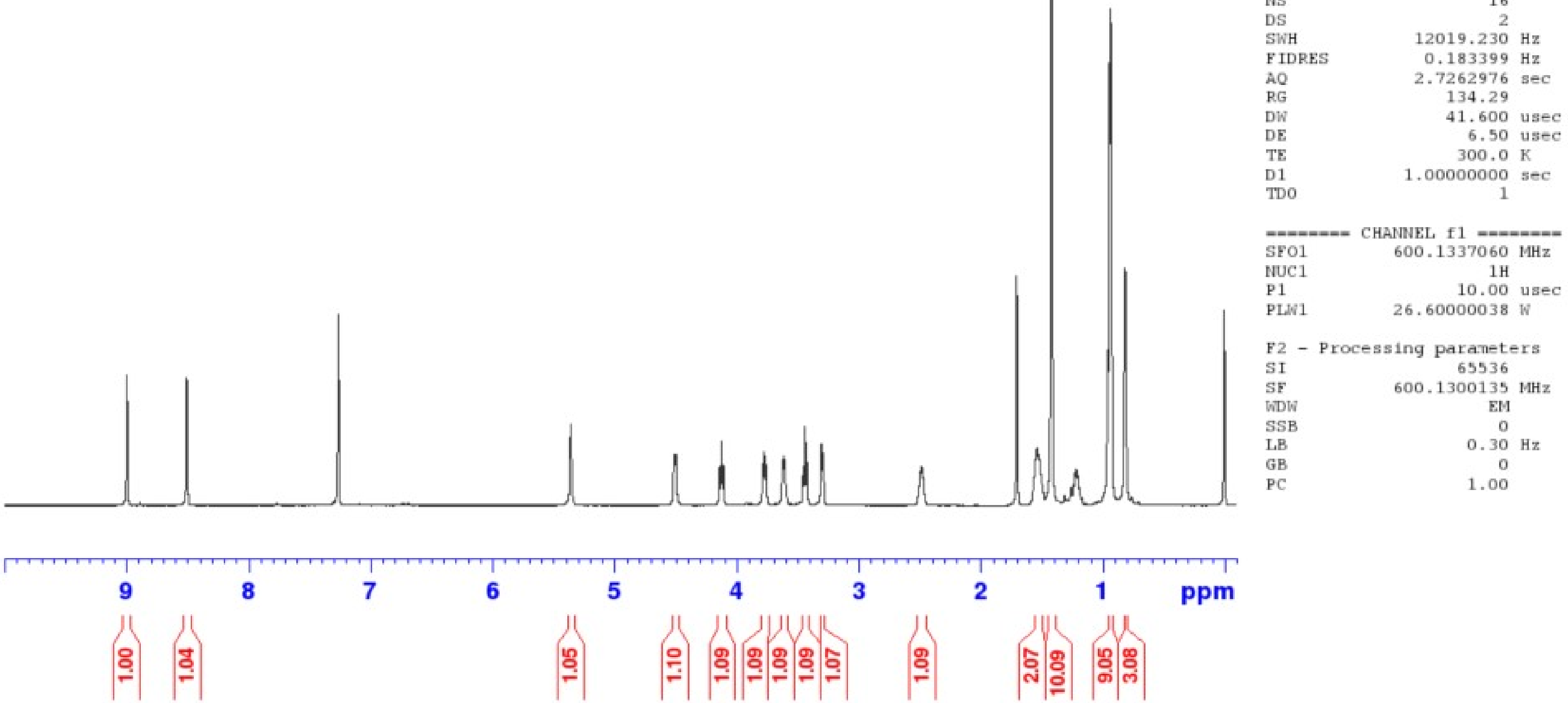

S22

^{13}C NMR

151 MHz

$CDCl_3$

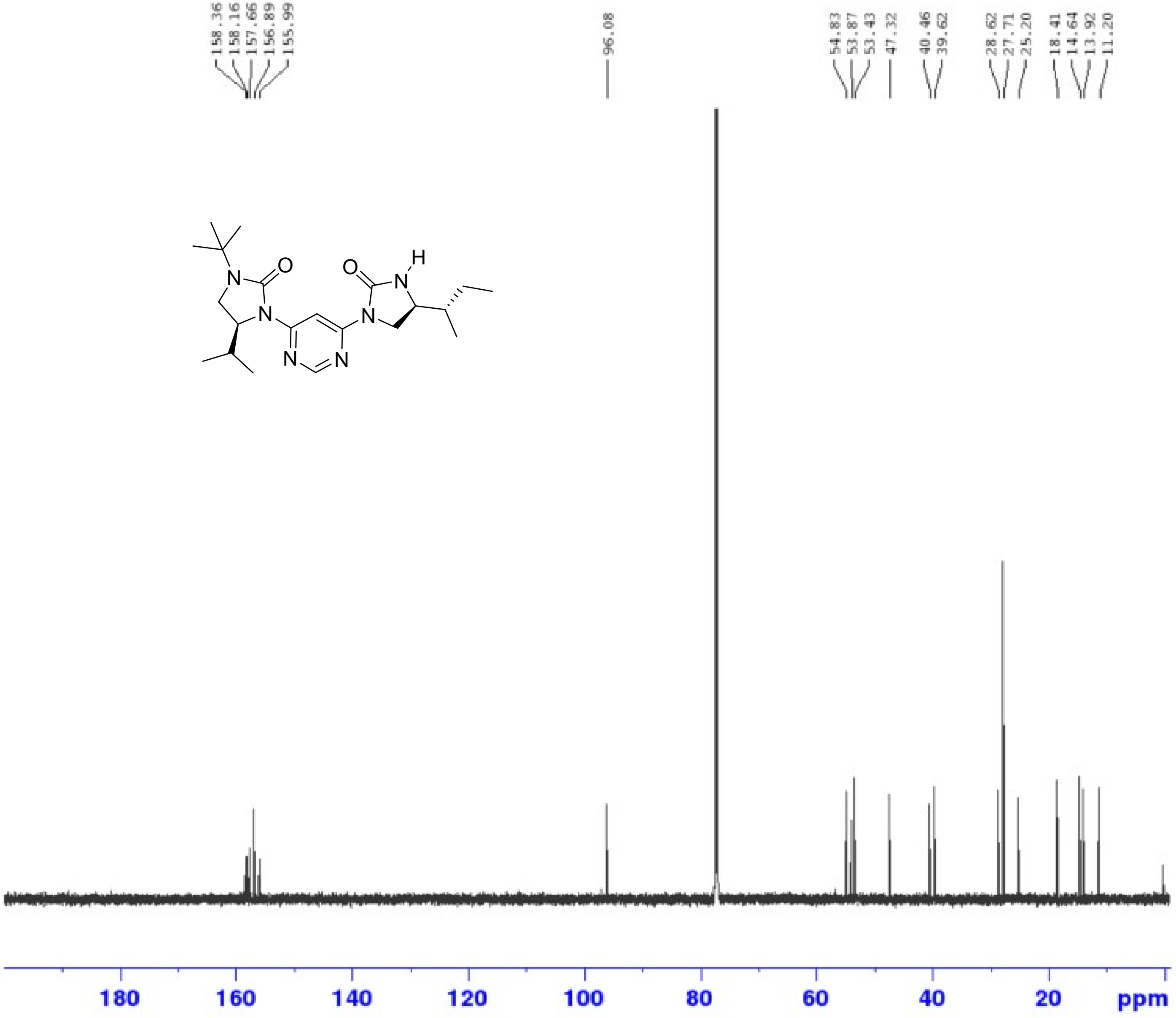


```
Current Data Parameters
NAME        TW-A-183-A TRIT 600
EXPNO                    21
PROCNO                    1

F2 - Acquisition Parameters
Date_              20190910
Time                  17.56
INSTRUM               spect
PROBHD     5 mm PABBO BB/
PULPROG             zgpg30
TD                   65536
SOLVENT              CDCl3
NS                    1024
DS                       4
SWH              36057.691 Hz
FIDRES            0.550197 Hz
AQ               0.9087659 sec
RG                  186.92
DW                  13.867 usec
DE                    6.50 usec
TE                   300.0 K
D1              2.00000000 sec
D11             0.03000000 sec
TD0                      1

======== CHANNEL f1 ========
SFO1           150.9178981 MHz
NUC1                   13C
P1                   11.80 usec
PLW1           85.00000000 W

======== CHANNEL f2 ========
SFO2           600.1324005 MHz
NUC2                    1H
CPDPRG[2           waltz16
PCPD2                80.00 usec
PLW2           27.00000000 W
PLW12           0.43891999 W
PLW13           0.28090999 W

F2 - Processing parameters
SI                   32768
SF             150.9027879 MHz
WDW                     EM
SSB                      0
LB                    1.00 Hz
GB                       0
PC                    1.40
```

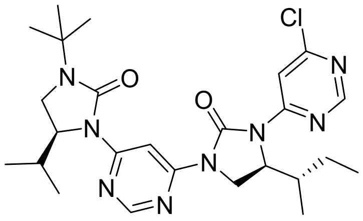

1a

^{1}H NMR

600 MHz

$CDCl_3$

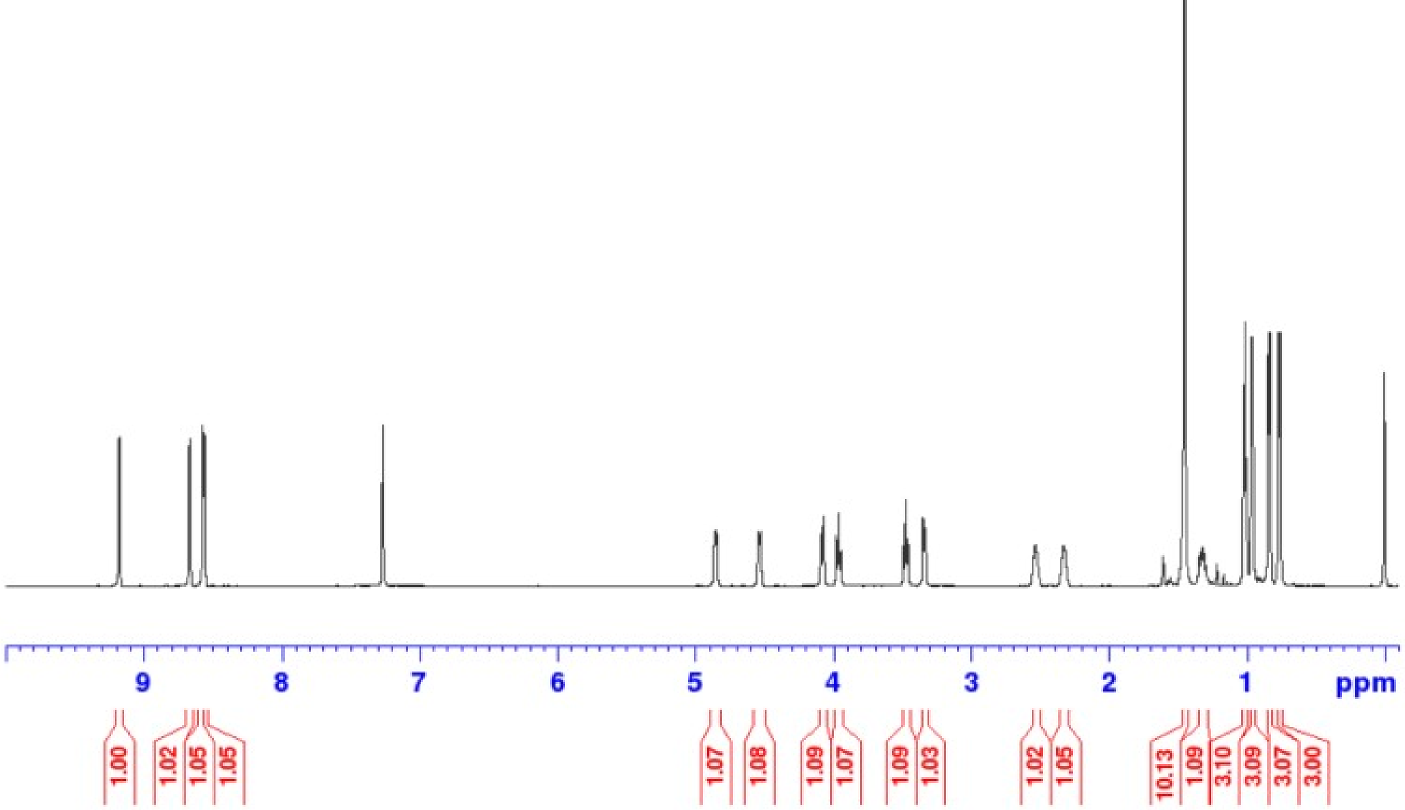

Current Data Parameters
NAME TW-A-184 TRIT 600
EXPNO 10
PROCNO 1

F2 - Acquisition Parameters
Date_ 20190904
Time 10.43
INSTRUM spect
PROBHD 5 mm PABBO BB/
PULPROG zg30
TD 65536
SOLVENT CDCl3
NS 16
DS 2
SWH 12019.230 Hz
FIDRES 0.183399 Hz
AQ 2.7262976 sec
RG 119.2
DW 41.600 usec
DE 6.50 usec
TE 300.0 K
D1 1.00000000 sec
TD0 1

======== CHANNEL f1 ========
SFO1 600.1337060 MHz
NUC1 1H
P1 10.00 usec
PLW1 26.60000038 W

F2 - Processing parameters
SI 65536
SF 600.1300127 MHz
WDW EM
SSB 0
LB 0.30 Hz
GB 0
PC 1.00

1a

^{13}C NMR

151 MHz

$CDCl_3$

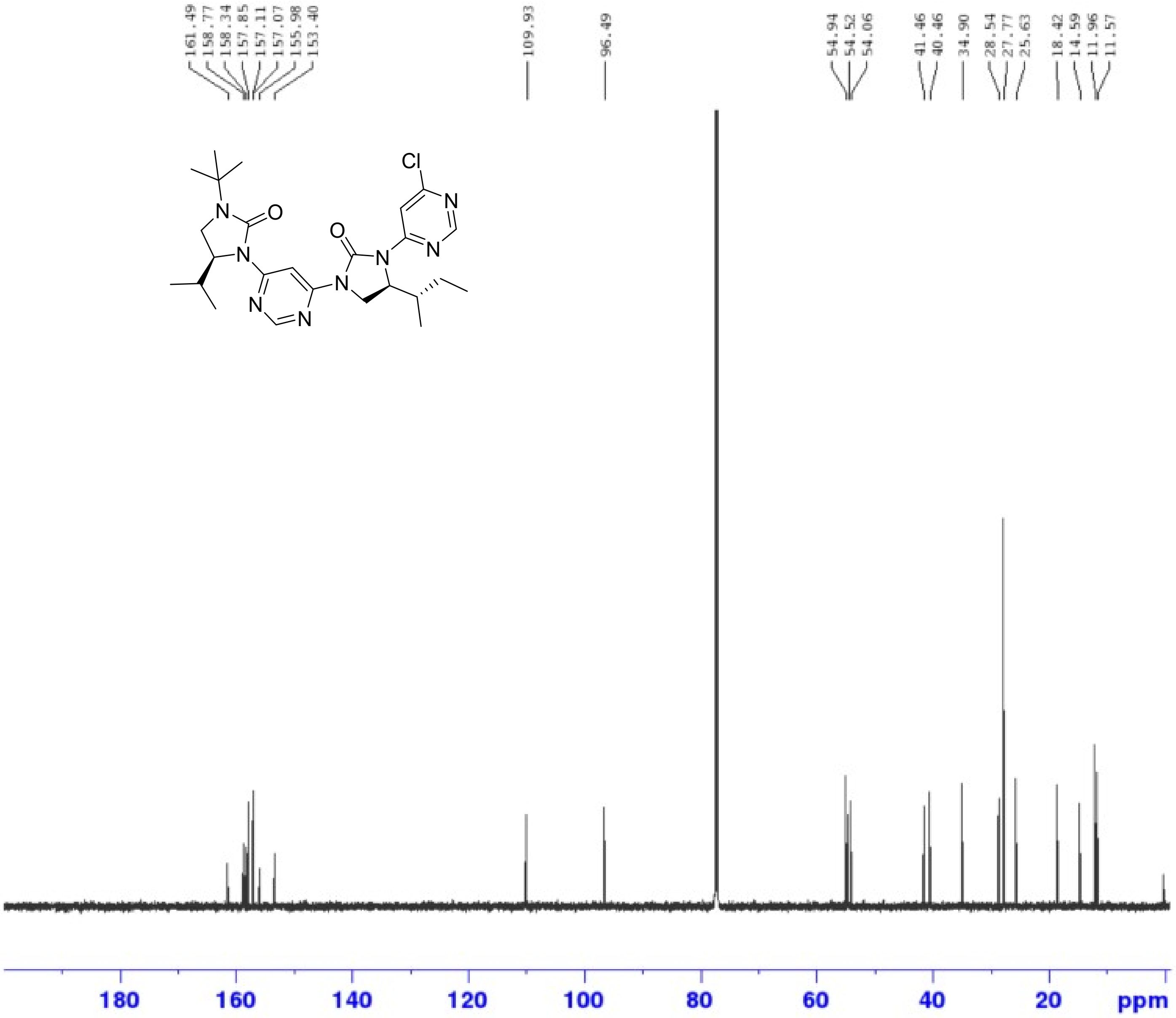

Current Data Parameters
NAME TW-A-184 TRIT 600
EXPNO 11
PROCNO 1

F2 - Acquisition Parameters
Date_ 20190906
Time 18.27
INSTRUM spect
PROBHD 5 mm PABBO BB/
PULPROG zgpg30
TD 65536
SOLVENT CDCl3
NS 1024
DS 4
SWH 36057.691 Hz
FIDRES 0.550197 Hz
AQ 0.9087659 sec
RG 186.92
DW 13.867 usec
DE 6.50 usec
TE 300.0 K
D1 2.00000000 sec
D11 0.03000000 sec
TD0 1

======== CHANNEL f1 ========
SFO1 150.9178981 MHz
NUC1 13C
P1 11.80 usec
PLW1 85.00000000 W

======== CHANNEL f2 ========
SFO2 600.1324005 MHz
NUC2 1H
CPDPRG[2 waltz16
PCPD2 80.00 usec
PLW2 27.00000000 W
PLW12 0.43891999 W
PLW13 0.28090999 W

F2 - Processing parameters
SI 32768
SF 150.9027876 MHz
WDW EM
SSB 0
LB 1.00 Hz
GB 0
PC 1.40

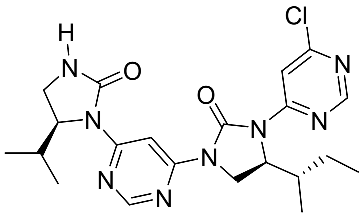

2a

^{1}H NMR

600 MHz

$CDCl_3$

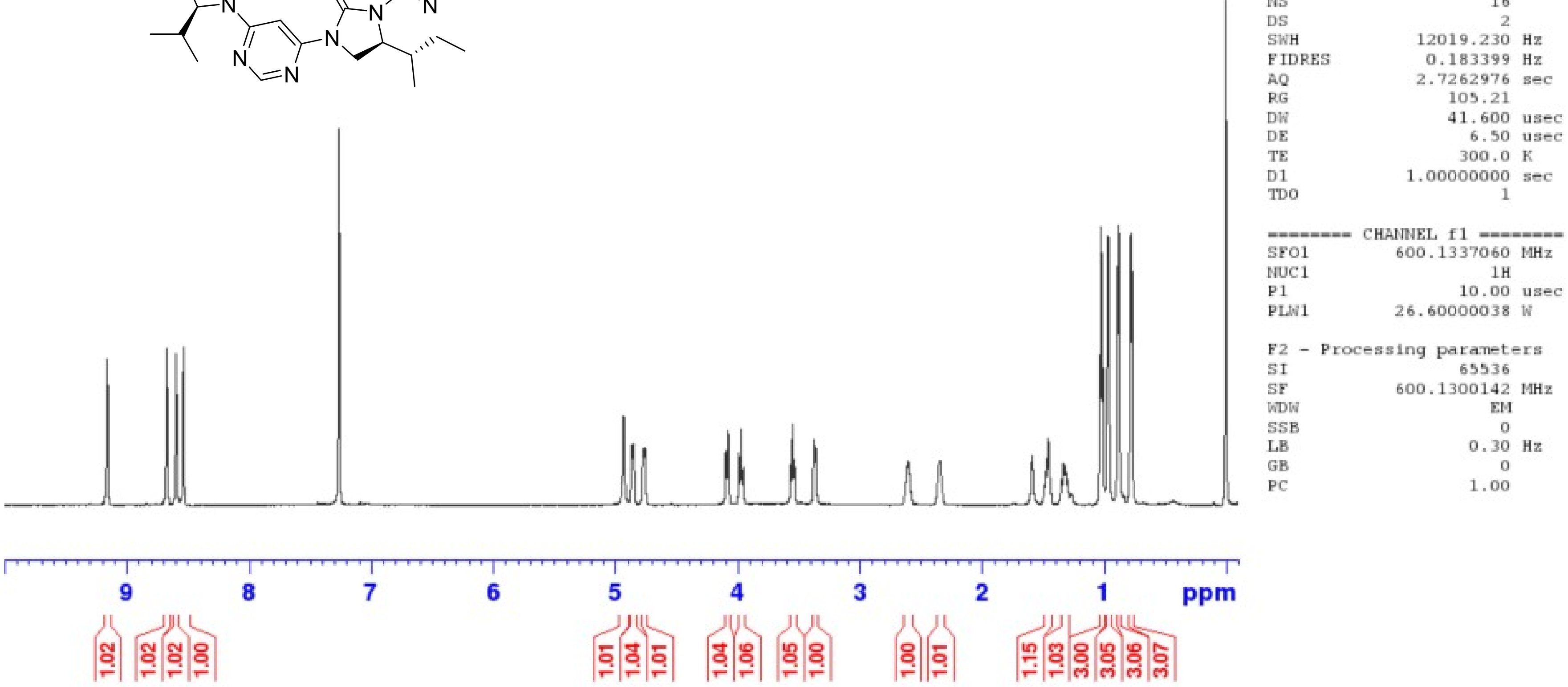

2a

^{13}C NMR

151 MHz

$CDCl_3$

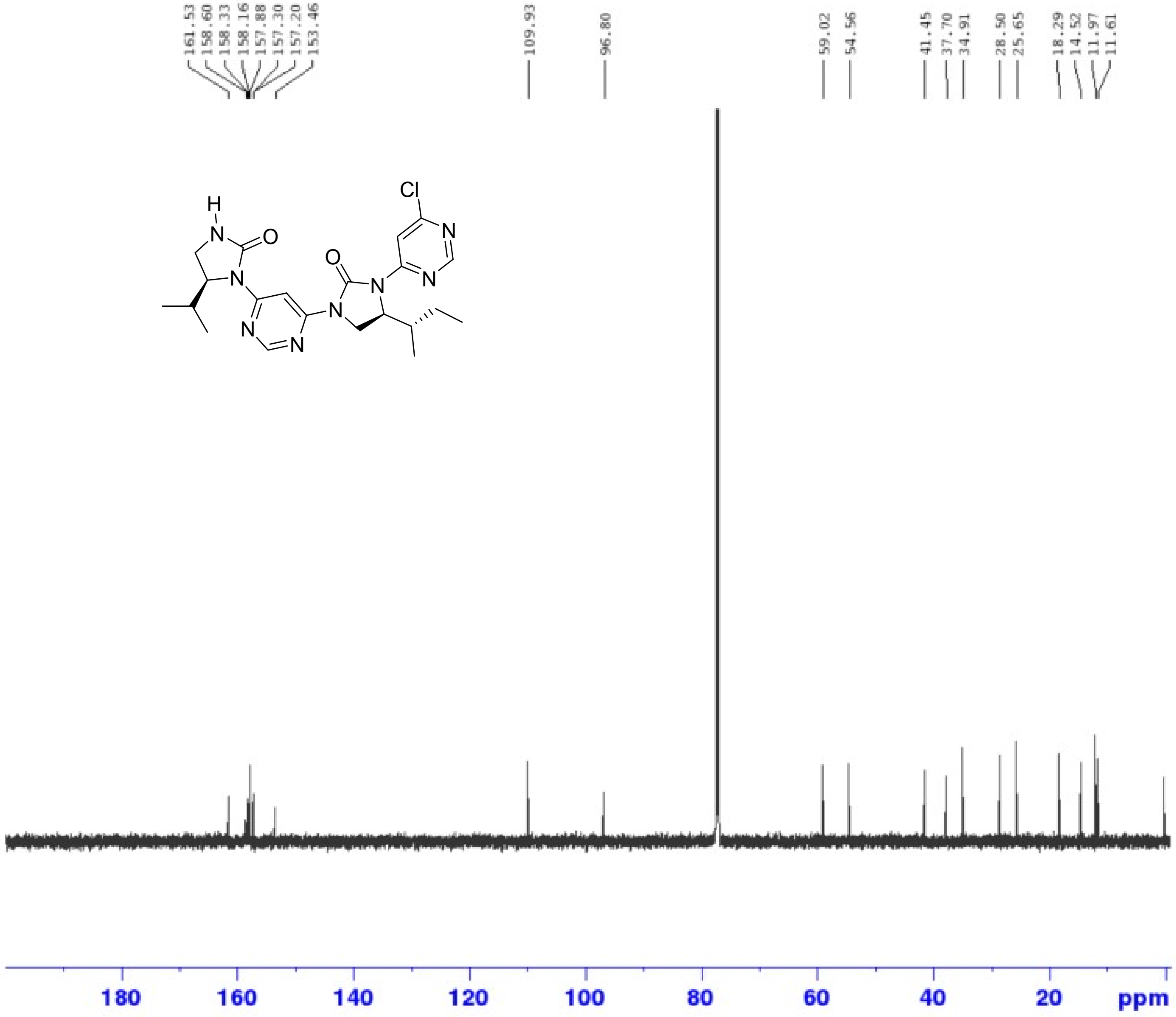

Current Data Parameters
NAME TW-A-185-A TRIT 600
EXPNO 11
PROCNO 1

F2 - Acquisition Parameters
Date_ 20190906
Time 23.03
INSTRUM spect
PROBHD 5 mm PABBO BB/
PULPROG zgpg30
TD 65536
SOLVENT CDCl3
NS 1024
DS 4
SWH 36057.691 Hz
FIDRES 0.550197 Hz
AQ 0.9087659 sec
RG 186.92
DW 13.867 usec
DE 6.50 usec
TE 300.0 K
D1 2.00000000 sec
D11 0.03000000 sec
TD0 1

======== CHANNEL f1 ========
SFO1 150.9178981 MHz
NUC1 13C
P1 11.80 usec
PLW1 85.00000000 W

======== CHANNEL f2 ========
SFO2 600.1324005 MHz
NUC2 1H
CPDPRG[2 waltz16
PCPD2 80.00 usec
PLW2 27.00000000 W
PLW12 0.43891999 W
PLW13 0.28090999 W

F2 - Processing parameters
SI 32768
SF 150.9027870 MHz
WDW EM
SSB 0
LB 1.00 Hz
GB 0
PC 1.40

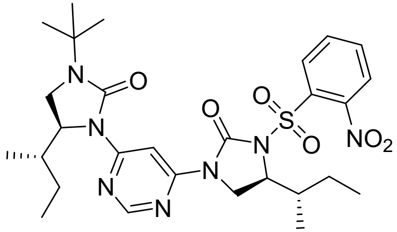

S23*

^{1}H NMR

600 MHz

$CDCl_3$

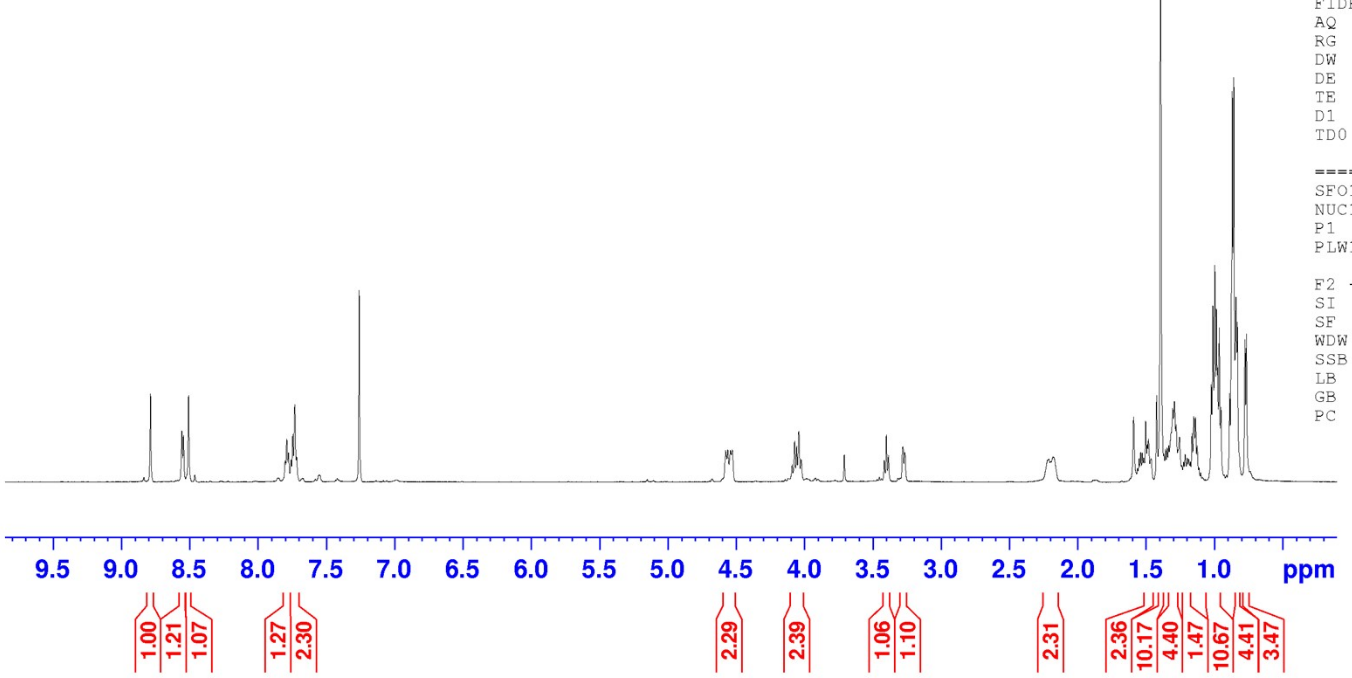


```
Current Data Parameters
NAME      TW-B-220-A TRIT 600
EXPNO                  10
PROCNO                 1

F2 - Acquisition Parameters
Date_             20190910
Time                  2.50
INSTRUM              spect
PROBHD    5 mm PABBO BB/
PULPROG               zg30
TD                   65536
SOLVENT              CDCl3
NS                      16
DS                       2
SWH            12019.230 Hz
FIDRES          0.183399 Hz
AQ             2.7262976 sec
RG                   119.2
DW                41.600 usec
DE                  6.50 usec
TE                 300.0 K
D1            1.00000000 sec
TD0                      1

======== CHANNEL f1 ========
SFO1         600.1337060 MHz
NUC1                    1H
P1                  10.00 usec
PLW1          26.60000038 W

F2 - Processing parameters
SI                   65536
SF           600.1300151 MHz
WDW                     EM
SSB                      0
LB                    0.30 Hz
GB                       0
PC                    1.00
```

* This compound was partially contaminated with hydrocarbon grease.

S23*

^{13}C NMR

151 MHz

$CDCl_3$

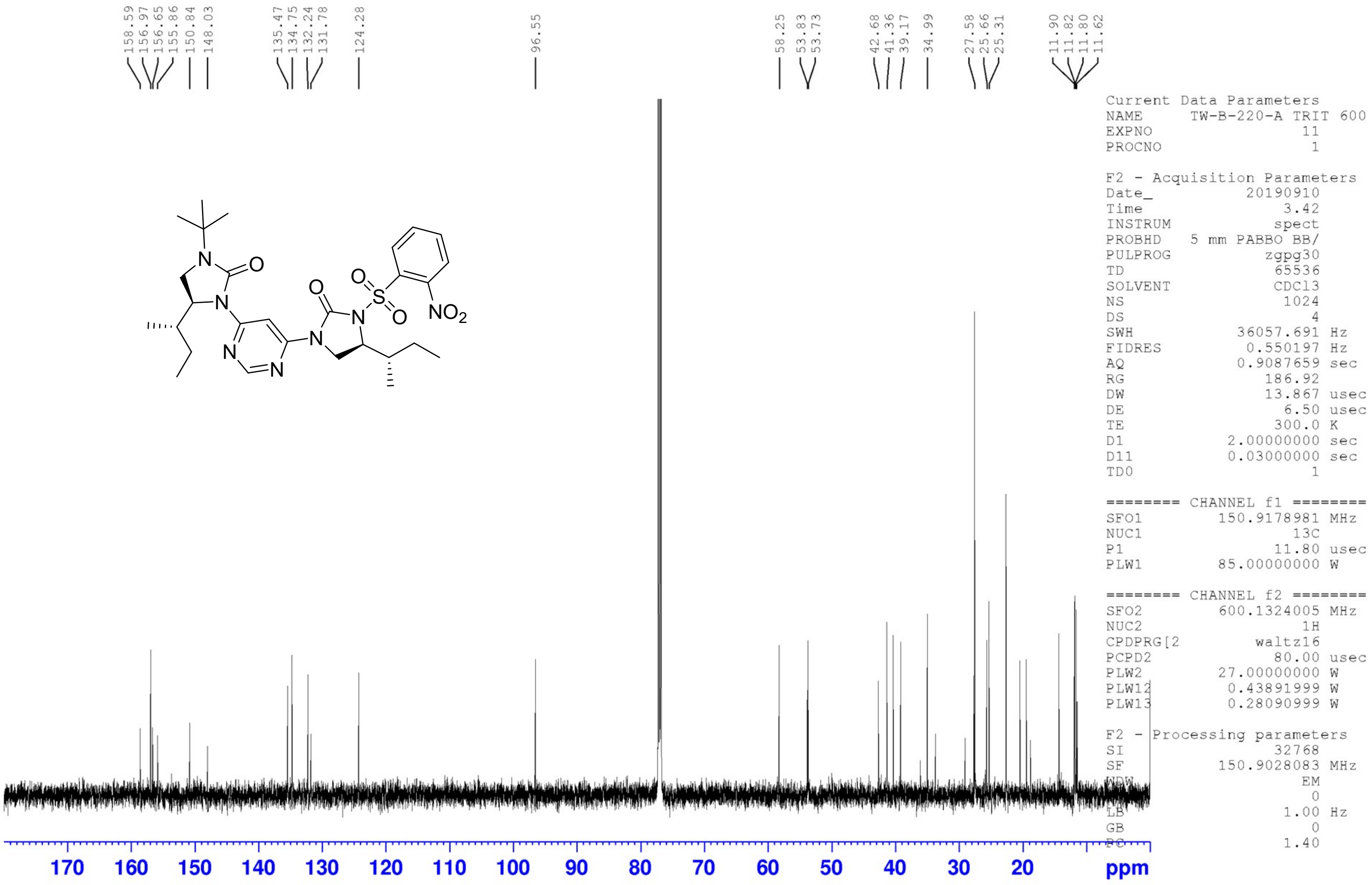

* This compound was partially contaminated with hydrocarbon grease.

S24

^{1}H NMR

600 MHz

$CDCl_3$

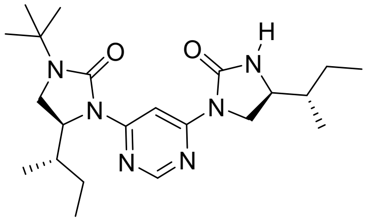

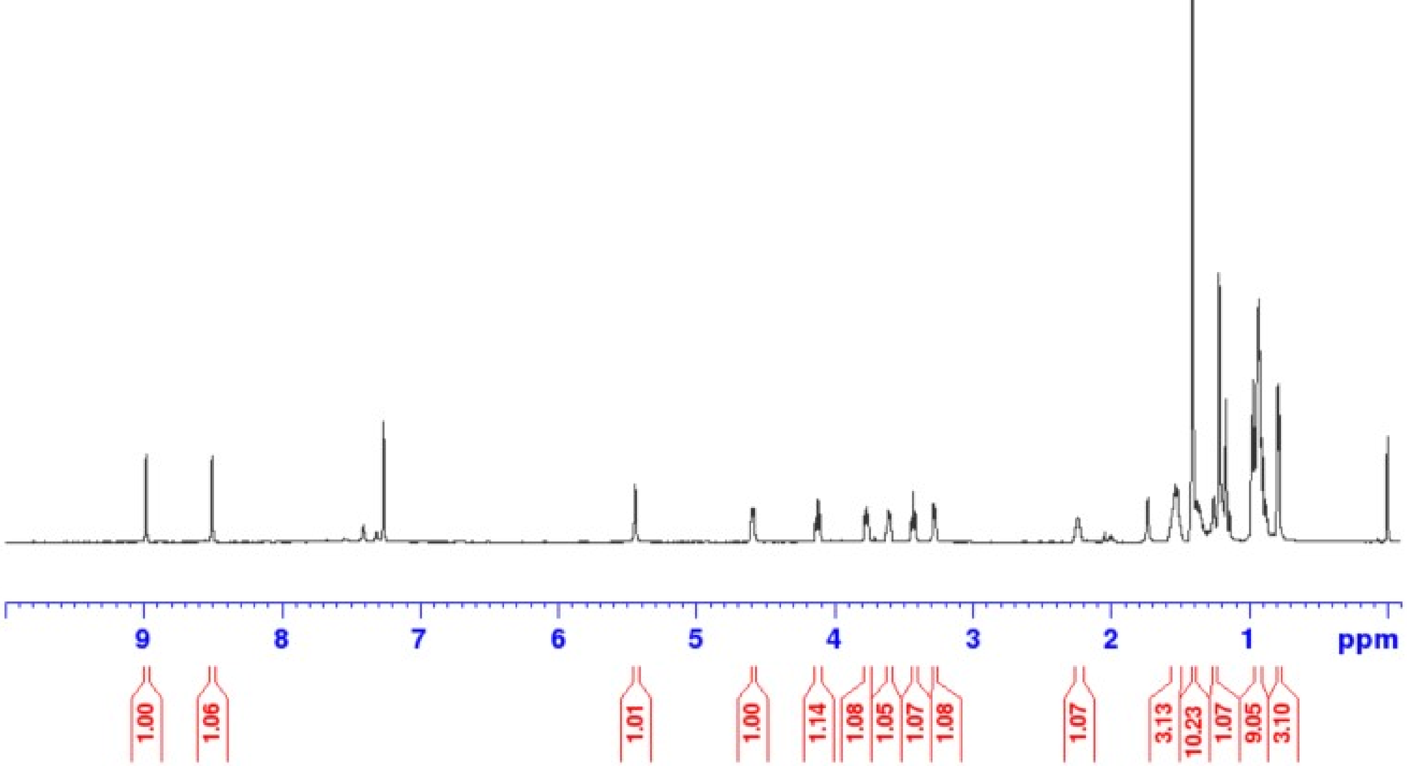


```
Current Data Parameters
NAME          TW-B-225 600
EXPNO                   90
PROCNO                   1

F2 - Acquisition Parameters
Date_             20190831
Time                 11.25
INSTRUM              spect
PROBHD     5 mm PABBO BB/
PULPROG               zg30
TD                   65536
SOLVENT              CDCl3
NS                      16
DS                       2
SWH            12019.230 Hz
FIDRES          0.183399 Hz
AQ             2.7262976 sec
RG                 105.21
DW               41.600 usec
DE                 6.50 usec
TE                300.0 K
D1           1.00000000 sec
TD0                      1

======== CHANNEL f1 ========
SFO1        600.1337060 MHz
NUC1                    1H
P1                10.00 usec
PLW1        26.60000038 W

F2 - Processing parameters
SI                   65536
SF          600.1300149 MHz
WDW                     EM
SSB                      0
LB                    0.30 Hz
GB                       0
PC                    1.00
```

S24

^{13}C NMR

151 MHz

$CDCl_3$

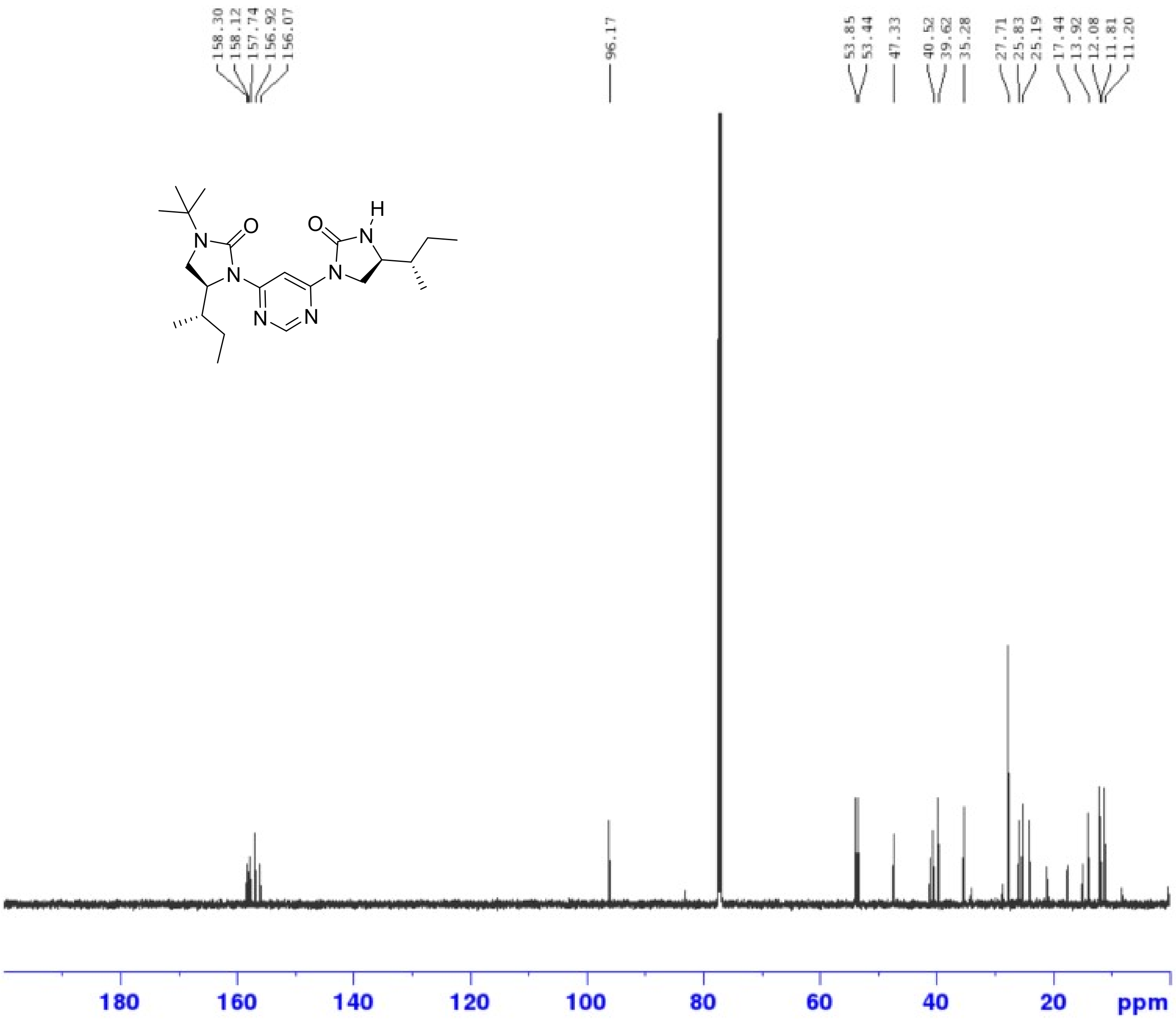

Current Data Parameters
NAME TW-B-225 600
EXPNO 92
PROCNO 1

F2 - Acquisition Parameters
Date_ 20190831
Time 12.16
INSTRUM spect
PROBHD 5 mm PABBO BB/
PULPROG zgpg30
TD 65536
SOLVENT CDCl3
NS 1024
DS 4
SWH 36057.691 Hz
FIDRES 0.550197 Hz
AQ 0.9087659 sec
RG 186.92
DW 13.867 usec
DE 6.50 usec
TE 300.0 K
D1 2.00000000 sec
D11 0.03000000 sec
TD0 1

======== CHANNEL f1 ========
SFO1 150.9178981 MHz
NUC1 13C
P1 11.80 usec
PLW1 85.00000000 W

======== CHANNEL f2 ========
SFO2 600.1324005 MHz
NUC2 1H
CPDPRG[2 waltz16
PCPD2 80.00 usec
PLW2 27.00000000 W
PLW12 0.43891999 W
PLW13 0.28090999 W

F2 - Processing parameters
SI 32768
SF 150.9027881 MHz
WDW EM
SSB 0
LB 1.00 Hz
GB 0
PC 1.40

1b

^1H NMR

600 MHz

$CDCl_3$

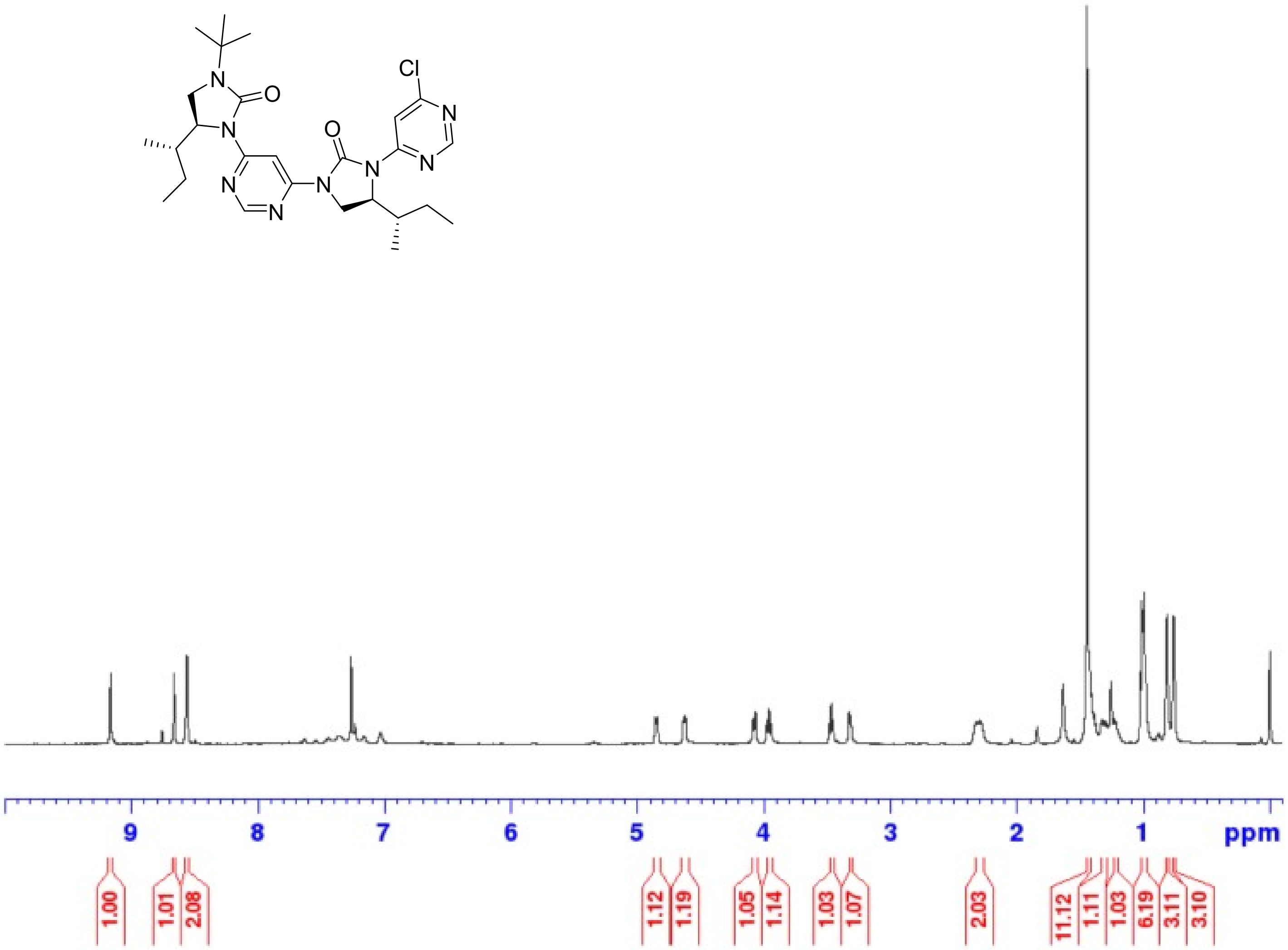


```
Current Data Parameters
NAME        TW-B-244-A 600
EXPNO                   10
PROCNO                   1

F2 - Acquisition Parameters
Date_             20190906
Time                  0.04
INSTRUM              spect
PROBHD     5 mm PABBO BB/
PULPROG               zg30
TD                   65536
SOLVENT              CDCl3
NS                      16
DS                       2
SWH              12019.230 Hz
FIDRES            0.183399 Hz
AQ               2.7262976 sec
RG                  105.21
DW                  41.600 usec
DE                    6.50 usec
TE                   300.0 K
D1              1.00000000 sec
TD0                      1

======== CHANNEL f1 ========
SFO1           600.1337060 MHz
NUC1                    1H
P1                   10.00 usec
PLW1           26.60000038 W

F2 - Processing parameters
SI                   65536
SF             600.1300152 MHz
WDW                     EM
SSB                      0
LB                    0.30 Hz
GB                       0
PC                    1.00
```

1b

^{13}C NMR

151 MHz

$CDCl_3$

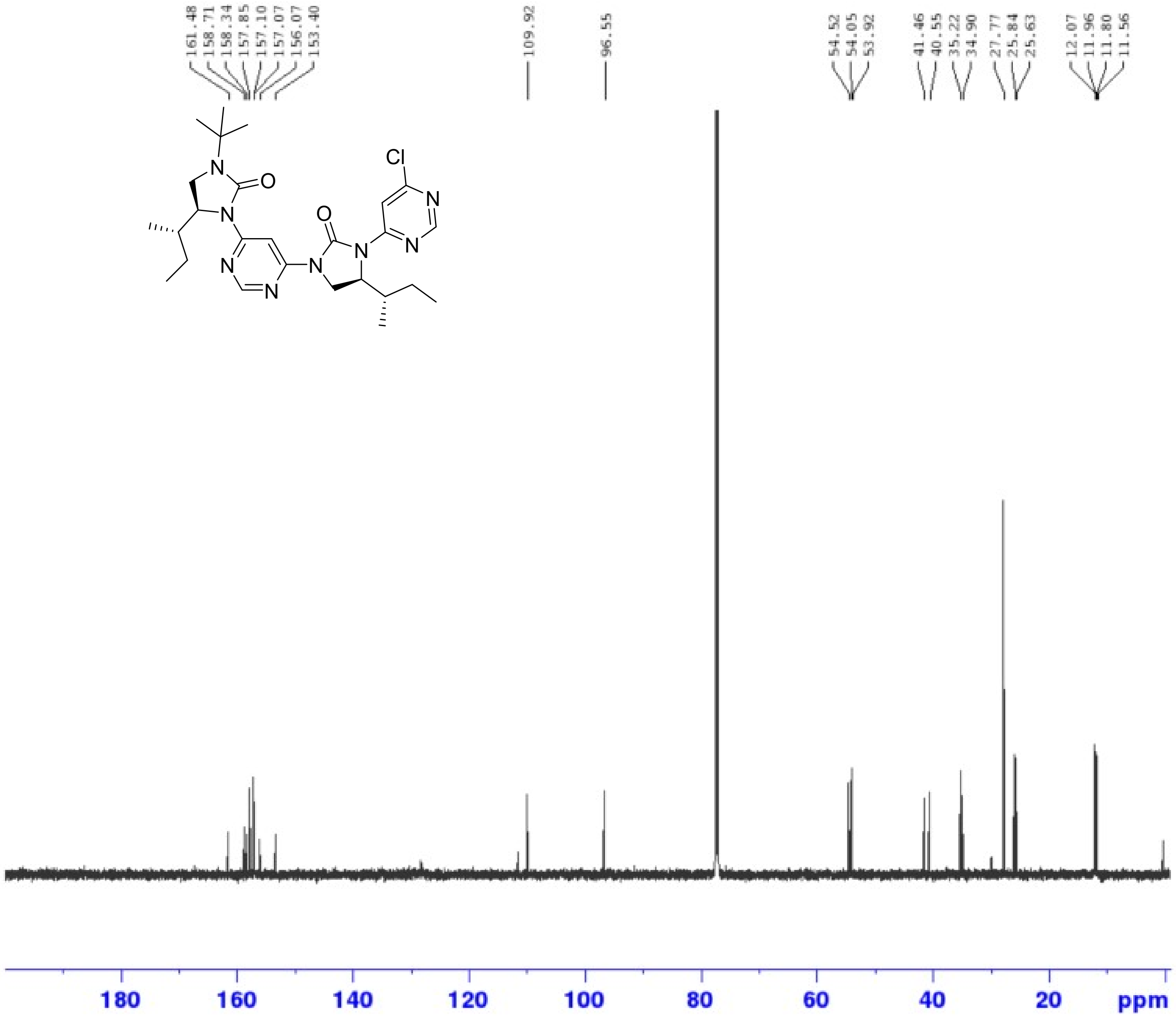


```
Current Data Parameters
NAME        TW-B-244-A 600
EXPNO                   12
PROCNO                   1

F2 - Acquisition Parameters
Date_             20190906
Time                  0.55
INSTRUM              spect
PROBHD     5 mm PABBO BB/
PULPROG            zgpg30
TD                   65536
SOLVENT              CDCl3
NS                    1024
DS                       4
SWH              36057.691 Hz
FIDRES            0.550197 Hz
AQ               0.9087659 sec
RG                  186.92
DW                  13.867 usec
DE                    6.50 usec
TE                   300.0 K
D1              2.00000000 sec
D11             0.03000000 sec
TD0                      1

======== CHANNEL f1 ========
SFO1           150.9178981 MHz
NUC1                   13C
P1                   11.80 usec
PLW1           85.00000000 W

======== CHANNEL f2 ========
SFO2           600.1324005 MHz
NUC2                    1H
CPDPRG[2           waltz16
PCPD2                80.00 usec
PLW2           27.00000000 W
PLW12           0.43891999 W
PLW13           0.28090999 W

F2 - Processing parameters
SI                   32768
SF             150.9027883 MHz
WDW                     EM
SSB                      0
LB                    1.00 Hz
GB                       0
PC                    1.40
```

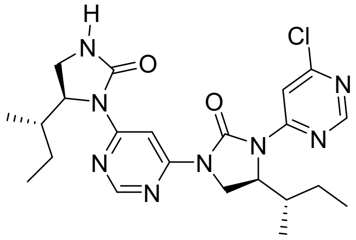

2b

^{1}H NMR

600 MHz

$CDCl_3$

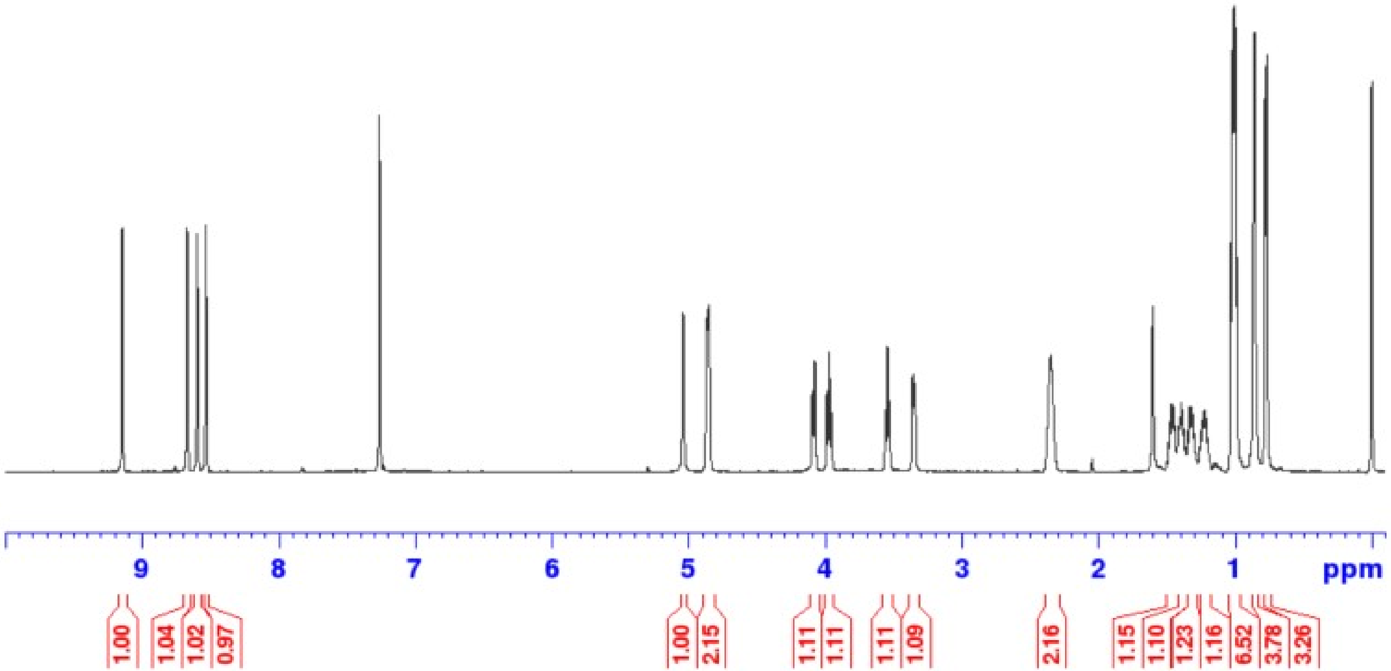


```
Current Data Parameters
NAME          TW-B-248 600
EXPNO                  100
PROCNO                   1

F2 - Acquisition Parameters
Date_             20190831
Time                 13.36
INSTRUM              spect
PROBHD     5 mm PABBO BB/
PULPROG               zg30
TD                   65536
SOLVENT              CDCl3
NS                      16
DS                       2
SWH             12019.230 Hz
FIDRES           0.183399 Hz
AQ              2.7262976 sec
RG                  168.12
DW                  41.600 usec
DE                    6.50 usec
TE                   300.0 K
D1              1.00000000 sec
TD0                      1

======== CHANNEL f1 ========
SFO1           600.1337060 MHz
NUC1                    1H
P1                   10.00 usec
PLW1           26.60000038 W

F2 - Processing parameters
SI                   65536
SF             600.1300153 MHz
WDW                     EM
SSB                      0
LB                    0.30 Hz
GB                       0
PC                    1.00
```

2b

^{13}C NMR

151 MHz

$CDCl_3$

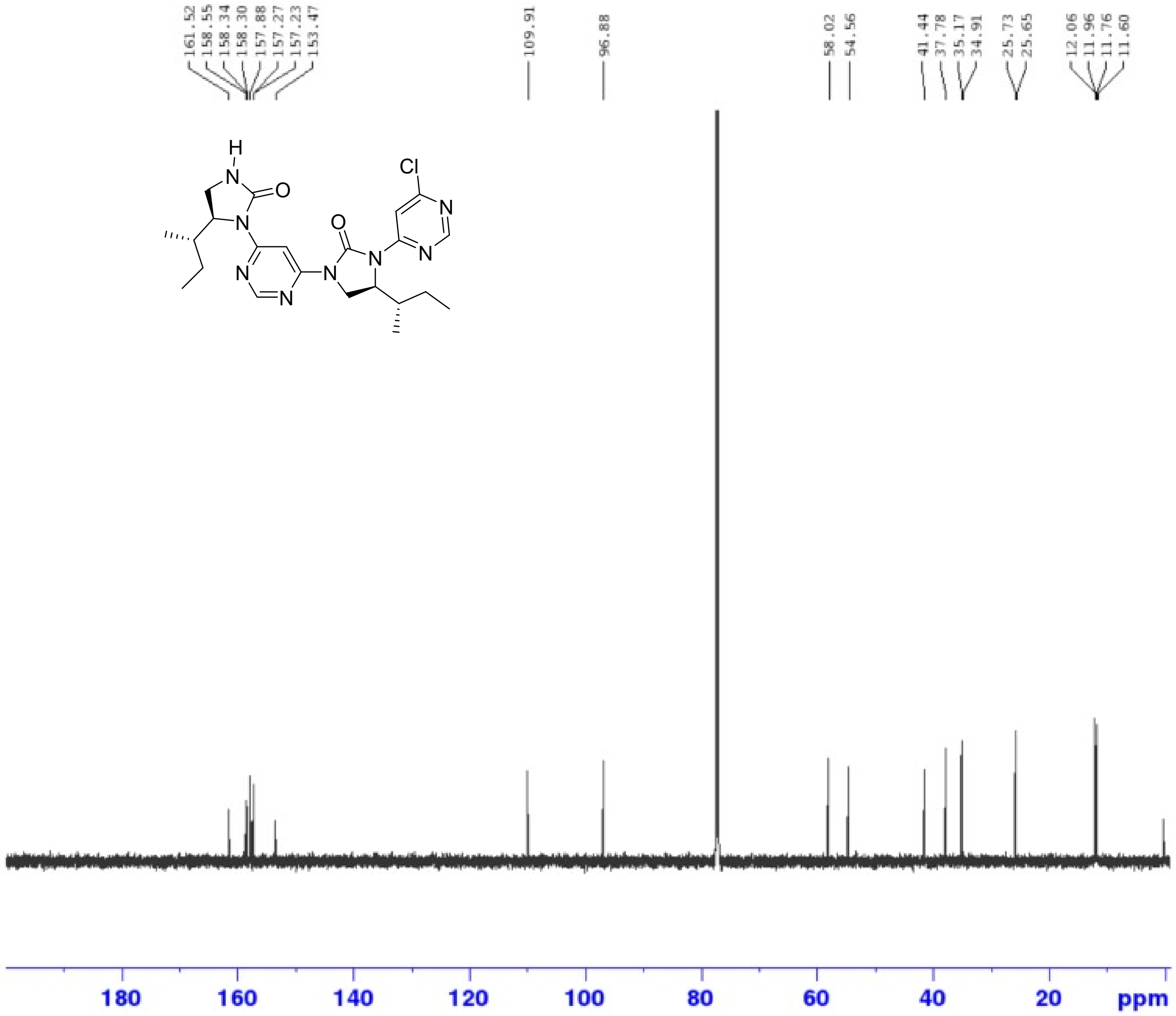

Current Data Parameters
NAME TW-B-248 600
EXPNO 102
PROCNO 1

F2 - Acquisition Parameters
Date_ 20190831
Time 14.27
INSTRUM spect
PROBHD 5 mm PABBO BB/
PULPROG zgpg30
TD 65536
SOLVENT CDCl3
NS 1024
DS 4
SWH 36057.691 Hz
FIDRES 0.550197 Hz
AQ 0.9087659 sec
RG 186.92
DW 13.867 usec
DE 6.50 usec
TE 300.0 K
D1 2.00000000 sec
D11 0.03000000 sec
TD0 1

======== CHANNEL f1 ========
SFO1 150.9178981 MHz
NUC1 13C
P1 11.80 usec
PLW1 85.00000000 W

======== CHANNEL f2 ========
SFO2 600.1324005 MHz
NUC2 1H
CPDPRG[2 waltz16
PCPD2 80.00 usec
PLW2 27.00000000 W
PLW12 0.43891999 W
PLW13 0.28090999 W

F2 - Processing parameters
SI 32768
SF 150.9027873 MHz
WDW EM
SSB 0
LB 1.00 Hz
GB 0
PC 1.40

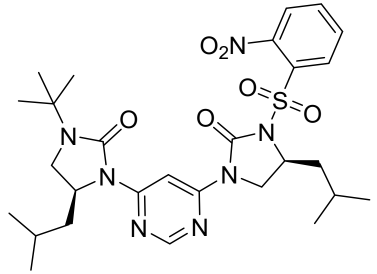

S25

^1H NMR

600 MHz

$CDCl_3$

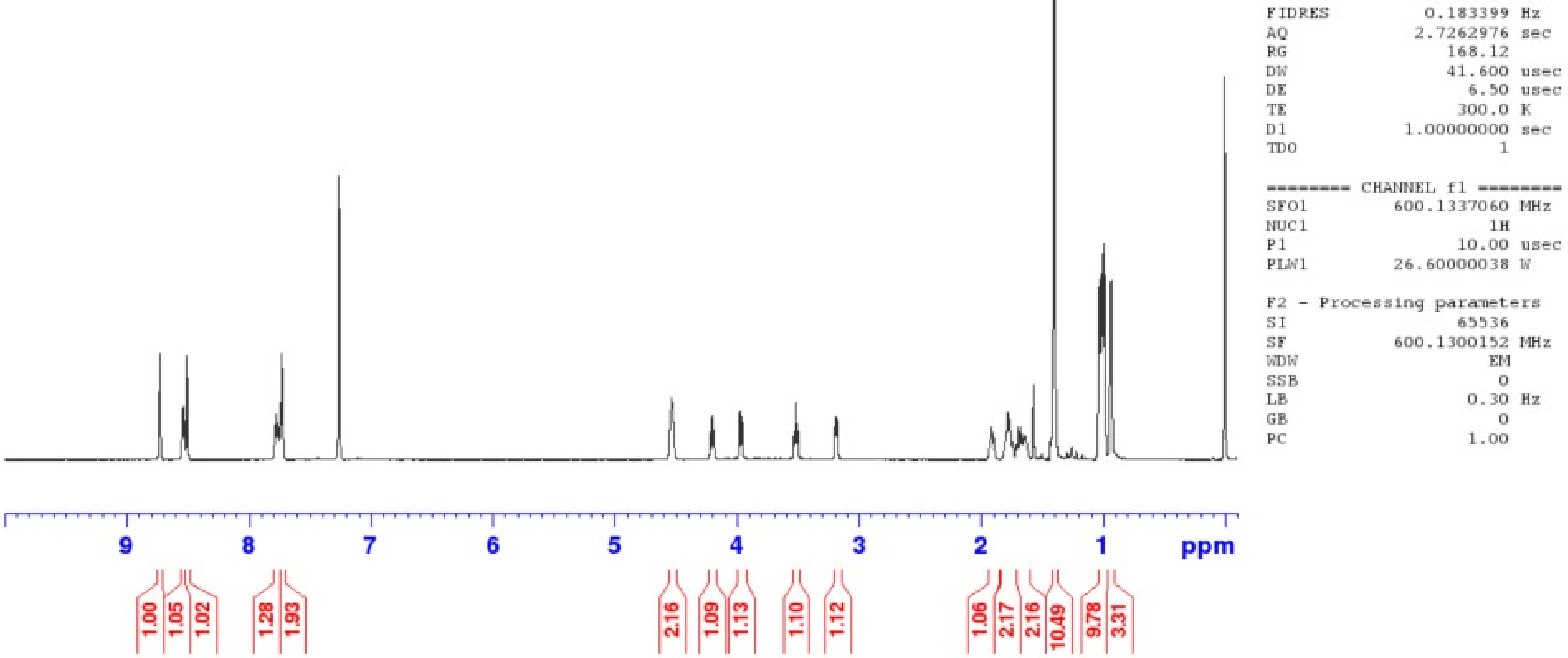

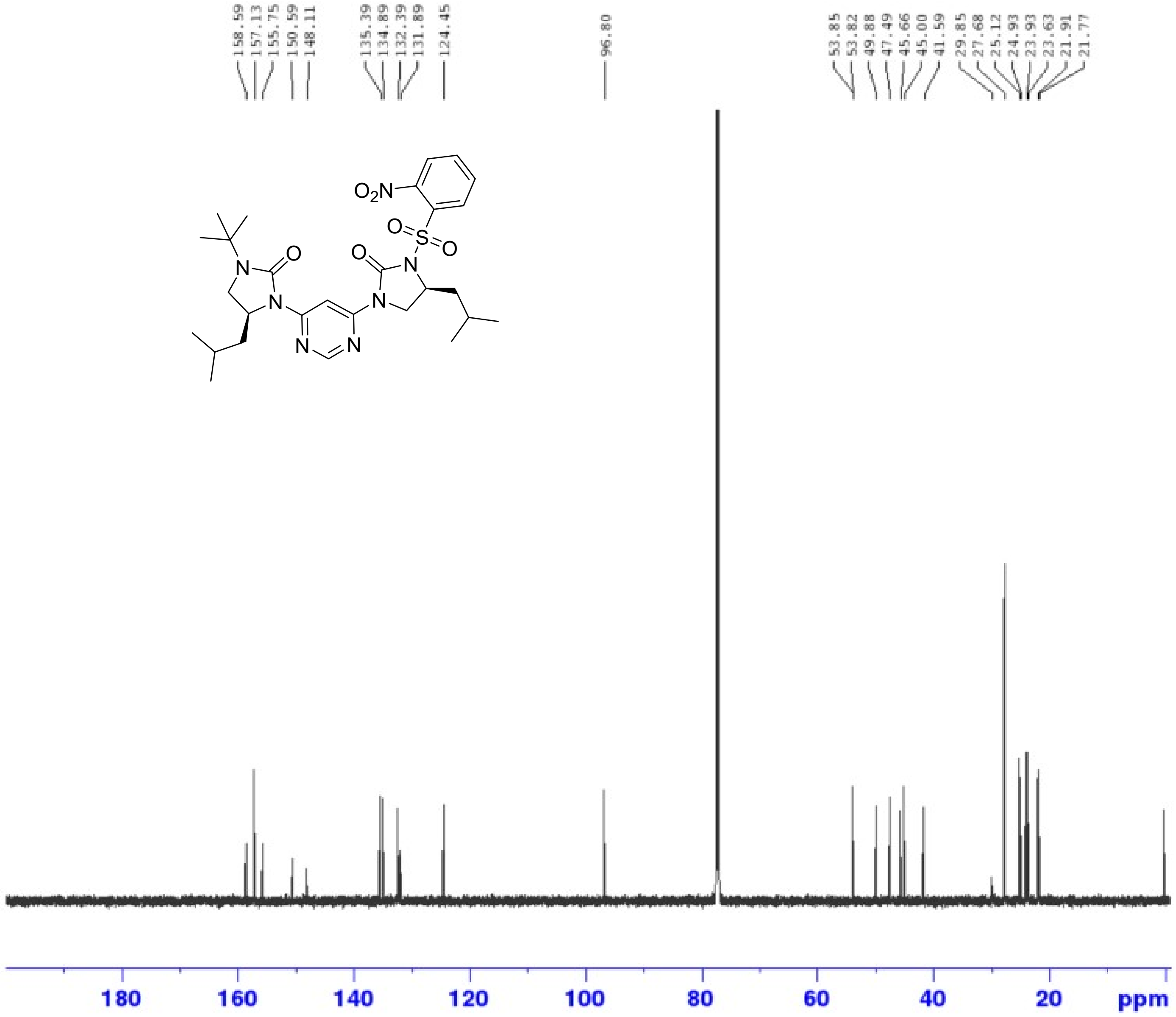

S25

^{13}C NMR

151 MHz

$CDCl_3$

```
Current Data Parameters
NAME      TW-A-144 CARBON
EXPNO                  10
PROCNO                  1

F2 - Acquisition Parameters
Date_            20190907
Time                22.26
INSTRUM             spect
PROBHD    5 mm PABBO BB/
PULPROG           zgpg30
TD                 65536
SOLVENT            CDCl3
NS                  4092
DS                     4
SWH           36057.691 Hz
FIDRES         0.550197 Hz
AQ            0.9087659 sec
RG               186.92
DW               13.867 usec
DE                 6.50 usec
TE                300.0 K
D1           2.00000000 sec
D11          0.03000000 sec
TD0                    1

======== CHANNEL f1 ========
SFO1        150.9178981 MHz
NUC1                 13C
P1                 11.80 usec
PLW1         85.00000000 W

======== CHANNEL f2 ========
SFO2        600.1324005 MHz
NUC2                  1H
CPDPRG[2         waltz16
PCPD2              80.00 usec
PLW2         27.00000000 W
PLW12         0.43891999 W
PLW13         0.28090999 W

F2 - Processing parameters
SI                 32768
SF          150.9027867 MHz
WDW                   EM
SSB                    0
LB                  1.00 Hz
GB                     0
PC                  1.40
```

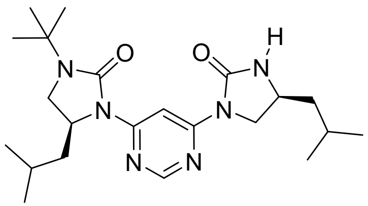

S26

^{1}H NMR

400 MHz

$CDCl_3$

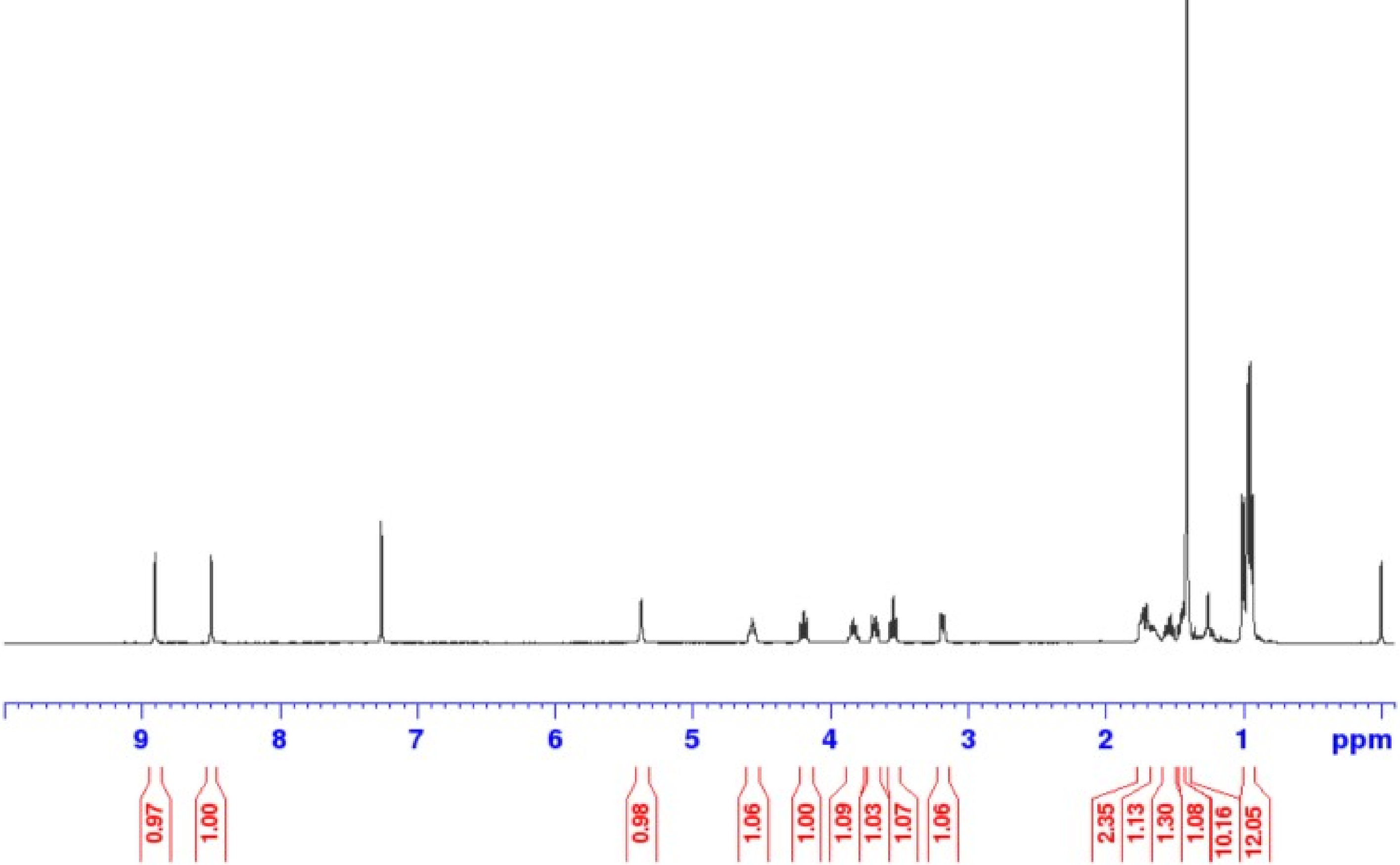


```
Current Data Parameters
NAME        TW-A-146-A RERUN
EXPNO                     50
PROCNO                     1

F2 - Acquisition Parameters
Date_               20190425
Time                   12.45
INSTRUM                spect
PROBHD    5 mm PABBO BB/
PULPROG                 zg30
TD                    131072
SOLVENT                CDCl3
NS                        64
DS                         0
SWH                12019.230 Hz
FIDRES              0.091699 Hz
AQ                 5.4525952 sec
RG                       203
DW                    41.600 usec
DE                      9.85 usec
TE                     300.0 K
D1                0.10000000 sec
TD0                        1

======== CHANNEL f1 ========
SFO1           399.9024695 MHz
NUC1                    1H
P1                    14.88 usec
PLW1             7.59999990 W

F2 - Processing parameters
SI                    131072
SF             399.9000097 MHz
WDW                       EM
SSB                        0
LB                      0.10 Hz
GB                         0
PC                      1.00
```

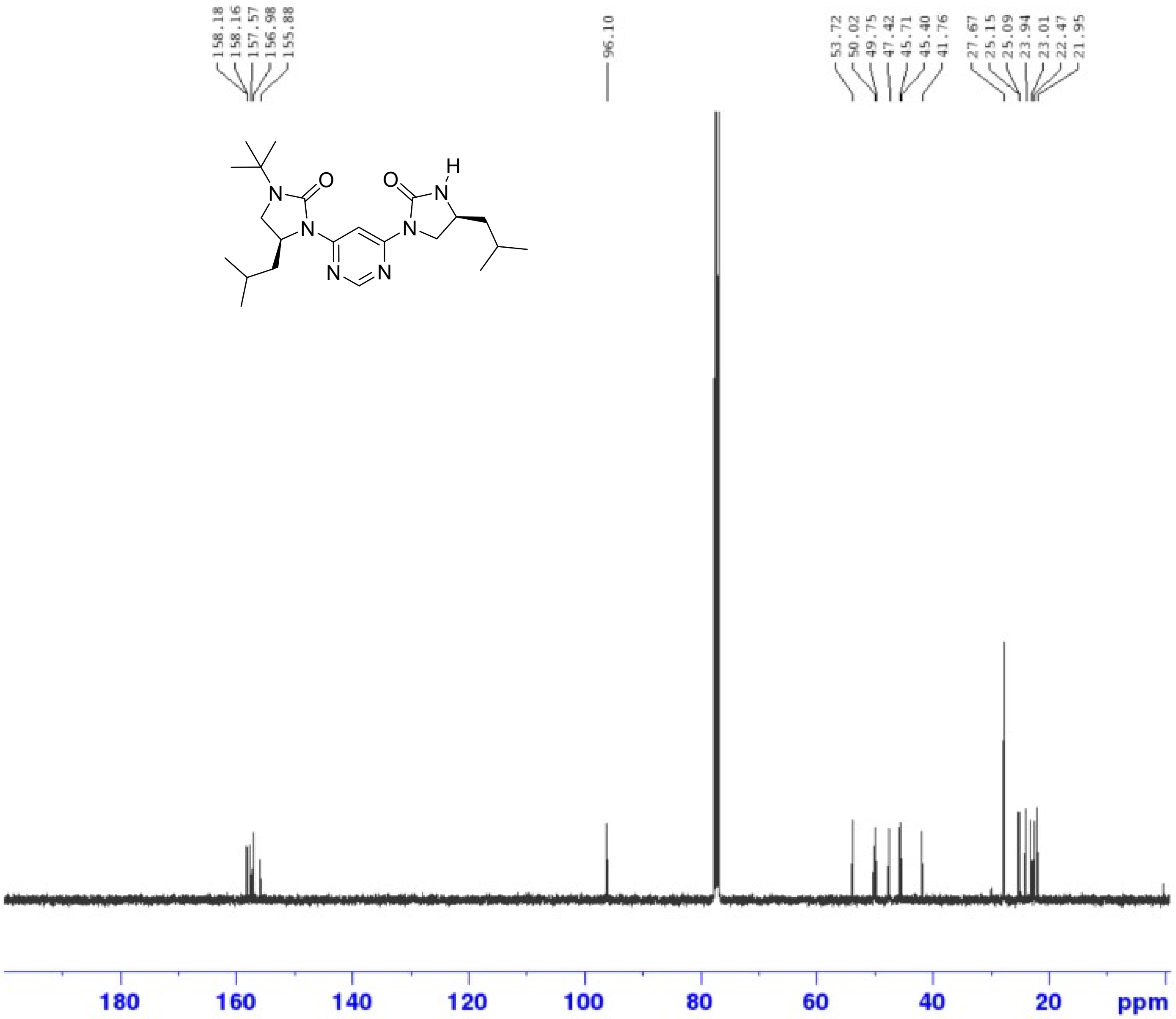

S26

^{13}C NMR

101 MHz

$CDCl_3$

Current Data Parameters
NAME TW-A-146-A RERUN
EXPNO 52
PROCNO 1

F2 - Acquisition Parameters
Date_ 20190426
Time 7.50
INSTRUM spect
PROBHD 5 mm PABBO BB/
PULPROG zgpg30
TD 65536
SOLVENT CDCl3
NS 1024
DS 4
SWH 24038.461 Hz
FIDRES 0.366798 Hz
AQ 1.3631488 sec
RG 203
DW 20.800 usec
DE 6.50 usec
TE 300.0 K
D1 2.00000000 sec
D11 0.03000000 sec
TD0 1

======== CHANNEL f1 ========
SFO1 100.5649900 MHz
NUC1 13C
P1 10.00 usec
PLW1 44.46300125 W

======== CHANNEL f2 ========
SFO2 399.9015996 MHz
NUC2 1H
CPDPRG[2 waltz16
PCPD2 90.00 usec
PLW2 7.59999990 W
PLW12 0.20774999 W
PLW13 0.16827001 W

F2 - Processing parameters
SI 32768
SF 100.5549210 MHz
WDW EM
SSB 0
LB 1.00 Hz
GB 0
PC 1.40

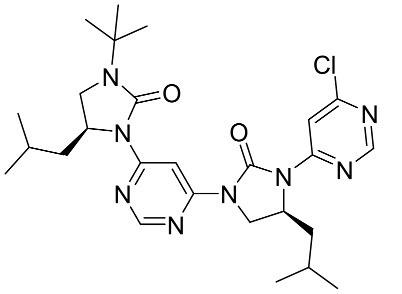

1c

^{1}H NMR

600 MHz

$CDCl_3$

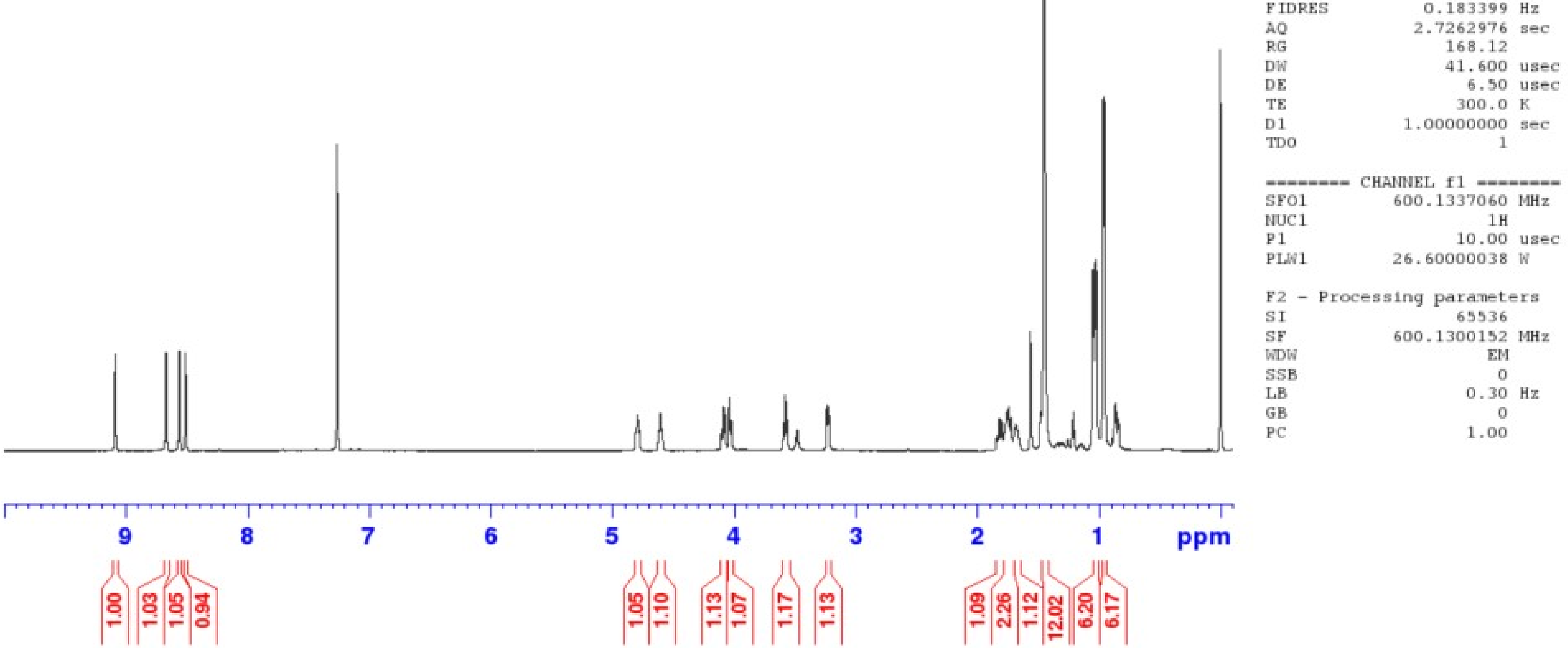

1c

^{13}C NMR

151 MHz

$CDCl_3$

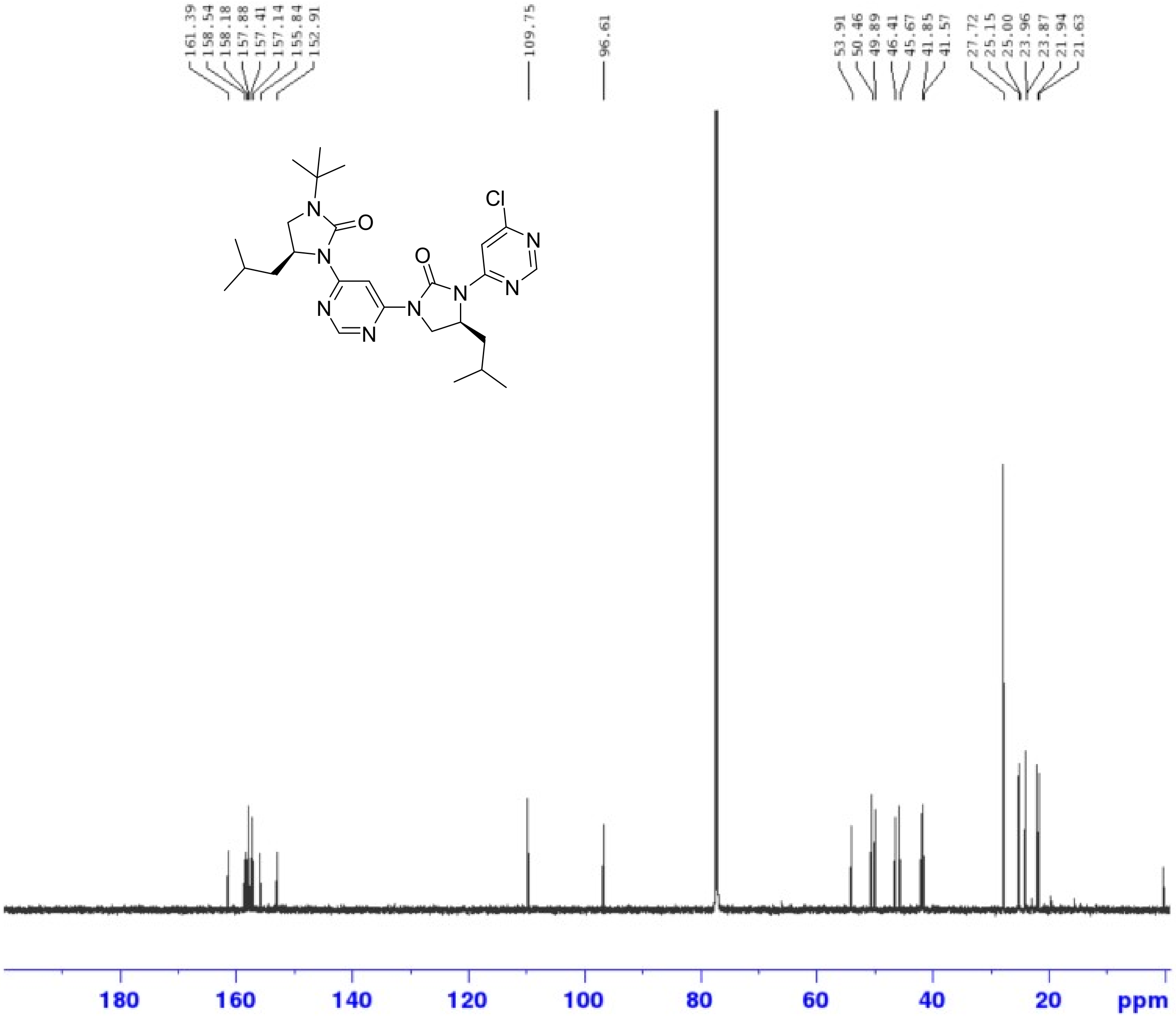


```
Current Data Parameters
NAME      TW-A-108 CARBON
EXPNO                  10
PROCNO                  1

F2 - Acquisition Parameters
Date_            20190910
Time                21.25
INSTRUM             spect
PROBHD   5 mm PABBO BB/
PULPROG           zgpg30
TD                 65536
SOLVENT            CDCl3
NS                  2048
DS                     4
SWH            36057.691 Hz
FIDRES          0.550197 Hz
AQ             0.9087659 sec
RG                186.92
DW                13.867 usec
DE                  6.50 usec
TE                 300.0 K
D1            2.00000000 sec
D11           0.03000000 sec
TD0                    1

======== CHANNEL f1 ========
SFO1         150.9178981 MHz
NUC1                 13C
P1                 11.80 usec
PLW1         85.00000000 W

======== CHANNEL f2 ========
SFO2         600.1324005 MHz
NUC2                  1H
CPDPRG[2         waltz16
PCPD2              80.00 usec
PLW2         27.00000000 W
PLW12         0.43891999 W
PLW13         0.28090999 W

F2 - Processing parameters
SI                 32768
SF           150.9027883 MHz
WDW                   EM
SSB                    0
LB                  1.00 Hz
GB                     0
PC                  1.40
```

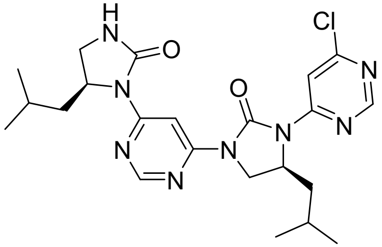

2c

^{1}H NMR

600 MHz

$CDCl_3$

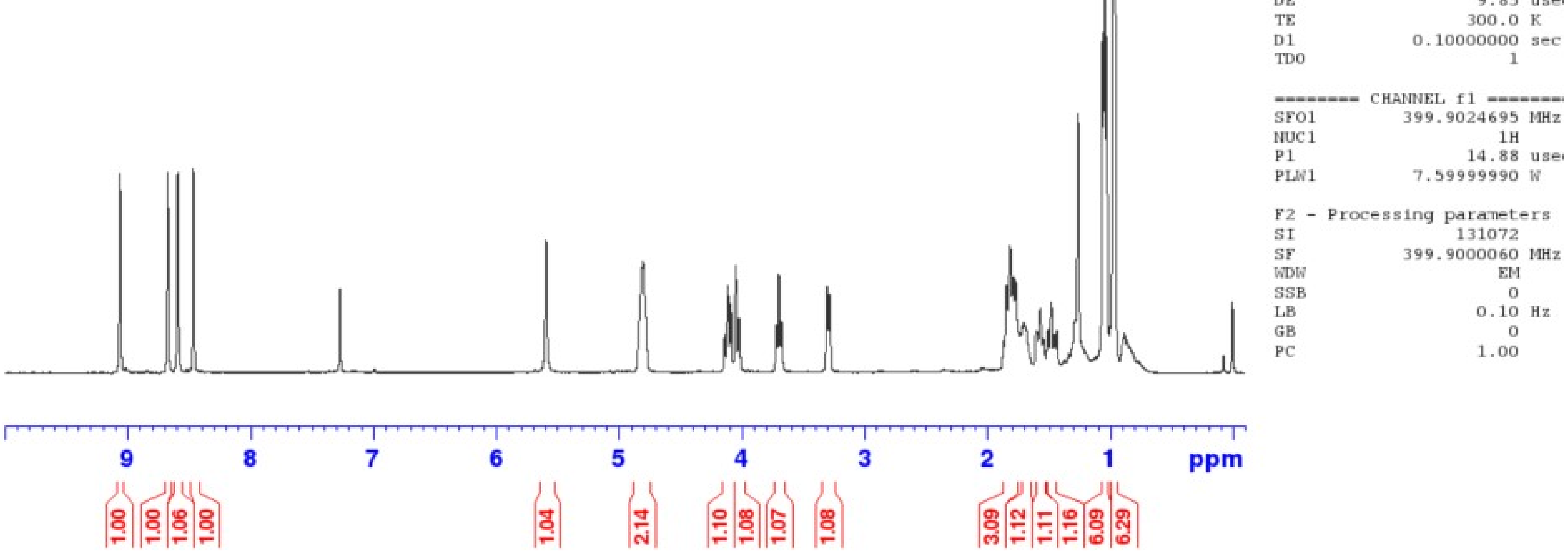

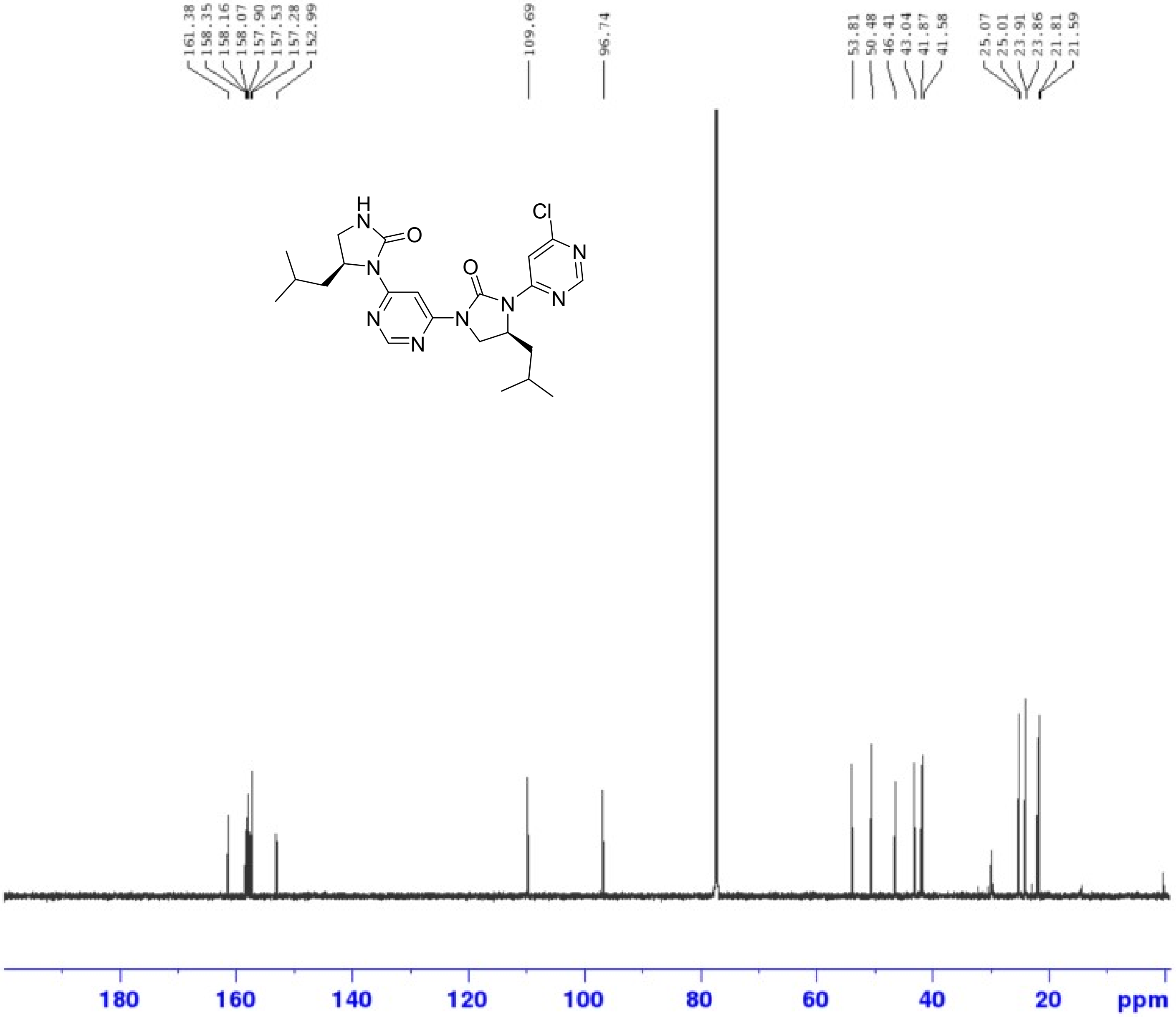

2c

^{13}C NMR

151 MHz

$CDCl_3$

```
Current Data Parameters
NAME          TW-B-164 600
EXPNO                   11
PROCNO                   1

F2 - Acquisition Parameters
Date_             20190904
Time                  7.36
INSTRUM              spect
PROBHD    5 mm PABBO BB/
PULPROG             zgpg30
TD                   65536
SOLVENT              CDCl3
NS                    1024
DS                       4
SWH             36057.691 Hz
FIDRES           0.550197 Hz
AQ              0.9087659 sec
RG                  186.92
DW                  13.867 usec
DE                    6.50 usec
TE                   300.0 K
D1              2.00000000 sec
D11             0.03000000 sec
TD0                      1

======== CHANNEL f1 ========
SFO1          150.9178981 MHz
NUC1                   13C
P1                   11.80 usec
PLW1           85.00000000 W

======== CHANNEL f2 ========
SFO2          600.1324005 MHz
NUC2                    1H
CPDPRG[2           waltz16
PCPD2                80.00 usec
PLW2           27.00000000 W
PLW12           0.43891999 W
PLW13           0.28090999 W

F2 - Processing parameters
SI                   32768
SF            150.9027897 MHz
WDW                     EM
SSB                      0
LB                    1.00 Hz
GB                       0
PC                    1.40
```

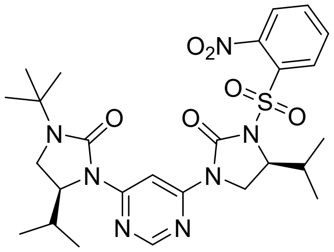

S27

^1H NMR

600 MHz

$CDCl_3$

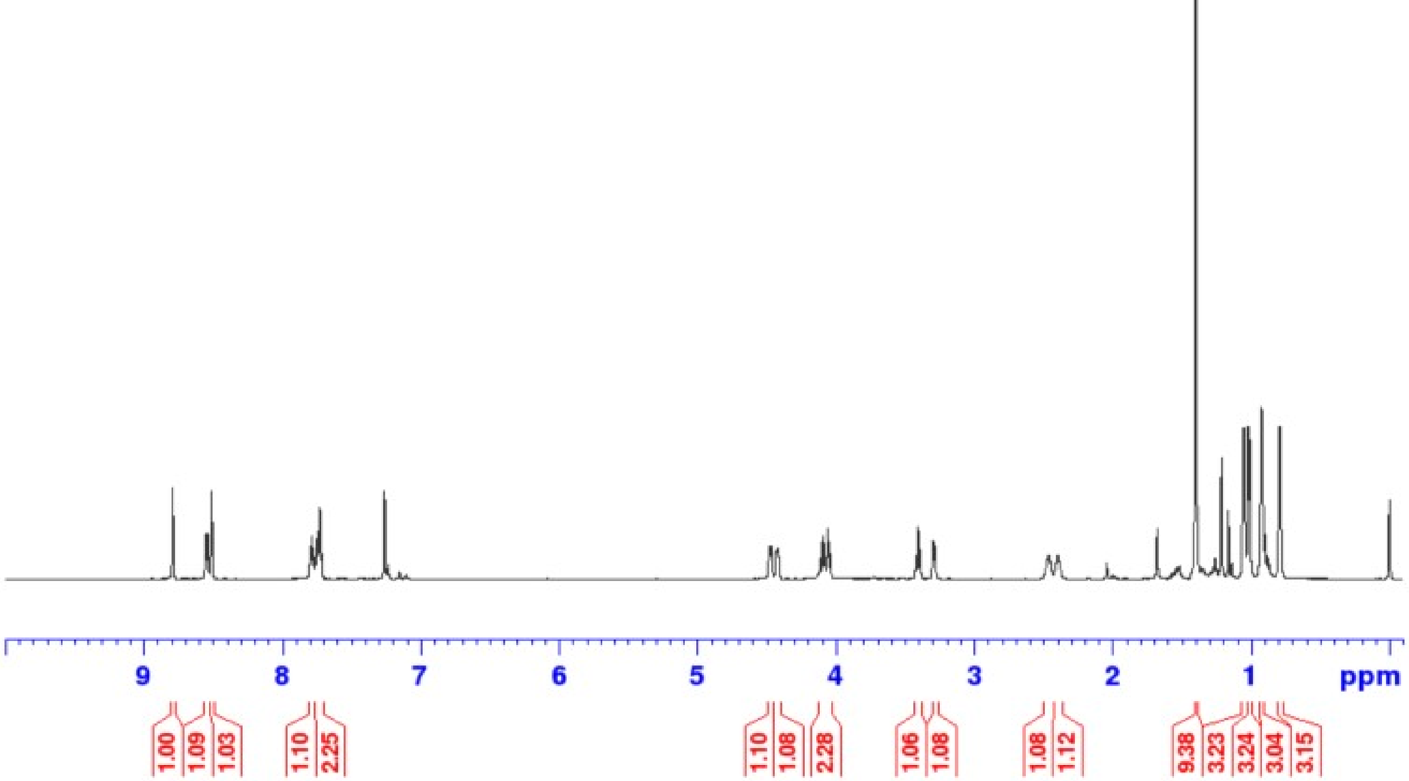


```
Current Data Parameters
NAME        TW-B-231-A 600
EXPNO                   40
PROCNO                   1

F2 - Acquisition Parameters
Date_             20190830
Time                 21.07
INSTRUM              spect
PROBHD    5 mm PABBO BB/
PULPROG               zg30
TD                   65536
SOLVENT              CDCl3
NS                      16
DS                       2
SWH              12019.230 Hz
FIDRES            0.183399 Hz
AQ               2.7262976 sec
RG                  105.21
DW                  41.600 usec
DE                    6.50 usec
TE                   300.0 K
D1              1.00000000 sec
TD0                      1

======== CHANNEL f1 ========
SFO1        600.1337060 MHz
NUC1                 1H
P1                10.00 usec
PLW1        26.60000038 W

F2 - Processing parameters
SI                   65536
SF             600.1300155 MHz
WDW                     EM
SSB                      0
LB                    0.30 Hz
GB                       0
PC                    1.00
```

S27

^{13}C NMR

151 MHz

$CDCl_3$

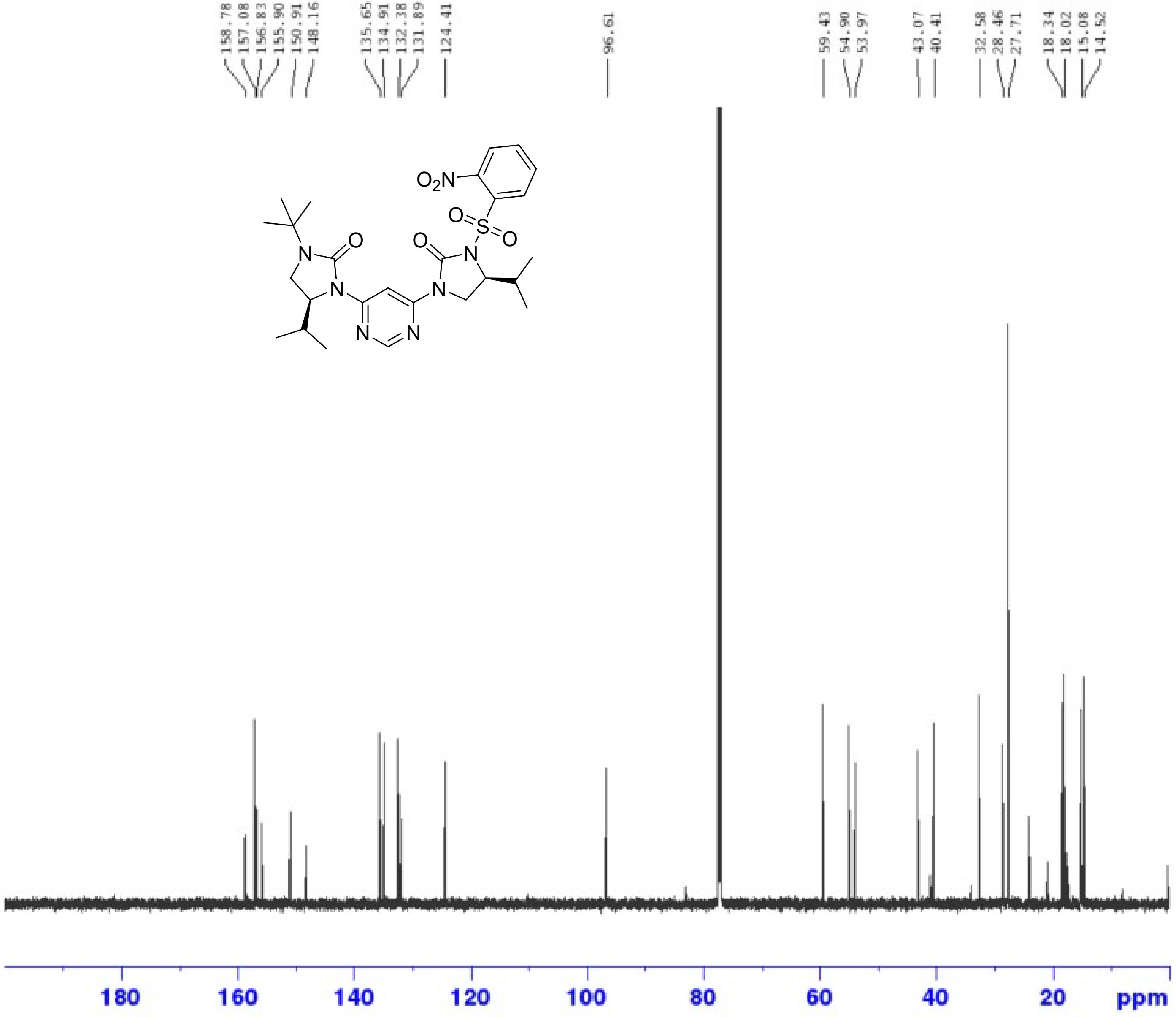


```
Current Data Parameters
NAME        TW-B-231-A 600
EXPNO                   42
PROCNO                   1

F2 - Acquisition Parameters
Date_             20190830
Time                 21.59
INSTRUM              spect
PROBHD     5 mm PABBO BB/
PULPROG             zgpg30
TD                   65536
SOLVENT              CDCl3
NS                    1024
DS                       4
SWH              36057.691 Hz
FIDRES            0.550197 Hz
AQ               0.9087659 sec
RG                  186.92
DW                  13.867 usec
DE                    6.50 usec
TE                   300.0 K
D1              2.00000000 sec
D11             0.03000000 sec
TD0                      1

======== CHANNEL f1 ========
SFO1          150.9178981 MHz
NUC1                  13C
P1                  11.80 usec
PLW1          85.00000000 W

======== CHANNEL f2 ========
SFO2          600.1324005 MHz
NUC2                   1H
CPDPRG[2             waltz16
PCPD2               80.00 usec
PLW2          27.00000000 W
PLW12          0.43891999 W
PLW13          0.28090999 W

F2 - Processing parameters
SI                   32768
SF            150.9027885 MHz
WDW                     EM
SSB                      0
LB                    1.00 Hz
GB                       0
PC                    1.40
```

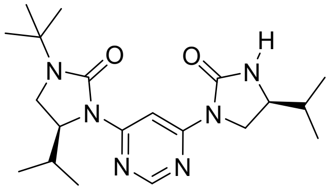

S28

^{1}H NMR

400 MHz

$CDCl_3$

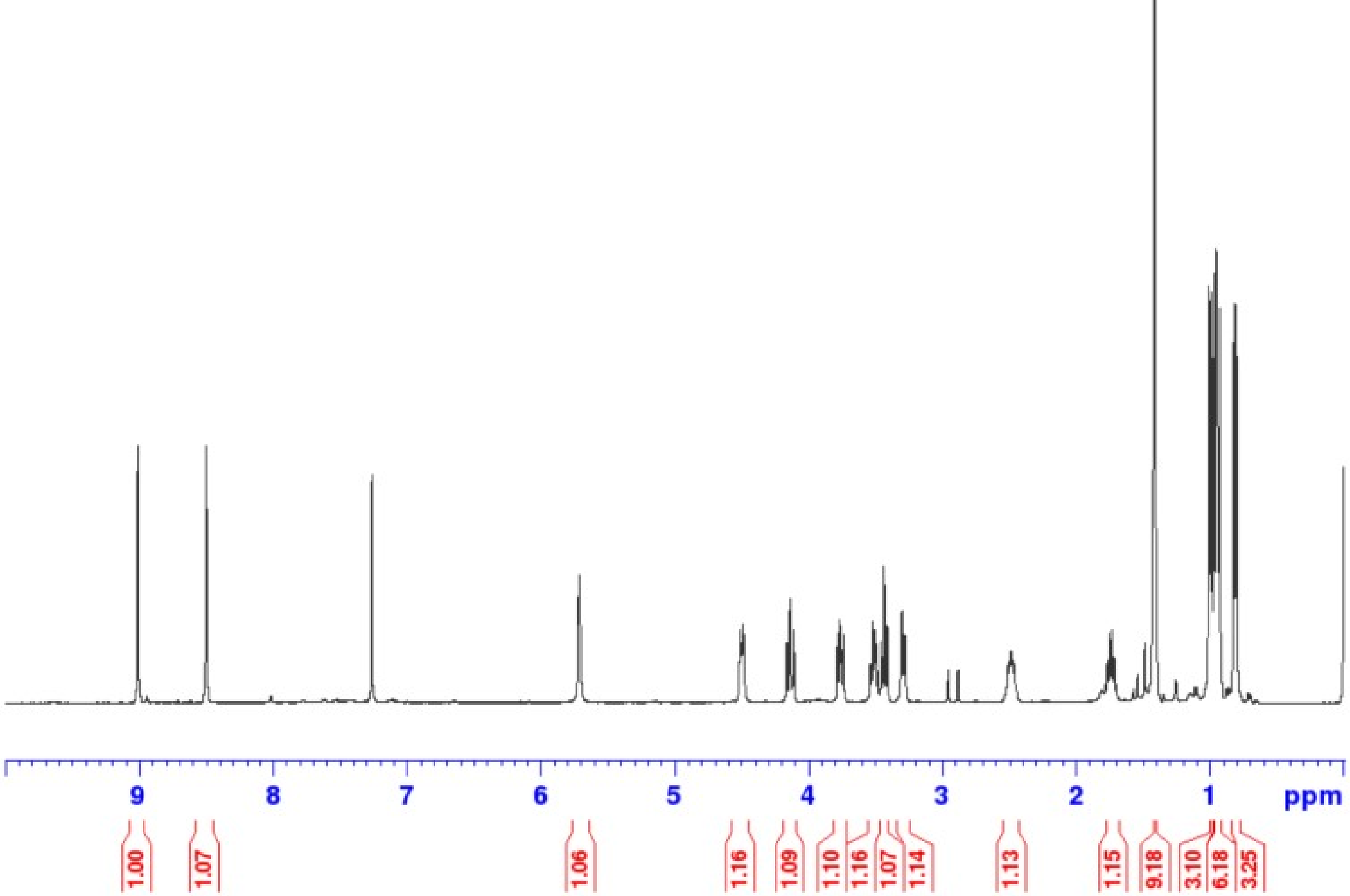


```
Current Data Parameters
NAME        TW-A-176-A RERUN
EXPNO                     90
PROCNO                     1

F2 - Acquisition Parameters
Date_               20190427
Time                   21.19
INSTRUM                spect
PROBHD      5 mm PABBO BB/
PULPROG                 zg30
TD                    131072
SOLVENT                CDCl3
NS                        64
DS                         0
SWH              12019.230 Hz
FIDRES            0.091699 Hz
AQ               5.4525952 sec
RG                       101
DW                    41.600 usec
DE                      9.85 usec
TE                     300.0 K
D1               0.10000000 sec
TD0                        1

======== CHANNEL f1 ========
SFO1           399.9024695 MHz
NUC1                      1H
P1                     14.88 usec
PLW1              7.59999990 W

F2 - Processing parameters
SI                    131072
SF             399.9000096 MHz
WDW                       EM
SSB                        0
LB                      0.10 Hz
GB                         0
PC                      1.00
```

S28

^{13}C NMR

101 MHz

$CDCl_3$

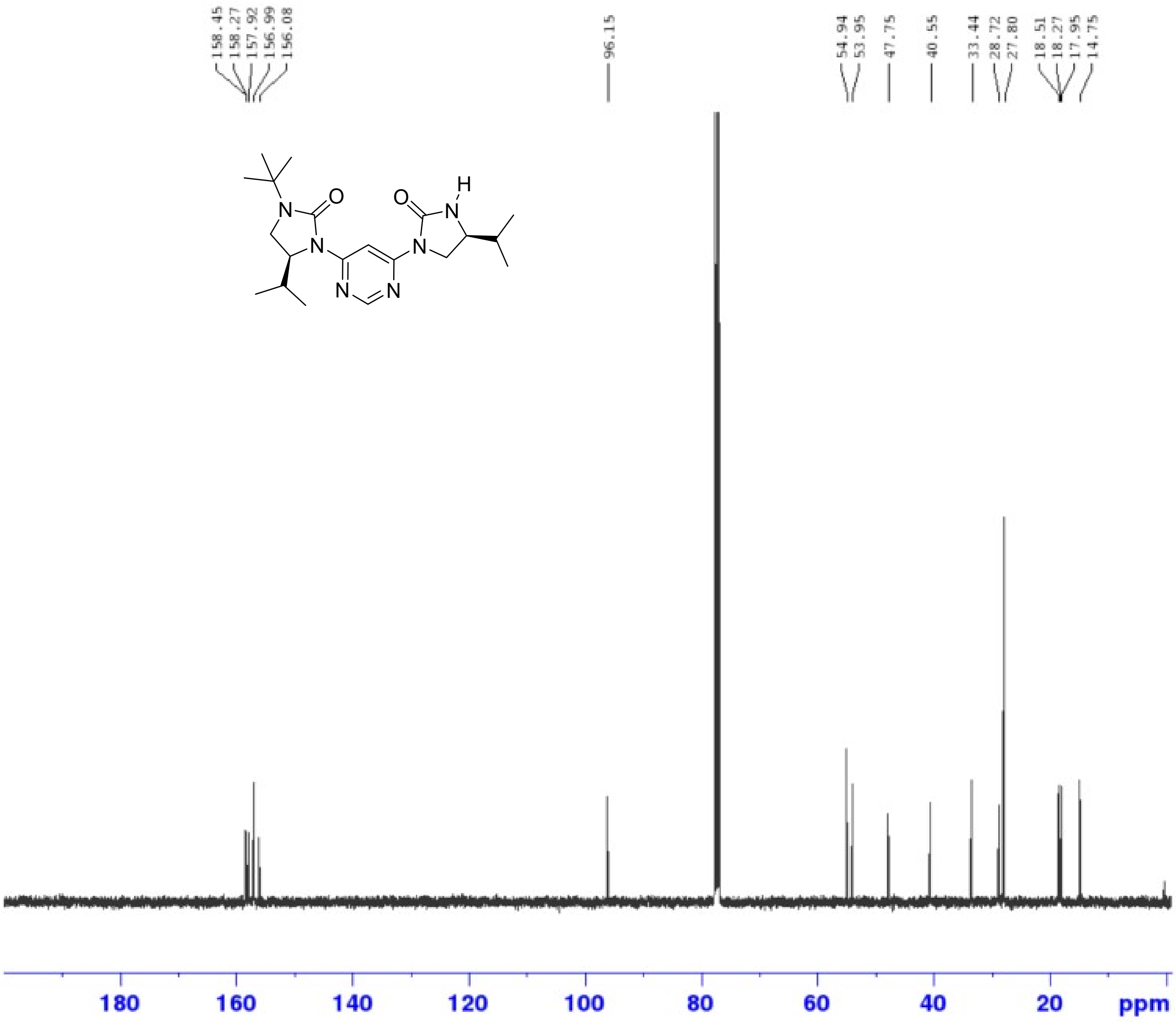

Current Data Parameters
NAME TW-A-176-A RERUN
EXPNO 92
PROCNO 1

F2 - Acquisition Parameters
Date_ 20190429
Time 7.50
INSTRUM spect
PROBHD 5 mm PABBO BB/
PULPROG zgpg30
TD 65536
SOLVENT CDC13
NS 1024
DS 4
SWH 24038.461 Hz
FIDRES 0.366798 Hz
AQ 1.3631488 sec
RG 203
DW 20.800 usec
DE 6.50 usec
TE 300.0 K
D1 2.00000000 sec
D11 0.03000000 sec
TD0 1

======== CHANNEL f1 ========
SFO1 100.5649900 MHz
NUC1 13C
P1 10.00 usec
PLW1 44.46300125 W

======== CHANNEL f2 ========
SFO2 399.9015996 MHz
NUC2 1H
CPDPRG[2 waltz16
PCPD2 90.00 usec
PLW2 7.59999990 W
PLW12 0.20774999 W
PLW13 0.16827001 W

F2 - Processing parameters
SI 32768
SF 100.5549112 MHz
WDW EM
SSB 0
LB 1.00 Hz
GB 0
PC 1.40

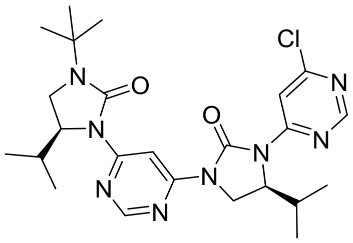

1d

^{1}H NMR

400 MHz

$CDCl_3$

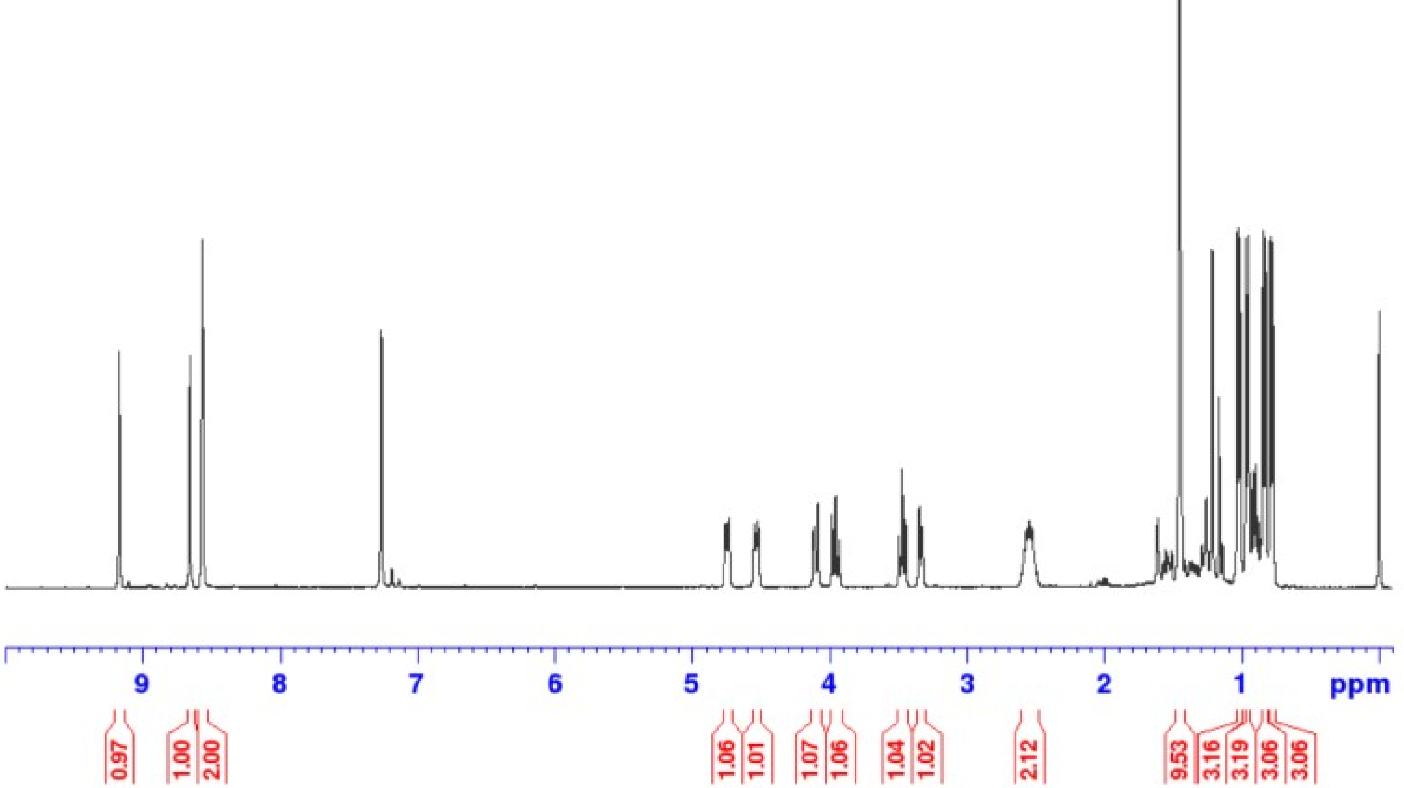

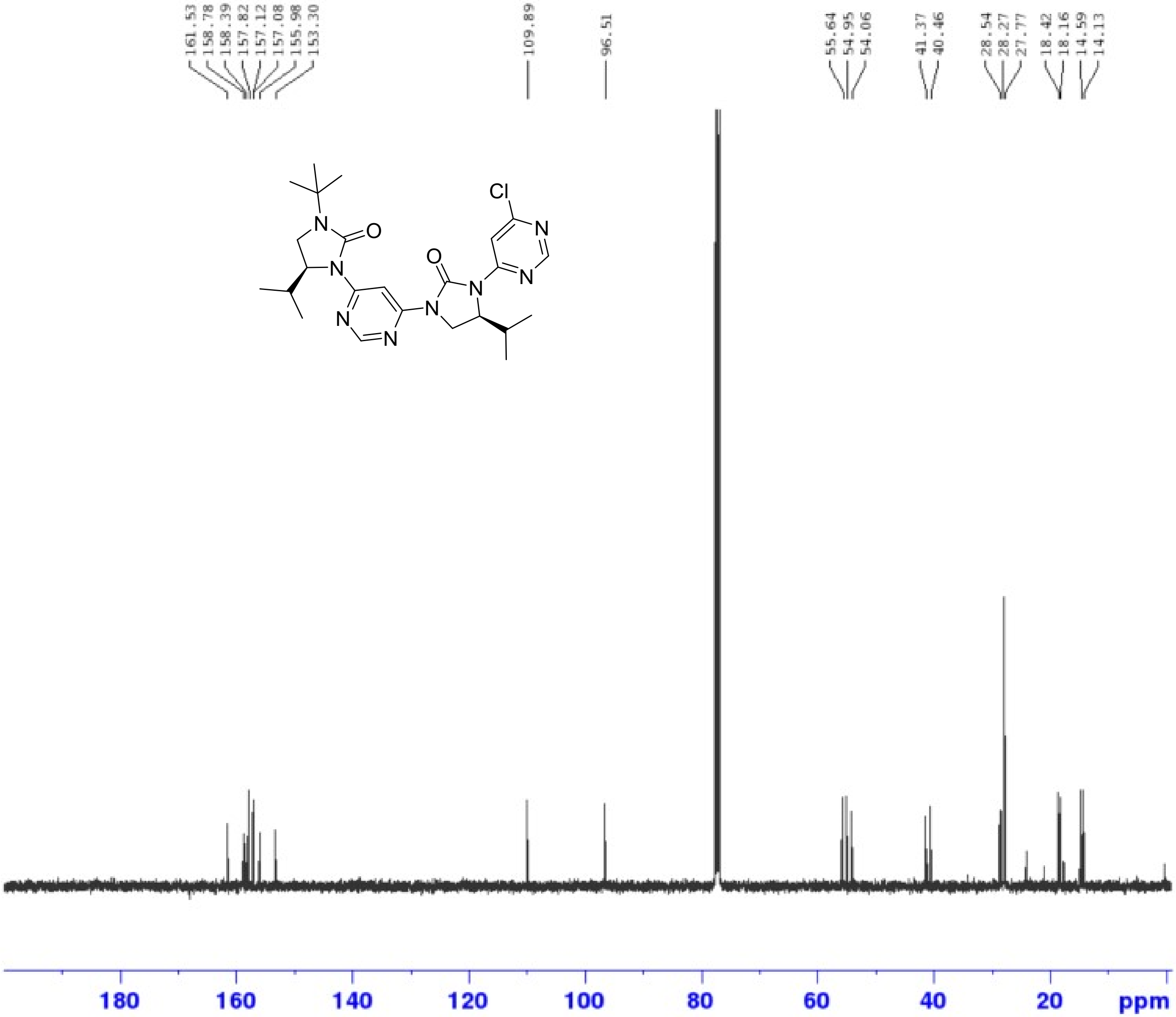

1d

^{13}C NMR

101 MHz

$CDCl_3$

```
Current Data Parameters
NAME      TW-A-178 RERUN
EXPNO                 12
PROCNO                 1

F2 - Acquisition Parameters
Date_           20190429
Time               19.03
INSTRUM            spect
PROBHD   5 mm PABBO BB/
PULPROG          zgpg30
TD                 65536
SOLVENT            CDCl3
NS                  1024
DS                     4
SWH           24038.461 Hz
FIDRES         0.366798 Hz
AQ            1.3631488 sec
RG                   181
DW                20.800 usec
DE                  6.50 usec
TE                 300.0 K
D1            2.00000000 sec
D11           0.03000000 sec
TD0                    1

======== CHANNEL f1 ========
SFO1        100.5649900 MHz
NUC1                 13C
P1                 10.00 usec
PLW1         44.46300125 W

======== CHANNEL f2 ========
SFO2        399.9015996 MHz
NUC2                  1H
CPDPRG[2         waltz16
PCPD2              90.00 usec
PLW2          7.59999990 W
PLW12         0.20774999 W
PLW13         0.16827001 W

F2 - Processing parameters
SI                 32768
SF          100.5549209 MHz
WDW                   EM
SSB                    0
LB                  1.00 Hz
GB                     0
PC                  1.40
```

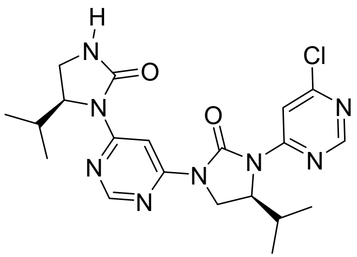

2d

^{1}H NMR

400 MHz

$CDCl_3$

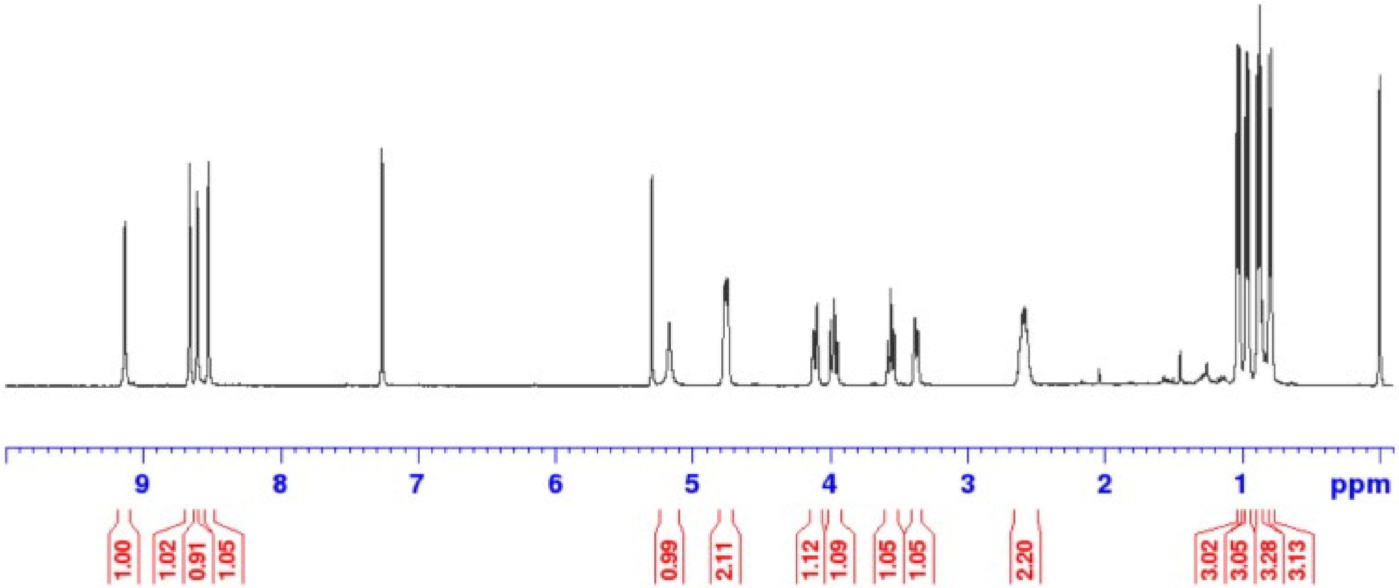


```
Current Data Parameters
NAME         TW-A-179 RERUN
EXPNO                    80
PROCNO                    1

F2 - Acquisition Parameters
Date_              20190427
Time                  21.38
INSTRUM               spect
PROBHD     5 mm PABBO BB/
PULPROG                zg30
TD                   131072
SOLVENT               CDCl3
NS                       64
DS                        0
SWH           12019.230 Hz
FIDRES         0.091699 Hz
AQ            5.4525952 sec
RG                      203
DW               41.600 usec
DE                 9.85 usec
TE                300.0 K
D1           0.10000000 sec
TD0                       1

======== CHANNEL f1 ========
SFO1        399.9024695 MHz
NUC1                     1H
P1                 14.88 usec
PLW1           7.59999990 W

F2 - Processing parameters
SI                   131072
SF          399.9000099 MHz
WDW                      EM
SSB                       0
LB                  0.10 Hz
GB                        0
PC                     1.00
```

2d

^{13}C NMR

101 MHz

$CDCl_3$

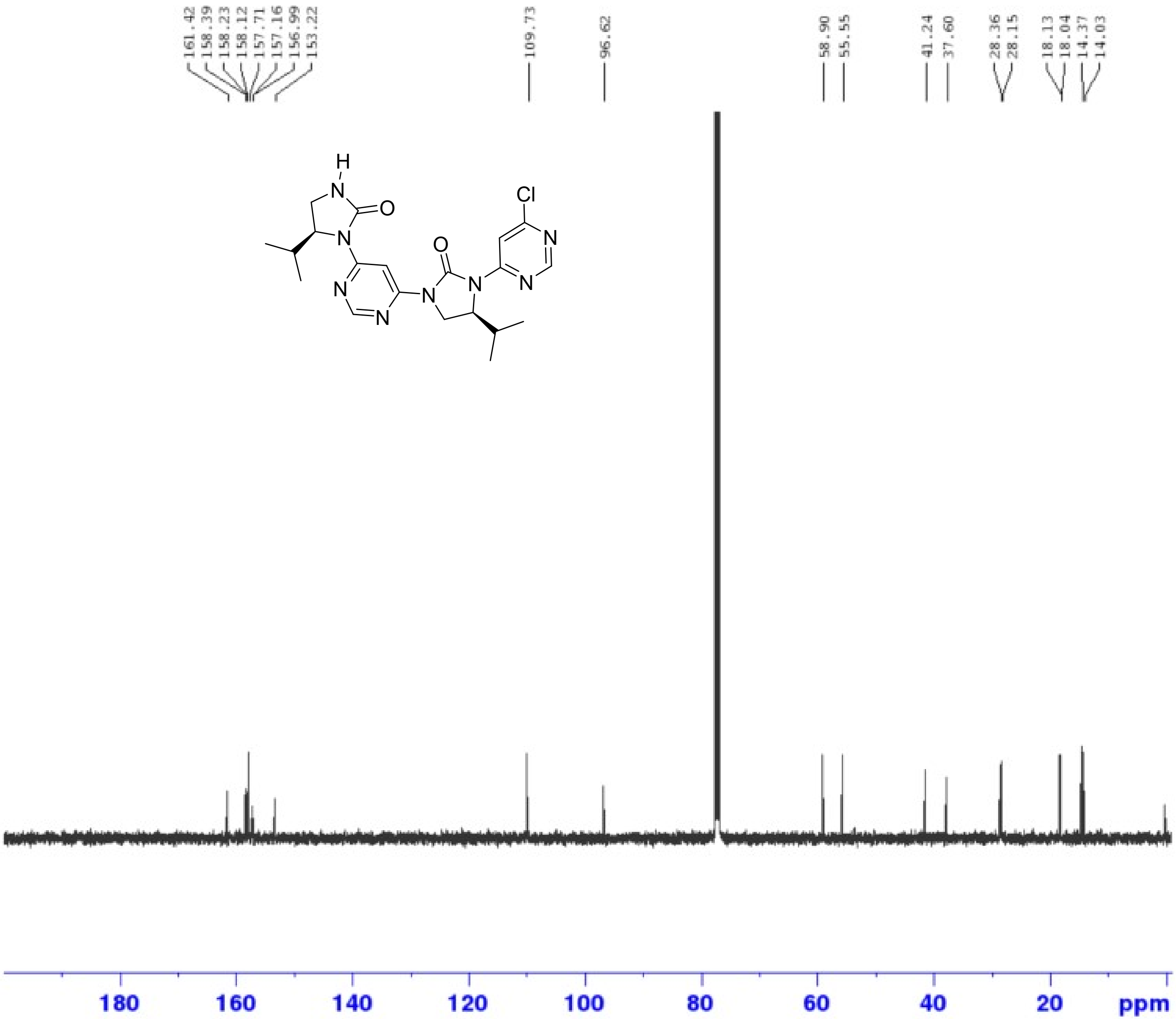

Current Data Parameters

NAME	TW-A-179 RERUN
EXPNO	82
PROCNO	1

F2 - Acquisition Parameters

Date_	20190427
Time	22.51
INSTRUM	spect
PROBHD	5 mm PABBO BB/
PULPROG	zgpg30
TD	65536
SOLVENT	CDCl3
NS	1024
DS	4
SWH	24038.461 Hz
FIDRES	0.366798 Hz
AQ	1.3631488 sec
RG	203
DW	20.800 usec
DE	6.50 usec
TE	300.0 K
D1	2.00000000 sec
D11	0.03000000 sec
TD0	1

======== CHANNEL f1 ========

SFO1	100.5649900 MHz
NUC1	13C
P1	10.00 usec
PLW1	44.46300125 W

======== CHANNEL f2 ========

SFO2	399.9015996 MHz
NUC2	1H
CPDPRG[2	waltz16
PCPD2	90.00 usec
PLW2	7.59999990 W
PLW12	0.20774999 W
PLW13	0.16827001 W

F2 - Processing parameters

SI	32768
SF	100.5549208 MHz
WDW	EM
SSB	0
LB	1.00 Hz
GB	0
PC	1.40

S29

^{1}H NMR

400 MHz

$CDCl_3$

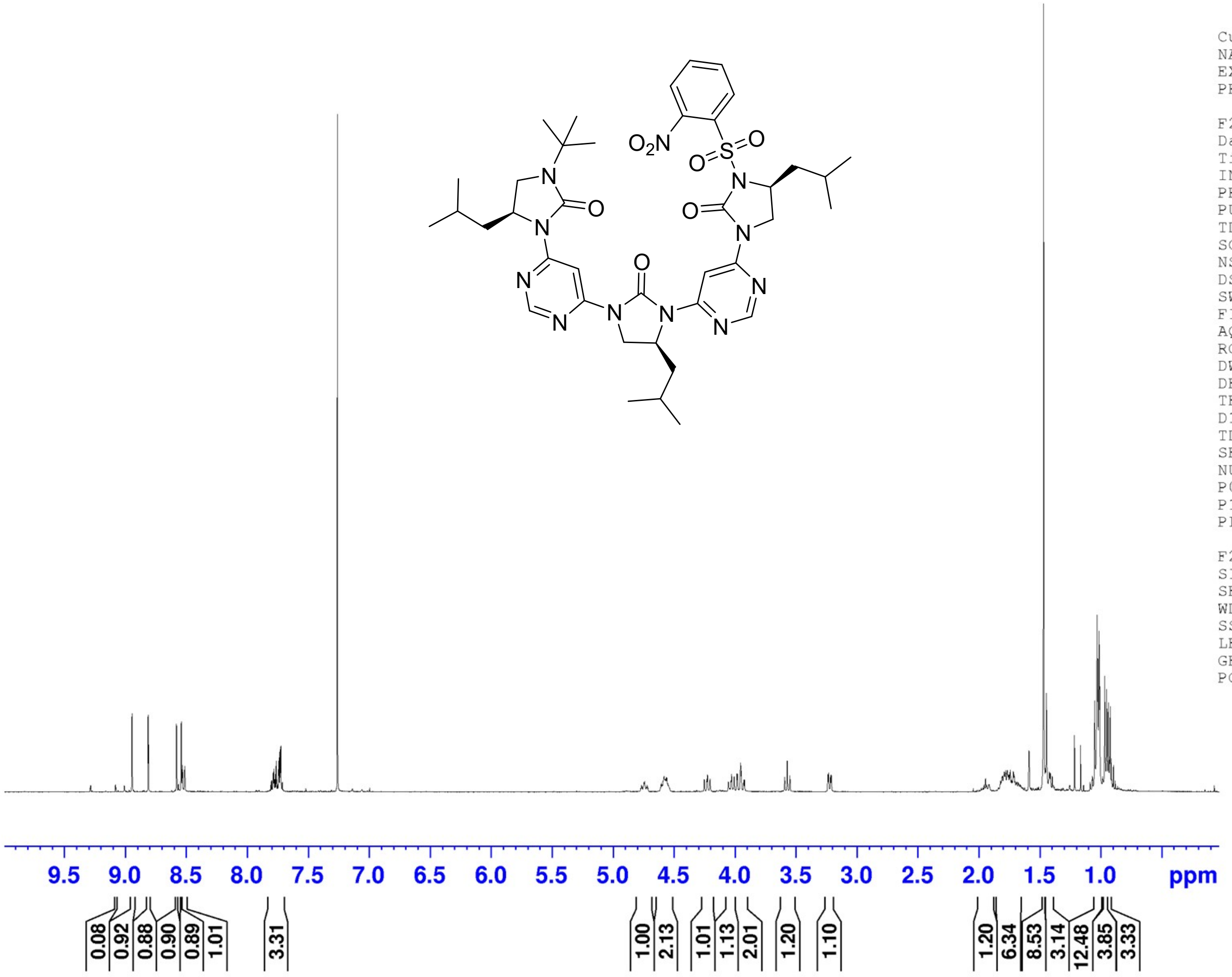


```
Current Data Parameters
NAME          TW-C-391-A
EXPNO                 10
PROCNO                 1

F2 - Acquisition Parameters
Date_           20201111
Time               17.14 h
INSTRUM        AVIII_400
PROBHD   Z108618_0146 (
PULPROG             zg30
TD                 65536
SOLVENT            CDCl3
NS                    16
DS                     2
SWH             8223.685 Hz
FIDRES          0.250967 Hz
AQ             3.9845889 sec
RG                   256
DW                60.800 usec
DE                 17.42 usec
TE                 300.0 K
D1            1.00000000 sec
TD0                    1
SFO1         400.1124708 MHz
NUC1                  1H
P0                  5.00 usec
P1                 15.00 usec
PLW1         17.29199982 W

F2 - Processing parameters
SI                 32768
SF           400.1100088 MHz
WDW                   EM
SSB                    0
LB                  0.30 Hz
GB                     0
PC                  1.00
```

S29

^{13}C NMR

151 MHz

$CDCl_3$

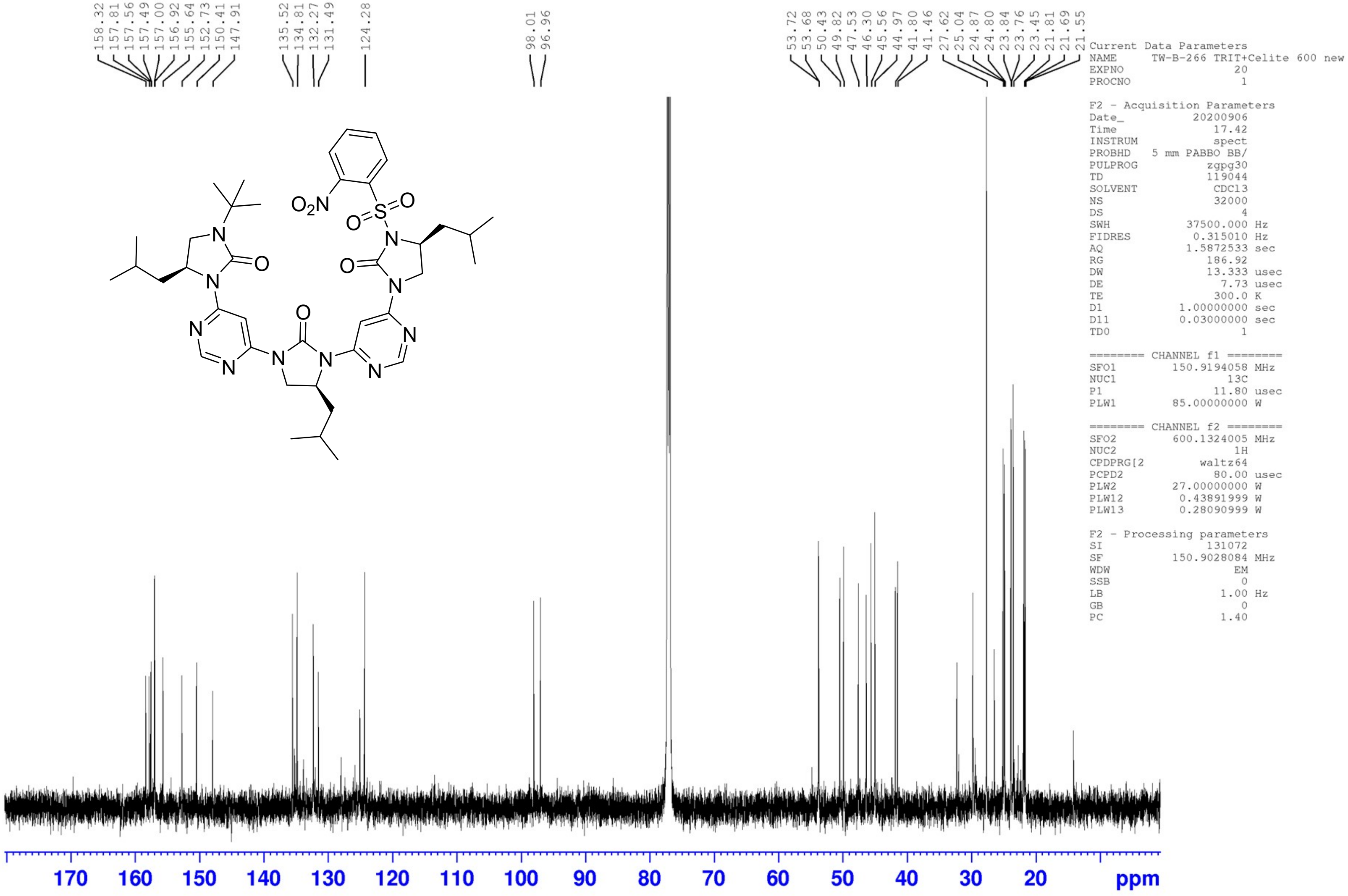

S30

^{1}H NMR

400 MHz

$CDCl_3$

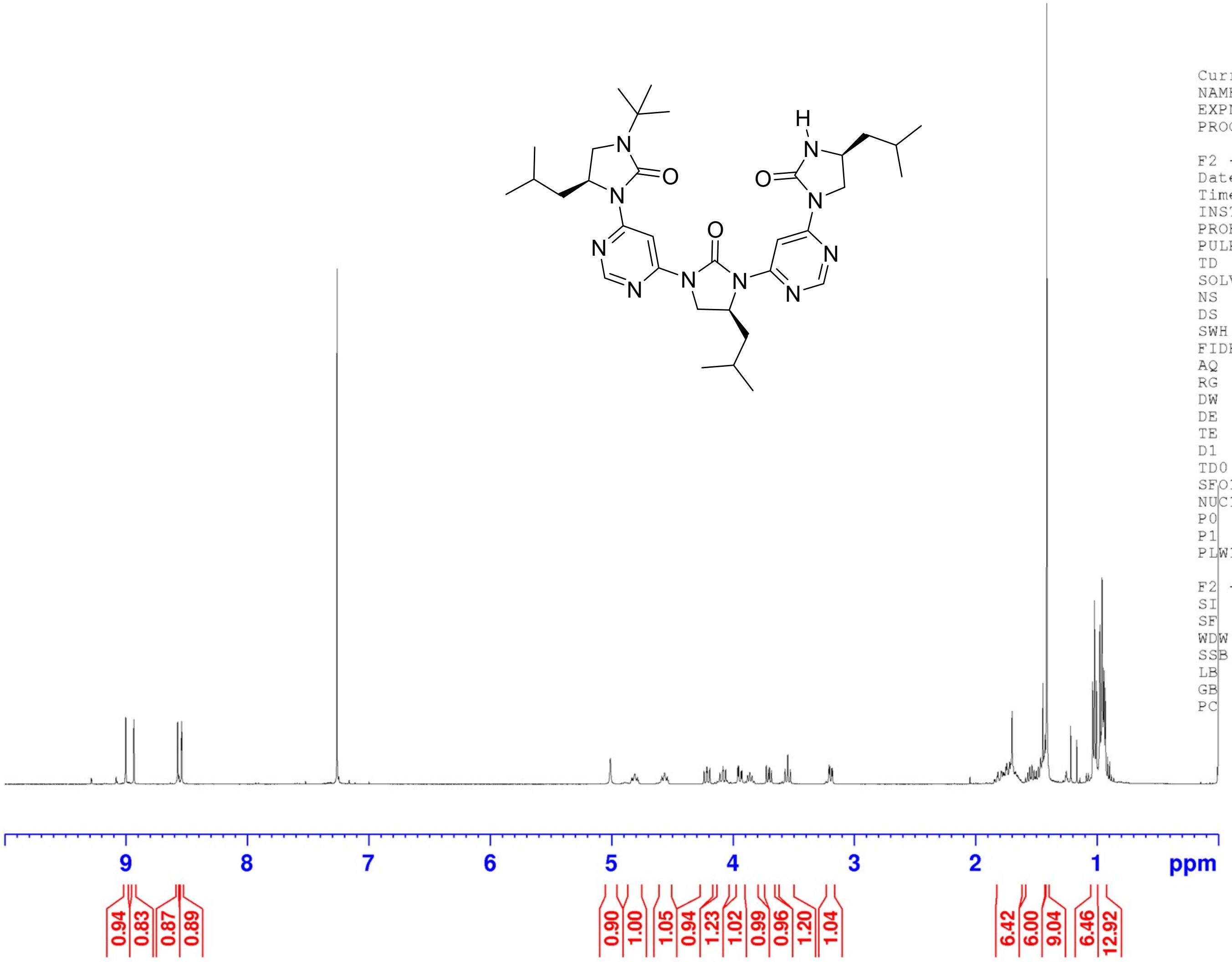


```
Current Data Parameters
NAME              TW-C-392
EXPNO                   10
PROCNO                   1

F2 - Acquisition Parameters
Date_             20201116
Time                9.43 h
INSTRUM           AVIII_400
PROBHD   Z108618_0146 (
PULPROG               zg30
TD                   65536
SOLVENT              CDCl3
NS                      64
DS                       2
SWH               8223.685 Hz
FIDRES            0.250967 Hz
AQ               3.9845889 sec
RG                     256
DW                  60.800 usec
DE                   17.42 usec
TE                   300.0 K
D1              1.00000000 sec
TD0                      1
SFO1           400.1124708 MHz
NUC1                    1H
P0                    5.00 usec
P1                   15.00 usec
PLW1           17.29199982 W

F2 - Processing parameters
SI                   32768
SF             400.1100081 MHz
WDW                     EM
SSB                      0
LB                    0.30 Hz
GB                       0
PC                    1.00
```

S30

^{13}C NMR

101 MHz

$CDCl_3$

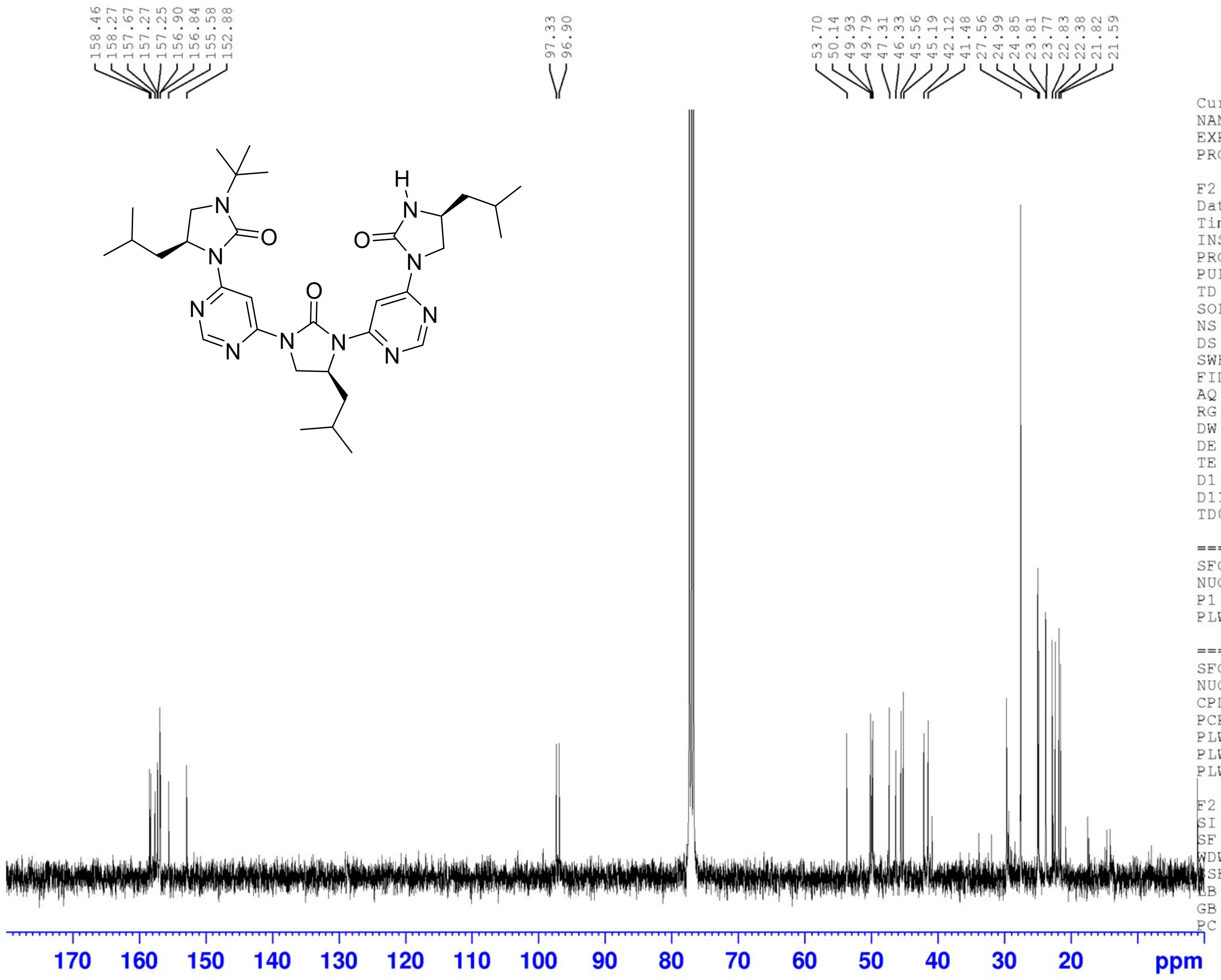


```
Current Data Parameters
NAME      TW-B-254-A FULL
EXPNO                  72
PROCNO                  1

F2 - Acquisition Parameters
Date_            20190828
Time                 5.23
INSTRUM             spect
PROBHD   5 mm PABBO BB/
PULPROG           zgpg30
TD                 65536
SOLVENT            CDCl3
NS                  1024
DS                     4
SWH            24038.461 Hz
FIDRES          0.366798 Hz
AQ             1.3631488 sec
RG                   203
DW                20.800 usec
DE                  6.50 usec
TE                 299.5 K
D1            2.00000000 sec
D11           0.03000000 sec
TD0                    1

======== CHANNEL f1 ========
SFO1         100.5649900 MHz
NUC1                 13C
P1                 10.00 usec
PLW1         44.46300125 W

======== CHANNEL f2 ========
SFO2         399.9015996 MHz
NUC2                  1H
CPDPRG[2         waltz16
PCPD2              90.00 usec
PLW2          7.59999990 W
PLW12         0.20774999 W
PLW13         0.16827001 W

F2 - Processing parameters
SI                 32768
SF           100.5549350 MHz
WDW                   EM
SSB                    0
LB                  1.00 Hz
GB                     0
PC                  1.40
```

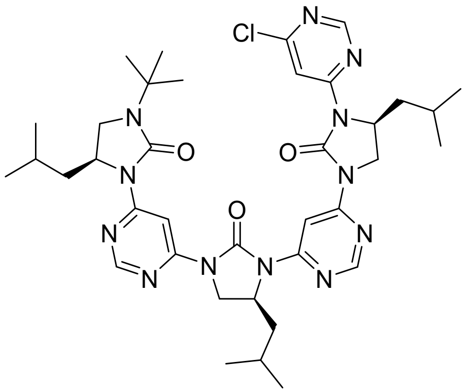

S31

^{1}H NMR

400 MHz

$CDCl_3$

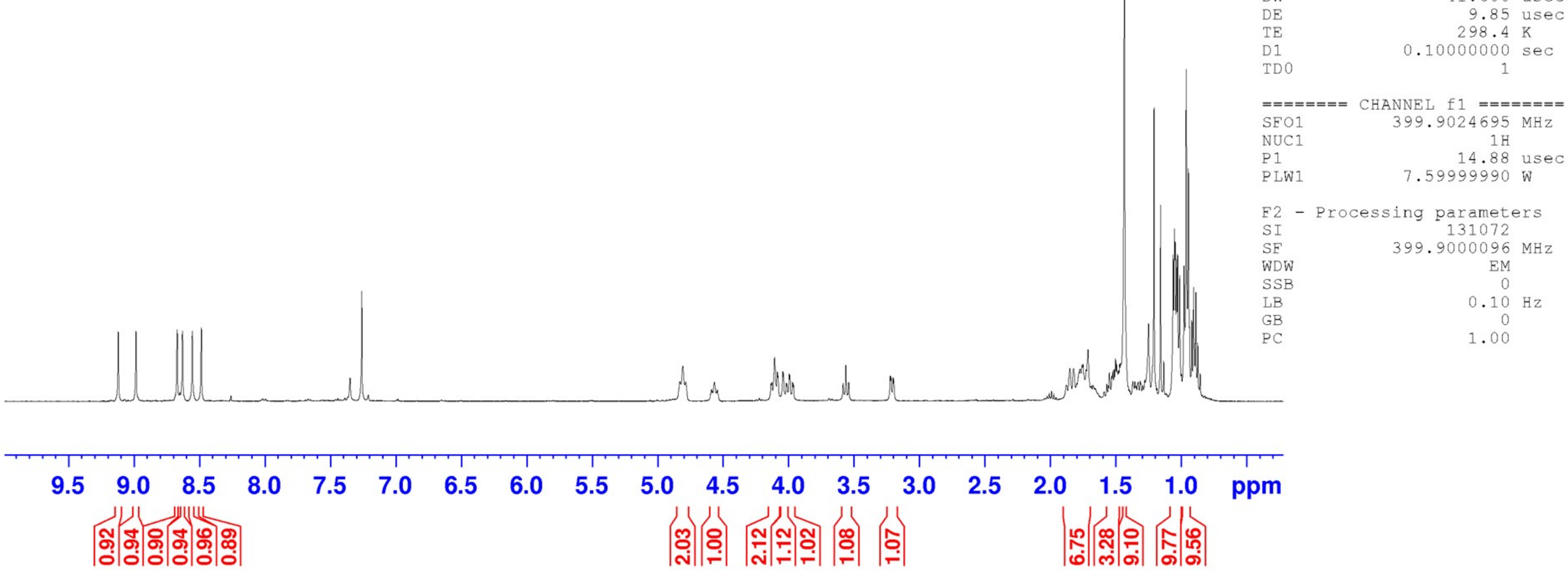

S31

^{13}C NMR

101 MHz

$CDCl_3$

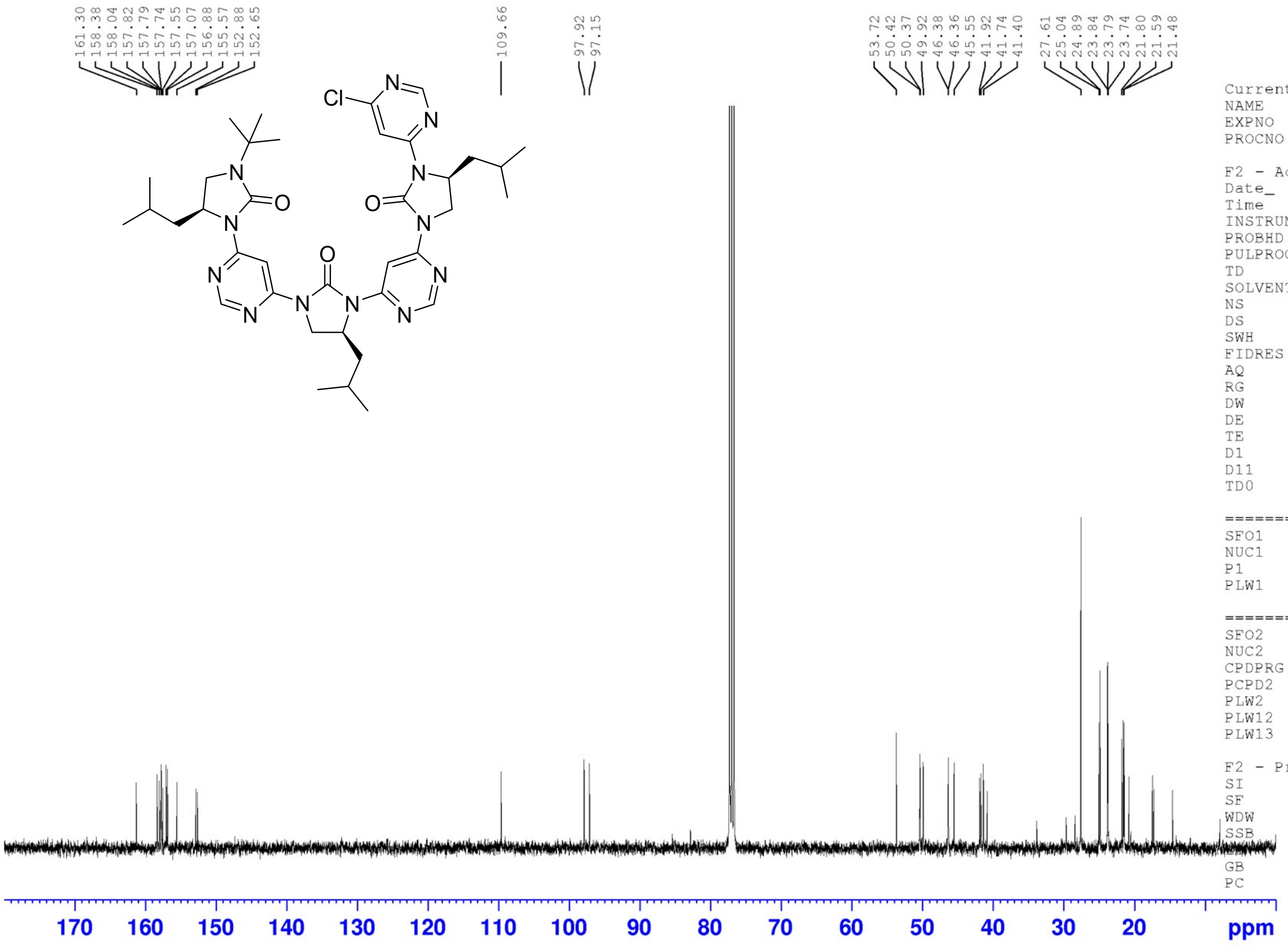

Current Data Parameters
NAME TW-B-274-A
EXPNO 11
PROCNO 1

F2 - Acquisition Parameters
Date_ 20190828
Time 20.06
INSTRUM spect
PROBHD 5 mm PABBO BB/
PULPROG zgpg30
TD 65536
SOLVENT CDCl3
NS 1024
DS 4
SWH 24038.461 Hz
FIDRES 0.366798 Hz
AQ 1.3631488 sec
RG 203
DW 20.800 usec
DE 6.50 usec
TE 299.5 K
D1 2.00000000 sec
D11 0.03000000 sec
TD0 1

======== CHANNEL f1 ========
SFO1 100.5649900 MHz
NUC1 13C
P1 10.00 usec
PLW1 44.46300125 W

======== CHANNEL f2 ========
SFO2 399.9015996 MHz
NUC2 1H
CPDPRG[2 waltz16
PCPD2 90.00 usec
PLW2 7.59999990 W
PLW12 0.20774999 W
PLW13 0.16827001 W

F2 - Processing parameters
SI 32768
SF 100.5549350 MHz
WDW EM
SSB 0
LB 1.00 Hz
GB 0
PC 1.40

4a

^{1}H NMR

600 MHz

$CDCl_3$

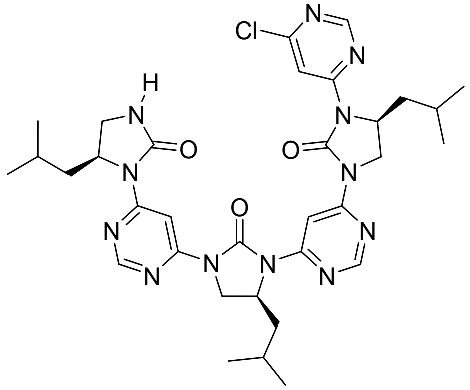

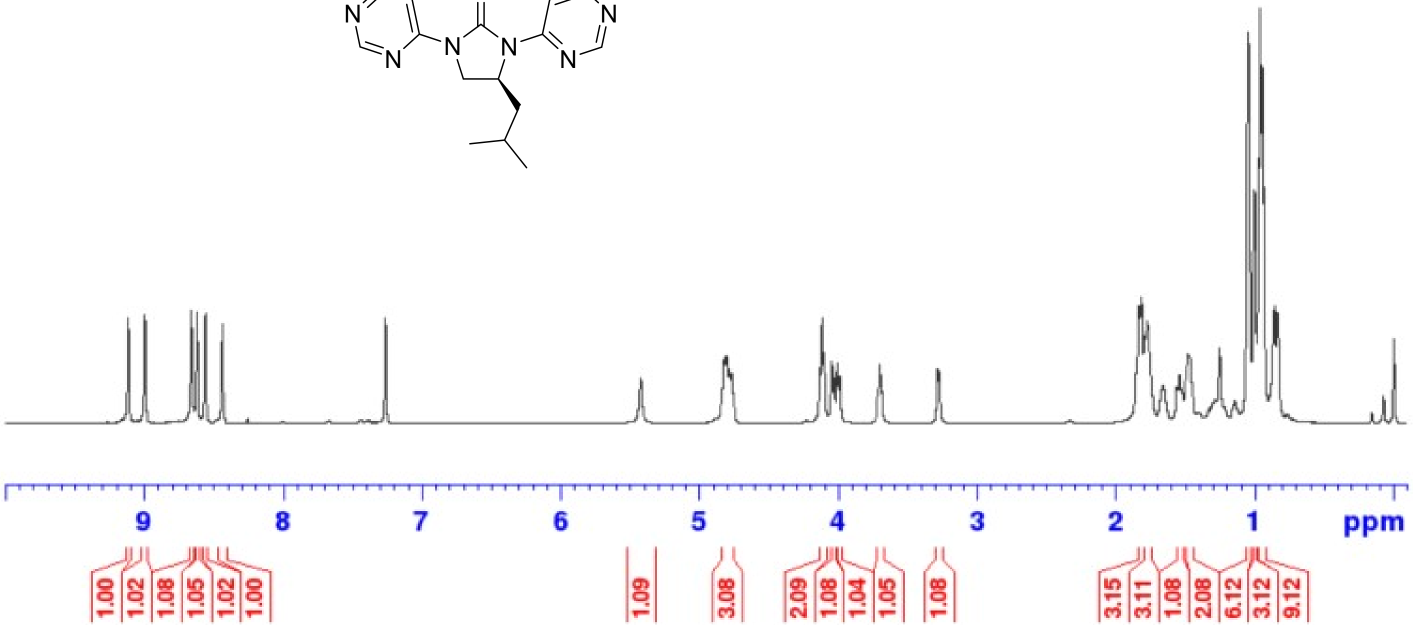


```
Current Data Parameters
NAME      TW-B-278-A TRIT
EXPNO                  10
PROCNO                  1

F2 - Acquisition Parameters
Date_            20190923
Time                19.50
INSTRUM             spect
PROBHD    5 mm PABBO BB/
PULPROG              zg30
TD                  65536
SOLVENT             CDCl3
NS                     16
DS                      2
SWH             12019.230 Hz
FIDRES           0.183399 Hz
AQ              2.7262976 sec
RG                  83.95
DW                 41.600 usec
DE                   6.50 usec
TE                  300.0 K
D1             1.00000000 sec
TD0                     1

======== CHANNEL f1 ========
SFO1          600.1337060 MHz
NUC1                   1H
P1                  10.00 usec
PLW1          26.60000038 W

F2 - Processing parameters
SI                  65536
SF            600.1300150 MHz
WDW                    EM
SSB                     0
LB                   0.30 Hz
GB                      0
PC                   1.00
```

4a

^{13}C NMR

151 MHz

$CDCl_3$

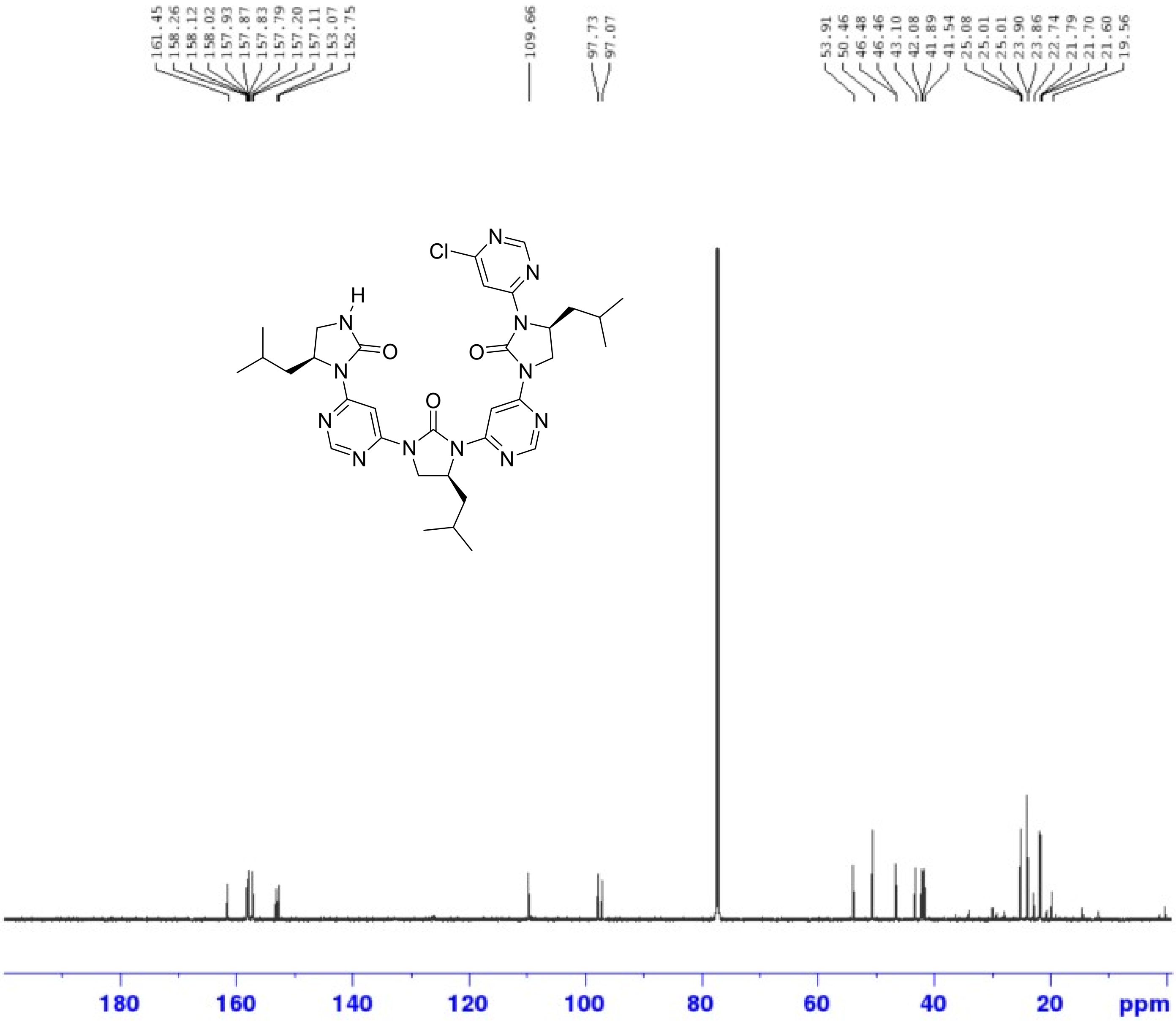


```
Current Data Parameters
NAME        TW-B-278-A TRIT
EXPNO                    11
PROCNO                    1

F2 - Acquisition Parameters
Date_              20190923
Time                  21.32
INSTRUM               spect
PROBHD     5 mm PABBO BB/
PULPROG             zgpg30
TD                    65536
SOLVENT               CDCl3
NS                     2048
DS                        4
SWH           36057.691 Hz
FIDRES         0.550197 Hz
AQ            0.9087659 sec
RG                   186.92
DW               13.867 usec
DE                 6.50 usec
TE                  300.0 K
D1           2.00000000 sec
D11          0.03000000 sec
TD0                       1

======== CHANNEL f1 ========
SFO1        150.9178981 MHz
NUC1                    13C
P1                11.80 usec
PLW1           85.00000000 W

======== CHANNEL f2 ========
SFO2        600.1324005 MHz
NUC2                     1H
CPDPRG[2            waltz16
PCPD2             80.00 usec
PLW2           27.00000000 W
PLW12           0.43891999 W
PLW13           0.28090999 W

F2 - Processing parameters
SI                    32768
SF          150.9027895 MHz
WDW                      EM
SSB                       0
LB                  1.00 Hz
GB                        0
PC                     1.40
```

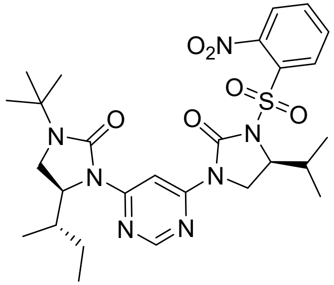

S32

^{1}H NMR

600 MHz

$CDCl_3$

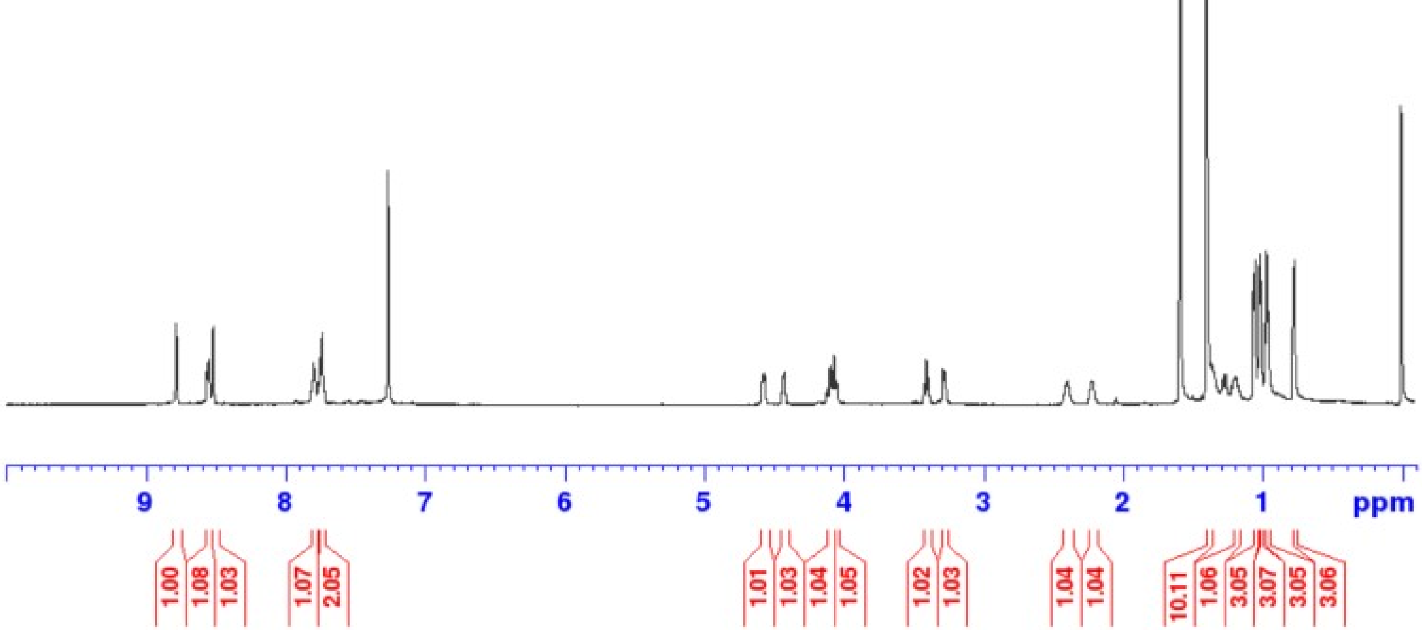

Current Data Parameters
NAME TW-B-267-A 600
EXPNO 140
PROCNO 1

F2 - Acquisition Parameters
Date_ 20190901
Time 3.46
INSTRUM spect
PROBHD 5 mm PABBO BB/
PULPROG zg30
TD 65536
SOLVENT CDCl3
NS 16
DS 2
SWH 12019.230 Hz
FIDRES 0.183399 Hz
AQ 2.7262976 sec
RG 105.21
DW 41.600 usec
DE 6.50 usec
TE 300.0 K
D1 1.00000000 sec
TD0 1

======== CHANNEL f1 ========
SFO1 600.1337060 MHz
NUC1 1H
P1 10.00 usec
PLW1 26.60000038 W

F2 - Processing parameters
SI 65536
SF 600.1300150 MHz
WDW EM
SSB 0
LB 0.30 Hz
GB 0
PC 1.00

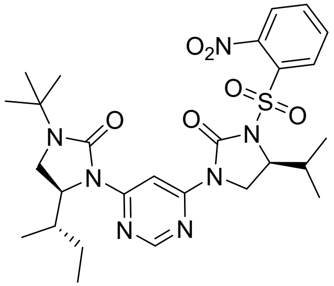

S32

^{13}C NMR

151 MHz

$CDCl_3$

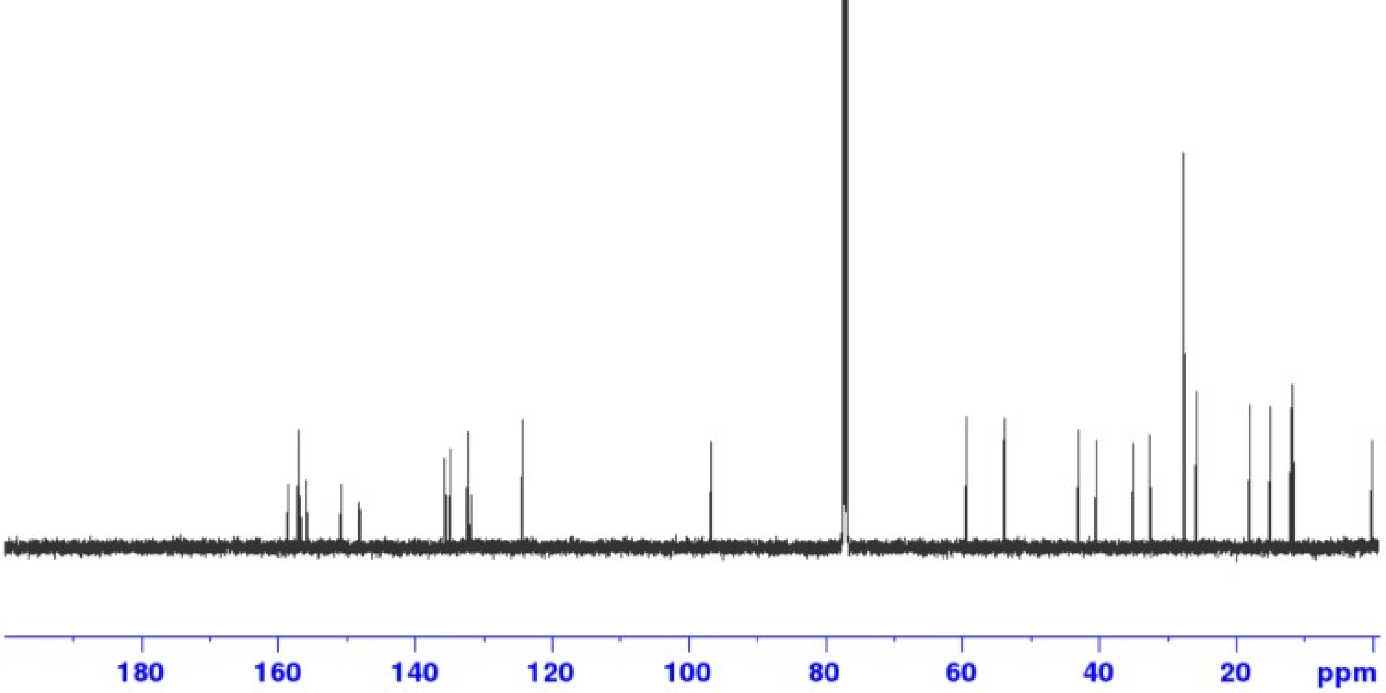

S33

^{1}H NMR

600 MHz

$CDCl_3$

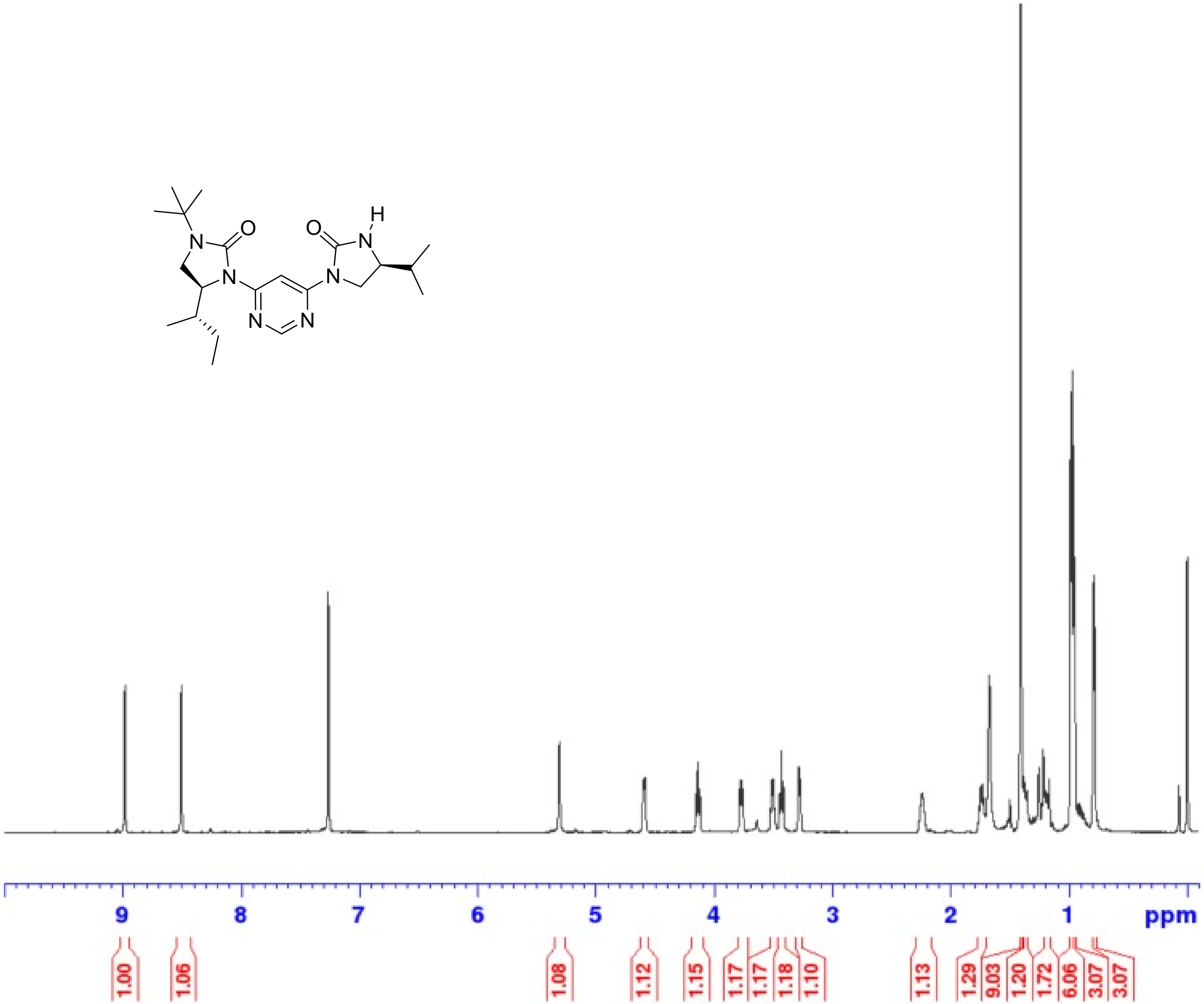


```
Current Data Parameters
NAME      TW-B-271-A 600
EXPNO                190
PROCNO                 1

F2 - Acquisition Parameters
Date_           20190901
Time               17.24
INSTRUM            spect
PROBHD    5 mm PABBO BB/
PULPROG             zg30
TD                 65536
SOLVENT            CDCl3
NS                    16
DS                     2
SWH            12019.230 Hz
FIDRES          0.183399 Hz
AQ             2.7262976 sec
RG                148.05
DW                41.600 usec
DE                  6.50 usec
TE                 300.0 K
D1            1.00000000 sec
TD0                    1

======== CHANNEL f1 ========
SFO1         600.1337060 MHz
NUC1                  1H
P1                 10.00 usec
PLW1         26.60000038 W

F2 - Processing parameters
SI                 65536
SF           600.1300153 MHz
WDW                   EM
SSB                    0
LB                  0.30 Hz
GB                     0
PC                  1.00
```

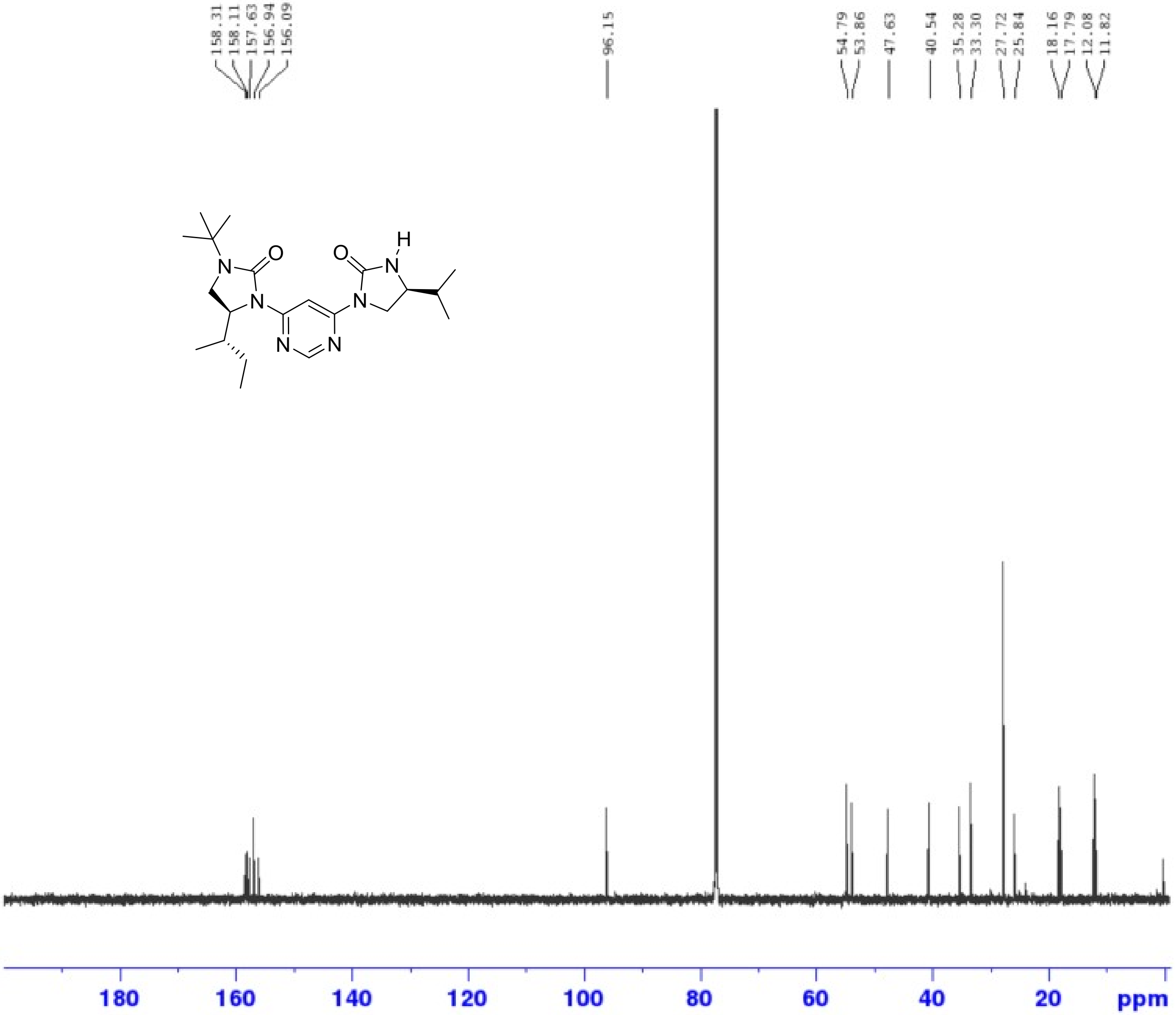

S33

^{13}C NMR

151 MHz

$CDCl_3$

Current Data Parameters
NAME TW-B-271-A 600
EXPNO 192
PROCNO 1

F2 - Acquisition Parameters
Date_ 20190901
Time 19.06
INSTRUM spect
PROBHD 5 mm PABBO BB/
PULPROG zgpg30
TD 65536
SOLVENT CDCl3
NS 2048
DS 4
SWH 36057.691 Hz
FIDRES 0.550197 Hz
AQ 0.9087659 sec
RG 186.92
DW 13.867 usec
DE 6.50 usec
TE 300.0 K
D1 2.00000000 sec
D11 0.03000000 sec
TD0 1

======== CHANNEL f1 ========
SFO1 150.9178981 MHz
NUC1 13C
P1 11.80 usec
PLW1 85.00000000 W

======== CHANNEL f2 ========
SFO2 600.1324005 MHz
NUC2 1H
CPDPRG[2 waltz16
PCPD2 80.00 usec
PLW2 27.00000000 W
PLW12 0.43891999 W
PLW13 0.28090999 W

F2 - Processing parameters
SI 32768
SF 150.9027871 MHz
WDW EM
SSB 0
LB 1.00 Hz
GB 0
PC 1.40

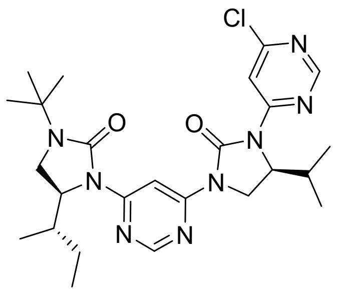

2e

^{1}H NMR

600 MHz

$CDCl_3$

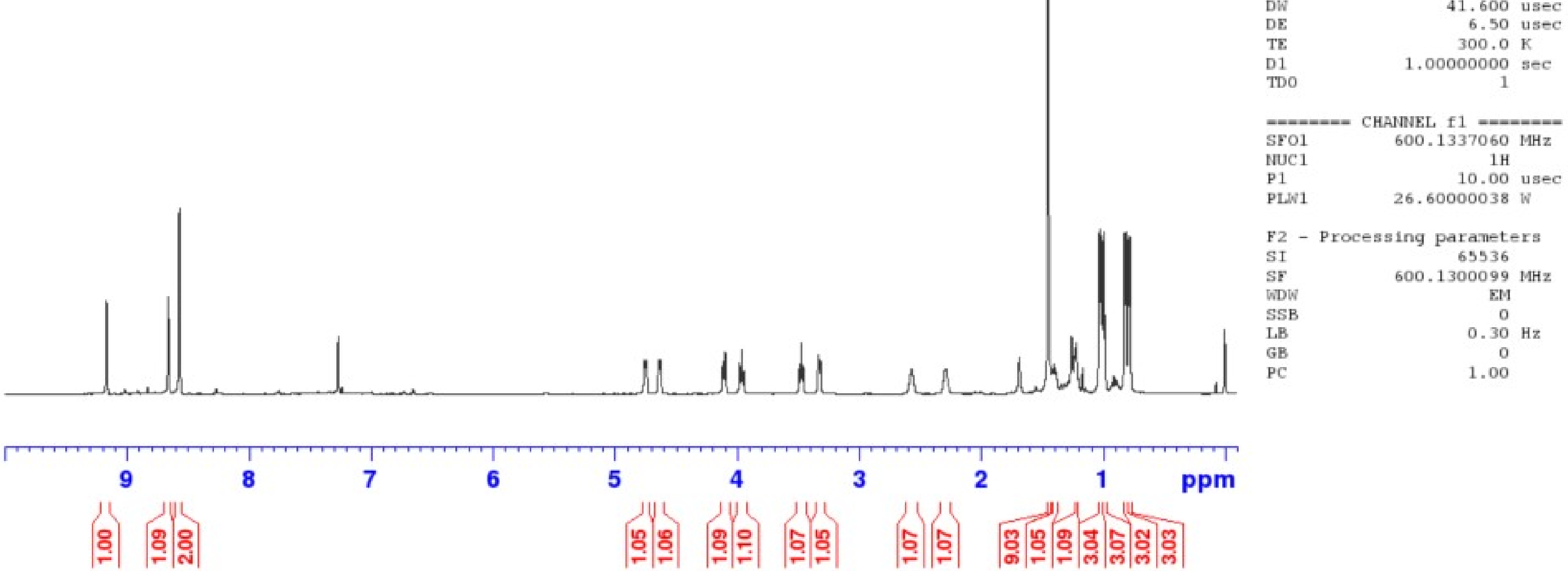

2e

^{13}C NMR

151 MHz

$CDCl_3$

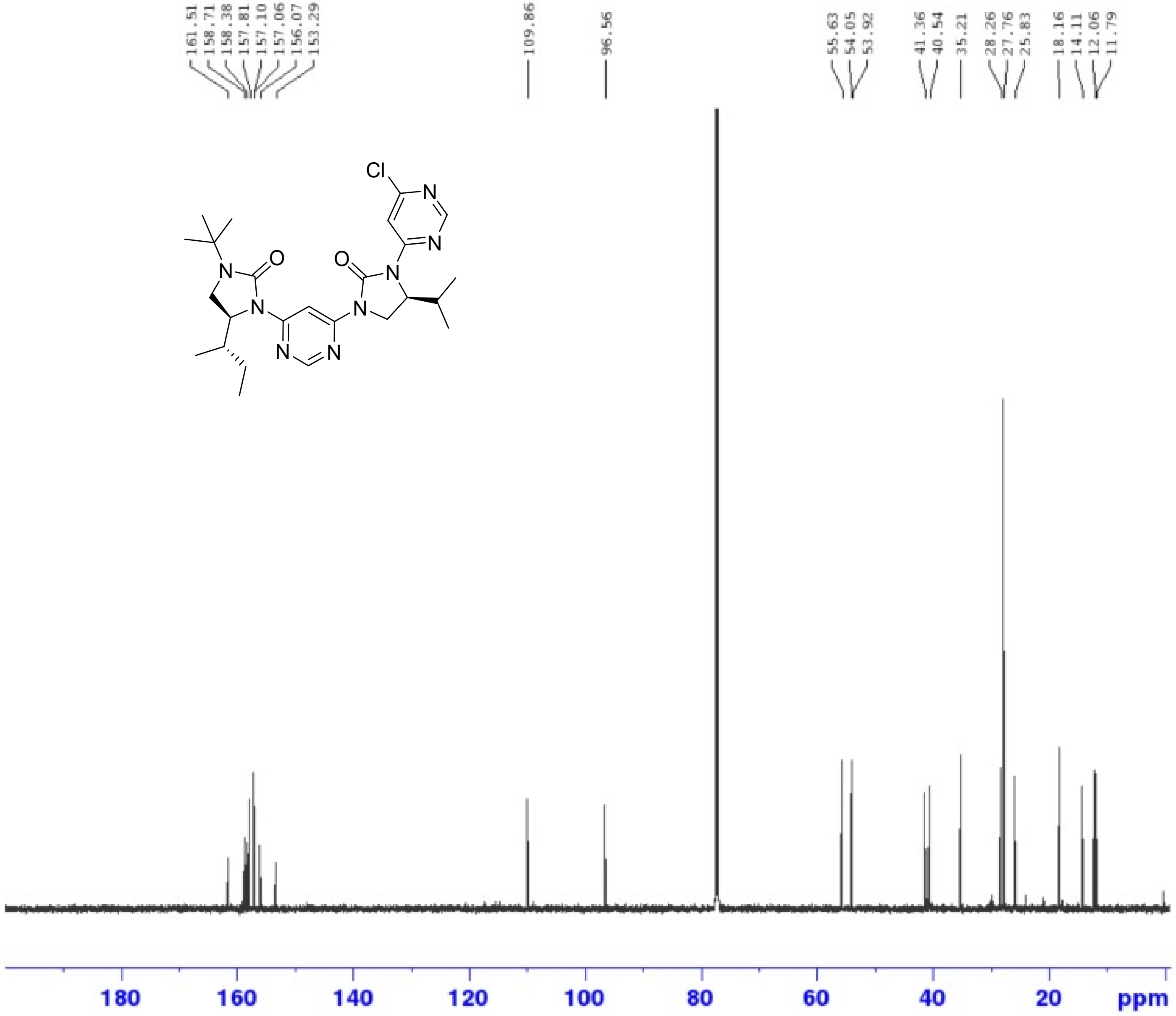

Current Data Parameters
NAME TW-B-276-A 600
EXPNO 11
PROCNO 1

F2 - Acquisition Parameters
Date_ 20190903
Time 8.55
INSTRUM spect
PROBHD 5 mm PABBO BB/
PULPROG zgpg30
TD 65536
SOLVENT CDCl3
NS 1024
DS 4
SWH 36057.691 Hz
FIDRES 0.550197 Hz
AQ 0.9087659 sec
RG 186.92
DW 13.867 usec
DE 6.50 usec
TE 300.0 K
D1 2.00000000 sec
D11 0.03000000 sec
TD0 1

======== CHANNEL f1 ========
SFO1 150.9178981 MHz
NUC1 13C
P1 11.80 usec
PLW1 85.00000000 W

======== CHANNEL f2 ========
SFO2 600.1324005 MHz
NUC2 1H
CPDPRG[2 waltz16
PCPD2 80.00 usec
PLW2 27.00000000 W
PLW12 0.43891999 W
PLW13 0.28090999 W

F2 - Processing parameters
SI 32768
SF 150.9027891 MHz
WDW EM
SSB 0
LB 1.00 Hz
GB 0
PC 1.40

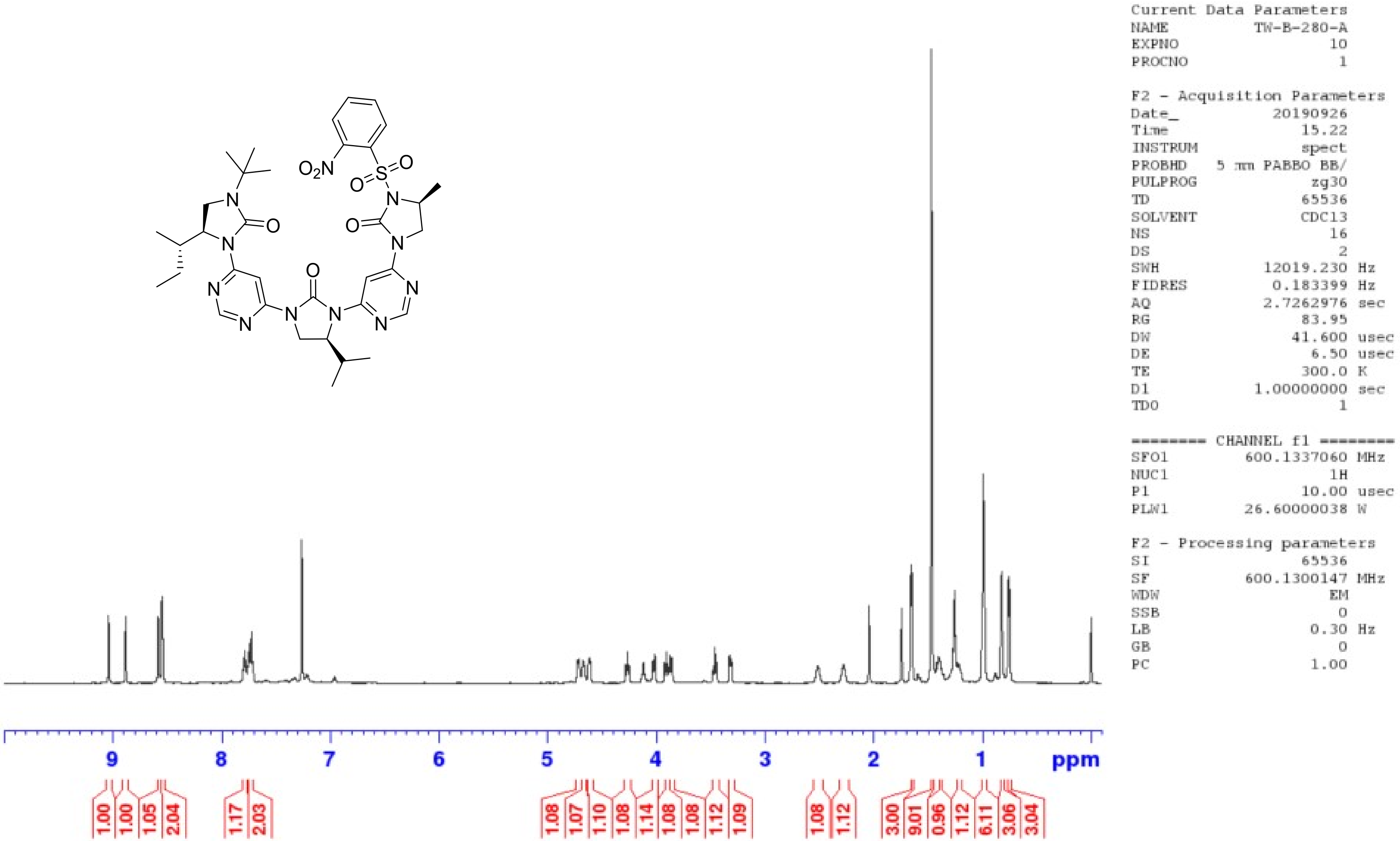

S34

^{1}H NMR

600 MHz

$CDCl_3$

S34

^{13}C NMR

151 MHz

$CDCl_3$

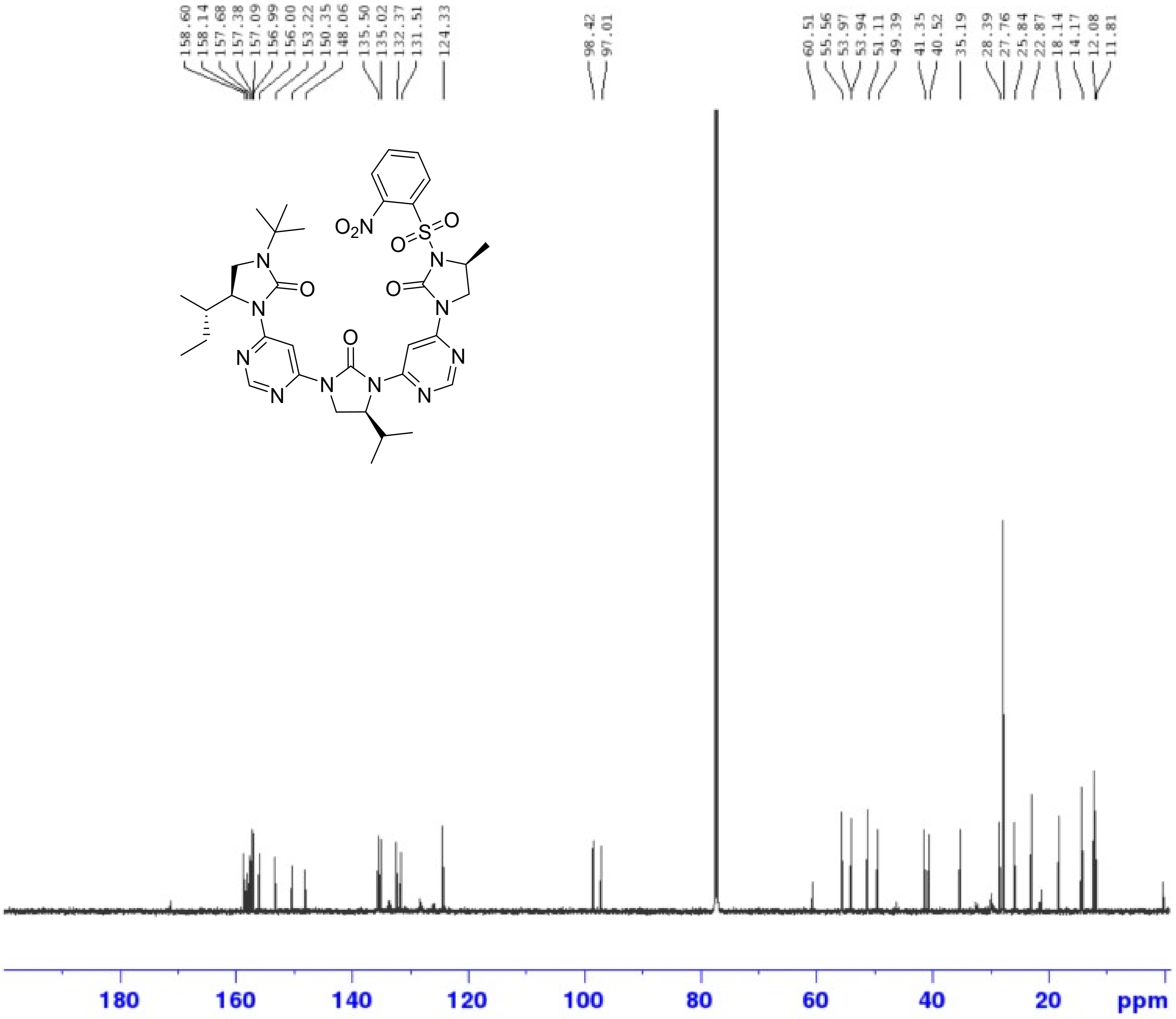


```
Current Data Parameters
NAME              TW-B-280-A
EXPNO                     11
PROCNO                     1

F2 - Acquisition Parameters
Date_               20190926
Time                   23.44
INSTRUM                spect
PROBHD     5 mm PABBO BB/
PULPROG              zgpg30
TD                     65536
SOLVENT                CDCl3
NS                      2048
DS                         4
SWH               36057.691 Hz
FIDRES             0.550197 Hz
AQ                0.9087659 sec
RG                    186.92
DW                    13.867 usec
DE                      6.50 usec
TE                     300.0 K
D1                2.00000000 sec
D11               0.03000000 sec
TD0                        1

======== CHANNEL f1 ========
SFO1             150.9178981 MHz
NUC1                     13C
P1                     11.80 usec
PLW1             85.00000000 W

======== CHANNEL f2 ========
SFO2             600.1324005 MHz
NUC2                      1H
CPDPRG[2             waltz16
PCPD2                  80.00 usec
PLW2             27.00000000 W
PLW12             0.43891999 W
PLW13             0.28090999 W

F2 - Processing parameters
SI                     32768
SF               150.9027896 MHz
WDW                       EM
SSB                        0
LB                      1.00 Hz
GB                         0
PC                      1.40
```

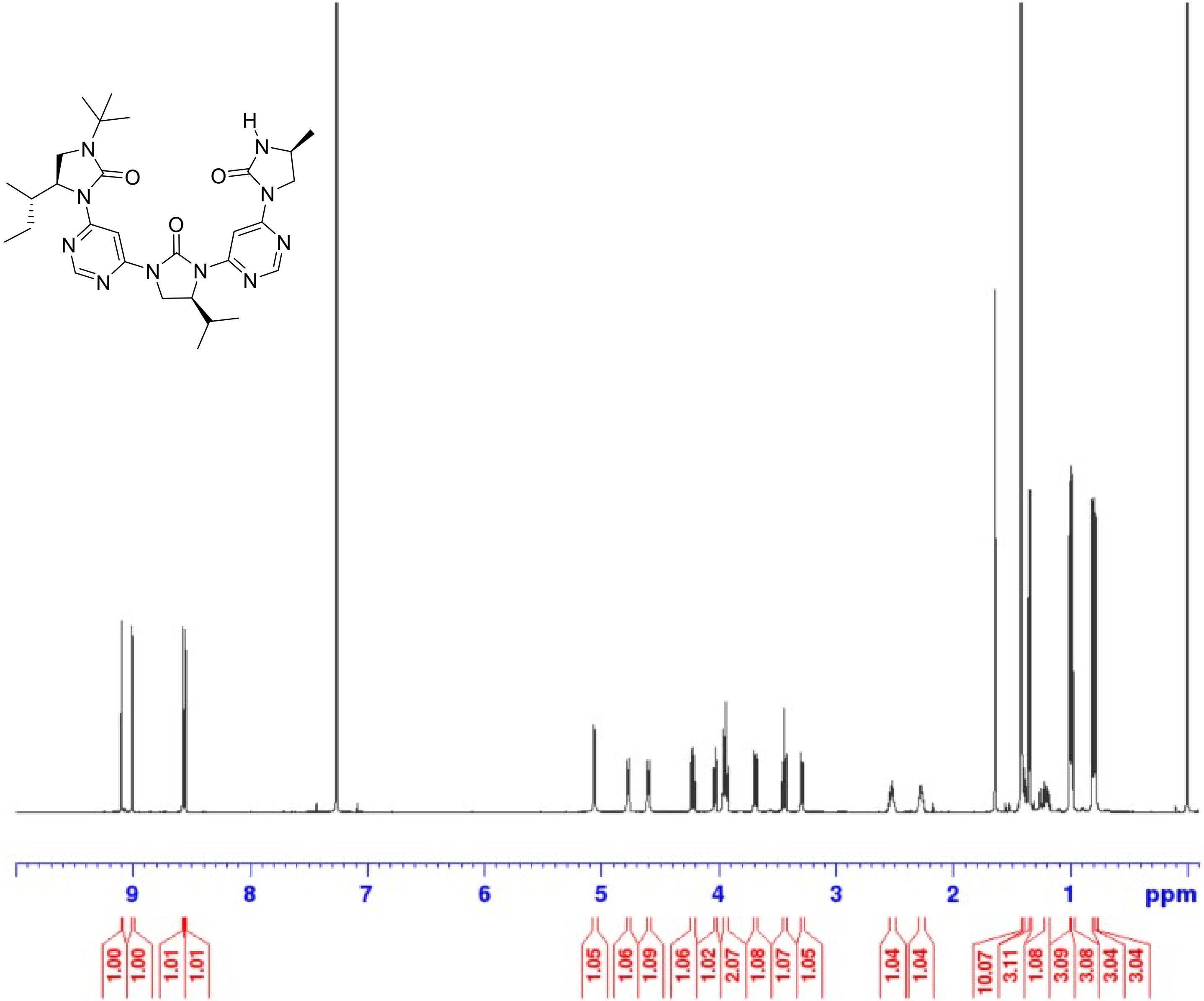

S35

^{1}H NMR

600 MHz

$CDCl_3$

```
Current Data Parameters
NAME       TW-C-405-600 FULL
EXPNO                    10
PROCNO                    1

F2 - Acquisition Parameters
Date_              20201213
Time                  14.56
INSTRUM               spect
PROBHD      5 mm PABBO BB/
PULPROG                zg30
TD                    65536
SOLVENT               CDCl3
NS                       64
DS                        2
SWH              12019.230 Hz
FIDRES            0.183399 Hz
AQ               2.7262976 sec
RG                  186.92
DW                  41.600 usec
DE                    6.50 usec
TE                   300.0 K
D1              1.00000000 sec
TD0                       1

======== CHANNEL f1 ========
SFO1         600.1337060 MHz
NUC1                  1H
P1                 10.00 usec
PLW1         26.60000038 W

F2 - Processing parameters
SI                    65536
SF           600.1300141 MHz
WDW                     EM
SSB                      0
LB                    0.30 Hz
GB                       0
PC                    1.00
```

S35

^{13}C NMR

151 MHz

$CDCl_3$

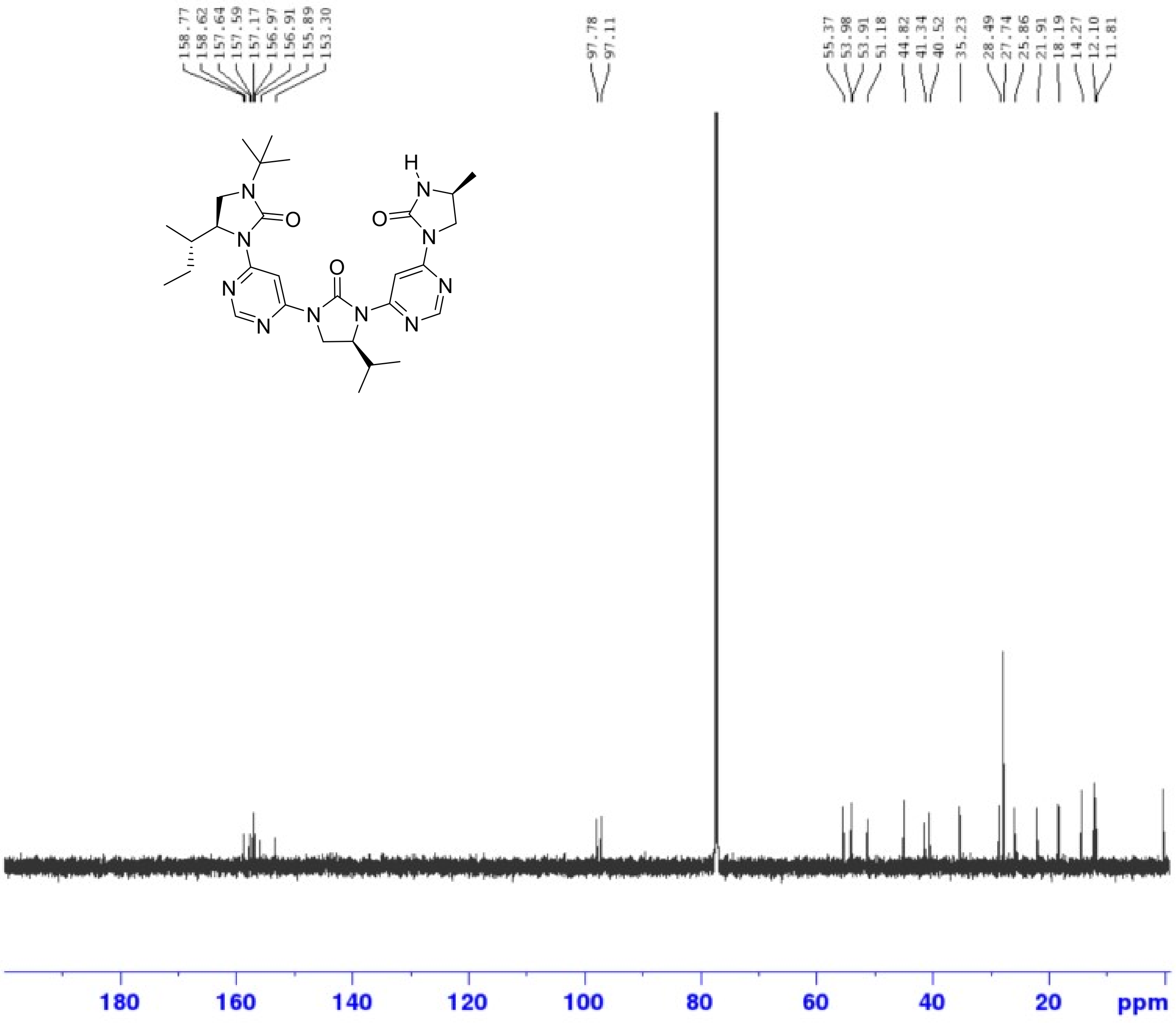


```
Current Data Parameters
NAME        TW-C-405-600 FULL
EXPNO                     11
PROCNO                     1

F2 - Acquisition Parameters
Date_               20201213
Time                   15.42
INSTRUM                spect
PROBHD    5 mm PABBO BB/
PULPROG              zgpg30
TD                    119044
SOLVENT                CDCl3
NS                      1024
DS                         4
SWH               37500.000 Hz
FIDRES             0.315010 Hz
AQ                1.5872533 sec
RG                    186.92
DW                    13.333 usec
DE                      7.73 usec
TE                     300.0 K
D1               1.00000000 sec
D11              0.03000000 sec
TD0                        1

======== CHANNEL f1 ========
SFO1           150.9194058 MHz
NUC1                     13C
P1                     11.80 usec
PLW1            85.00000000 W

======== CHANNEL f2 ========
SFO2           600.1324005 MHz
NUC2                      1H
CPDPRG[2             waltz64
PCPD2                  80.00 usec
PLW2            27.00000000 W
PLW12            0.43891999 W
PLW13            0.28090999 W

F2 - Processing parameters
SI                    131072
SF             150.9027872 MHz
WDW                       EM
SSB                        0
LB                      1.00 Hz
GB                         0
PC                      1.40
```

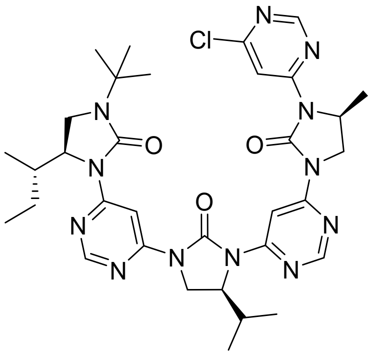

S36

^{1}H NMR

600 MHz

$CDCl_3$

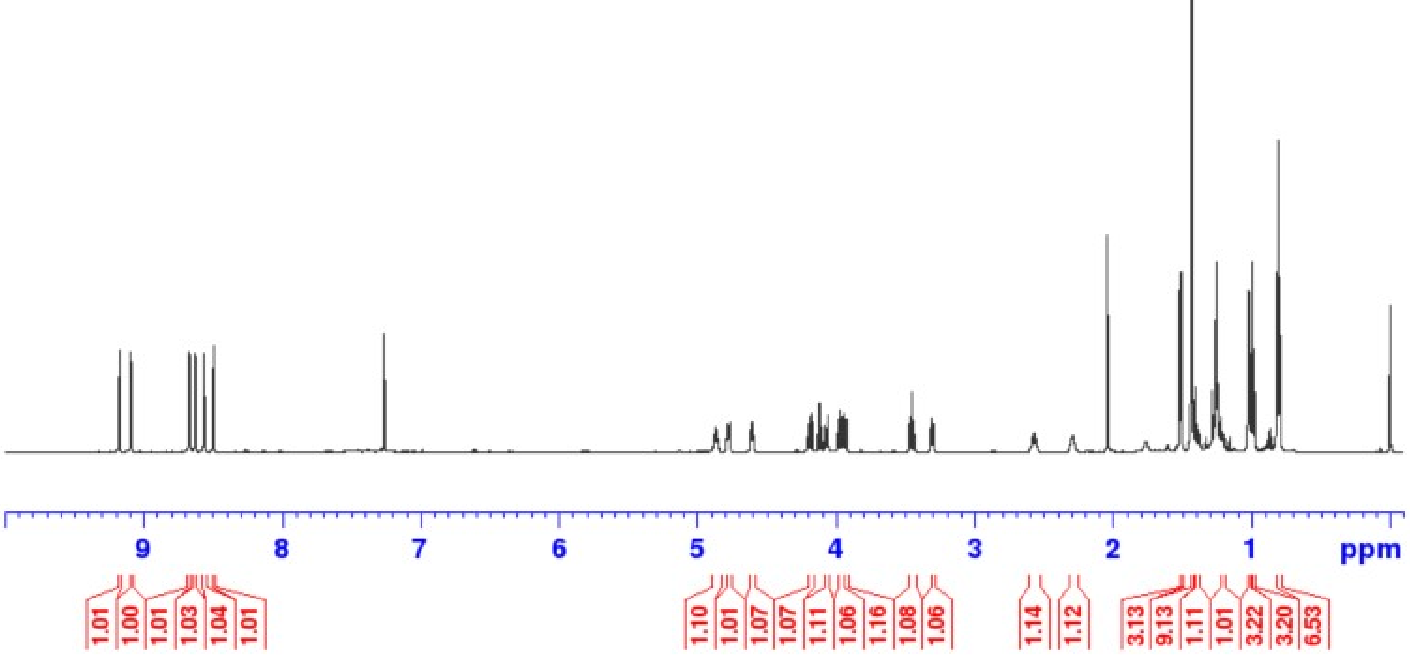


```
Current Data Parameters
NAME          TW-B-296-B
EXPNO                 10
PROCNO                 1

F2 - Acquisition Parameters
Date_           20191126
Time               16.23
INSTRUM            spect
PROBHD     5 mm PABBO BB/
PULPROG             zg30
TD                 65536
SOLVENT            CDCl3
NS                    16
DS                     2
SWH            12019.230 Hz
FIDRES          0.183399 Hz
AQ             2.7262976 sec
RG                 83.95
DW                41.600 usec
DE                  6.50 usec
TE                 300.0 K
D1            1.00000000 sec
TD0                    1

======== CHANNEL f1 ========
SFO1         600.1337060 MHz
NUC1                  1H
P1                 10.00 usec
PLW1         26.60000038 W

F2 - Processing parameters
SI                 65536
SF           600.1300148 MHz
WDW                   EM
SSB                    0
LB                  0.30 Hz
GB                     0
PC                  1.00
```

S36

^{13}C NMR

151 MHz

$CDCl_3$

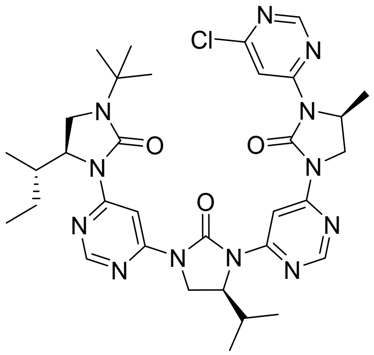

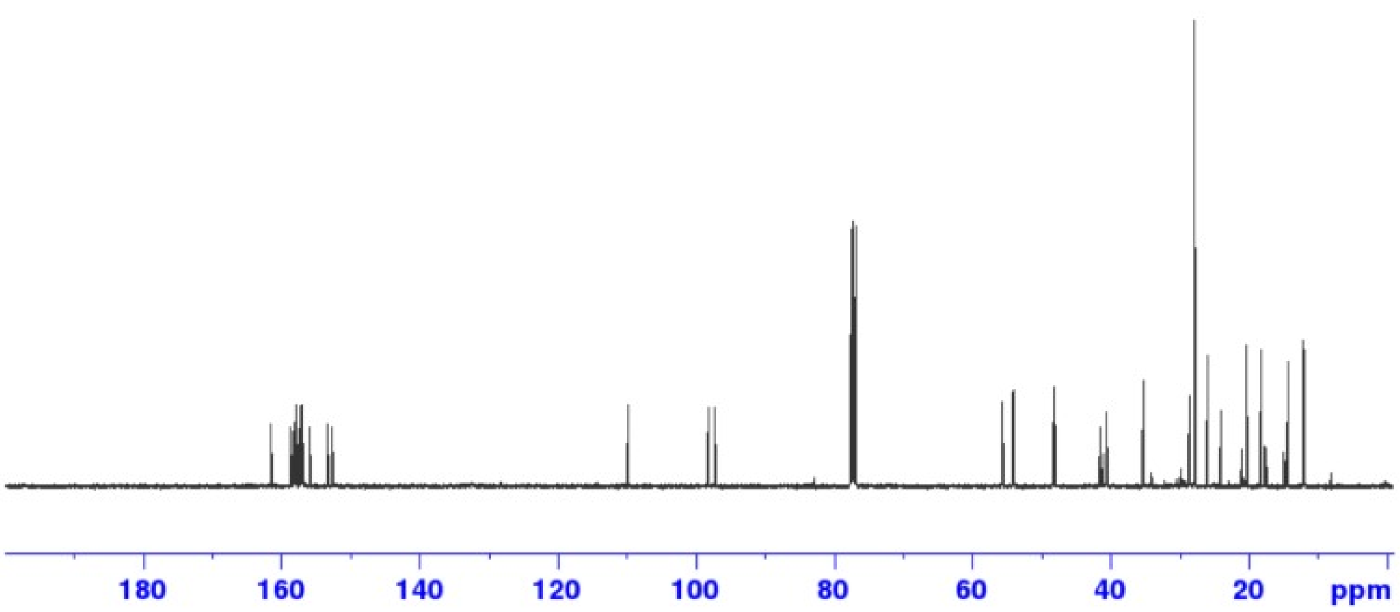

Current Data Parameters
NAME TW-C-407
EXPNO 11
PROCNO 1

F2 - Acquisition Parameters
Date_ 20201216
Time 0.52
INSTRUM AVIII_400
PROBHD 5 mm PABBO BB/
PULPROG zgpg30
TD 96150
SOLVENT CDCl3
NS 1024
DS 4
SWH 24038.461 Hz
FIDRES 0.250010 Hz
AQ 1.9999200 sec
RG 80.6
DW 20.800 usec
DE 6.50 usec
TE 299.6 K
D1 1.00000000 sec
D11 0.03000000 sec
TD0 1

======== CHANNEL f1 ========
SFO1 100.5675047 MHz
NUC1 13C
P1 9.00 usec
PLW1 96.68000031 W

======== CHANNEL f2 ========
SFO2 399.9115996 MHz
NUC2 1H
CPDPRG[2 waltz64
PCPD2 90.00 usec
PLW2 17.29199982 W
PLW12 0.48032999 W
PLW13 0.38907000 W

F2 - Processing parameters
SI 131072
SF 100.5574376 MHz
WDW EM
SSB 0
LB 1.00 Hz
GB 0
PC 1.40

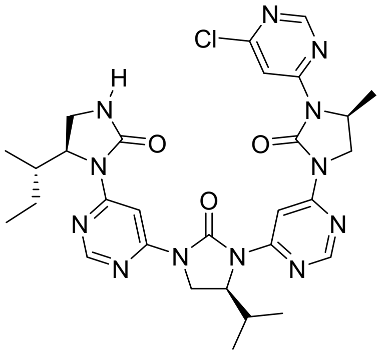

4b

^{1}H NMR

400 MHz

$CDCl_3$

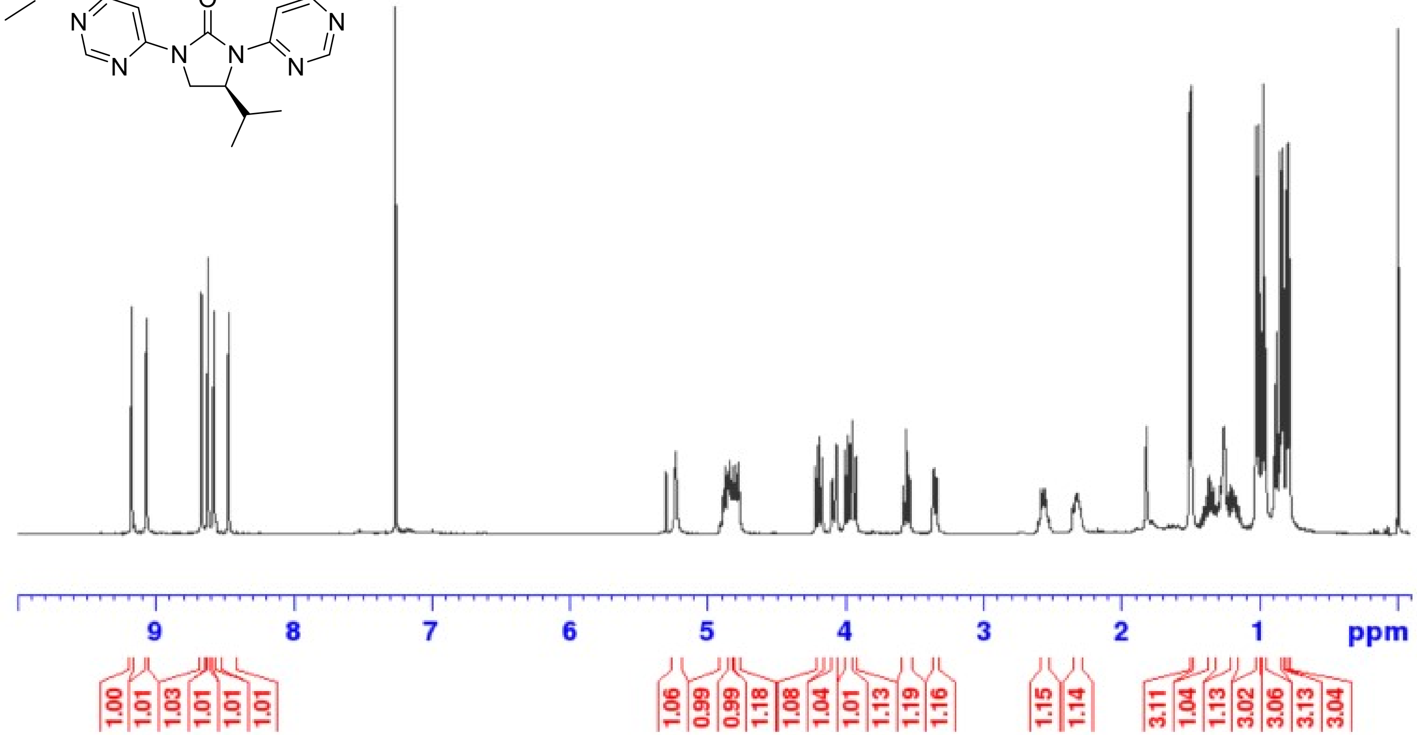


```
Current Data Parameters
NAME              TW-C-408
EXPNO                   10
PROCNO                   1

F2 - Acquisition Parameters
Date_             20201216
Time                 15.13
INSTRUM          AVIII_400
PROBHD     5 mm PABBO BB/
PULPROG               zg30
TD                   65536
SOLVENT              CDCl3
NS                      16
DS                       2
SWH               8223.685 Hz
FIDRES            0.125483 Hz
AQ               3.9845889 sec
RG                      64
DW                  60.800 usec
DE                    6.50 usec
TE                   294.3 K
D1              1.00000000 sec
TD0                      1

======== CHANNEL f1 ========
SFO1           399.9124696 MHz
NUC1                    1H
P1                   15.00 usec
PLW1           17.29199982 W

F2 - Processing parameters
SI                   32768
SF             399.9100098 MHz
WDW                     EM
SSB                      0
LB                    0.30 Hz
GB                       0
PC                    1.00
```

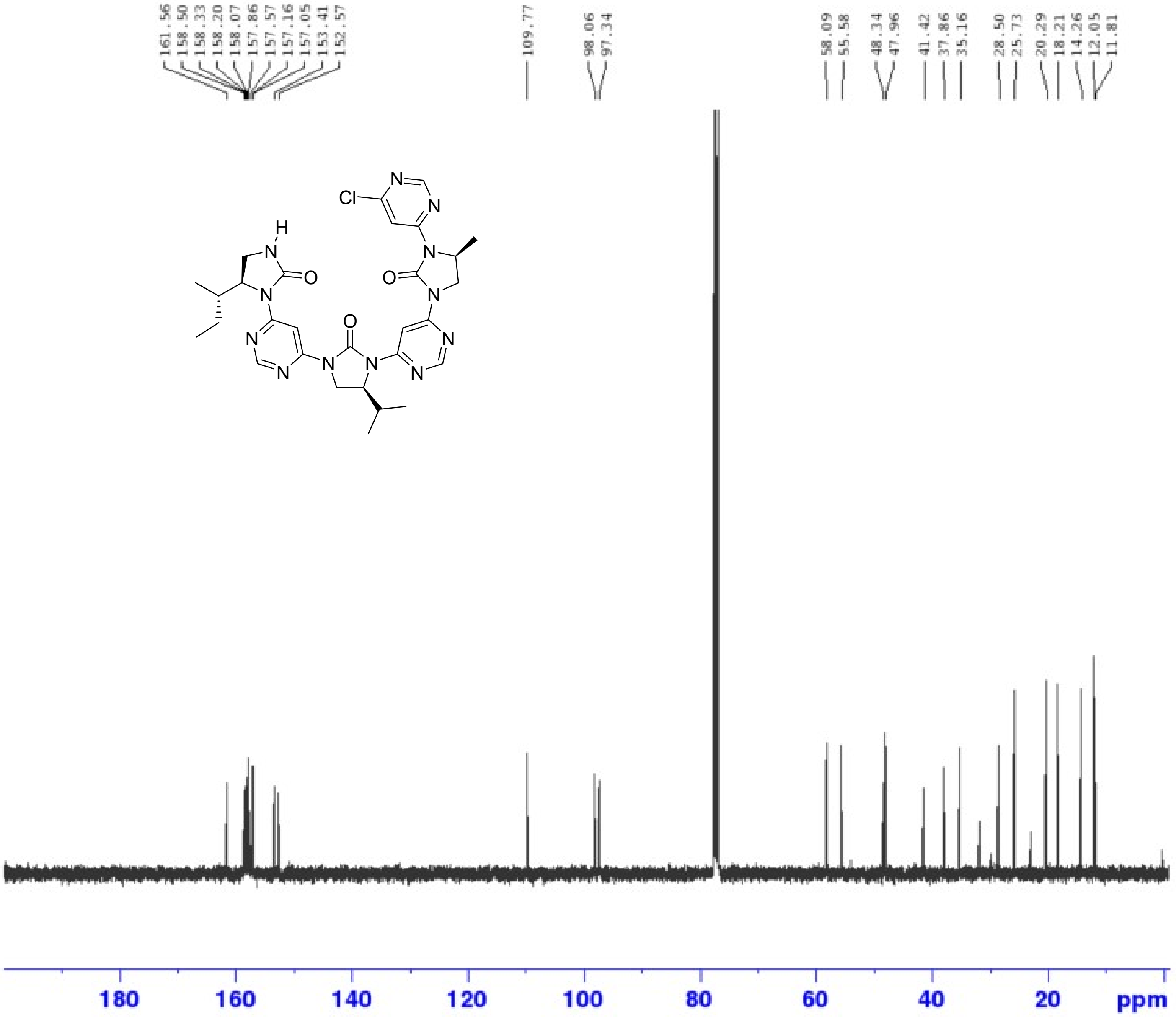

4b

^{13}C NMR

101 MHz

$CDCl_3$

Current Data Parameters

NAME	TW-C-408
EXPNO	11
PROCNO	1

F2 - Acquisition Parameters

Date_	20201216
Time	20.49
INSTRUM	AVIII_400
PROBHD	5 mm PABBO BB/
PULPROG	zgpg30
TD	96150
SOLVENT	CDCl3
NS	1024
DS	4
SWH	24038.461 Hz
FIDRES	0.250010 Hz
AQ	1.9999200 sec
RG	144
DW	20.800 usec
DE	6.50 usec
TE	300.3 K
D1	1.00000000 sec
D11	0.03000000 sec
TD0	1

======== CHANNEL f1 ========

SFO1	100.5675047 MHz
NUC1	13C
P1	9.00 usec
PLW1	96.68000031 W

======== CHANNEL f2 ========

SFO2	399.9115996 MHz
NUC2	1H
CPDPRG[2	waltz64
PCPD2	90.00 usec
PLW2	17.29199982 W
PLW12	0.48032999 W
PLW13	0.38907000 W

F2 - Processing parameters

SI	131072
SF	100.5574351 MHz
WDW	EM
SSB	0
LB	1.00 Hz
GB	0
PC	1.40

3a

^{1}H NMR

600 MHz

$CDCl_3$

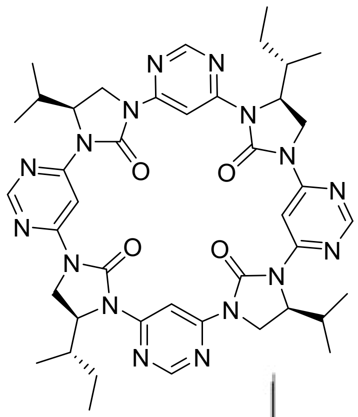

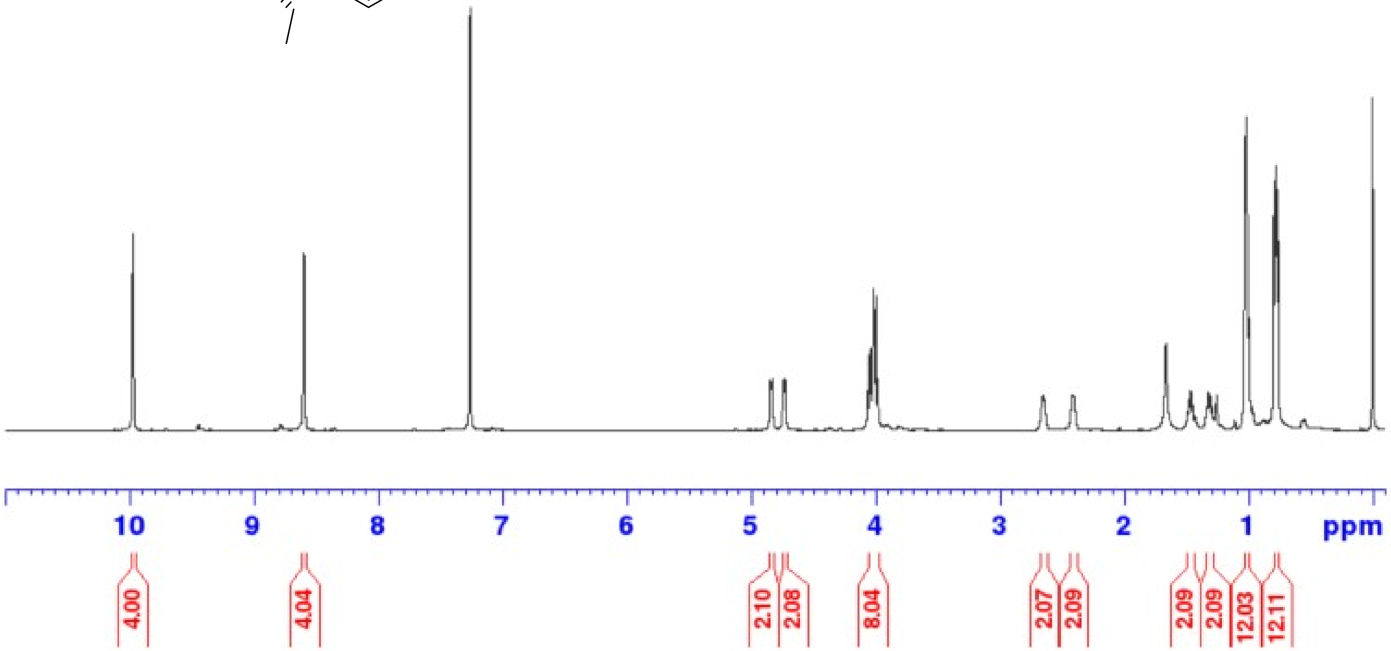


```
Current Data Parameters
NAME       TW-A-186 600 31-08-20
EXPNO                      10
PROCNO                      1

F2 - Acquisition Parameters
Date_               20200829
Time                    6.50
INSTRUM                spect
PROBHD    5 mm PABBO BB/
PULPROG                 zg30
TD                     65536
SOLVENT                CDCl3
NS                        64
DS                         2
SWH                12019.230 Hz
FIDRES              0.183399 Hz
AQ                 2.7262976 sec
RG                    148.05
DW                    41.600 usec
DE                      6.50 usec
TE                     300.0 K
D1                1.00000000 sec
TD0                        1

======== CHANNEL f1 ========
SFO1          600.1337060 MHz
NUC1                   1H
P1                  10.00 usec
PLW1          26.60000038 W

F2 - Processing parameters
SI                     65536
SF            600.1300146 MHz
WDW                       EM
SSB                        0
LB                      0.30 Hz
GB                         0
PC                      1.00
```

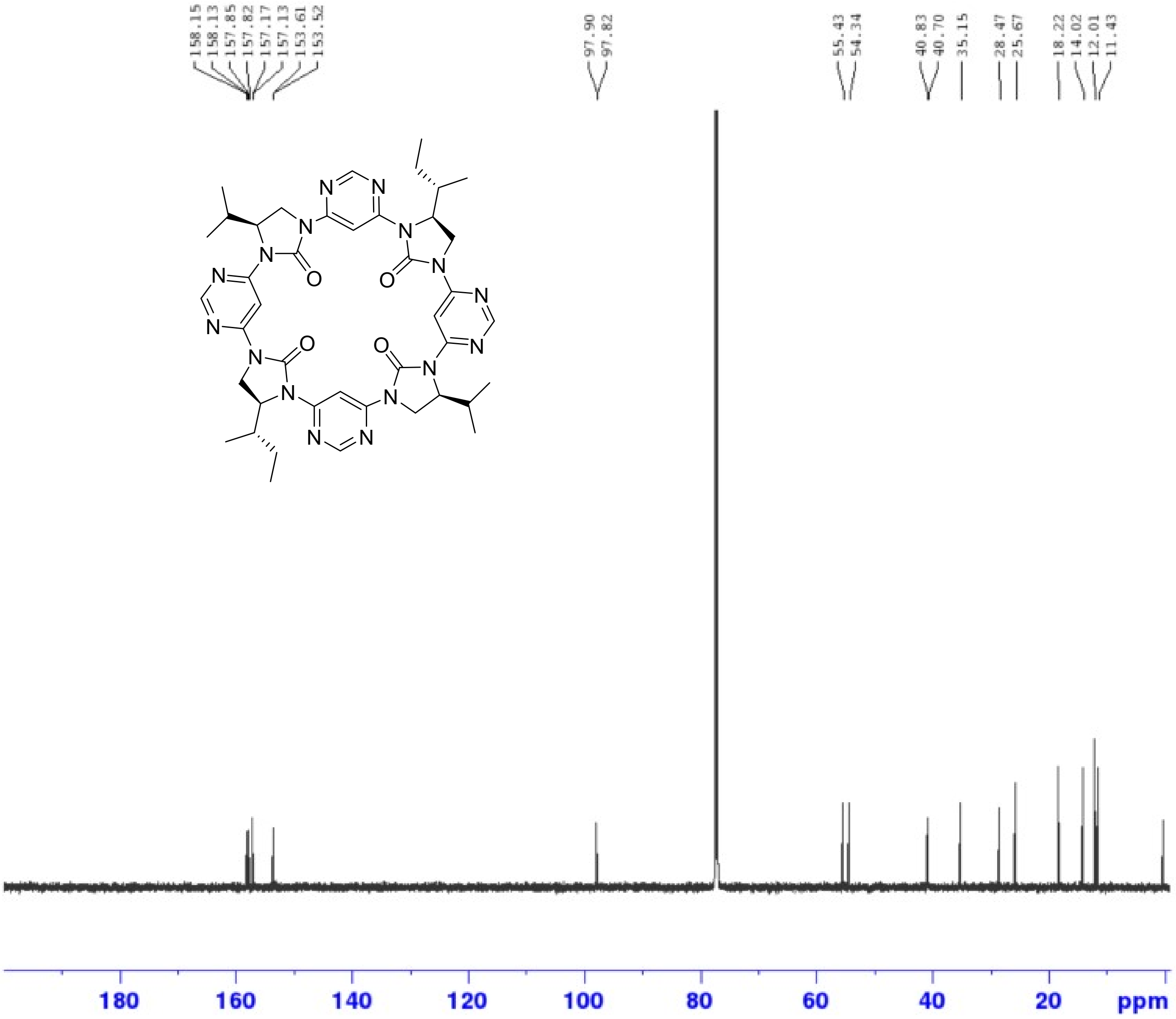

3a

^{13}C NMR

151 MHz

$CDCl_3$

```
Current Data Parameters
NAME        TW-A-186 600 31-08-20
EXPNO                  17
PROCNO                  1

F2 - Acquisition Parameters
Date_            20200830
Time                 4.48
INSTRUM             spect
PROBHD     5 mm PABBO BB/
PULPROG           zgpg30
TD                119044
SOLVENT            CDCl3
NS                  4000
DS                     4
SWH            37500.000 Hz
FIDRES          0.315010 Hz
AQ             1.5872533 sec
RG                186.92
DW                13.333 usec
DE                  7.73 usec
TE                 300.0 K
D1            1.00000000 sec
D11           0.03000000 sec
TD0                    1

======== CHANNEL f1 ========
SFO1         150.9194058 MHz
NUC1                 13C
P1                 11.80 usec
PLW1         85.00000000 W

======== CHANNEL f2 ========
SFO2         600.1324005 MHz
NUC2                  1H
CPDPRG[2         waltz64
PCPD2              80.00 usec
PLW2         27.00000000 W
PLW12         0.43891999 W
PLW13         0.28090999 W

F2 - Processing parameters
SI                131072
SF           150.9027870 MHz
WDW                   EM
SSB                    0
LB                  1.00 Hz
GB                     0
PC                  1.40
```

3b

^{1}H NMR

600 MHz

$CDCl_3$

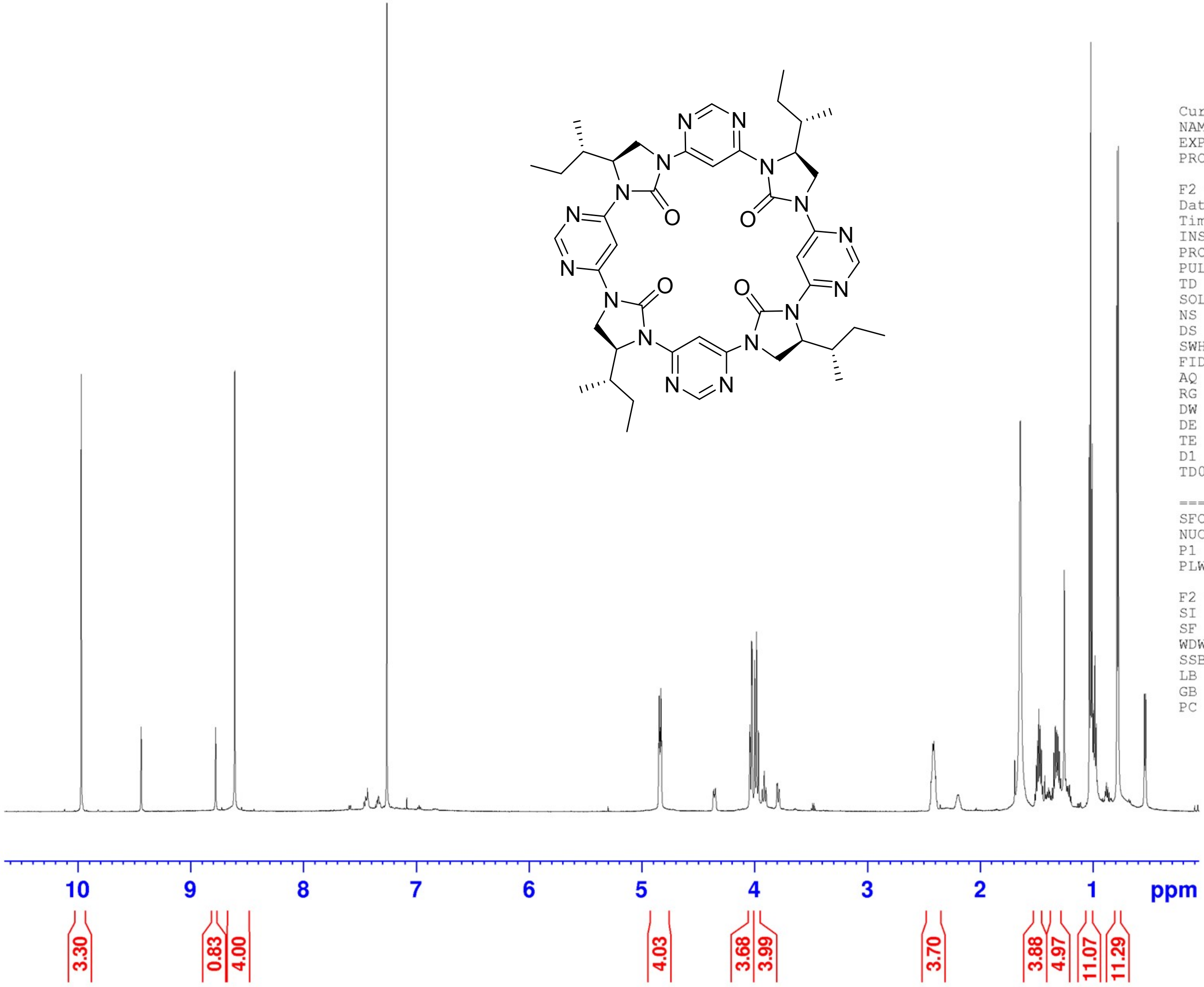


```
Current Data Parameters
NAME      TW-A-250 600 31-08-20
EXPNO                 10
PROCNO                 1

F2 - Acquisition Parameters
Date_           20200829
Time               11.38
INSTRUM            spect
PROBHD   5 mm PABBO BB/
PULPROG             zg30
TD                 65536
SOLVENT            CDCl3
NS                    64
DS                     2
SWH            12019.230 Hz
FIDRES          0.183399 Hz
AQ             2.7262976 sec
RG                168.12
DW                41.600 usec
DE                  6.50 usec
TE                 300.0 K
D1            1.00000000 sec
TD0                    1

======== CHANNEL f1 ========
SFO1         600.1337060 MHz
NUC1                  1H
P1                 10.00 usec
PLW1         26.60000038 W

F2 - Processing parameters
SI                 65536
SF           600.1300132 MHz
WDW                   EM
SSB                    0
LB                  0.30 Hz
GB                     0
PC                  1.00
```

3b

^{13}C NMR

151 MHz

$CDCl_3$

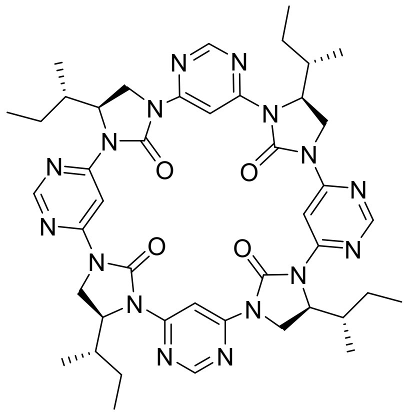

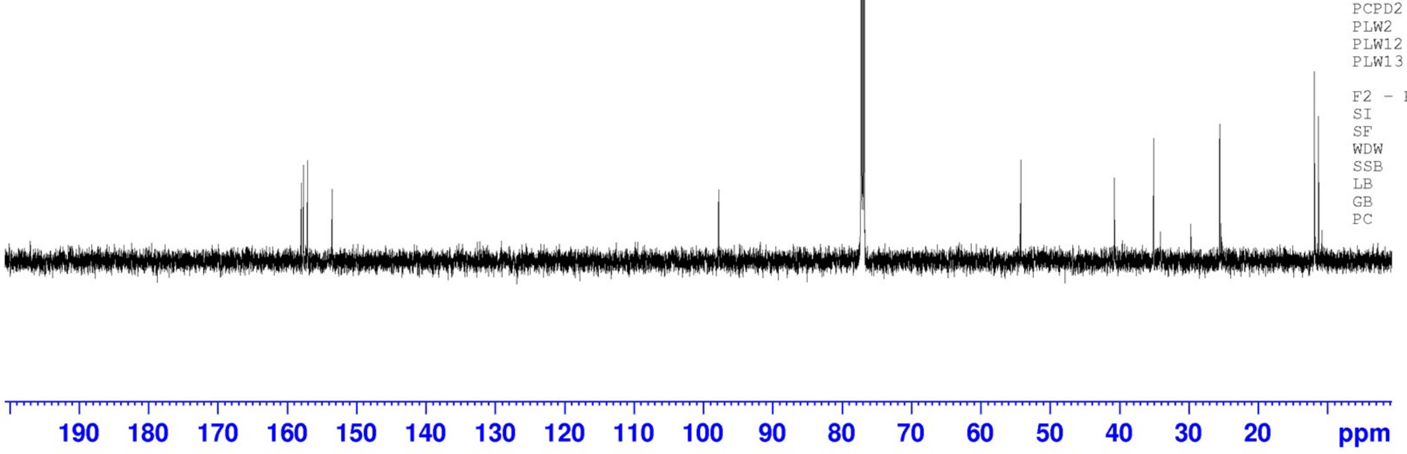


```
Current Data Parameters
NAME     TW-A-250 600 31-08-20
EXPNO                  12
PROCNO                  1

F2 - Acquisition Parameters
Date_            20200829
Time                13.28
INSTRUM             spect
PROBHD   5 mm PABBO BB/
PULPROG            zgpg30
TD                  65536
SOLVENT             CDCl3
NS                   1024
DS                      4
SWH             36057.691 Hz
FIDRES           0.550197 Hz
AQ              0.9087659 sec
RG                 186.92
DW                 13.867 usec
DE                   6.50 usec
TE                  300.0 K
D1             2.00000000 sec
D11            0.03000000 sec
TD0                     1

======== CHANNEL f1 ========
SFO1         150.9178981 MHz
NUC1                  13C
P1                  11.80 usec
PLW1          85.00000000 W

======== CHANNEL f2 ========
SFO2         600.1324005 MHz
NUC2                   1H
CPDPRG[2          waltz16
PCPD2               80.00 usec
PLW2          27.00000000 W
PLW12          0.43891999 W
PLW13          0.28090999 W

F2 - Processing parameters
SI                  32768
SF           150.9028085 MHz
WDW                    EM
SSB                     0
LB                   1.00 Hz
GB                      0
PC                   1.40
```

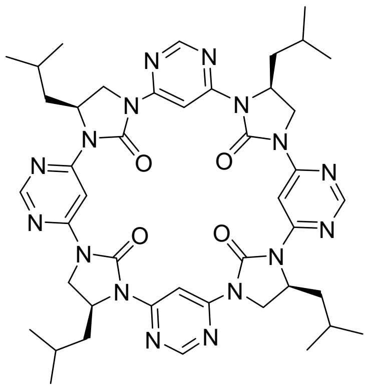

3c

^{1}H NMR

600 MHz

$CDCl_3$

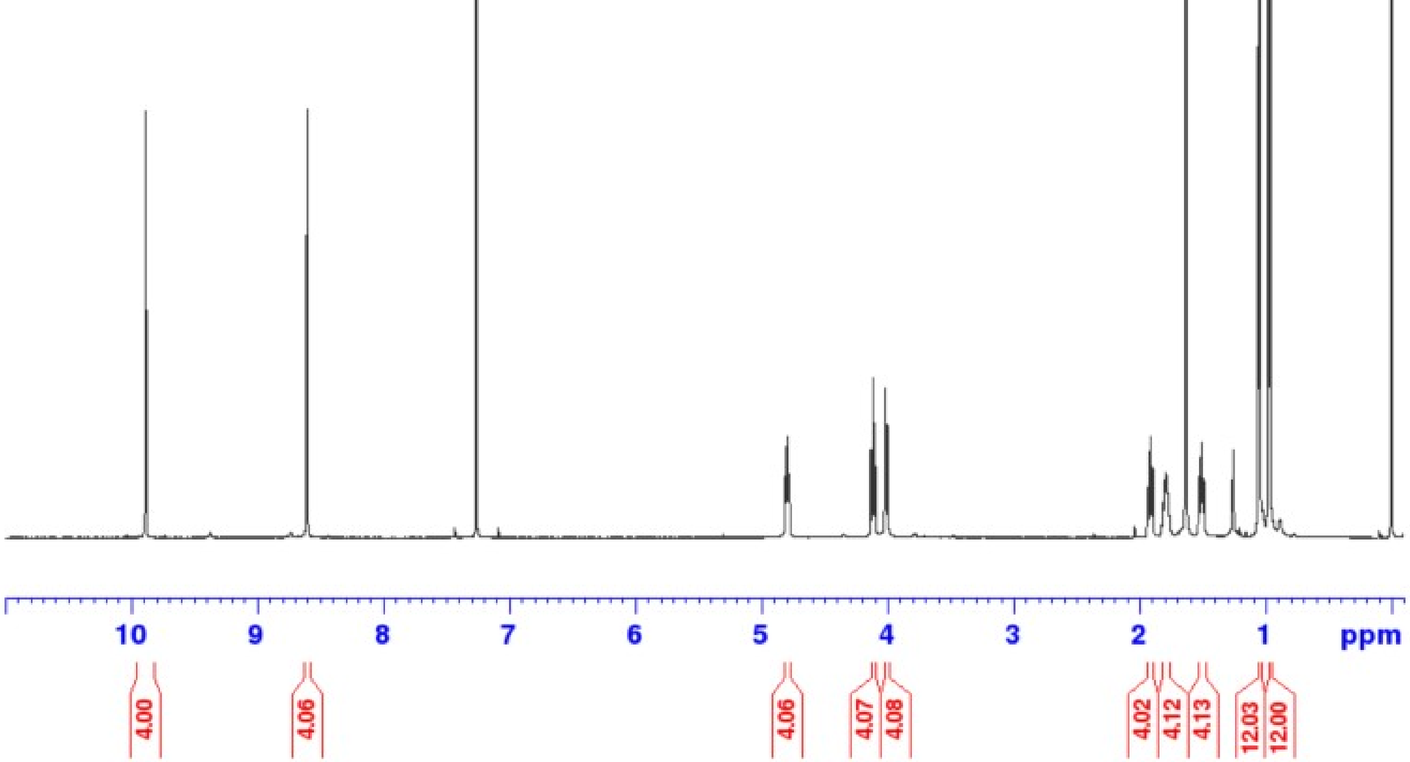


```
Current Data Parameters
NAME      TW-A-167-A 600 31-08-20
EXPNO                  10
PROCNO                 1

F2 - Acquisition Parameters
Date_          20200828
Time              21.15
INSTRUM           spect
PROBHD    5 mm PABBO BB/
PULPROG            zg30
TD                65536
SOLVENT           CDCl3
NS                   64
DS                    2
SWH           12019.230 Hz
FIDRES         0.183399 Hz
AQ            2.7262976 sec
RG               105.21
DW               41.600 usec
DE                 6.50 usec
TE                300.0 K
D1           1.00000000 sec
TD0                   1

======== CHANNEL f1 ========
SFO1        600.1337060 MHz
NUC1                 1H
P1                10.00 usec
PLW1        26.60000038 W

F2 - Processing parameters
SI                65536
SF          600.1300144 MHz
WDW                  EM
SSB                   0
LB                 0.30 Hz
GB                    0
PC                 1.00
```

3c

^{13}C NMR

151 MHz

$CDCl_3$

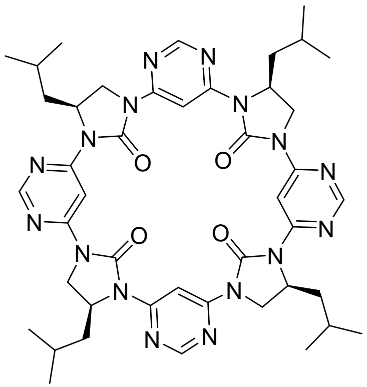

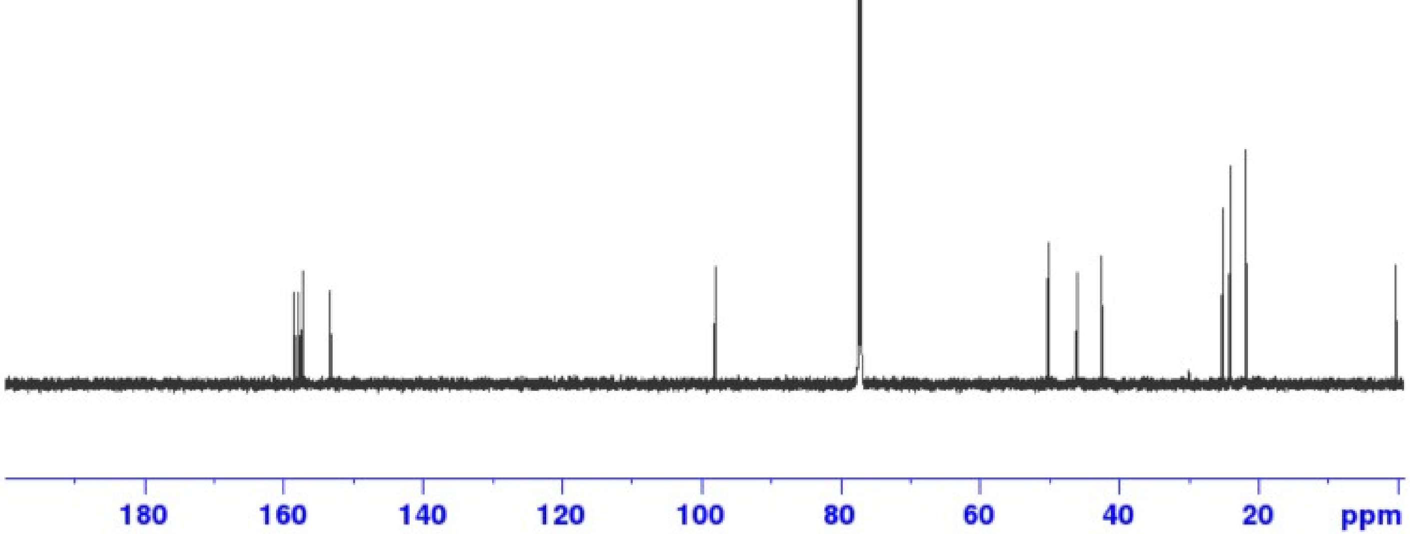


```
Current Data Parameters
NAME       TW-A-167-A 600 31-08-20
EXPNO                   17
PROCNO                   1

F2 - Acquisition Parameters
Date_             20200830
Time                  0.05
INSTRUM              spect
PROBHD    5 mm PABBO BB/
PULPROG             zgpg30
TD                  119044
SOLVENT              CDCl3
NS                    4096
DS                       4
SWH              37500.000 Hz
FIDRES            0.315010 Hz
AQ               1.5872533 sec
RG                  186.92
DW                  13.333 usec
DE                    7.73 usec
TE                   300.0 K
D1              1.00000000 sec
D11             0.03000000 sec
TD0                      1

======== CHANNEL f1 ========
SFO1           150.9194058 MHz
NUC1                   13C
P1                   11.80 usec
PLW1           85.00000000 W

======== CHANNEL f2 ========
SFO2           600.1324005 MHz
NUC2                    1H
CPDPRG[2           waltz64
PCPD2                80.00 usec
PLW2           27.00000000 W
PLW12           0.43891999 W
PLW13           0.28090999 W

F2 - Processing parameters
SI                  131072
SF             150.9027869 MHz
WDW                     EM
SSB                      0
LB                    1.00 Hz
GB                       0
PC                    1.40
```

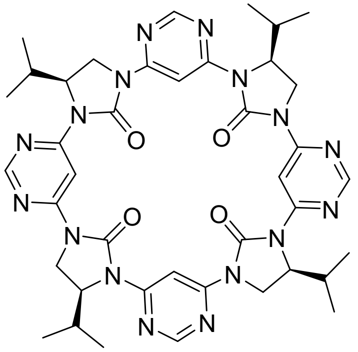

3d

^{1}H NMR

600 MHz

$CDCl_3$

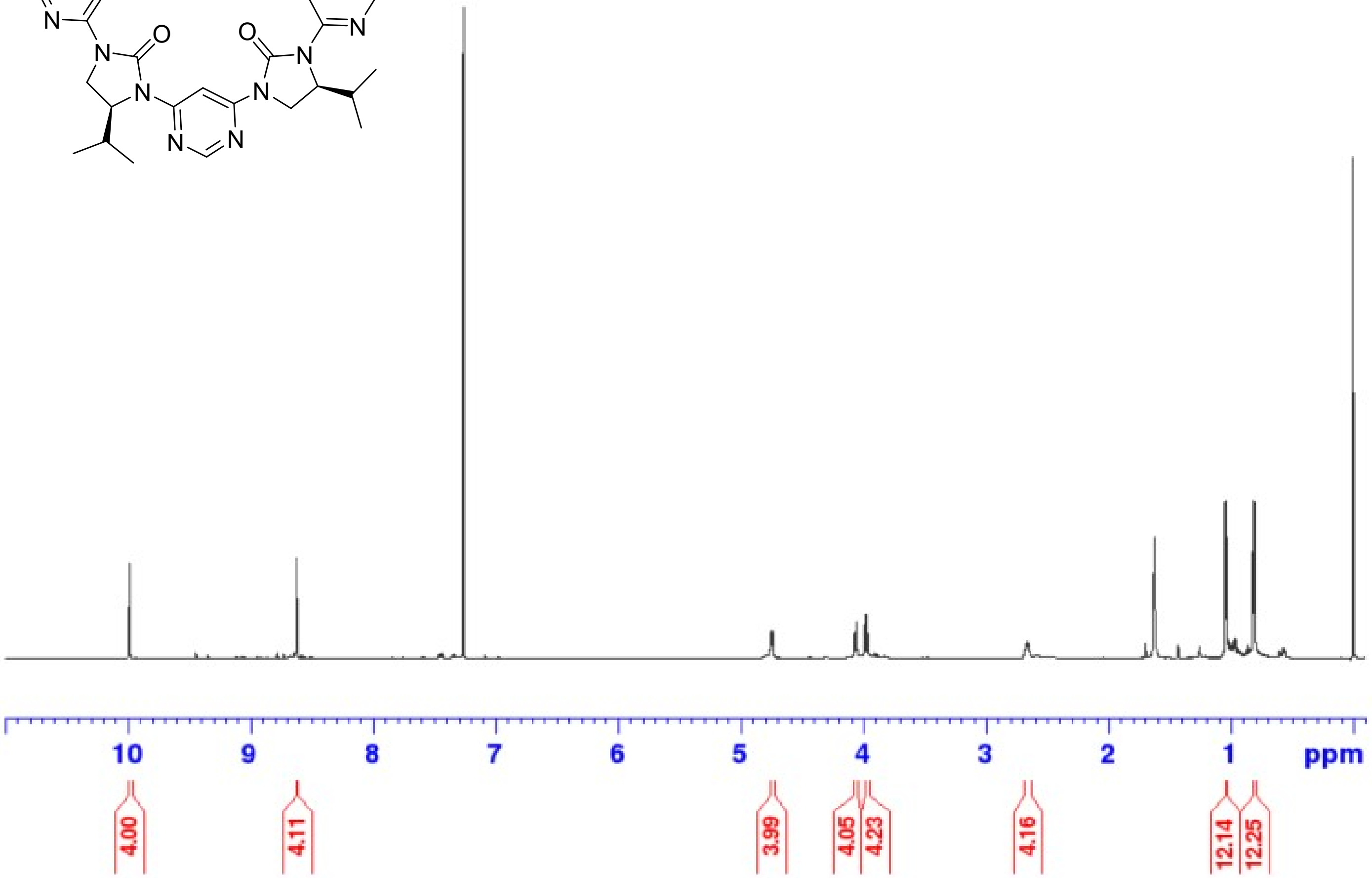

Current Data Parameters
NAME TW-A-238 600 31-08-20
EXPNO 10
PROCNO 1

F2 - Acquisition Parameters
Date_ 20200829
Time 2.02
INSTRUM spect
PROBHD 5 mm PABBO BB/
PULPROG zg30
TD 65536
SOLVENT CDCl3
NS 64
DS 2
SWH 12019.230 Hz
FIDRES 0.183399 Hz
AQ 2.7262976 sec
RG 105.21
DW 41.600 usec
DE 6.50 usec
TE 300.0 K
D1 1.00000000 sec
TD0 1

======== CHANNEL f1 ========
SFO1 600.1337060 MHz
NUC1 1H
P1 10.00 usec
PLW1 26.60000038 W

F2 - Processing parameters
SI 65536
SF 600.1300147 MHz
WDW EM
SSB 0
LB 0.30 Hz
GB 0
PC 1.00

3d

^{13}C NMR

151 MHz

$CDCl_3$

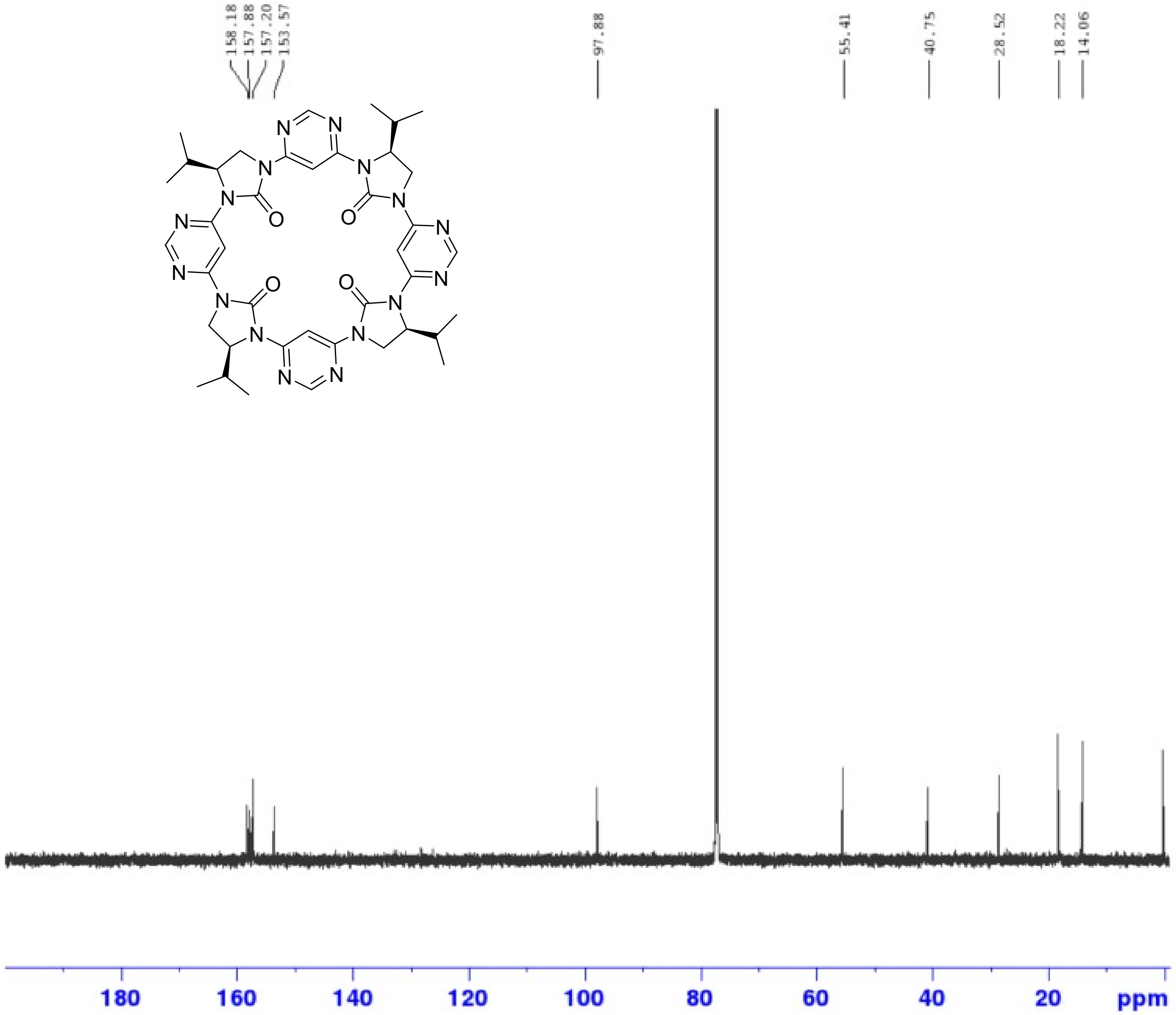


```
Current Data Parameters
NAME       TW-A-238 600 31-08-20
EXPNO                    17
PROCNO                    1

F2 - Acquisition Parameters
Date_              20200830
Time                   9.29
INSTRUM               spect
PROBHD    5 mm PABBO BB/
PULPROG             zgpg30
TD                  119044
SOLVENT              CDCl3
NS                    4000
DS                       4
SWH              37500.000 Hz
FIDRES            0.315010 Hz
AQ               1.5872533 sec
RG                  186.92
DW                  13.333 usec
DE                    7.73 usec
TE                   300.0 K
D1              1.00000000 sec
D11             0.03000000 sec
TD0                      1

======== CHANNEL f1 ========
SFO1          150.9194058 MHz
NUC1                  13C
P1                  11.80 usec
PLW1          85.00000000 W

======== CHANNEL f2 ========
SFO2          600.1324005 MHz
NUC2                   1H
CPDPRG[2           waltz64
PCPD2               80.00 usec
PLW2          27.00000000 W
PLW12          0.43891999 W
PLW13          0.28090999 W

F2 - Processing parameters
SI                  131072
SF            150.9027867 MHz
WDW                     EM
SSB                      0
LB                    1.00 Hz
GB                       0
PC                    1.40
```

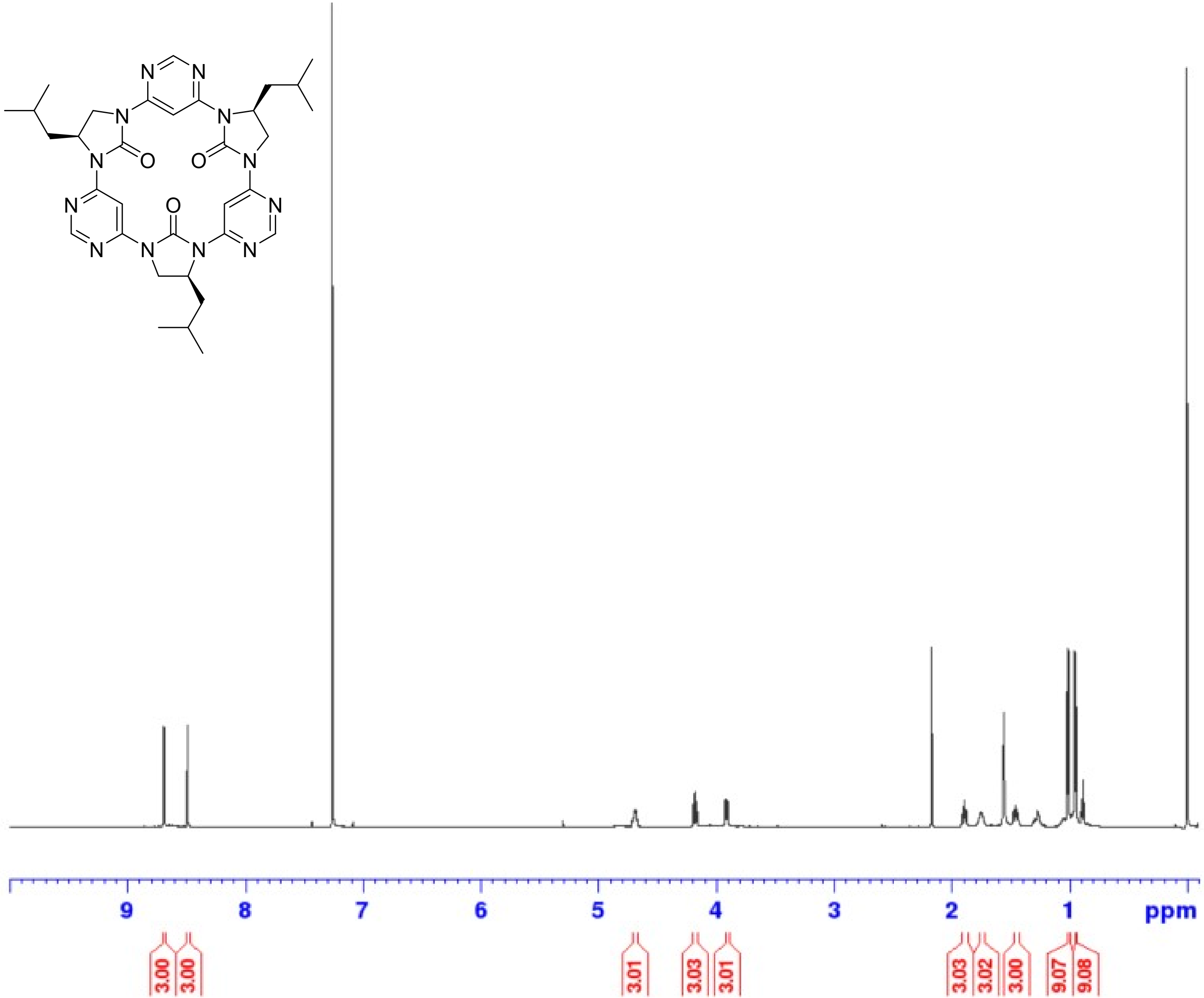

5a

^{1}H NMR

600 MHz

$CDCl_3$

```
Current Data Parameters
NAME        TW-B-404 pure Full
EXPNO                       10
PROCNO                       1

F2 - Acquisition Parameters
Date_                 20201215
Time                      3.23
INSTRUM                  spect
PROBHD      5 mm PABBO BB/
PULPROG                   zg30
TD                       65536
SOLVENT                  CDCl3
NS                         256
DS                           2
SWH              12019.230 Hz
FIDRES            0.183399 Hz
AQ               2.7262976 sec
RG                      186.92
DW                 41.600 usec
DE                   6.50 usec
TE                    300.0 K
D1              1.00000000 sec
TD0                          1

======== CHANNEL f1 ========
SFO1          600.1337060 MHz
NUC1                        1H
P1                 10.00 usec
PLW1           26.60000038 W

F2 - Processing parameters
SI                       65536
SF            600.1300146 MHz
WDW                         EM
SSB                          0
LB                     0.30 Hz
GB                           0
PC                        1.00
```

5a

^{13}C NMR

151 MHz

$CDCl_3$

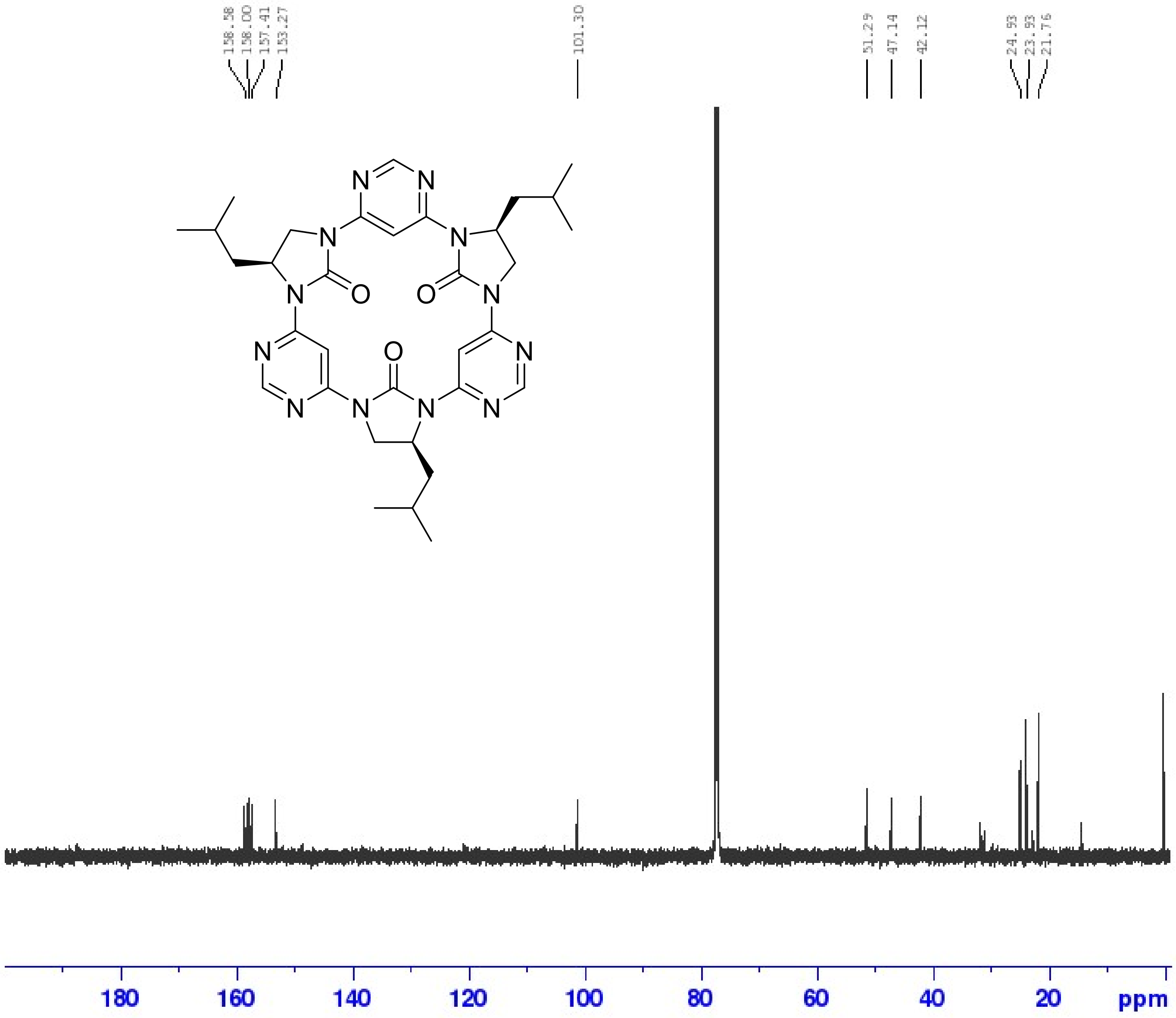


```
Current Data Parameters
NAME        TW-B-404 pure Full
EXPNO                       11
PROCNO                       1

F2 - Acquisition Parameters
Date_                20201215
Time                     8.24
INSTRUM                 spect
PROBHD     5 mm PABBO BB/
PULPROG               zgpg30
TD                    119044
SOLVENT                CDCl3
NS                      8192
DS                         4
SWH                37500.000 Hz
FIDRES              0.315010 Hz
AQ                 1.5872533 sec
RG                    186.92
DW                    13.333 use
DE                      7.73 use
TE                     300.0 K
D1                1.00000000 sec
D11               0.03000000 sec
TD0                        1

======== CHANNEL f1 =======
SFO1             150.9194058 MHz
NUC1                     13C
P1                     11.80 use
PLW1             85.00000000 W

======== CHANNEL f2 =======
SFO2             600.1324005 MHz
NUC2                      1H
CPDPRG[2             waltz64
PCPD2                  80.00 use
PLW2             27.00000000 W
PLW12             0.43891999 W
PLW13             0.28090999 W

F2 - Processing parameters
SI                    131072
SF               150.9027863 MHz
WDW                       EM
SSB                        0
LB                      1.00 Hz
GB                         0
PC                      1.40
```

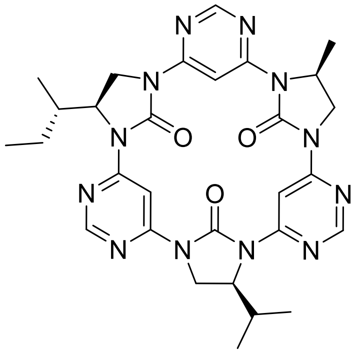

6a

^{1}H NMR

400 MHz

$CDCl_3$

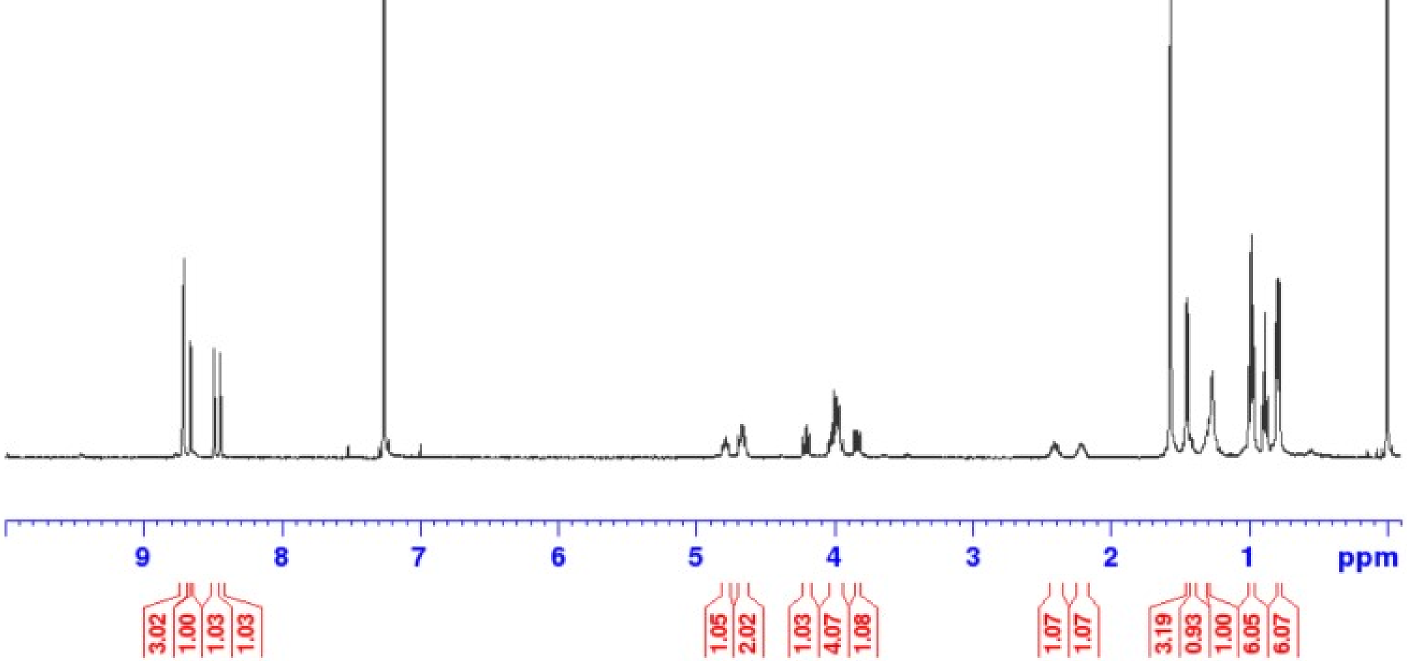


```
Current Data Parameters
NAME        TW-C-409-TRIT
EXPNO                  10
PROCNO                  1

F2 - Acquisition Parameters
Date_            20201217
Time                14.52 h
INSTRUM         AVIII_400
PROBHD   Z108618_0146 (
PULPROG              zg30
TD                  65536
SOLVENT             CDCl3
NS                     16
DS                      2
SWH              8223.685 Hz
FIDRES           0.250967 Hz
AQ              3.9845889 sec
RG                    456
DW                 60.800 usec
DE                  17.42 usec
TE                  300.0 K
D1             1.00000000 sec
TD0                     1
SFO1          400.1124708 MHz
NUC1                   1H
P0                   5.00 usec
P1                  15.00 usec
PLW1          17.29199982 W

F2 - Processing parameters
SI                  32768
SF            400.1100095 MHz
WDW                    EM
SSB                     0
LB                   0.30 Hz
GB                      0
PC                   1.00
```

6a

^{13}C NMR

151 MHz

$CDCl_3$

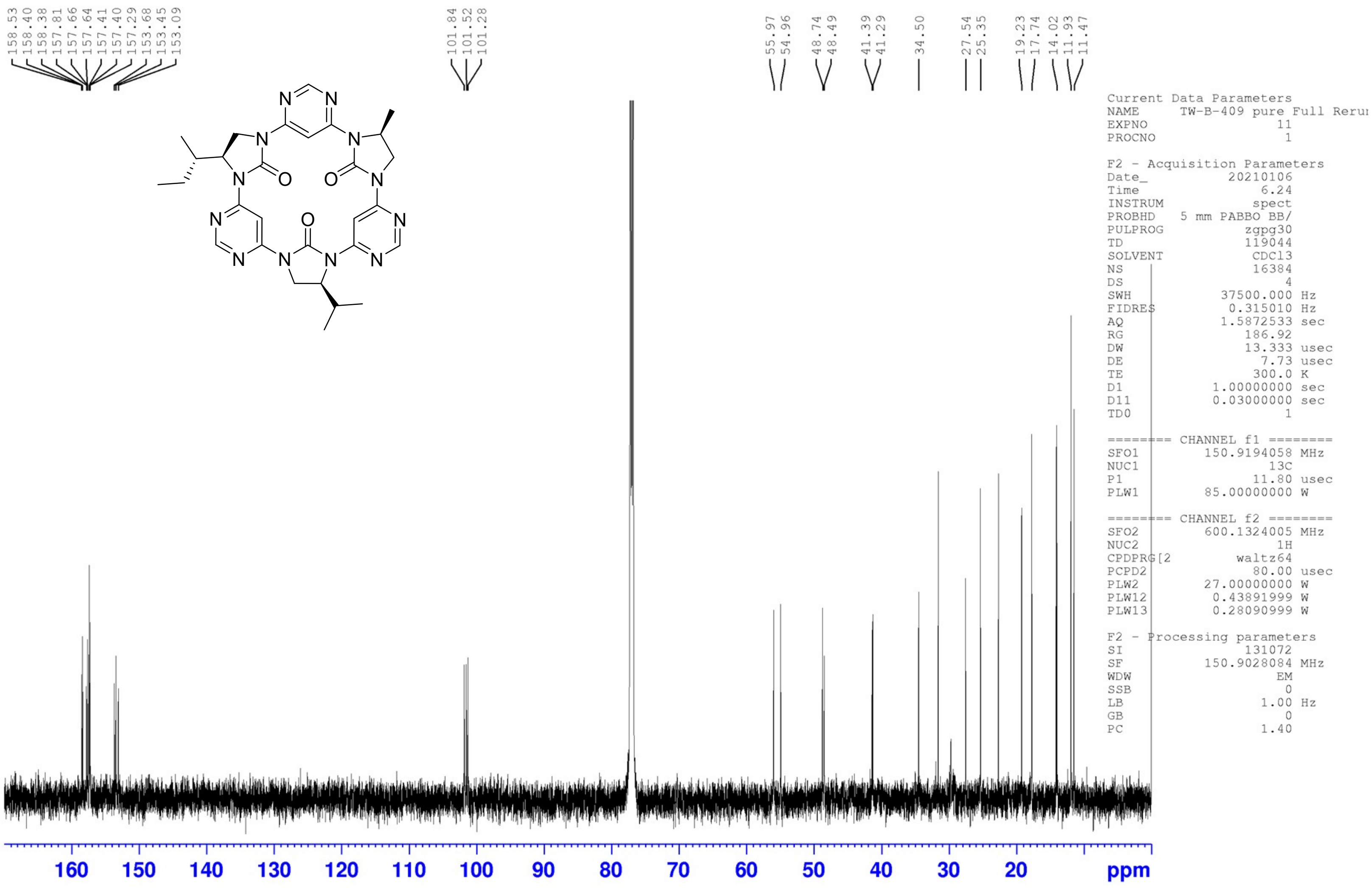

8 Appendix – Coordinates for DFT-Optimized Geometries

8.1 Trimer Coordinates (Figure 3B)

R=Me, Pseudo-Axial

N	4.486655	-0.59285	-0.55038
C	3.404083	-0.71712	0.241927
C	2.767509	0.380589	0.817981
C	3.190042	1.635894	0.364419
N	4.271136	1.789424	-0.41998
C	4.873436	0.658887	-0.79518
N	2.436773	2.78761	0.619049
C	1.123257	2.682348	1.127182
N	0.305771	3.507017	0.353718
C	1.074351	4.140241	-0.74675
C	2.519316	3.979568	-0.23237
C	-1.0811	3.306276	0.241582
C	-1.71384	2.206601	0.81821
C	-3.01195	1.944439	0.363992
C	-3.00679	3.889864	-0.79723
N	-1.72957	4.181046	-0.55179
N	-3.63313	0.716485	0.61893
C	-2.8859	-0.36867	1.127712
N	-3.19107	-1.48894	0.35405
C	-4.12239	-1.13944	-0.74738
C	-4.70634	0.191955	-0.23292
N	2.884538	-2.01857	0.353968
C	3.048946	-3.00077	-0.74648
C	2.186287	-4.17143	-0.23289
N	1.195391	-3.50384	0.618575
C	1.761192	-2.31394	1.127002
C	-0.17867	-3.58035	0.364038
N	-0.58576	-4.59254	-0.4217
C	-1.86582	-4.54871	-0.79735
N	-2.7567	-3.58831	-0.55183
C	-2.32363	-2.58953	0.241945
C	-1.05497	-2.5876	0.81869
N	-3.68492	2.803299	-0.42156
O	1.328167	-1.66796	2.05803
O	-2.11046	-0.31683	2.05917
O	0.780319	1.9842	2.058099
H	1.937554	0.267078	1.49188
H	5.77273	0.773816	-1.39645
H	0.785121	5.188637	-0.82647
H	3.241298	3.817124	-1.03021
H	2.835678	4.836565	0.369637
H	-1.2011	1.545202	1.493116
H	-3.55564	4.610757	-1.39935
H	-4.8855	-1.91415	-0.82854
H	-5.60682	0.037042	0.368813
H	-4.92657	0.898568	-1.03066
H	4.101425	-3.27502	-0.82537
H	2.769624	-4.87466	0.368879
H	1.684421	-4.71471	-1.03113
H	-2.21554	-5.38437	-1.39971
H	-0.73874	-1.81297	1.493825
C	0.866887	3.421166	-2.08077
H	1.175776	2.373465	-2.00188
H	-0.17759	3.462976	-2.38984
H	1.475824	3.899473	-2.85348
C	-3.39398	-0.9587	-2.08019
H	-2.90746	-1.88392	-2.38921
H	-4.11153	-0.67	-2.8538
H	-2.64122	-0.16741	-1.99954
C	2.531426	-2.4612	-2.0809
H	1.469806	-2.20392	-2.00292
H	3.090865	-1.57805	-2.38955
H	2.641185	-3.22786	-2.85346

R=Me, Pseudo-Equatorial

N	-0.20656	-4.5456	-0.73496
C	-0.42198	-3.50851	0.095925
C	0.619429	-2.75128	0.63898
C	1.885489	-2.99503	0.101315
N	2.130956	-4.0265	-0.72936
C	1.068298	-4.76024	-1.0615
N	2.955753	-2.11171	0.298052
C	2.77548	-0.86896	0.917695
N	3.619973	0.052035	0.286534
C	4.549871	-0.61274	-0.62916
C	4.106636	-2.10238	-0.62377
C	3.249669	1.388843	0.096257
C	2.072979	1.911887	0.639062
C	1.651064	3.130234	0.101422
C	3.588592	3.305426	-1.06098
N	4.040228	2.094104	-0.73439
N	0.350915	3.615428	0.29796
C	-0.63527	2.838048	0.917584
N	-1.85502	3.108862	0.286219
C	-1.74411	4.24633	-0.62975
C	-0.23246	4.607443	-0.62395
N	-1.76484	-3.16079	0.286283
C	-2.80565	-3.6338	-0.62918
C	-3.87407	-2.5051	-0.62367
N	-3.30671	-1.50384	0.298269
C	-2.14027	-1.96916	0.917783

C	-3.53658	-0.13528	0.101572
N	-4.5527	0.167825	-0.72899
C	-4.65691	1.454967	-1.06113
N	-3.83362	2.451752	-0.7347
C	-2.82766	2.119812	0.096069
C	-2.69246	0.839344	0.639142
N	2.421697	3.858677	-0.72905
O	-1.54376	-1.42226	1.822178
O	-0.45985	2.048087	1.822047
O	2.003454	-0.62588	1.821999
H	0.44113	-1.9584	1.342231
H	1.261341	-5.62181	-1.69713
H	5.578129	-0.50072	-0.27432
H	4.486941	-0.15733	-1.6184
H	3.751291	-2.40421	-1.61183
H	1.475468	1.36081	1.342147
H	4.238286	3.903518	-1.69642
H	-2.10666	3.963774	-1.61903
H	-2.35549	5.080841	-0.27539
H	0.206886	4.450732	-1.61191
H	-2.37997	-3.80711	-1.61847
H	-3.2227	-4.58027	-0.27414
H	-3.95778	-2.04637	-1.6117
H	-5.49969	1.718528	-1.69664
H	-1.91658	0.597481	1.342366
C	0.046961	6.035077	-0.16996
H	1.122265	6.221138	-0.16936
H	-0.43275	6.746335	-0.85046
H	-0.34527	6.200591	0.838321
C	5.203233	-3.0583	-0.16992
H	4.826699	-4.08257	-0.16947
H	6.05908	-2.9984	-0.85038
H	5.542657	-2.80154	0.838419
C	-5.25023	-2.97677	-0.16985
H	-5.94895	-2.13851	-0.16952
H	-5.62628	-3.74797	-0.85024
H	-5.19762	-3.39899	0.838538

R=iPr, Pseudo-Axial

N	0.725283	-4.5173	0.060649
C	0.817889	-3.39611	-0.68108
C	-0.29699	-2.77332	-1.23843
C	-1.53956	-3.25011	-0.80536
N	-1.66153	-4.36339	-0.06122
C	-0.51398	-4.95031	0.28781
N	-2.71019	-2.5138	-1.012
C	-2.65159	-1.20401	-1.5287
N	-3.4893	-0.40575	-0.75195
C	-4.09268	-1.17234	0.365027
C	-3.85927	-2.62465	-0.10717
C	-3.34976	0.990755	-0.68219
C	-2.2529	1.645207	-1.23919
C	-2.04456	2.959473	-0.80529
C	-4.0301	2.921044	0.287151
N	-4.27468	1.631389	0.059375
N	-0.82146	3.605215	-1.01088
C	0.283715	2.900144	-1.52806
N	1.393451	3.225246	-0.75026
C	1.030396	4.128451	0.368511
C	-0.3432	4.654114	-0.10394
N	2.097019	-2.8187	-0.75066
C	3.061776	-2.9567	0.367096
C	4.203375	-2.02954	-0.10585
N	3.533298	-1.09036	-1.01163
C	2.369729	-1.6947	-1.52821
C	3.585498	0.291643	-0.80531
N	4.610602	0.742723	-0.06123
C	4.545152	2.030001	0.287657
N	3.550405	2.88661	0.060575
C	2.533126	2.406194	-0.68115
C	2.551346	1.129341	-1.23866
N	-2.9478	3.621394	-0.06099
O	1.722941	-1.28856	-2.47177
O	0.255491	2.137816	-2.47234
O	-1.97606	-0.84709	-2.47198
H	-0.20711	-1.91314	-1.87704
H	-0.60316	-5.87737	0.849936
H	-5.15849	-0.94277	0.414411
H	-3.62521	-3.31244	0.701948
H	-4.72281	-3.01259	-0.65473
H	-1.55278	1.137606	-1.87795
H	-4.78856	3.461607	0.8492
H	1.762393	4.936237	0.420829
H	-0.2464	5.59682	-0.64979
H	-1.05647	4.794272	0.704818
H	3.395497	-3.99453	0.418248
H	4.970793	-2.58441	-0.65286
H	4.682322	-1.48246	0.702722
H	5.392665	2.416398	0.849613
H	1.76152	0.777045	-1.87733
C	-3.45168	-0.84316	1.733306
H	-3.62642	0.22422	1.893734
C	0.993249	3.404768	1.734735
H	2.004577	3.021179	1.894894
C	2.455051	-2.56385	1.734309
H	1.617666	-3.24837	1.895039
C	-4.17659	-1.59916	2.852758
H	-4.02228	-2.68139	2.781759
H	-3.80189	-1.28168	3.830268
H	-5.25467	-1.40874	2.830514

C	-1.93898	-1.09128	1.775328
H	-1.69597	-2.15582	1.699651
H	-1.41556	-0.56394	0.972717
H	-1.52755	-0.72901	2.722167
C	0.700837	4.407445	2.856946
H	0.786918	3.921117	3.833066
H	1.405697	5.245187	2.83801
H	-0.31305	4.816228	2.786164
C	0.021334	2.219188	1.772489
H	-1.02204	2.541718	1.699426
H	0.130046	1.677495	2.716809
H	0.215095	1.505729	0.966471
C	1.913805	-1.12964	1.773547
H	2.714438	-0.38719	1.698004
H	1.196684	-0.94113	0.96956
H	1.392994	-0.95304	2.719358
C	3.470978	-2.81215	2.855186
H	3.00794	-2.64383	3.831914
H	3.844131	-3.84141	2.835555
H	4.331765	-2.13836	2.783507

R=iPr, Pseudo-Equatorial

N	-0.88425	-4.4648	-0.78082
C	-0.94165	-3.40449	0.046981
C	0.200225	-2.81239	0.590895
C	1.416643	-3.24882	0.058981
N	1.505883	-4.31475	-0.75946
C	0.34428	-4.87741	-1.0951
N	2.601622	-2.53075	0.251046
C	2.613752	-1.27334	0.866481
N	3.581873	-0.49404	0.223985
C	4.339954	-1.27881	-0.75636
C	3.770573	-2.71679	-0.62309
C	3.419332	0.886689	0.045592
C	2.336471	1.579674	0.591063
C	2.105394	2.851152	0.059007
C	4.0507	2.736745	-1.09727
N	4.307862	1.466461	-0.78338
N	0.891095	3.518203	0.251975
C	-0.20373	2.899521	0.867294
N	-1.36291	3.348618	0.225362
C	-1.06271	4.398441	-0.75408
C	0.467417	4.623955	-0.62149
N	-2.2186	-2.85491	0.225889
C	-3.27821	-3.12017	-0.75307
C	-4.23793	-1.90714	-0.6215
N	-3.49251	-0.98768	0.252652
C	-2.40924	-1.62651	0.867839
C	-3.52188	0.397525	0.059944
N	-4.48923	0.852673	-0.75927
C	-4.39554	2.139747	-1.09576
N	-3.42393	2.997472	-0.78185
C	-2.47742	2.517603	0.046807
C	-2.53613	1.233203	0.591981
N	2.982971	3.46136	-0.76047
O	-1.74057	-1.18351	1.778994
O	-0.15434	2.099044	1.778525
O	1.896463	-0.91589	1.778237
H	0.142763	-1.9967	1.28847
H	0.405943	-5.76342	-1.72345
H	5.40765	-1.23286	-0.537
H	4.191273	-0.8647	-1.75579
H	3.418993	-3.08102	-1.59028
H	1.659744	1.122229	1.289671
H	4.786525	3.233172	-1.72637
H	-1.34766	4.063715	-1.75368
H	-1.63602	5.300021	-0.53335
H	0.95811	4.50191	-1.58898
H	-2.84612	-3.20088	-1.75267
H	-3.77286	-4.06695	-0.53131
H	-4.37628	-1.42111	-1.58917
H	-5.19347	2.528981	-1.72461
H	-1.80141	0.875602	1.290238
C	0.879325	5.991677	-0.04398
H	1.971372	5.947403	0.04067
C	4.749596	-3.7571	-0.04587
H	4.16529	-4.68066	0.039566
C	-5.62918	-2.23355	-0.04555
H	-6.13671	-1.26551	0.03802
C	-5.55825	-2.86421	1.348583
H	-6.56553	-3.02832	1.742556
H	-5.05345	-3.83673	1.330734
H	-5.02599	-2.21856	2.052409
C	-6.42198	-3.10255	-1.02818
H	-5.96686	-4.09164	-1.15383
H	-7.44195	-3.25879	-0.66527
H	-6.48542	-2.63273	-2.01544
C	0.523966	7.113547	-1.02599
H	-0.56004	7.213562	-1.15306
H	0.897708	8.074789	-0.66162
H	0.964015	6.934725	-2.01281
C	0.296251	6.244255	1.349783
H	-0.7984	6.292773	1.331017
H	0.656893	7.198547	1.744783
C	5.898104	-4.01064	-1.02866
H	6.526594	-3.1219	-1.1565
H	6.544011	-4.81479	-0.66453
H	5.522462	-4.30266	-2.0151
C	5.260925	-3.37796	1.347385
H	5.851625	-2.45512	1.327665

H	4.435948	-3.23777	2.051151
H	5.906066	-4.16804	1.742755
H	0.589067	5.460172	2.053342

R=ABC, Pseudo-Axial

N	3.201946	3.395137	0.580853
C	2.277033	2.894786	-0.26217
C	2.47673	1.716228	-0.97817
C	3.604435	0.968343	-0.61833
N	4.549392	1.449555	0.208033
C	4.300689	2.65563	0.725197
N	3.73755	-0.37031	-1.00609
C	2.626004	-1.05104	-1.54922
N	2.485564	-2.25153	-0.85595
C	3.541317	-2.42399	0.170627
C	4.549692	-1.32498	-0.24363
C	1.277297	-2.96631	-0.81871
C	0.100779	-2.43669	-1.34611
C	-1.08288	-3.08955	-0.98329
C	0.122068	-4.72384	0.022871
N	1.306427	-4.13902	-0.15536
N	-2.33006	-2.49771	-1.20237
C	-2.42585	-1.1499	-1.59694
N	-3.40901	-0.5473	-0.81373
C	-4.03618	-1.5142	0.121934
C	-3.52529	-2.86394	-0.44021
N	1.048499	3.577167	-0.28361
C	0.55965	4.29889	0.916852
C	-0.84855	4.729193	0.456711
N	-1.19569	3.710886	-0.53989
C	-0.01266	3.192285	-1.10377
C	-2.35307	2.930403	-0.44417
N	-3.33128	3.402171	0.349134
C	-4.34721	2.562637	0.563267
N	-4.45222	1.291527	0.174441
C	-3.44721	0.83991	-0.60028
C	-2.42402	1.673049	-1.05147
N	-1.08278	-4.25688	-0.31513
O	0.055988	2.523909	-2.11465
O	-1.7494	-0.61679	-2.45226
O	1.91837	-0.63975	-2.44468
H	1.752987	1.354486	-1.68591
H	5.077274	3.069805	1.364583
H	3.977727	-3.41823	0.05668
H	5.012925	-0.82204	0.601902
H	5.338176	-1.72606	-0.88644
H	0.096058	-1.53329	-1.92791
H	0.135372	-5.68784	0.52674
H	-5.1196	-1.43755	0.010063
H	-4.25862	-3.32598	-1.10727
H	-3.26308	-3.58148	0.333639
H	1.204856	5.157662	1.104566
H	-0.8404	5.717162	-0.01358
H	-1.58005	4.726019	1.262739
H	-5.17357	2.952856	1.153352
H	-1.66389	1.326399	-1.72677
C	3.022493	-2.27499	1.617885
H	2.222102	-3.01111	1.728475
C	-3.70234	-1.30859	1.618785
H	-4.01838	-0.292	1.867084
C	0.503579	3.388438	2.144778
H	-0.15322	2.532964	1.956918
H	1.496866	3.021454	2.404401
H	0.106594	3.945582	2.998494
C	4.131528	-2.64628	2.609101
H	4.976523	-1.95073	2.55402
H	3.752047	-2.61514	3.634669
H	4.511535	-3.65617	2.423281
C	2.4498	-0.8851	1.914614
H	3.228649	-0.11671	1.928418
H	1.696694	-0.58935	1.179544
H	1.970218	-0.88127	2.898249
C	-4.57157	-2.27134	2.442632
H	-4.4655	-2.05919	3.510198
H	-5.6315	-2.17324	2.186609
H	-4.28364	-3.31742	2.289203
C	-2.21896	-1.45671	2.00969
H	-1.82158	-2.41728	1.659051
H	-2.19145	-1.51606	3.104558
C	-1.29006	-0.31478	1.581007
H	-1.05271	-0.32791	0.515164
H	-1.73827	0.658351	1.807351
H	-0.34287	-0.37693	2.121368

R=ABC, Pseudo-Equatorial

N	4.497552	1.769752	-0.69241
C	3.586777	1.20144	0.119871
C	2.513658	1.913652	0.661891
C	2.314683	3.193537	0.137097
N	3.211552	3.788562	-0.6724
C	4.269465	3.046445	-1.00109
N	1.11314	3.892942	0.321177
C	-0.00707	3.278052	0.895831
N	-1.14621	3.764579	0.244141
C	-0.82392	4.903835	-0.61661
C	0.728332	4.977985	-0.6018
C	-2.26275	2.956756	-0.00287
C	-2.34834	1.652099	0.488183
C	-3.33147	0.850438	-0.09743
C	-4.15471	2.644845	-1.20809

N	-3.18482	3.480006	-0.833
N	-3.32584	-0.53845	0.049922
C	-2.29483	-1.2169	0.703726
N	-2.11322	-2.4432	0.054874
C	-3.1442	-2.66417	-0.96549
C	-4.07569	-1.42635	-0.84937
N	3.71787	-0.18434	0.283072
C	4.492293	-0.96831	-0.68546
C	3.901617	-2.39963	-0.58363
N	2.705055	-2.2051	0.250196
C	2.715074	-0.95544	0.881481
C	1.516367	-2.90107	0.006215
N	1.615931	-3.95396	-0.82792
C	0.457393	-4.49033	-1.2134
N	-0.77432	-4.06093	-0.93526
C	-0.84189	-3.01531	-0.09021
C	0.290121	-2.45469	0.505435
N	-4.27135	1.346872	-0.92504
O	1.971376	-0.597	1.771383
O	-1.6622	-0.80896	1.656998
O	0.00839	2.448712	1.781656
H	1.819847	1.463526	1.348155
H	5.021882	3.532314	-1.61866
H	-1.27005	5.81927	-0.2173
H	-1.22978	4.742636	-1.61588
H	1.136261	4.742974	-1.58809
H	-1.63647	1.258754	1.190871
H	-4.93084	3.067385	-1.8427
H	-2.67921	-2.7424	-1.95038
H	-3.67231	-3.59948	-0.77473
H	-4.16802	-0.92806	-1.81652
H	4.375511	-0.54158	-1.6838
H	5.554163	-0.93788	-0.43717
H	3.5798	-2.75219	-1.56529
H	0.525808	-5.3665	-1.85469
H	0.222609	-1.65184	1.216922
C	-5.49901	-1.72571	-0.33756
H	-5.99603	-0.75009	-0.27777
C	1.262653	6.329914	-0.14551
H	2.353688	6.317721	-0.1442
H	0.920123	7.117259	-0.82518
H	0.905719	6.562128	0.862686
C	4.8463	-3.45664	0.01974
H	4.247149	-4.3728	0.078992
C	5.314853	-3.09325	1.432139
H	5.936452	-3.89398	1.843492
H	5.917257	-2.17782	1.438616
H	4.468504	-2.94734	2.108839
C	6.024447	-3.7196	-0.92474
H	6.66895	-2.83877	-1.02449
H	6.646438	-4.53493	-0.54424
H	5.679288	-3.99989	-1.9256
C	-6.24286	-2.56917	-1.3826
H	-5.82838	-3.58088	-1.4588
H	-7.29732	-2.67052	-1.11113
H	-6.195	-2.11108	-2.37627
C	-5.55478	-2.38426	1.057185
H	-4.80957	-3.18845	1.124017
H	-6.52712	-2.88395	1.144838
C	-5.39985	-1.43045	2.248707
H	-4.40042	-0.99907	2.32244
H	-6.11292	-0.60218	2.174657
H	-5.6	-1.9578	3.186701

8.2 Tetramer Coordinates (Figure 3A)

R=iPr

N	-0.68871	-4.64602	-0.28316
C	-0.19785	-5.95903	-0.70194
C	1.315576	-5.8954	-0.39875
N	1.542436	-4.44976	-0.24308
C	0.351866	-3.74501	-0.05894
O	0.240966	-2.57172	0.239254
C	2.805376	-3.86679	-0.32665
N	3.811341	-4.71281	-0.62931
C	5.010766	-4.15073	-0.77363
N	5.328789	-2.86042	-0.67192
C	4.308099	-2.03805	-0.358
C	3.005411	-2.49592	-0.1452
C	1.75182	-6.68272	0.855382
C	1.61723	-8.18952	0.609659
C	1.008412	-6.24256	2.120812
N	4.646241	-0.68891	-0.28341
C	5.959419	-0.19795	-0.70151
C	5.895502	1.315496	-0.39844
N	4.449748	1.542206	-0.24336
C	3.745017	0.351588	-0.05964
O	2.571534	0.240549	0.237763
C	3.866785	2.805171	-0.32674
N	4.712853	3.811122	-0.62932
C	4.150864	5.010598	-0.77352
N	2.86058	5.328708	-0.67173
C	2.038168	4.308056	-0.35781
C	2.49593	3.005297	-0.14518
C	6.682285	1.751924	0.855937
C	8.18923	1.617919	0.610693
C	6.241987	1.008245	2.121168
N	0.689048	4.646214	-0.28315

C	0.198017	5.959188	-0.70179
C	-1.31547	5.895213	-0.3989
N	-1.54206	4.449545	-0.24298
C	-0.35135	3.745067	-0.05868
O	-0.24012	2.571895	0.239851
C	-2.80499	3.866517	-0.32639
N	-3.81079	4.712486	-0.62971
C	-5.01022	4.150469	-0.77422
N	-5.32837	2.860244	-0.67214
C	-4.30789	2.037902	-0.3574
C	-3.00526	2.495744	-0.14415
C	-1.75221	6.682658	0.854957
C	-1.61746	8.189441	0.609153
C	-1.00934	6.242554	2.120729
N	-4.64622	0.688835	-0.28261
C	-5.95901	0.197863	-0.70191
C	-5.89538	-1.31554	-0.39866
N	-4.44975	-1.54228	-0.24246
C	-3.74526	-0.35165	-0.05771
O	-2.57216	-0.24059	0.241179
C	-3.86667	-2.80515	-0.32618
N	-4.71258	-3.81107	-0.62929
C	-4.15042	-5.01042	-0.77394
N	-2.86012	-5.32839	-0.67217
C	-2.03783	-4.30778	-0.35774
C	-2.49579	-3.00517	-0.14456
C	-6.68315	-1.7518	0.855183
C	-8.1899	-1.61777	0.608716
C	-6.24385	-1.00797	2.120658
H	2.201649	-1.8301	0.110619
H	2.815953	-6.45692	0.981943
H	2.012188	-8.75287	1.460127
H	2.170453	-8.49896	-0.2834
H	0.57104	-8.48895	0.479333
H	1.390089	-6.78508	2.990879
H	1.138911	-5.17399	2.313662
H	-0.06605	-6.4461	2.054484
H	-0.70735	-6.74757	-0.14906
H	-0.40353	-6.11465	-1.76539
H	6.747715	-0.70736	-0.1482
H	6.115614	-0.40366	-1.76488
H	6.250326	1.900614	-1.24857
H	4.827553	5.829127	-1.01085
H	1.900735	-6.24998	-1.24894
H	1.830032	2.201589	0.110619
H	6.456082	2.815955	0.98252
H	8.752156	2.013135	1.461327
H	8.498731	2.17125	-0.28228
H	8.48912	0.571857	0.480494
H	6.783841	1.390274	2.991493
H	6.446261	-0.0661	2.054963
H	5.173243	1.138039	2.313479
H	0.707252	6.74772	-0.14865
H	0.403914	6.115045	-1.76516
H	-1.90052	6.249473	-1.2493
H	-5.82863	4.827112	-1.01207
H	-2.20168	1.829971	0.112422
H	-2.8164	6.456925	0.981106
H	-2.01282	8.752881	1.459376
H	-0.57121	8.488842	0.479301
H	-2.17025	8.498798	-0.2842
H	-1.3914	6.785097	2.990612
H	0.065153	6.446115	2.054872
H	-1.13989	5.173992	2.313546
H	-6.74783	0.707338	-0.1494
H	-6.11419	0.40347	-1.76544
H	-6.24959	-1.90073	-1.24897
H	-4.82698	-5.82892	-1.01173
H	-1.83007	-2.20146	0.111685
H	-6.45703	-2.81583	0.982059
H	-8.75351	-2.01293	1.458914
H	-8.4987	-2.17112	-0.28448
H	-8.48968	-0.57171	0.478231
H	-6.7866	-1.38971	2.990565
H	-5.17532	-1.13799	2.313987
H	-6.44781	0.066403	2.054029
H	5.829316	-4.82737	-1.01102

R=Me, carbonyls on same face

C	0.445327	-6.47917	-0.52522
N	-0.78866	-5.99606	-0.37773
C	-0.86174	-4.70498	0.000145
C	0.269647	-3.92465	0.248629
C	1.497251	-4.54307	0.00121
N	1.601607	-5.83204	-0.37655
N	2.708783	-3.86015	0.107665
C	2.823212	-2.47596	0.2625
N	4.166019	-2.15474	0.062331
C	5.002613	-3.34886	-0.05909
C	3.974148	-4.46873	-0.31134
N	-2.15531	-4.19404	0.1056
C	-3.32534	-4.96966	-0.31446
C	-4.49739	-4.00109	-0.06218
N	-3.832	-2.70387	0.060504
C	-2.45797	-2.8386	0.261141
C	-4.5258	-1.49622	0.055857
C	4.688069	-0.86353	0.056777
C	3.869497	0.265677	0.126561
C	4.525821	1.496225	0.055629
N	5.864262	1.604123	-0.0559

C	6.52798	0.448359	-0.10115
N	6.028689	-0.78733	-0.05475
N	-5.86418	-1.60411	-0.05641
C	-6.52788	-0.44835	-0.10191
N	-6.02861	0.787329	-0.05523
C	-4.68805	0.863539	0.057004
C	-3.86952	-0.26568	0.12731
N	3.831983	2.703842	0.060024
N	-4.16604	2.154755	0.062769
C	-5.00261	3.348845	-0.05905
C	-3.97413	4.468663	-0.31151
N	-2.70878	3.860154	0.107618
C	-2.82323	2.476012	0.26286
C	2.457945	2.838537	0.260718
N	2.155298	4.194016	0.105496
C	3.325337	4.969609	-0.31464
C	4.497349	4.001142	-0.06193
C	0.861728	4.704976	0.000103
C	-0.26966	3.924632	0.248525
C	-1.49726	4.543079	0.00122
N	-1.60162	5.832086	-0.37643
C	-0.44534	6.479219	-0.52508
N	0.788653	5.996095	-0.37765
O	-1.92441	1.700245	0.520924
O	1.673747	1.94721	0.519242
O	1.924375	-1.70012	0.520316
O	-1.67377	-1.94734	0.519875
H	0.517294	-7.52584	-0.81382
H	0.199059	-2.89732	0.554707
H	5.71579	-3.23489	-0.87522
H	4.178637	-5.36976	0.267137
H	3.920366	-4.75375	-1.36671
H	-3.23254	-5.244	-1.36995
H	-5.03867	-4.23586	0.859963
H	-5.21904	-3.98512	-0.87867
H	2.802639	0.192345	0.231313
H	7.610103	0.522716	-0.18774
H	-7.60996	-0.5227	-0.18902
H	-2.80272	-0.19236	0.232644
H	-5.71573	3.234661	-0.87519
H	-3.92034	4.753478	-1.36694
H	-4.17862	5.369817	0.266778
H	3.232659	5.243596	-1.37024
H	5.219489	3.985473	-0.87799
H	5.038029	4.235734	0.860617
H	-0.19907	2.897266	0.554498
H	-0.5173	7.525916	-0.8136
C	3.437707	6.270829	0.501329
H	4.469312	6.492365	0.679128
H	2.989391	7.074236	-0.04498
H	2.932492	6.149979	1.436772
C	-5.80136	3.571451	1.238662
H	-5.86761	4.620001	1.44128
H	-6.78556	3.167176	1.125485
H	-5.30589	3.081843	2.050875
C	-3.43789	-6.27063	0.501895
H	-4.46952	-6.49201	0.679705
H	-2.98962	-7.07424	-0.04414
H	-2.93271	-6.14954	1.437329
C	5.801248	-3.57112	1.23875
H	5.869259	-4.61972	1.440535
H	6.784781	-3.16502	1.126289
H	5.304598	-3.08305	2.05117

R=Me, one carbonyl on different face

C	4.829627	4.348502	-0.51284
N	5.217443	3.080826	-0.37857
C	4.241131	2.204533	-0.06707
C	2.914212	2.595113	0.134598
C	2.643766	3.950469	-0.06989
N	3.599019	4.844861	-0.3931
N	1.357931	4.480646	0.020605
C	0.195509	3.733271	0.220793
N	-0.88014	4.601858	0.029642
C	-0.44508	6.00122	-0.11442
C	1.06198	5.84755	-0.41261
N	4.641571	0.871572	-0.0038
C	6.031326	0.441478	-0.23083
C	5.863288	-1.0629	-0.53901
N	4.521288	-1.36618	-0.03851
C	3.790939	-0.20826	0.235735
C	3.978521	-2.64638	-0.13245
C	-2.21608	4.214621	-0.05444
C	-2.6222	2.89	0.127754
C	-3.97836	2.63565	-0.09314
N	-4.85889	3.602267	-0.41925
C	-4.34949	4.829886	-0.51546
N	-3.08032	5.203996	-0.35917
N	4.853845	-3.61008	-0.47934
C	4.342252	-4.8366	-0.58641
N	3.072224	-5.21066	-0.43163
C	2.212941	-4.22097	-0.11813
C	2.624133	-2.9037	0.093646
N	-4.52042	1.353276	-0.02338
N	0.876677	-4.60064	0.006699
C	0.444349	-6.00708	0.062538
C	-1.07366	-5.89994	-0.21101
N	-1.3612	-4.48219	0.010967
C	-0.19586	-3.71117	-0.01195
C	-3.79051	0.190482	0.226807

N	-4.64403	-0.88444	-0.02705
C	-6.03437	-0.44864	-0.2368
C	-5.86446	1.059495	-0.52487
C	-4.24562	-2.21713	-0.10428
C	-2.91914	-2.60791	0.091799
C	-2.64704	-3.9612	-0.11887
N	-3.60257	-4.86016	-0.42462
C	-4.8344	-4.36337	-0.5416
N	-5.22181	-3.09458	-0.41359
O	-0.13234	-2.49793	-0.04126
O	-2.63441	0.12901	0.597213
O	0.134898	2.552761	0.503669
O	2.636824	-0.15334	0.613063
H	5.612216	5.063894	-0.7577
H	2.144541	1.892555	0.39815
H	-0.974	6.446642	-0.95787
H	1.668821	6.564512	0.141195
H	1.293905	5.961396	-1.4764
H	6.428202	0.981817	-1.0912
H	6.609122	-1.67499	-0.03082
H	5.920797	-1.28164	-1.61014
H	-1.92963	2.110875	0.389818
H	-5.05441	5.622385	-0.7588
H	5.045898	-5.62726	-0.83893
H	1.934325	-2.12539	0.364066
H	0.950111	-6.56107	-0.72967
H	-1.33606	-6.18317	-1.23502
H	-1.66098	-6.5196	0.468105
H	-6.43945	-0.97572	-1.10166
H	-5.92407	1.292751	-1.59285
H	-6.60775	1.666003	-0.0066
H	-2.14926	-1.9025	0.344932
H	-5.61848	-5.08017	-0.77722
C	-6.89834	-0.72933	0.991209
H	-7.92186	-0.38289	0.816302
H	-6.92255	-1.80095	1.195289
H	-6.50026	-0.20619	1.866554
C	-0.7261	6.80407	1.154217
H	-0.38183	7.835525	1.028895
H	-1.79762	6.817237	1.360467
H	-0.20185	6.364803	2.008901
C	6.904174	0.707487	0.994038
H	7.927013	0.365649	0.806531
H	6.927788	1.77634	1.21224
H	6.514017	0.171936	1.865428
C	0.771558	-6.63015	1.417682
H	0.434237	-7.67102	1.444158
H	1.849385	-6.6118	1.590618
H	0.271699	-6.08116	2.221862

R=H, flat

C	-4.22109	-5.02914	-0.04444
N	-4.7658	-3.81101	-0.03775
C	-3.88773	-2.7894	-0.00791
C	-2.50574	-2.98291	0.018494
C	-2.07385	-4.31007	0.002975
N	-2.92664	-5.35286	-0.02818
N	-0.72072	-4.64429	0.017667
C	0.325337	-3.72244	0.02417
N	1.515057	-4.44946	0.012706
C	1.291613	-5.89508	0.019647
C	-0.25066	-6.02901	-0.0229
N	-4.45196	-1.51499	-0.00594
C	-5.89749	-1.29193	-0.0321
C	-6.03247	0.250471	0.005493
N	-4.64725	0.720606	-0.00942
C	-3.72515	-0.32513	-0.00135
C	-4.31255	2.073595	0.000921
C	2.789837	-3.886	0.010874
C	2.984569	-2.50402	-0.00514
C	4.312277	-2.07365	-0.00161
N	5.354538	-2.92754	0.012749
C	5.029594	-4.22174	0.023038
N	3.810862	-4.76517	0.023564
N	-5.35468	2.927709	0.009805
C	-5.02946	4.221856	0.017488
N	-3.8106	4.765017	0.019458
C	-2.7897	3.885642	0.011909
C	-2.98474	2.503652	0.000193
N	4.646923	-0.72065	-0.01358
N	-1.51491	4.449121	0.014389
C	-1.29186	5.894835	0.014885
C	0.25066	6.029295	-0.01841
N	0.720838	4.644477	0.016364
C	-0.32499	3.72242	0.024607
C	3.724854	0.325137	-0.01093
N	4.451736	1.514956	-0.0099
C	5.897351	1.291851	-0.02923
C	6.032058	-0.25055	0.008894
C	3.887631	2.789424	-0.01032
C	2.505626	2.98305	0.01366
C	2.073975	4.310324	0.002172
N	2.926927	5.353111	-0.02435
C	4.221361	5.029244	-0.0394
N	4.765885	3.811018	-0.03518
O	-0.21935	2.511816	0.038907
O	2.514151	0.219626	-0.01084
O	0.220314	-2.51178	0.038112
O	-2.51449	-0.2195	0.006798
H	-4.91997	-5.86284	-0.06754

H	-1.81663	-2.16021	0.042282
H	1.776135	-6.35738	-0.84288
H	1.724886	-6.34024	0.918571
H	-0.64575	-6.58851	0.827568
H	-0.603	-6.52077	-0.93287
H	-6.33079	-1.72729	-0.93573
H	-6.37092	-1.77476	0.825389
H	-6.54178	0.60409	0.905203
H	-6.57547	0.644227	-0.85626
H	2.162532	-1.81373	-0.01501
H	5.862728	-4.92161	0.033158
H	-5.86245	4.921936	0.02356
H	-2.1629	1.813103	-0.00548
H	-1.73117	6.344905	0.908311
H	-1.77093	6.352112	-0.85345
H	0.607921	6.526778	-0.92323
H	0.640942	6.583566	0.837791
H	6.366677	1.774612	0.830541
H	6.334996	1.727222	-0.93077
H	6.579772	-0.64417	-0.84993
H	6.536413	-0.60434	0.911327
H	1.81631	2.160403	0.033667
H	4.920399	5.862906	-0.05883
